# The Role of Selection in Shaping Diversity of Natural *M. tuberculosis* Populations

**DOI:** 10.1371/journal.ppat.1003543

**Published:** 2013-08-15

**Authors:** Caitlin S. Pepperell, Amanda M. Casto, Andrew Kitchen, Julie M. Granka, Omar E. Cornejo, Eddie C. Holmes, Bruce Birren, James Galagan, Marcus W. Feldman

**Affiliations:** 1 Departments of Medicine and Medical Microbiology and Immunology, School of Medicine and Public Health, University of Wisconsin-Madison, Madison, Wisconsin, United States of America; 2 Department of Biology, Stanford University, Stanford, California, United States of America; 3 Department of Anthropology, University of Iowa, Iowa City, Iowa, United States of America; 4 Department of Genetics, Stanford University School of Medicine, Stanford, California, United States of America; 5 Sydney Emerging Infections and Biosecurity Institute, School of Biological Sciences and Sydney Medical School, University of Sydney, Sydney, Australia; 6 The Broad Institute, Cambridge, Massachusetts, United States of America; 7 Department of Biomedical Engineering and Microbiology, Boston University, Boston, Massachusetts, United States of America; University of Massachusetts, United States of America

## Abstract

*Mycobacterium tuberculosis* (*M.tb*), the cause of tuberculosis (TB), is estimated to infect a new host every second. While analyses of genetic data from natural populations of *M.tb* have emphasized the role of genetic drift in shaping patterns of diversity, the influence of natural selection on this successful pathogen is less well understood. We investigated the effects of natural selection on patterns of diversity in 63 globally extant genomes of *M.tb* and related pathogenic mycobacteria. We found evidence of strong purifying selection, with an estimated genome-wide selection coefficient equal to −9.5×10^−4^ (95% CI −1.1×10^−3^ to −6.8×10^−4^); this is several orders of magnitude higher than recent estimates for eukaryotic and prokaryotic organisms. We also identified different patterns of variation across categories of gene function. Genes involved in transport and metabolism of inorganic ions exhibited very low levels of non-synonymous polymorphism, equivalent to categories under strong purifying selection (essential and translation-associated genes). The highest levels of non-synonymous variation were seen in a group of transporter genes, likely due to either diversifying selection or local selective sweeps. In addition to selection, we identified other important influences on *M.tb* genetic diversity, such as a 25-fold expansion of global *M.tb* populations coincident with explosive growth in human populations (estimated timing 1684 C.E., 95% CI 1620–1713 C.E.). These results emphasize the parallel demographic histories of this obligate pathogen and its human host, and suggest that the dominant effect of selection on *M.tb* is removal of novel variants, with exceptions in an interesting group of genes involved in transportation and defense. We speculate that the hostile environment within a host imposes strict demands on *M.tb* physiology, and thus a substantial fitness cost for most new mutations. In this respect, obligate bacterial pathogens may differ from other host-associated microbes such as symbionts.

## Author Summary

*Mycobacterium tuberculosis* (*M.tb*), the etiologic agent of tuberculosis (TB), is a highly prevalent pathogen of humans, estimated to infect one-third of the world's population. Previous investigations of *M.tb* evolution have emphasized the influence of chance events on populations of these bacteria. To understand why this organism is so well adapted to its niche, we sought to characterize the influence of natural selection on *M.tb*. DNA sequence data from *M.tb* populations appeared to be strongly influenced by genome-wide selection against deleterious mutations. This type of selection was particularly evident in three functional categories: genes essential for infection (identified in an animal model of TB), genes involved in protein translation, and genes involved in trafficking and metabolism of inorganic ions. By contrast, a fourth category (‘defense’ genes) exhibited high levels of diversity, consistent with selection for advantageous mutations. In addition to effects of selection on *M.tb* genomic data, we identified the influence of a pronounced recent expansion in *M.tb* populations, coincident with explosive growth of human populations around the world. Our results suggest that growth of *M.tb* populations parallels that of its human host population, and that complex influences lead to emergence and maintenance of adaptive traits in *M.tb*.

## Introduction

*Mycobacterium tuberculosis* (*M.tb*) is among a group of highly virulent bacteria that exhibit extremely low levels of population genetic diversity [1]. The adaptive influences shaping patterns of genetic diversity among these organisms are not well understood. In comparisons with free-living bacteria, relaxation of purifying selection has been invoked to explain features such as an elevated ratio of non-synonymous to synonymous single nucleotide polymorphisms (SNPs) [2]. It is not clear whether the pathogenic lifestyle permits a broader range of new non-synonymous mutations, relative to existence outside a host. Alternatively, there may be a reduction in genetic effective population size (*N_e_*) associated with restriction to the pathogenic niche, and consequently a reduction in the efficiency of selection against deleterious mutations. Positive selection is also clearly playing a role in ongoing evolution of *M.tb*, as shown by the progressive emergence of multiply, extensively, and now totally drug resistant TB [3]. Detailed characterization of the evolutionary constraints on natural populations of *M.tb* would allow development of TB control strategies that explicitly account for ongoing bacterial adaptation. Our goal here was to test *M.tb* population genomic data for signatures of both positive and negative selection, to identify regional differences in the strength and type of selection on the *M.tb* genome, and to quantify fitness effects of new mutations among natural populations of *M.tb*. We performed these analyses on whole genome sequence data from a globally representative sample of *M.tb* and related pathogenic mycobacteria.

## Results

**Figure 1:**
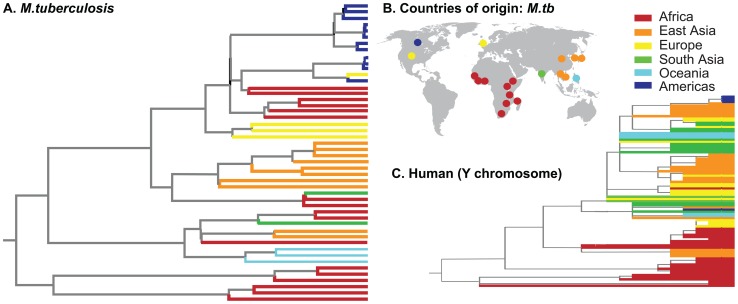
Geographic and genetic structure of global sample of *M.tb* genomes. A) Maximum clade credibility phylogeny inferred from genome-wide *M.tb* SNP data using BEAST [83]. Tips are colored by the geographic origin of the *M.tb* isolate (see key). Descriptions of the 48 *M.tb* isolates shown here are in **Table S1**. B) Countries of origin for *M.tb* isolates used in this study are shown as colored dots on global map. One dot is shown per country but some countries were represented by >1 *M.tb* isolate. Colors correspond to global regions (see key). C) Phylogeny of global human populations from [91], based on Y chromosome data. Tips are colored according to the same scheme as the *M.tb* phylogeny (A).

Countries of origin for *M.tb* isolates included in this study and a Bayesian phylogeny based on *M.tb* genomic data are shown in **Figure 1**, along with a global human phylogeny based on Y chromosome data (Accession numbers are in **Table S1**). The overall genetic structure of the global *M.tb* population inferred here is similar to previously published studies (e.g. [2]), and has led to speculation that differentiation of continental *M.tb* populations is driven by adaptation to distinct human sub-populations [4]. However, global phylogeographic structures of human (**Fig. 1C**) and *M.tb* (**Fig. 1A**) populations are not congruent, which suggests that co-divergence with continental human populations has not been a major influence on diversification of *M.tb*. Similarly, regressions of *M.tb* divergence times on human divergence times failed to identify a strong signature of co-divergence, and there was no statistical support for co-divergence of *M.tb* with its host in formal tests of phylogenetic congruence (**Text S1**, **Table S2** and **Figures S1** and **S2**, SOM).

The results of the McDonald-Kreitman (MK) tests of selection pressure based on genome-wide patterns of synonymous and non-synonymous variation are shown in **Table 1**. These indicate purifying selection during divergence of *M.tb* from *Mycobacterium marinum, Mycobacterium ulcerans* and *Mycobacterium leprae* (p = 0, chi-square). Alignments of *M.tb* and *Mycobacterium canettii* reveal a large number of sites that are segregating in *M. canettii* and fixed in *M.tb*, relative to comparisons between more distantly related species. Most of the *M. canettii* polymorphisms are synonymous (polymorphisms in *M. canettii*: 19,262 non-synonymous *vs* 39,912 synonymous; polymorphisms in *M.tb*: 5,500 non-synonymous *vs* 3,279 synonymous). In comparisons of *M.tb* with *Mycobacterium bovis* (both of which are within the *Mycobacterium Tuberculosis* Complex, MTBC), the pattern of polymorphisms and divergences is consistent with neutral evolution.

**Table 1:** Results of the McDonald-Kreitman test on whole genome sequences of *M.tb* and related mycobacteria.

Comparison		d_N_/d_S_ a	p_N_/p_S_ b	p-value^c^
*M.tb*	*M.ulcerans*	0.5 (84265/186102)	1.5 (3551/2359)	0
*M.tb*	*M.marinum*	0.5 (99679/183427)	1.5 (3748/2459)	0
*M.tb*	*M.leprae*	0.4 (49477/112509)	1.3 (1931/1447)	0
*M.tb*	*M.canettii*	0.6 (900/1572)	0.6 (24762/43191)	NS
*M.tb*	*M.bovis*	1.5 (199/130)	1.7 (5857/3517)	NS

a d_N_ = fixed non-synonymous difference, d_S_ = fixed synonymous difference.

b d_N_ = non-synonymous polymorphism, d_S_ = synonymous polymorphism.

c chi-square test.

**Figure 2:**
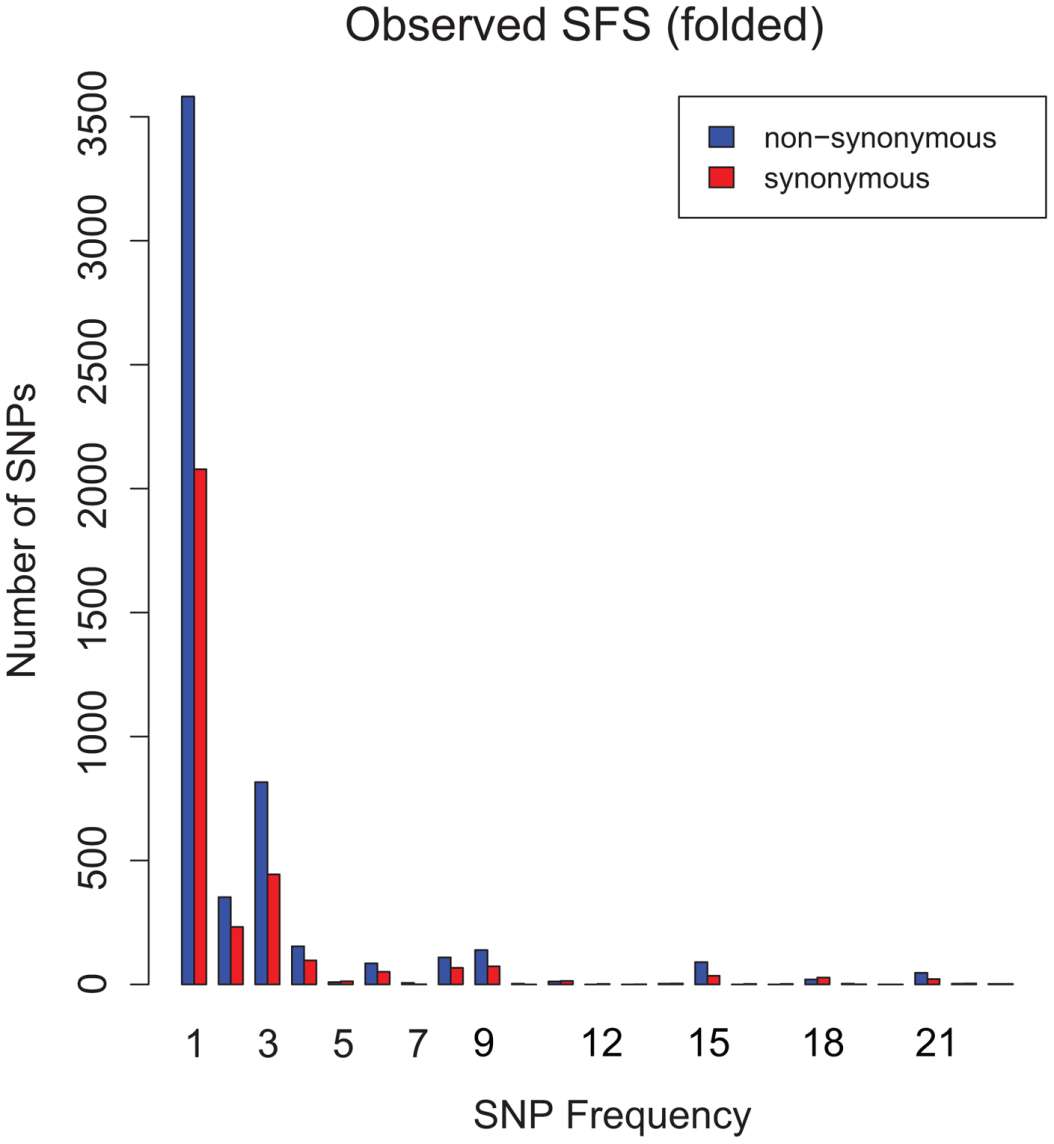
Observed folded site frequency spectrum (SFS) of synonymous and non-synonymous SNPs. Numbers of single nucleotide polymorphisms (SNPs, Y axis) in frequency classes 1–23 (X axis). The SFS is leptokurtic and bumpy, consistent with purifying selection and linkage of sites (see text).

**Figure 3:**
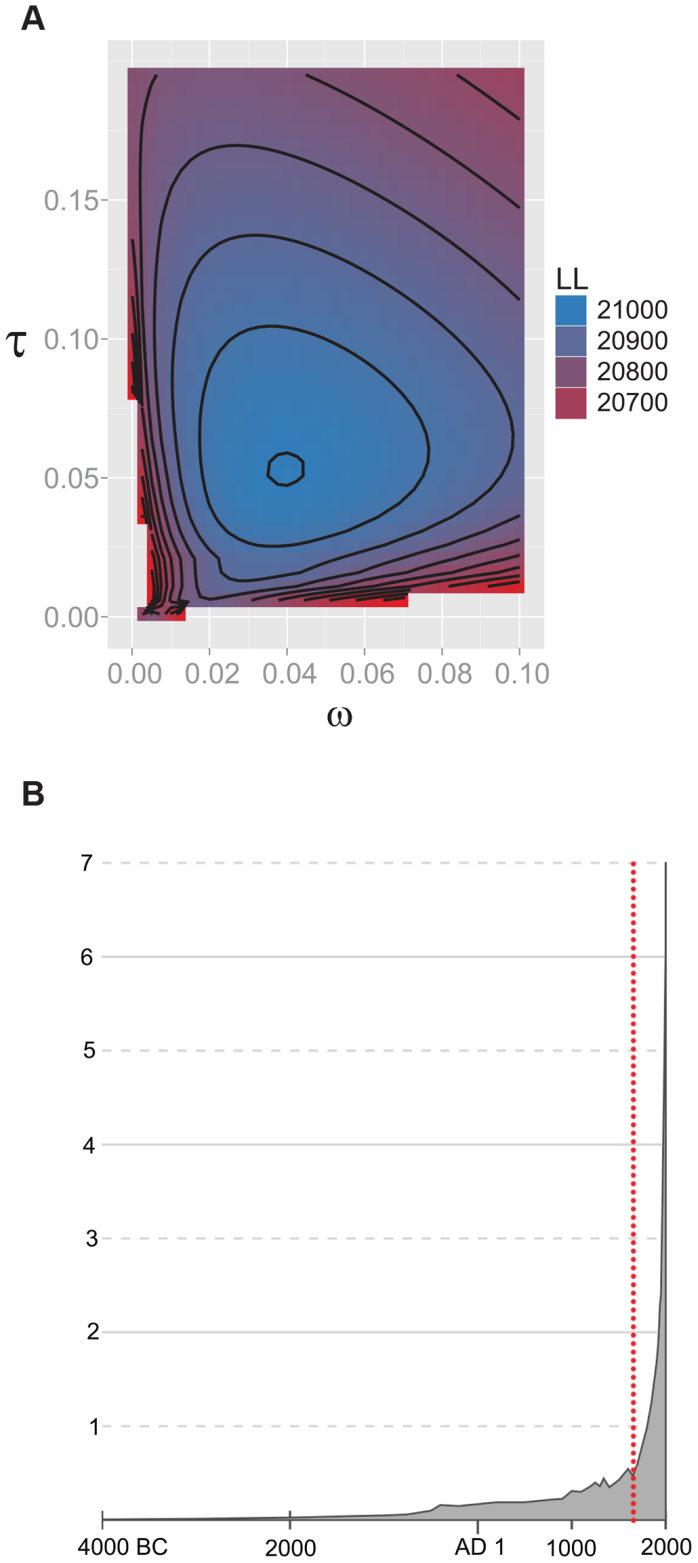
A) Heatmap of likelihoods: demographic inference. Heatmap of log_10_ likelihood values over a grid of values for two demographic parameters: τ = generations since expansion/*N_e_* (Y axis) and ω = *N_anc_/N_e_* (X axis) where *N_anc_* is the effective size of the ancestral population prior to expansion and *N_e_* is the effective size of the current population. Log_10_ likelihood (LL) values of the data given various parameter values are indicated on the color key. There is a well demarcated peak in likelihood values. B) Historical growth of human and *M.tb* populations. Historical patterns of growth of human populations are shown in the gray curve, with calendar years on the X axis and size of global human population in billions on the Y axis. Data and image from http://en.wikipedia.org/wiki/World_population. The estimated timing of expansion of the global *M.tb* population is shown as a red dotted line (instantaneous expansion model, see text).

The folded site frequency spectrum (SFS) from the global sample of *M.tb* is shown in **Figure 2**: the SFS is leptokurtic, with an irregular decay in the frequency of minor alleles as the counts increase. Results of analyses of the SFS using *prfreq* [5] are shown in **Table 2**. The likelihood of the observed synonymous SFS was estimated for constant size, instantaneous expansion and exponential models of growth. The best fit growth model is of a sudden expansion [p<0.001, likelihood ratio test (LRT), comparison with constant size model] with an estimated timing in calendar years of 1684 C.E. (95% CI 1620–1713 C.E.). The likelihood surface for the data given different demographic parameters in the sudden expansion model shows a well-demarcated peak around the two maximum likelihood estimates (timing and magnitude of expansion, **Figure 3A**). **Figure 3B** shows the estimated timing of expansion of the global *M.tb* population in relation to historic growth of global human populations.

**Table 2:** Estimates of demographic and selection parameters from the site frequency spectrum.

Parameter	Value
No. of isolates	47
θ^a^ = 2*N_e_*μ	1230
Models	
LL^b^ of neutral constant size	36,400
LL of neutral expansion	38,925 (p = 0)^c^
ω = *N_anc_/N_e_* (95%CI)^d^	0.04 (0.035–0.045)
τ = generations^e^/*N_e_* (95%CI)	0.051 (0.046–0.061)
LL of expansion+point mass^f^	38,943 (p<0.001)
γ = 2*N_e_*s (95%CI)	−5.83 (−4.17 to −6.67)
LL of expansion+pt mass+neutral^g^	39,025 (p = 0)
*p_0_* (95%CI)	0.05 (0.05)^h^
γ = 2*N_e_*s (95%CI)	−1900 (−1200 to −1900)

a Watterson's estimate of θ, see *Methods*.

b Log likelihood of observed SFS given the demographic/selection model.

c Likelihood ratio test for comparison with next less complex model.

d *N_anc_* = genetic effective size of population prior to instantaneous expansion; 95% confidence intervals reported here are based on 10,000 bootstrapped parameter estimates, see *Methods*.

e Generations elapsed since expansion.

f Model includes a single selection coefficient (*s*) at all sites in the genome.

g Model includes a category of neutral sites (fraction = p_0_) and a second category (1-p_0_) with selection coefficient *s*.

h Bootstrapped estimates all equal to 0.05.

**Figure 4:**
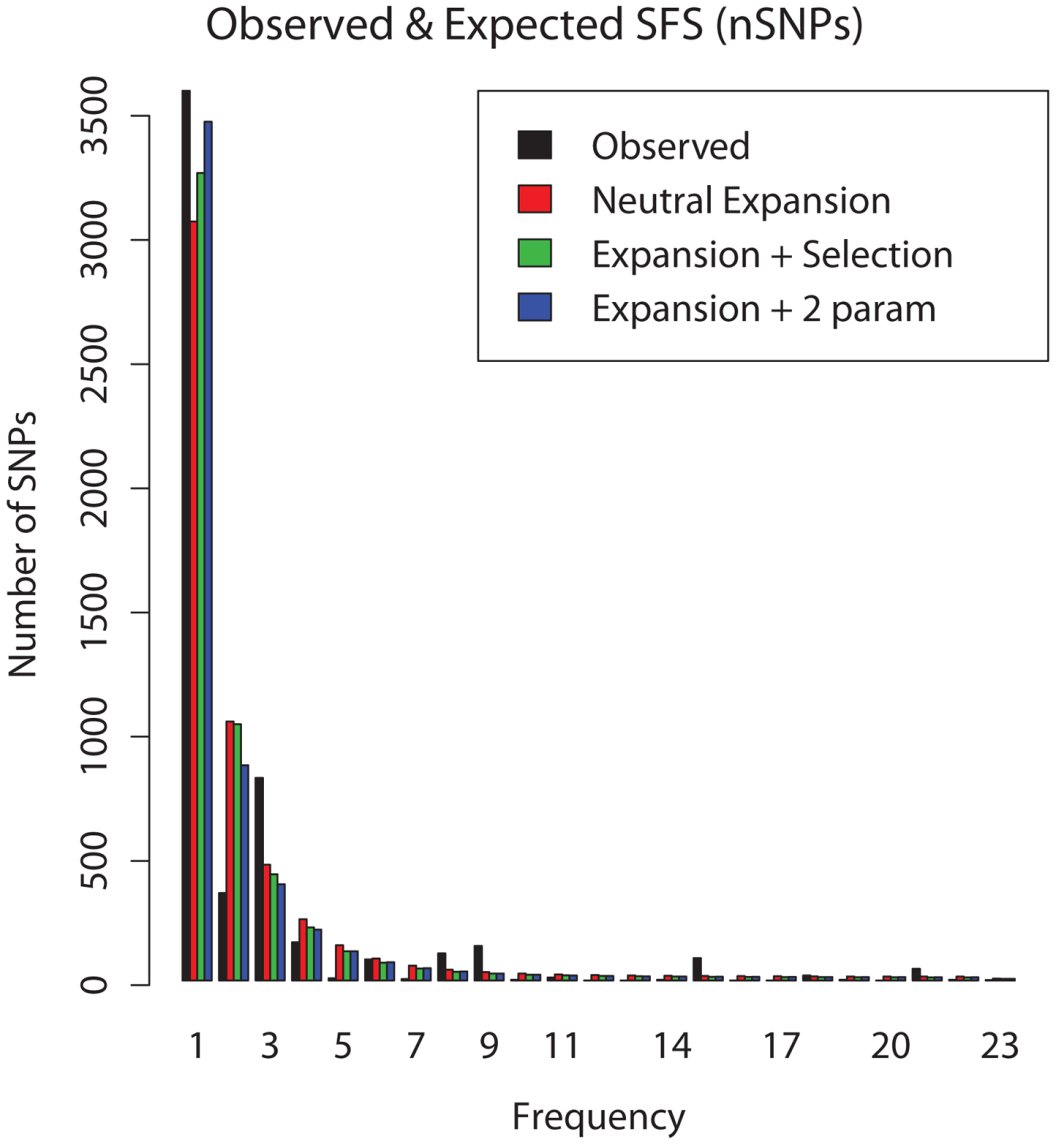
Expected site frequency spectrum (SFS) under various demographic and selective models. The folded SFS for non-synonymous SNPs: observed values are shown in black, and expected values under different models are shown in colors. Expected SFS for an instantaneous past expansion is shown in red, expansion plus a single selection coefficient at all sites is shown in green, and expansion plus two coefficients of selection (one negative and the other zero) is shown in blue. The improved fit of the two parameter selection model appears to be driven primarily by the large number of singleton SNPs in the observed data.

**Figure 5:**
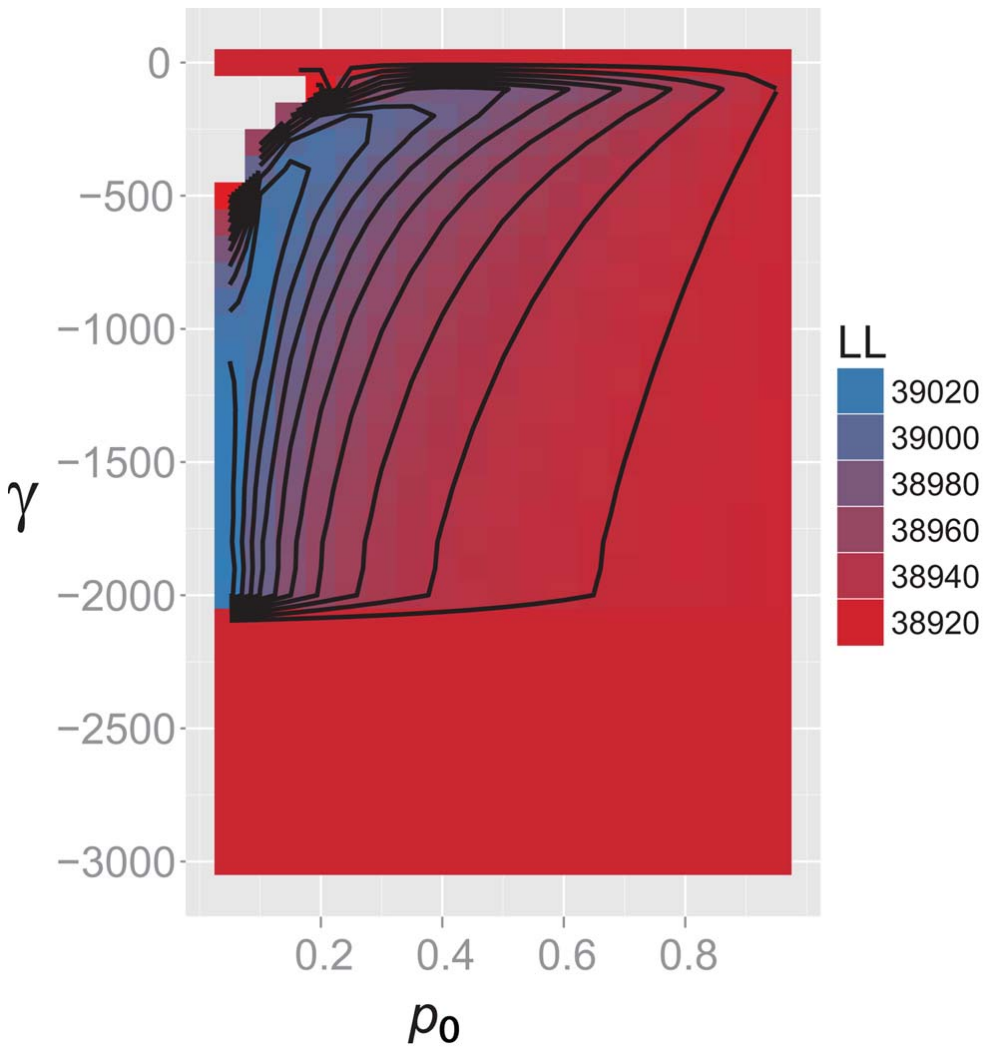
Heatmap of likelihoods: inference of selection. Heatmap of likelihood values over a grid of parameter values for neutral plus selected sites model. The proportion of neutral sites (*p_0_*) is shown on the X axis; the composite parameter γ (2*N_e_s*) is shown on the Y axis. There is a steep ridge of likelihoods at low values of *p_0_*.

Models that included the effects of natural selection in addition to demography were also examined. The simplest model of selection – a single negative selection coefficient *s* at all sites in the genome - was a significant improvement over the neutral expansion model (p<0.001, LRT) with a maximum likelihood estimate of *s* = −9.5×10^−4^ (95% CI −1.1×10^−3^ to −6.8×10^−4^). A two parameter model of selection, which includes a fraction *p_0_* of neutral sites, offered an even better fit to the data (Δ log likelihood relative to single parameter model = 82, p = 0, LRT). Improved fit of this model appeared to be driven primarily by the large number of singletons observed (**Figure 4**). Maximum likelihood estimates of the two parameters in this model indicated that the majority of sites in the genome were under very strong purifying selection (*p_0_* = 0.05; *s* = −0.31, 95%CI −0.31 to −0.19). Purifying selection of this magnitude affecting 95% of sites is unexpected, and the likelihood surface for the two parameters is unusual, with a steep ridge at values of *p_0_* less than 0.2 (**Figure 5**).

Several studies have shown that the combination of purifying selection and strong linkage of sites results in a skew in the site frequency spectrum towards rare frequencies [6]–[12]. We did not find any evidence of lateral gene transfer among *M.tb* isolates in our sample (see *Methods*), which is consistent with many studies indicating predominantly clonal evolution of *M.tb* [2], [13]–[16]. Given this apparent lack of lateral gene transfer and known effects of linkage on the SFS, we hypothesized that implausible results obtained with the two parameter model of selection may have been affected by complete or near-complete linkage of sites. To explore this hypothesis, we simulated the SFS under complete linkage and estimated coefficients of selection from these simulated data (see *Methods* for details). For the initial simulation, we simulated purifying selection of equal strength at all sites (*s_sim_*). Simulated SFS were then used for model selection (neutral versus purifying selection) and estimation of a selection coefficient using *prfreq* [5], as we did with the observed data. In 9,857/10,000 (99%) simulations, the “point mass” model of purifying selection (i.e. single selection coefficient) was favored over the neutral model (p<0.05, LRT). Estimates of the selection coefficient from simulated data (*s_est_*) were very similar to the value that was included in the simulation (*s_sim_* = −0.005, median *s_est_* = −0.003, 95%CI −0.005 to −0.001 from 10,000 simulations). With the point mass model and a single non-recombining locus, the method therefore provided an accurate estimate of the strength of purifying selection in these simulations.

**Figure 6:**
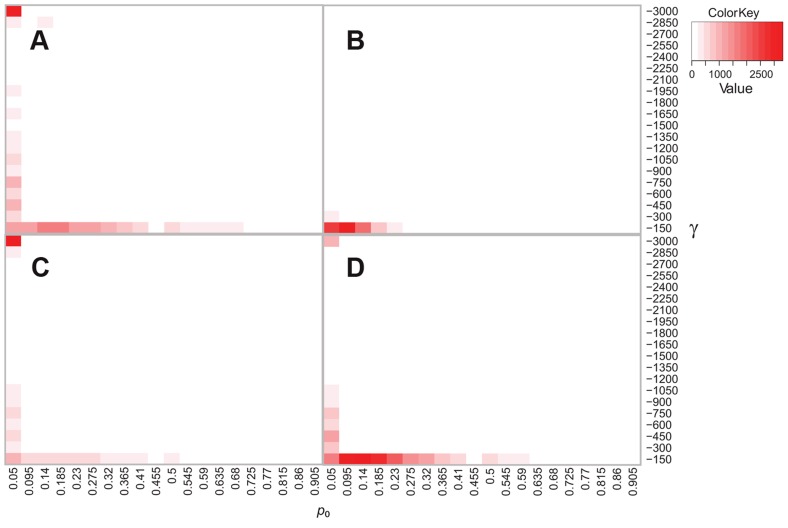
Simulation and inference under purifying selection and complete linkage. Results of four sets of simulation experiments (10,000 simulations/set). In all cases, a single completely linked locus of length equal to 2,753,618 bp was simulated under purifying selection, and inference of selection was done with a two parameter model (category of neutral sites plus single selection coefficient at remaining sites). The composite parameter γ ( = 2*N_e_s*) and proportion of neutral sites (*p_0_*) were estimated from the simulated data. These are shown on the Y and X axes of each panel, respectively. The number of counts of simulations with estimates within each grid value is indicated in the color key. A) Simulated γ = 1, *p_0_* = 0; B) Simulated γ = 10, *p_0_* = 0; C) Simulated γ = 1, *p_0_* = 0.9; D) Simulated γ = 10, *p_0_* = 0.9. Simulations of relatively weak purifying selection (panels A & C) paradoxically result in inference of extremely strong purifying selection (γ∼−3,000) in a large proportion of cases. This pattern disappears when stronger selection is simulated (panel B). Even when 90% of sites are evolving neutrally, purifying selection is inferred at a large proportion of sites (panels C & D), likely due to linkage of sites.

We then simulated the SFS in an identical manner, but used a two-parameter model of selection (*p_0_* and *s*) in the estimation of the magnitude of selection from the data. This model was favored over the neutral model in 7190/10,000 (72%) of simulations. Results from these simulations indicate that the data are consistent with a wide range of parameter values, with the highest count of values at extremely strong selection and few neutral sites (**Figure 6A**). Interestingly, this pattern was less evident when we simulated stronger purifying selection: the range of parameters estimated from the data is smaller and more accurate (i.e. *s_est_* is lower, **Figure 6B**). When we included a category of neutral sites in the simulation, the same pattern of improved performance with stronger versus weaker selection was observed (**Figures 6C & D**). The proportion of neutral sites was not estimated accurately in the presence of linkage.

Taken together, simulation experiments using the two parameter model of selection suggest the model performs poorly in the presence of weak purifying selection and complete linkage but appears to perform better with stronger purifying selection.

In our data, the observed pattern of inference of extremely strong purifying selection and a small number of neutral sites (**Figure 5**) is consistent with results from simulations of relatively weak purifying selection. This pattern was seen with simulations of both large and small proportions of neutral sites (**Figure 6**), suggesting that either scenario could hold true for our observed data.

**Figure 7:**
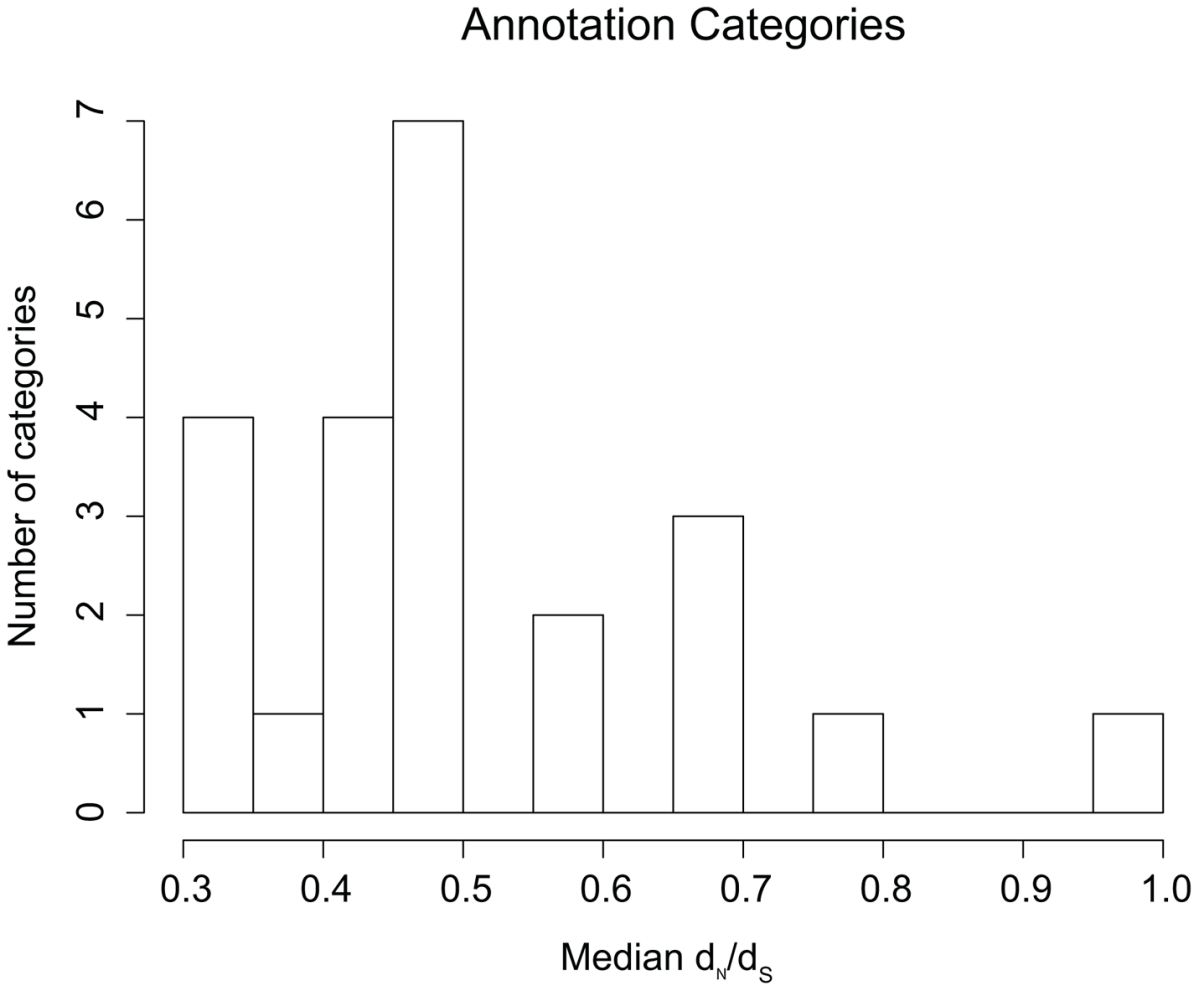
Median d_N_/d_S_ values for pairwise comparisons among 47 *M.tb* isolates. Observed median d_N_/d_S_ values for pairwise comparisons among 47 isolates of *M.tb*. Median d_N_/d_S_ values are shown on the X axis, while the number of COG (annotation) categories with each median value is shown on the Y axis.

**Figure 8:**
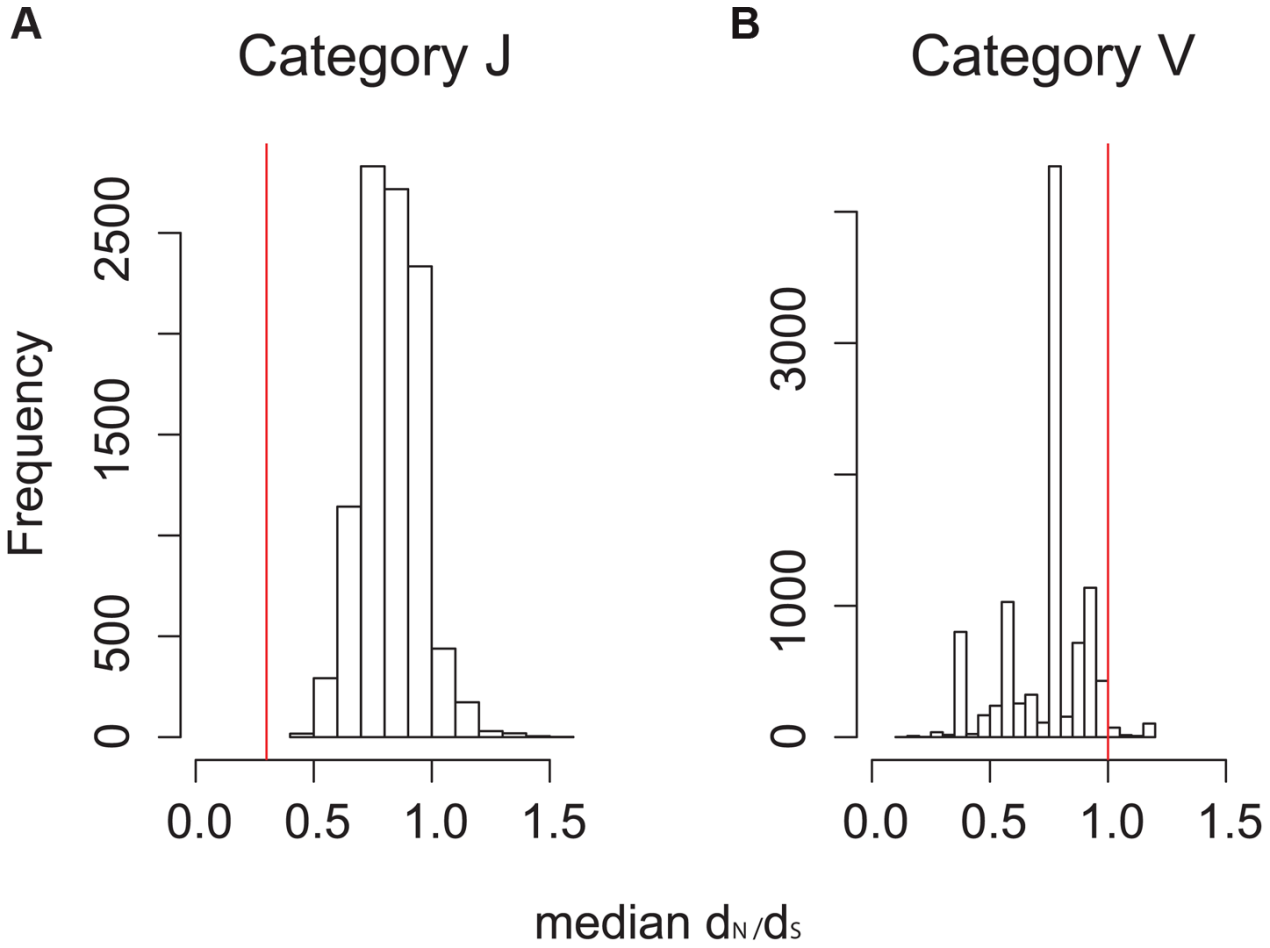
Null distributions of median d_N_/d_S_ for two COG categories. Distributions of median d_N_/d_S_ from 10,000 simulations in which synonymous and non-synonymous sites within the COG category were shuffled randomly. Red lines show observed median d_N_/d_S_ value for the category. **A**: category J, translation and ribosomal structure. **B**: category V, defense.

It is possible that the distribution of fitness effects of mutations differs among genes and gene categories within the *M.tb* genome. To identify potential effects of gene function on the type of selection manifest in patterns of genetic diversity, we looked for differences in proportions of synonymous and non-synonymous polymorphisms among gene annotation categories. For each of 23 gene categories, and for all pairs of clinical *M.tb* isolates (total 47), we estimated the ratio of non-synonymous to synonymous polymorphisms (d_N_/d_S_, see *Methods* for details). Distributions of pairwise d_N_/d_S_ varied widely among clusters of orthologous groups (COG) categories, with median values ranging from 0.3 to 1 (**Figure 7** **and Table S3**). We hypothesized that COG categories at the edges of this distribution contained genes under purifying (left edge) and positive selection (right edge). To determine whether the edge values differed from a null/neutral model, we performed a permutation procedure for each COG category. For each group of genes, synonymous and non-synonymous sites were shuffled randomly in the 47 bacterial isolates, pairwise d_N_/d_S_ were estimated, and summary statistics calculated from these pairwise values (procedure repeated 10,000 times, more details in *Methods*). We replicated the finding of an earlier study of a subset of *M.tb* T cell antigens that found them to be under purifying selection [17]: T cell antigens were at the left edge of the d_N_/d_S_ distribution, with a median pairwise value of 0.33 (p = 0.0001). The null distribution of median d_N_/d_S_ values from two COG categories are shown in **Figure 8**. Category J, “translation, ribosomal structure and biogenesis”, has an observed median d_N_/d_S_ value of 0.30 (left edge of distribution); this is outside the null distribution for the category (p = 0, **Figure 8A**). The same was true for the other two categories at the left edge of the observed distribution (category P, “inorganic ion transport and metabolism”, observed median = 0.33, p = 0; category of genes found essential *in vivo* [18], observed median = 0.33, p = 0). Interestingly, median d_N_/d_S_ of genes essential for growth *in vitro* [19] were in the middle of the distribution of observed values (median = 0.5, **Table S1**), suggesting that purifying selection on these genes may be less stringent relative to genes that are only essential in an animal model of TB. Category V, “defense”, has an observed median value of one (right edge of observed distribution); this value is in the right tail of the null distribution (p = 0.03, **Figure 8B**). This suggests that genes in category V may be under positive selection, and categories J and P, as well as *in vivo* essential genes, are under purifying selection.

## Discussion

### Neutral and selective influences on an obligate pathogen

Recent analyses of the differentiation of bacterial pathogens and symbionts from closely related, free-living species invoke “relaxed purifying selection” to explain observed differences in genome size, d_N_/d_S_ and GC content [2], [20], [21]. This points to a decrease in the strength and/or efficiency of selection against deleterious mutations in host-associated bacteria. The efficiency of selection scales with the effective size of a population (*N_e_*) and takes time to become evident; it follows that the effects of purifying selection will be less obvious in small, recently diverged populations than in larger, older populations even if the two groups have identical coefficients of selection (*s*) [22]. Kryazhimskiy et al. identified a further property of d_N_/d_S_, namely its low power to estimate the strength of selection from patterns of polymorphism among closely related organisms [23].

It is not clear whether the apparent weakness of purifying selection among bacterial pathogens is due to a small *N_e_*, recent origin, or relaxation of selective constraints during transition to within-host environments (due for example to less competition and predation or more stable abiotic factors). We have previously identified numerous features of TB disease ecology that could reduce the *N_e_* of *M. tuberculosis*: bottlenecks associated with epidemic expansion, fractured transmission networks that lead to the isolation of bacterial sub-populations, and patchy patterns of migration among populations of hosts [24], [25].

Some investigators have argued that the demographic history of human populations – particularly the out of Africa migrations of human sub-populations [4], [26] – has driven adaptive evolution among lineages of *M.tb*. However, we find no evidence to suggest an adaptive influence of human population structure on patterns of *M.tb* differentiation. Congruence analyses of human and *M.tb* phylogenies were not consistent with human-*M.tb* co-divergence, which is a prerequisite for co-adaptation. Correlation between divergence times of *M.tb* and human sub-populations was also weak relative to bacteria with compelling evidence of co-divergence, such as *H.pylori* (**Text S1**, **Table S2** and **Figures S1** and **S2**, SOM). Indeed, patterns of differentiation between pathogenic mycobacteria with different host species ranges (*M.tb* and *M. bovis*) did not show evidence of adaptive evolution (**Table 1**). In our opinion, human demography has strong effects on evolution of *M.tb*, in that bacterial population growth is constrained by the size and connectivity of host populations, but these effects are largely selectively neutral. Consistent with this hypothesis, we find evidence here of a recent 25-fold expansion in the global *M.tb* population, coincident with recent explosive growth in human populations around the world (**Figure 3B**) [27], [28].

Along with recent expansion in parallel with human populations, our analyses identify purifying selection as an additional influence on the site frequency spectrum of global populations of *M.tb* (**Table 2**, **Figure 4**). We also find evidence of purifying selection in the pattern of genetic differences that are fixed relative to closely related species of mycobacteria (**Table 1**), and in the d_N_/d_S_ of gene categories where we expect purifying selection (ribosomal proteins, essential genes). These observations are consistent with a recent analysis of 22 *M.tb* genomes, where the authors identified higher d_N_/d_S_ values in terminal versus internal branches of the phylogenetic tree, which is consistent with purifying selection during evolution of the sampled bacterial population [29].

### Strength of purifying selection and the effect of linkage

Our estimates of the common selection coefficient (*s*) across all non-synonymous sites in the genome, and the composite parameter γ ( = 2*N_e_s*) are −9.5×10^−4^ and −5.83, respectively. Values of *s* estimated recently for autosomal mutations among two species of *Drosophila* ranged from −2×10^−5^ to −8×10^−6^ (harmonic mean values [30]), while mean *s* for non-synonymous mutations segregating among humans has been estimated at −1×10^−4^ [5]. There have been few attempts to quantify the strength of purifying selection on bacteria; one example is the cavity-causing bacterium *Streptococcus mutans*, for which estimates of *s* and γ are −3.4×10^−6^ and −17 [31], respectively.

*M.tb* is among a group of pathogenic bacteria characterized by low sequence diversity and clonal evolution [1], [32], [33], although there is evidence of lateral gene transfer (LGT) having occurred during divergence of the pathogenic mycobacteria and some investigators have argued that it has occurred more recently [29], [34]–[37]. We did not find any evidence of LGT among the *M.tb* isolates included in our sample (see *Methods*). The method we used to estimate the strength of selection from the site frequency spectrum assumes free recombination [5]. Previously published simulation studies indicate that the method actually performs well in the presence of linkage [5]. Our own simulations indicate that the method used in [5] accurately captures the features of a simple model – a single, negative selection coefficient at all sites – for a genome with complete linkage. However, the same was not true of a more complex model, which included a category of neutral sites. The strength of selection included in the simulation was not inferred accurately using the two-parameter model, most commonly being overestimated by an order of magnitude or more. These simulations of a single, completely linked locus under purifying selection corresponded well with our observed data, where we saw a marked discrepancy between selection coefficients estimated with one versus two-parameter models.

Interestingly, when a stronger selection coefficient was simulated, the selection coefficient estimated with the two parameter model decreased (i.e. became more accurate). The combination of weak purifying selection and linkage can result in interference between deleterious alleles, and extreme distortions of patterns of neutral variation [38]. It is possible that these effects account for the paradoxical results and inference from our simulations with the two-parameter model of selection.

The combination of purifying selection and linkage (background selection) has complex effects on the genetics of populations (reviewed in [39]). Among these effects is a reduction of *N_e_* and overall genetic variation, as well as distortions of gene genealogies that result in an excess of rare variants. Large numbers of singleton SNPs have been observed consistently in the *M.tb* genomics literature [40], suggesting a broad influence of background selection on patterns of diversity among *M.tb*. The comparison with *M. canettii* (**Table 1**) is illustrative, as we observed large numbers of neutral polymorphisms segregating among *M. canettii*, which appears to undergo frequent recombination [41]. There were fewer polymorphisms segregating among *M.tb*, and these were predominantly non-synonymous. The contrast between *M.tb* and *M. canettii* emphasizes the important role of LGT (and other forms of inter-genomic recombination) in generating diversity and purging deleterious variants.

We have demonstrated here that inference of selection from *M.tb* genomic data is strongly affected by linkage of sites, in ways that are not always intuitive, nor predictable. These effects should be explored in analyses of genomic data from *M.tb* and other clonal pathogens, where they are likely to be important.

Our estimate of the genome-wide selection coefficient on *M.tb* is higher than average estimates for humans, *Drosophila* and a pathogenic species of *Streptococcus*. Caution is warranted in direct comparisons of these estimates, which reflect distinct data sources, sampling strategies, methods of inference as well as differences in ploidy and mode of reproduction. For example, whereas average human, *Drosophila* and *S.mutans* estimates of *s* are based on distributions of fitness effects (DFE) of new mutations, the strong linkage of sites in the *M.tb* genome limited us to inferences based on a single selection coefficient across all sites (although it is possible that a flat distribution is a reasonable approximation of the DFE in the absence of recombination).

Despite these caveats, it is unlikely that the selection coefficient in *M.tb* is *weaker* than in the few organisms for which we have comparable estimates. This suggests that the relaxation of purifying selection apparent among *M.tb* relative to free-living organisms is due to a decrease in *N_e_*, rather than a decrease in the strength of selection against deleterious mutations associated with the pathogenic lifestyle. In an analysis of several species of yeast, Elyashiv et al recently demonstrated that genome-wide measures of the strength of purifying selection scale with proxies of *N_e_* [42]. This suggests that differences in the apparent strength of purifying selection among these yeast are also related to differences in *N_e_*, rather than *s*. It is worth noting that evolutionary constraints on intracellular bacterial pathogens, which subsist in hostile environments in an antagonistic relationship with their hosts, may be qualitatively and quantitatively distinct from constraints on bacterial symbionts, notwithstanding the commonality of reduced *N_e_* relative to free living organisms.

### Variation of d_N_/d_S_ among gene annotation categories

As noted above, the d_N_/d_S_ statistic is not ideally suited to analysis of polymorphisms segregating among closely related organisms [23]. While the general properties of d_N_/d_S_ hold for population polymorphisms – namely d_N_/d_S_<1 is indicative of negative selection and d_N_/d_S_>1 of positive selection – its magnitude may not be informative of the strength of natural selection. Given these limitations, we elected to compare relative values of d_N_/d_S_ among genes in the *M.tb* genome. [17]. The following gene annotation categories had the lowest median values of d_N_/d_S_: *in vivo* “essential” genes (p = 0), genes encoding ribosomal proteins (p = 0), genes involved in the transport and metabolism of inorganic ions (p = 0), as well as a subset of T cell antigens previously shown to be under purifying selection (p = 0.0001) [17]. The *in vivo* “essential” gene category consists of all *M.tb* genes that have been identified by systematic mutagenesis experiments as essential for establishing infection in an animal model of tuberculosis [18]. Category J, “translation, ribosomal structure and biogenesis”, contains 121 genes encoding ribosomal and other proteins involved in translation. *In vivo* essential and translation associated genes are expected to be under strong purifying selection and their identification supports the validity of our method, as does our replication of results from an earlier study of *M.tb* T cell antigens. Interestingly, d_N_/d_S_ for two distinct types of essential genes – those essential for growth *in vitro* and those not essential for *in vitro* growth but required for survival in an animal model – were in different parts of the distribution of annotation categories. *In vivo* essential genes were at the low end and *in vitro* essential grouped in the middle of the distribution. This suggests that *in vivo* essential genes are under stronger and/or more uniform purifying selection, and points to the specificity of constraints imposed in *M.tb*'s natural environment.

Genes in COG category P – “inorganic ion transport and metabolism” – also appear to be under strong purifying selection. This category contains 123 genes involved in the transport and metabolism of iron, magnesium, molybdenum, nickel, arsenic, potassium, sulfur and other elements. Bacterial genes under strong purifying selection are attractive targets for antimicrobial drugs, since their products are likely to have critical and central roles in cellular metabolism. Relative to loci under relaxed evolutionary constraint, there may also be fewer mutational pathways available to permit the acquisition of drug resistance. Indeed, category P contains two existing antibiotic targets, *katG*, targeted by isoniazid [43] and *ethA*, which is a target of ethionamide [44]. Further studies of these groups of genes may yield new targets for the treatment of tuberculosis. More generally, the approach outlined here could be used to complement existing methods of identifying novel antibiotic targets for a range of pathogens.

### *M.tb* genes under diversifying selection

We identified a single category of genes, Category V (**Figure 8B**) for which the distribution of pairwise d_N_/d_S_ was higher than observed in neutral simulations (median d_N_/d_S_ = 1, p = 0.03). COG category V, “defense mechanisms”, contains 39 genes that encode efflux pumps of which the majority are ABC transporters (n = 19), genes with beta-lactamase and penicillin binding protein domains as well as other antibiotic resistance genes, restriction modification system genes, genes involved in the assembly of immunomodulatory cell surface lipids (phthiocerol dimycocerosate aka PDIM), and other genes of unknown function thought to be involved in lipid metabolism [45] (Note that the functions of these genes are unknown/incomplete in many cases). Twenty three of the genes in category V encode proteins known to be located in the bacterial cell membrane [45]. ABC transporters, which make up the largest group of genes in category V, have been associated with a broad range of functions in *M.tb*. These functions include: drug efflux [46] and resistance to other toxic agents [47], iron trafficking [48], [49], and transport and localization of PDIM to the cell surface [50], [51].

Genes in category V could have high pairwise values of d_N_/d_S_ relative to other categories as a result of relaxation of purifying selection, local selective sweeps (positive selection under a regime of restricted migration), or some form of diversifying selection. The importance of the functions ascribed to genes in this category makes relaxation of purifying selection unlikely. For example, PDIM must be properly localized by its associated ABC transporter in order for *M.tb* to maintain cell surface integrity [51] and survive in macrophages [50].

Selective sweeps have been observed in antimicrobial resistance genes of malarial parasites [52], [53]. Given the evidence of restricted migration of *M.tb* [24], positively selected mutations are likely to have elevated measures of non-synonymous variation across bacterial sub-populations, as we observed for this category of genes. Note that as discussed above, genes under strong purifying selection are attractive targets for antimicrobials. However, once any genetic barriers to resistance have been overcome, resistance-conferring mutations are likely to be positively selected and may leave the signature of a selective sweep. Genetic loci associated with drug resistance in *M.tb* were recently shown to have elevated measures of diversity, consistent with localized selective sweeps [54]. However, mutations in category V genes may also be advantageous for reasons other than classically defined drug resistance. For example, recent work identified a role for *M.tb* efflux pumps in persistence, an incompletely understood, adaptive phenotype in which bacteria escape immune and drug-mediated killing [55].

Numerous studies of bacterial pathogens, including *M.tb*, have identified high levels of variability (elevated d_N_/d_S_ and/or recombination hotspots) among genes involved in interactions with the host [54], [56]–[67]. High levels of standing variation among pathogen genes are usually thought to result from selection by host immunity, e.g. negative frequency dependent selection [68], although population genetic data often do not conform to the predictions of simplified models of host-pathogen interactions. Indeed, studies of *M.tb* antigens have demonstrated that some subsets appear to be under purifying selection whereas others are highly diverse [17], [67]. Many of the products of category V genes are cell surface associated and involved in interactions with the host; they may be under diversifying selection, as appears to be the case for similar genes from other pathogens. In particular, ABC transporter genes have been associated with high d_N_/d_S_ in studies of uropathogenic *E.coli* [58] and *Microbotryum* [69] (a fungal pathogen of plants). Although none of the genes in category V are known to encode antigens, they could be involved in the generation of antigenic diversity via their role in the transport and localization of antigens to the microbial cell surface. Heterogeneity of cell surfaces among populations of pathogenic bacteria may also be adaptive for other reasons, for example by allowing bacterial sub-populations to bind in distinct micro-environments within the host [70]. Comparative studies of *M.tb* gene subsets under distinct evolutionary regimes, be they antigens or cell-surface associated, may provide insight into the complex interactions between *M.tb* and its human host. Further studies are needed to define the functions of genes in this category and their potentially important role in reciprocal evolutionary dynamics between populations of humans and *M.tb*.

### Conclusions

In previous work we identified complex neutral influences on the structure of *M.tb* populations. Here we find evidence in complete DNA sequences from a global sample of *M.tb* of both purifying and positive selection. Elucidation of these neutral and selective influences on patterns of genetic variation among *M.tb* provides insight into the process by which pathogenicity evolves and may lead to new avenues of discovery in the search for better drugs, vaccines and control strategies to combat tuberculosis.

## Methods

### Data set

We analyzed published genome sequence data from *Mycobacterium marinum* (1 isolate), *Mycobacterium ulcerans* (1 isolate), *Mycobacterium leprae* (2 isolates), *Mycobacterium canettii* (6 isolates), *Mycobacterium bovis* (5 isolates), and a globally representative sample of *M.tb* (47 clinical isolates plus H37Rv). Names and accession numbers of the 63 bacterial isolates analyzed in this study are in **Table S1**. The rationale for studying genetic diversity among *M.tb* relative to *M. canettii*, *M. leprae*, *M.ulcerans* and *M. marinum* is that they are the mycobacteria most closely related to MTBC [71]. Genome sequence data from *M. ulcerans* are described in [72], [73] *M. marinum* data are described in [74], and *M. canettii* data in [41]. The *M.tb* global diversity genome sequence data set included in our analyses is described in [17]; further descriptions and data are also available at *tbdb.org* [45]. The *M.tb* strains included in the global diversity project were chosen to provide a broad perspective on diversity among globally extant bacteria, and to sample major lineages of *M.tb*. Phylogenetic analyses of *M.tb* sequencing data – be it whole genome data, SNP data, or gene based phylogenies – have produced congruent definitions of these global lineages [40]. Among the 47 clinical isolates of *M.tb*, we sequenced twelve *M.tb* genomes from Aboriginal communities in Western Canada; the source population is described in [24]. The Canadian *M.tb* strains, which belong to the most commonly circulating lineages in these communities, were chosen to maximize temporal diversity of the sample: both with respect to the date of TB diagnosis (which ranged from 1986 to 2004) and age of the patient. For time periods from which we had multiple *M.tb* strains, we chose the strain randomly from among the available options. Estimated *M.tb* strain acquisition dates (based on patient age, history of TST, and diagnosis date) span the twentieth century. These strains are all susceptible to first line anti-TB agents. Where available, drug susceptibility data for other *M.tb* strains are shown in **Table S1**. *M.tb* genomes were sequenced using 454 titanium whole genome shotgun methodology (454 Life Sciences) to a coverage of 15× from 180 bp fragments and 30× from 3 Kb jumps. Sequence reads were assembled with Newbler (454 Life Sciences).

The *M.tb* sample included in this study was designed to provide an in-depth perspective from a single population (the Canadian sample) as well as a broad view of genetic diversity among globally extant lineages of *M.tb*. Although we could have included more *M.tb* strains from the expanding public repositories of genomic data, we chose this sample to study because these strains are very well characterized, and beyond inclusion of a modest number of individuals, increasing sample size is a very inefficient means of increasing the accuracy of coalescent based analyses [75].

### Genome sequence alignments

Bacterial genome sequences were annotated using Kodon (v 3.62, Applied Maths). Prior to their alignment, the following elements were removed from individual annotated sequences: transposable elements, phage elements, and repetitive families of genes that are prone to homoplasies, and are poorly resolved with re-sequencing technologies (PE and PPE genes). We used Kodon to annotate any genome sequences for which the annotation was not already available. A list of excluded elements and their coordinates in H37Rv are in the SOM. *M.tb* genome sequences were aligned to the Sanger sequenced laboratory reference strain H37Rv using Kodon.

### McDonald-Kreitman tests

We used Kodon to identify SNPs in alignments of the 47 *M.tb* genomes and comparator mycobacteria (*M. marinum*, *M. ulcerans*, *M. leprae*, *M. canettii* and *M. bovis*/BCG). McDonald-Kreitman tests of selection pressure [76] were conducted using a script in R (2.15.2 [77]).

### Recombination

The 4,100,080 bp long multiple alignment of 47 clinical *M.tb* genomes to H37Rv was found to have 19,009 variable sites, excluding sites defined as variable on the basis of gaps (‘-’) and/or unknown (‘n’) characters. This alignment of 19,009 variable sites was further reduced by eliminating variable loci at which >25% of sequences had a gap (3,926 sites). The resulting alignment (15,083 sites) was scanned for inter-genomic recombination using SplitsTree [78] and Bayesian phylogenetic analysis. There was no statistical support for recombination according to analyses implemented in SplitsTree (Phi = 0.94). We used a sliding window Bayesian phylogenetic approach similar to that implemented in SlidingBayes [79] to identify possible recombinant regions. Phylogenies were estimated in Mr. Bayes (v3.2; [80]) for windows consisting of 200 sites every 50 positions (total 299 windows), with the estimated posterior support for six previously defined *M.tb* groups [i.e., West Africa 1, West Africa 2, Rim of the Indian Ocean, India and East Africa, East Asia, and Europe [2]] calculated at each position in the alignment to identify regions of discordant phylogenetic signal. Six regions of discordant phylogenetic signal were discovered. Gene by gene alignments indicated they resulted from spurious SNPs in focal areas of misalignment. These misaligned regions were removed from the analysis. The alignment was visually inspected and edited by hand to ensure correct alignment and minimize the incorporation of mis-identified substitutions (e.g., incorrect gaps, anomalous insertions or likely mis-identified bases).

### Nucleotide substitution rate

We used two external reference points to calibrate the rate of *M.tb* evolution. We applied the first calibration point to eight of the sequenced *M.tb* isolates from Aboriginal communities in Canada. These isolates have the DS6_Quebec_ deletion and we have shown previously that they were introduced to Western Canadian Aboriginal populations via the fur trade ([25]). We used a truncated normal distribution of dates from 1710–1870 C.E. with mean 1790 C.E. for this calibration. The early bound of this interval derives from historical accounts of the fur trade, which indicate that this is the earliest possible time of contact between Europeans and Aboriginal Canadians from this region. The later bound corresponds to the time when contact between these populations ceased. We placed the mean at the midpoint of this interval (1790); trading activities peaked at the end of the seventeenth century [81], and analyses of minisatellite data are consistent with introduction of the DS6_Quebec_ lineage at this time [25].

The H37Rv reference sequence forms a monophyletic group with a non-DS6_Quebec_ sequence from Canada. Since H37Rv was isolated from a patient specimen in 1905 and has been passaged in laboratories since then [82], we placed a minimum date of 1905 for the divergence of these two isolates.

The variable site alignment was analyzed using the Bayesian Markov Chain Monte Carlo coalescent method implemented in BEAST [83] with the BEAGLE library (v1.0; [84]) to facilitate rapid likelihood calculations. Analyses were performed using multiple substitution, molecular clock, and demographic models. Estimates from the analysis with the highest marginal likelihood (*i.e.*, General Time Reversible with gamma distributed site variation substitution model/uncorrelated log normal distribution of rates/Bayesian Skyline Plot demographic model) are reported here. All model combinations produced similar rate estimates (**Table S4**). Rate estimates made using only the fur trade calibration are consistent with those using both calibrations. All Markov chains were run for at least 100 million generations, sampled every 10,000 generations, and with the first 30 million generations discarded as burn-in; estimated sample size (ESS) values of important parameters were >200 for all analyses.

Our externally calibrated rate analysis produced a mean nucleotide substitution rate estimate of 1.30×10^−7^ substitutions/site/year [95% highest posterior density (HPD) = 8.40×10^−8^–1.81×10^−7^]. The rate is similar to that observed in 14 *M.tb* SNPs from serially sampled laboratory populations of primates (1.19×10^−7^ SNPs/site/year [85]), indicating that estimates do not change with the time-scale of sampling [86], and fall within the range of substitution rates estimated for some other pathogenic bacteria (e.g. *Yersinia pestis* = 2.3×10^−8^ and *Vibrio cholera* = 8.3×10^−7^; [87],[88]).

### Estimates of demographic and selective parameters from the observed site frequency spectrum (SFS)

We used Kodon to generate tables of synonymous, non-synonymous and intergenic SNPs from the alignment of *M.tb* genomes. Loci at which any sequence had a gap or unknown character were removed from the data set. We also removed loci with spurious SNPs in locally misaligned regions identified using SlidingBayes (see ‘Recombination’ section above).

We used the method of Boyko et al. [5], based on Poisson Random Field (PRF) population genetic models, to estimate the strength of purifying selection on our sample of *M.tb* genomes. This method uses the synonymous site frequency spectrum (SFS) to infer the demographic history of the sample. We modeled constant population size, instantaneous expansion and exponential growth, and identified the best-fit model and maximum likelihood parameters of the demographic model given our observed data. This demographic history was then incorporated in models of the non-synonymous SFS to determine whether purifying selection provided further explanatory power for the observed data, and to identify maximum likelihood estimates of selection model parameters.

Watterson's estimator [89] was used to estimate the compound parameter θ ( = 2*N_e_*μ) from segregating sites in the sample. We used the substitution rate estimate (μ) from our calibrated Bayesian phylogenetic analysis to estimate *N_e_* and thus translate compound parameters (τ and γ) estimated from the SFS into individual parameters reported in the results (calendar year of past expansion and selection coefficient).

To estimate bootstrap confidence intervals for our parameter estimates, we resampled the observed synonymous and non-synonymous SFS with replacement. Demographic history and effects of selection were inferred from resampled SFS using the same method as for observed data. Confidence intervals reported here are based on 10,000 cycles of resampling and inference.

We used simulation experiments to explore performance of the Poisson Random Field method in the presence of linkage of sites (the method assumes sites are unlinked). We used SFS_CODE [90] to simulate a single locus of length equal to the number of sites analyzed among the 47 *M.tb* genomes (i.e. total number of sites on the alignment that were free of gaps or unknown bases). We set θ ( = 2*N_e_*μ) per site equal to the value estimated from observed data and simulated the SFS of linked genomes under different regimes of purifying selection. Parameters of demographic and selection models were then inferred using the same methods that were implemented with observed data.

### Analysis of the relative rates of synonymous and non-synonymous substitution per site (d_N_/d_S_)

We analyzed patterns of synonymous and non-synonymous substitution among gene annotation (COG) categories. COG Categories for *M.tb* genes are defined on the TB Database website (www.tbdb.org); COG annotations for genes in H37Rv are also listed in the SOM (**Table S5**). For each COG category, a single file was created containing the concatenated sequences of all genes in that category. Using these files as input, pairwise d_N_/d_S_ values for individual COG categories were then calculated using the program yn00 found in the PAML package (http://abacus.gene.ucl.ac.uk/software/paml.html).

We created null distributions for pairwise d_N_/d_S_ values for each COG category using a perl script and the program yn00. For each COG category, the perl script randomly shuffled the sequences of all 47 samples so that corresponding positions were always sent to corresponding positions (that is, if base pair one in the original sequence of sample A became base pair four in the shuffled version of sequence A, then base pair one of sequence B became base pair four of the shuffled version of sequence B, etc.). This preserved the number of pairwise differences between each sequence pair while changing the codon context of these differences, possibly altering whether they represented synonymous or non-synonymous changes. The full null distributions were created by repeating this shuffling process 10,000 times for each COG category.

## Supporting Information

Figure S1 **Regression of divergences among continental populations of humans and** ***M.tb*** **.** Compared with other bacterial pathogens for which there is a clear pattern of host –pathogen co-divergence, the correlation of *M.tb* lineage divergences with those of associated host populations is weak (see text).
(PDF)

Figure S2 **Tanglegram describing source phylogenies for reconciliation analysis.** Simplified human [92] and *M.tb* phylogenies are shown on the left and right, respectively. Terminal branches of the *M.tb* phylogeny are colored according to the conventions in [2].
(PDF)

Table S1 **Accession numbers of genome sequences.**
(XLSX)

Table S2 **Divergences among continental populations of humans and** ***M.tb*** **.**
(DOCX)

Table S3 **Median values of pairwise d_N_/d_S_ for COG categories, essential genes and T cell antigens.**
(DOCX)

Table S4 **Substitution rate estimates.**
(DOCX)

Table S5 **List of genes in H37Rv with COG annotations.**
(TXT)

Text S1 **Analyses of human-** ***M.tb*** **co-divergence.**
(DOCX)

Text S2 **List of repetitive elements (e.g. transposable elements and mycobacteriophage) and difficult-to-resolve genes (e.g. PE/PPE genes) that were removed from individual bacterial genome sequences prior to their alignment: descriptions and coordinates in H37Rv.**
(PDF)
