# Correction: The Role of Selection in Shaping Diversity of Natural *M. tuberculosis* Populations

**DOI:** 10.1371/annotation/cff22061-44d5-4301-b853-41702d160203

**Published:** 2013-08-15

**Authors:** Caitlin S. Pepperell, Amanda M. Casto, Andrew Kitchen, Julie M. Granka, Omar E. Cornejo, Edward C. Holmes, Bruce Birren, James Galagan, Marcus W. Feldman

The sixth author's name is incorrect. The correct name is: Edward C. Holmes.

