# Supplementary figures and images for: The Role of Selection in Shaping Diversity of Natural M. tuberculosis Populations
Source: PLoS Pathog. 2013 Aug 15;9(8):e1003543. doi: 10.1371/journal.ppat.1003543 (PMC3744410; doi:10.1371/journal.ppat.1003543)

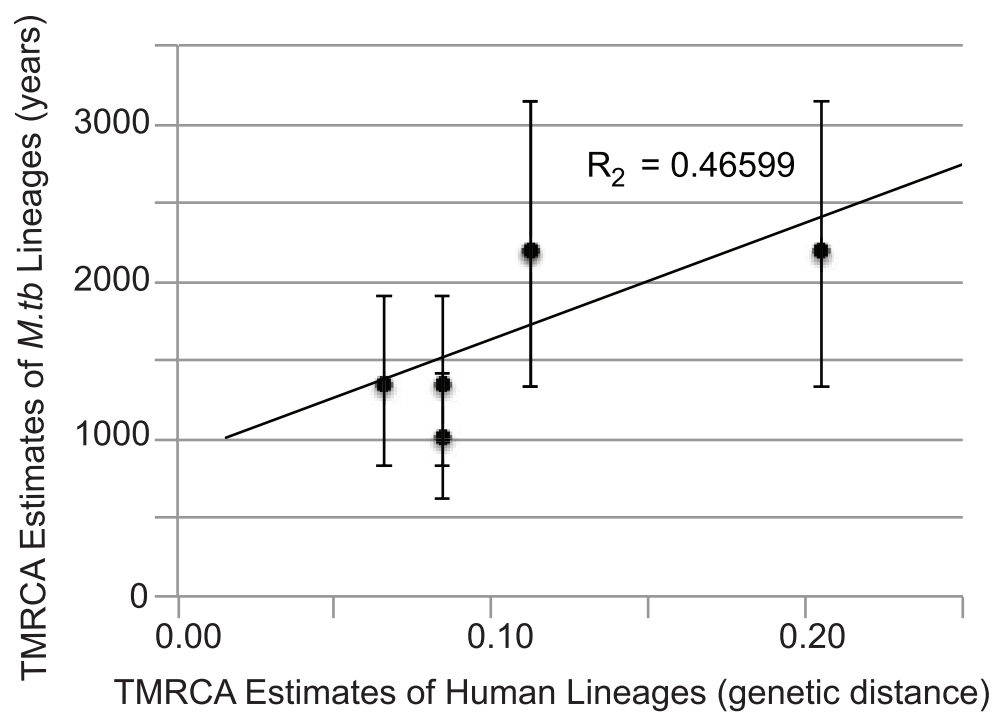

Supplement: Figure S1 — Regression of divergences among continental populations of humans and M.tb . Compared with other bacterial pathogens for which there is a clear pattern of host –pathogen co-divergence, the correlation of M.tb lineage divergences with those of associated host populations is weak (see text). (PDF) [file ppat.1003543.s001.pdf]

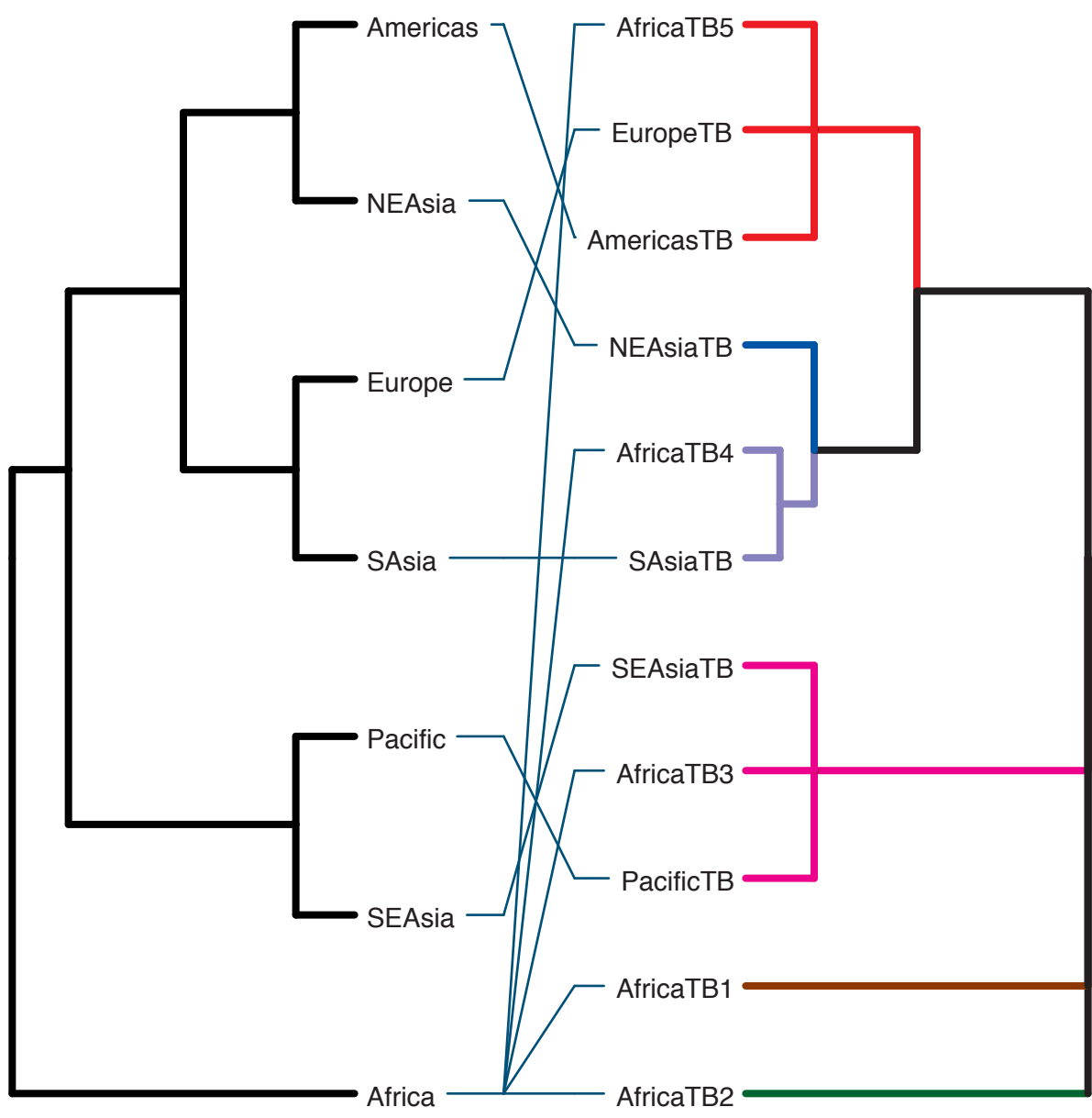

Supplement: Figure S2 — Tanglegram describing source phylogenies for reconciliation analysis. Simplified human [92] and M.tb phylogenies are shown on the left and right, respectively. Terminal branches of the M.tb phylogeny are colored according to the conventions in [2]. (PDF) [file ppat.1003543.s002.pdf]
