# Supplementary material for: The Role of Selection in Shaping Diversity of Natural M. tuberculosis Populations
Source: PLoS Pathog. 2013 Aug 15;9(8):e1003543. doi: 10.1371/journal.ppat.1003543 (PMC3744410; doi:10.1371/journal.ppat.1003543)
Supplement: Table S2 — Divergences among continental populations of humans and M.tb . (DOCX) [file ppat.1003543.s004.docx]

**Table S2. Divergences among continental populations of humans and *M.tb***

| **Population Comparisons** | **Human Calibration^1^** | ***M.tb* TMRCA Estimates^2^** | | |
| --- | --- | --- | --- | --- |
|  |  | Mean | 95% Low | 95% High |
| Africa vs. non-Africa | 0.205 | 2190 | 1331 | 3142 |
| non-Africa TMRCA | 0.112 | 2190 | 1331 | 3142 |
| Northeast vs. Southeast Asia | 0.112 | 2190 | 1331 | 3142 |
| Europe vs. Americas | 0.084 | 1007 | 621 | 1416 |
| Europe vs. Northeast Asia | 0.084 | 1347 | 830 | 1907 |
| Northeast Asia vs. Americas | 0.066 | 1347 | 830 | 1907 |

^1^Genetic distances between human populations taken from Cavalli-Sforza & Feldman 2003 [[1](#_ENREF_1)]

^2^TMRCA estimates for *M.*tb lineages made from the *M.tb* genome phylogeny estimated here by collapsing branches with posterior support < 0.80

1. Cavalli-Sforza LL, Feldman MW (2003) The application of molecular genetic approaches to the study of human evolution. Nat Genet 33 Suppl: 266-275.
