# Supplementary material for: The Role of Selection in Shaping Diversity of Natural M. tuberculosis Populations
Source: PLoS Pathog. 2013 Aug 15;9(8):e1003543. doi: 10.1371/journal.ppat.1003543 (PMC3744410; doi:10.1371/journal.ppat.1003543)
Supplement: Table S3 — Median values of pairwise dN/dS for COG categories, essential genes and T cell antigens. (DOCX) [file ppat.1003543.s005.docx]

**Table S3. Median values of pairwise d_N_/d_S_ for COG categories, essential genes and T cell antigens**

| **Gene category** | **No. of genes** | **Median d_N_/d_S_** |
| --- | --- | --- |
| **COG J** | 121 | 0.30 |
| **Essential *in vivo*** | 193 | 0.33 |
| **COG P** | 123 | 0.33 |
| **T cell antigen** | 78 | 0.33 |
| **COG M** | 104 | 0.40 |
| **COG F** | 63 | 0.42 |
| **Essential (*in vivo* &/or *in vitro*)** | 790 | 0.43 |
| **COG K** | 188 | 0.43 |
| **COG I** | 254 | 0.44 |
| **COG E** | 183 | 0.50 |
| **Essential *in vitro*** | 614 | 0.50 |
| **COG O** | 94 | 0.50 |
| **COG Q** | 234 | 0.50 |
| **COG S** | 208 | 0.50 |
| **COG T** | 97 | 0.50 |
| **COG R** | 436 | 0.50 |
| **COG U** | 15 | 0.60 |
| **COG L** | 194 | 0.60 |
| **COG G** | 111 | 0.67 |
| **COG H** | 119 | 0.67 |
| **COG D** | 28 | 0.67 |
| **COG C** | 210 | 0.80 |
| **COG V** | 39 | 1.00 |
