# Supplementary material for: The Role of Selection in Shaping Diversity of Natural M. tuberculosis Populations
Source: PLoS Pathog. 2013 Aug 15;9(8):e1003543. doi: 10.1371/journal.ppat.1003543 (PMC3744410; doi:10.1371/journal.ppat.1003543)
Supplement: Table S4 — Substitution rate estimates. (DOCX) [file ppat.1003543.s006.docx]

**Table S4. Substitution rate estimates**

| Sub. Model^1^ | Clock Model^2^ | Demo. Model^3^ | Rate Estimate^4^ | Marg. ln(like.)^5^ |
| --- | --- | --- | --- | --- |
| GTR+Γ | UCLN | BSP | 3.13 (2.02 – 4.35) x 10^-5^ | -95484 |
| GTR+Γ | UCLN | Constant | 2.75 (1.83 – 3.70) x 10^-5^ | -95484 |
| GTR+Γ | Strict | BSP | 2.64 (2.16 – 3.12) x 10^-5^ | -96139 |
| GTR+Γ | Strict | Constant | 2.56 (2.09 – 3.03) x 10^-5^ | -96138 |
| GTR | Strict | Constant | 2.56 (2.11 – 3.04) x 10^-5^ | -96134 |
| HKY+Γ | UCLN | BSP | 3.14 (2.07 – 4.33) x 10^-5^ | -95704 |
| HKY+Γ | UCLN | Constant | 2.74 (1.81 – 3.79) x 10^-5^ | -95704 |
| HKY+Γ | Strict | BSP | 2.62 (2.15 – 3.09) x 10^-5^ | -96357 |
| HKY+Γ | Strict | Constant | 2.55 (2.10 – 3.03) x 10^-5^ | -96357 |
| HKY | Strict | Constant | 2.55 (2.09 – 3.03) x 10^-5^ | -96352 |

^1^Substitution models: Hasegawa-Kishino-Yano (HKY); general time reversible (GTR); a gamma distribution of site-specific rate heterogeneity (+Γ).

^2^Molecular clock models: strict clock; relaxed clock with an uncorrelated lognormal distribution of branch-specific rates (UCLN).

^3^Demographic models: constant population size; variable size Bayesian skyline plot model (BSP).

^4^Nucleotide substitution rate estimates: mean (95% highest posterior density) in substitutions per *polymorphic* site per year (sub./site/year). N.B.: the *genome-wide* substitution rate reported in the main text is scaled by the ratio of variable (13,921) to total sites (3,364,004) in the alignment.

^5^Marginal ln(likelihood): The marginal log_e_ likelihood calculated from the posterior distribution of tree likelihoods using the harmonic mean estimator .
