# Supplementary material for: The Role of Selection in Shaping Diversity of Natural M. tuberculosis Populations
Source: PLoS Pathog. 2013 Aug 15;9(8):e1003543. doi: 10.1371/journal.ppat.1003543 (PMC3744410; doi:10.1371/journal.ppat.1003543)
Supplement: Text S1 — Analyses of human- M.tb co-divergence. (DOCX) [file ppat.1003543.s008.docx]

**Text S1. Analyses of human-*M.tb* co-divergence**

Our *M.tb* substitution rate estimate of 1.3 X 10^-7^ subs/site/y is high enough to suggest that the observed genetic diversity of *M.tb* is not correlated with ancient events in human history. Furthermore, comparison of relative divergence times among continental populations (**Table S2** and **Figure S1**) does not indicate a strong correlation between divergences of human and *M.tb* sub-populations: R^2^ = 0.47 for the correlation between human genetic distances and times to most recent common ancestor (TMRCA) of associated *M.tb* lineages. Similar regressions of *H. pylori* and human divergence times produced R^2^ values > 0.95 [[1](#_ENREF_1)].

Additionally, formal tests of co-divergence implemented in TreeMap [[2](#_ENREF_2)] of simplified human [[3](#_ENREF_3)] and *M.tb* phylogenies indicate that there is no significant global signature of co-divergence (**Figure S2**). We performed two sets of analyses: one in which lineages of *M.tb* were associated with human sub-populations based on geographic co-localization, and one in which the associations were based on the scheme described in [[4](#_ENREF_4)]. In congruence analyses that identified all potentially optimal reconciliations of human and *M.tb* phylogenies based on geographic associations, we observed a maximum of eight co-divergence events (CE) and a minimum of 18 non-codivergence events (NCE). These did not differ significantly from CEs and NCEs observed with randomly generated pathogen phylogenies (*p* > 0.88 for ≥8 CE and *p* > 0.19 for ≤18 NCE in 100 randomizations). For the analysis based on Hershberg et al’s hypothesized associations, we observed a maximum of 6 CEs and a minimum of 6 NCEs (p > 0.51 for ≥ 6 CE and *p* > 0.1 for ≤ 6 NCE in 100 randomizations).

**References**

1. Moodley Y, Linz B, Bond RP, Nieuwoudt M, Soodyall H, et al. (2012) Age of the association between Helicobacter pylori and man. PLoS Pathog 8: e1002693.

2. Jackson AP, Charleston MA (2004) A cophylogenetic perspective of RNA-virus evolution. Mol Biol Evol 21: 45-57.

3. Cavalli-Sforza LL, Feldman MW (2003) The application of molecular genetic approaches to the study of human evolution. Nat Genet 33 Suppl: 266-275.

4. Hershberg R, Lipatov M, Small PM, Sheffer H, Niemann S, et al. (2008) High functional diversity in Mycobacterium tuberculosis driven by genetic drift and human demography. PLoS Biol 6: e311.
