# Supplementary material for: The Role of Selection in Shaping Diversity of Natural M. tuberculosis Populations
Source: PLoS Pathog. 2013 Aug 15;9(8):e1003543. doi: 10.1371/journal.ppat.1003543 (PMC3744410; doi:10.1371/journal.ppat.1003543)
Supplement: Text S2 — List of repetitive elements (e.g. transposable elements and mycobacteriophage) and difficult-to-resolve genes (e.g. PE/PPE genes) that were removed from individual bacterial genome sequences prior to their alignment: descriptions and coordinates in H37Rv. (PDF) [file ppat.1003543.s009.pdf]

```
     CDS             129990..131480
                     /gene="PE_PGRS1"
                     /locus_tag="Rv0109"
                     /experiment="EXISTENCE: identified in proteomics study"
                     /note="Rv0109, (MTV031.03c), len: 496 aa. PE_PGRS1, Member of the M. tuberculosis PE
                     family, PGRS subfamily of gly-rich proteins (see Brennan and Delogu, 2002), highly
                     similar to many e.g. Q50615|Y0DP_MYCTU hypothetical glycine-rich 40.8 kDa protein
                     from Mycobacterium tuberculosis (498 aa), FASTA scores: opt: 1772, E(): 0,(57.3%
                     identity in 513 aa overlap); etc."
                     /codon_start=1
                     /transl_table=11
                     /product="PE-PGRS family protein PE_PGRS1"
                     /protein_id="YP_177692.1"
                     /db_xref="GI:57116691 GeneID:886912"
                     /translation="MSLLITSPATVAAAATHLAGIGSALSTANAAAAAPTTALSVAGADEVSVLIAALFEAYAQEYQALSAQALA
                     FHDQFVQALNMGAVCYAAAETANATPLQALQTVQQNVLTVVNAPTQALLGRPIIGNGANGLPNTGQDGGPGGLLFGNGGNGGSGG
                     VDQAGGNGGAAGLIGNGGSGGVGGPGIAGSAGGAGGAGGLLFGNGGPGGAGGIGTTGDGGPGGAGGNAIGLFGSGGTGGMGGVGG
                     MGGVGNGGNAGNGGTAGLFGHGGAGGAGGIGSADGGLGGGGGNGRFMGNGGVGGAGGYGASGDGGNAGNGGLGGVFGDGGAGGTG
                     GLGDVNGGLAGIGGNAGFVRNGGAGGNGQLGSGAVSSAGGMGGNGGLVFGNGGPGGLGGPGTSAGNGGMGGNAVGLFGQGGAGGA
                     GGSGFGAGIPGGRGGDGGSGGLIGDGGTGGGAGAGDAAASAGGNGGNARLIGNGGDGGPGMFGGPGGAGGSGGTIFGFAGTPGPS"
     CDS             148141..149604
                     /gene="PE_PGRS2"
                     /locus_tag="Rv0124"
                     /experiment="EXISTENCE: identified in proteomics study"
                     /note="Rv0124, (MTCI418B.06), len: 487 aa. PE_PGRS2, Member of the Mycobacterium
                     tuberculosis PE family, PGRS subfamily of gly-rich proteins (see Brennan and Delogu,
                     2002), highly similar to many e.g. Y0DP_MYCTU|Q50615 from Mycobacterium tuberculosis
                     (498 aa), FASTA scores: opt: 1730, E(): 0,(60.7% identity in 504 aa overlap)."
                     /codon_start=1
                     /transl_table=11
                     /product="PE-PGRS family protein PE_PGRS2"
                     /protein_id="YP_177693.1"
                     /db_xref="GI:57116692 GeneID:886883"
                     /translation="MSFVSVAPEIVVAAATDLAGIGSAISAANAAAAAPTTAVLAAGADEVSAAIAALFSGHAQAYQALSAQAAA
                     FHQQFVQTLAGGAGAYAAAEAQVEQQLLAAINAPTQALLGRPLIGNGADGAPGTGQAGGAGGILYGNGGNGGSGAAGQAGGAGGP
                     AGLIGHGGSGGAGGSGAAGGAGGHGGWLWGNGGVGGSGGAGVGAGVAGGHGGAGGAAGLWGAGGGGGNGGNGADANIVSGGDGGL
                     GGAGGGGGWLYGDGGAGGHGGQGAIGLGGGAGGDGGQGGAGRGLWGTGGAGGHGGQGGGTGGPPLPGQAGMGAAGGAGGLIGNGG
                     AGGDGGVGASGGVAGVGGAGGNAMLIGHGGAGGAGGDSSFANGAAGGAGGAGGHLFGNGGSGGHGGAVTAGNTGIGGAGGVGGDA
                     RLIGHGGAGGAGGDRAGALVGRDGGPGGNGGAGGQLYGNGGDGAPGTGGTLQAAVSGLVTALFGAPGQPGDTGQPG"
     CDS             complement(328607..331480)
                     /gene="PE_PGRS3"
                     /locus_tag="Rv0278c"
                     /experiment="EXISTENCE: identified in proteomics study"
                     /inference="protein motif:PROSITE:PS00583"
                     /note="Rv0278c, (MTV035.06c), len: 957 aa. PE_PGRS3, Member of the Mycobacterium
                     tuberculosis PE family (see citation below), PGRS subfamily of gly-rich proteins,
                     similar to many e.g. Z95890|MTCY28_25|Rv1759c from Mycobacterium tuberculosis (914
                     aa), FASTA scores: opt: 3849, E(): 0,(67.8% identity in 903 aa overlap). Contains
                     PS00583 pfkB family of carbohydrate kinases signature 1."
                     /codon_start=1
                     /transl_table=11
                     /product="PE-PGRS family protein PE_PGRS3"
                     /protein_id="YP_177707.1"
                     /db_xref="GI:57116712 GeneID:886623"
                     /translation="MSFVIAAPEVIAAAATDLASLGSSISAANAAAAANTTALMAAGADEVSTAIAALFGAHGQAYQALSAQAQA
                     FHAQFVQALTSGGGAYAAAEAAAVSPLLDPINEFFLANTGRPLIGNGANGAPGTGANGGDGGWLIGNGGAGGSGAAGVNGGAGGN
                     GGAGGNGGAGGLIGNGGAGGAGGVASSGIGGSGGAGGNAMLFGAGGAGGAGGGVVALTGGAGGAGGAGGNAGLLFGAAGVGGAGG
                     FTNGSALGGAGGAGGAGGLFATGGVGGSGGAGSSGGAGGAGGAGGLFGAGGTGGHGGFADSSFGGVGGAGGAGGLFGAGGEGGSG
                     GHSLVAGGDGGAGGNAGMLALGAAGGAGGIGGDGGTLTAGGIGGAGGAGGNAGLLFGSGGSGGAGGFGFADGGQGGPGGNAGTVF
                     GSGGAGGNGGVGQGFAGGIGGAGGTPGLIGNGGNGGNGGASAVTGGNGGIGGTGVLIGNGGNGGSGGIGAGKAGVGGVSGLLLGL
                     DGFNAPASTSPLHTLQQNVLNVVNEPFQTLTGRPLIGNGANGTPGTGADGGAGGWLFGNGANGTPGTGAAGGAGGWLFGNGGNGG
                     HGATNTAATATGGAGGAGGILFGTGGNGGTGGIATGAGGIGGAGGAGGVSLLIGSGGTGGNGGNSIGVAGIGGAGGRGGDAGLLF
                     GAAGTGGHGAAGGVPAGVGGAGGNGGLFANGGAGGAGGFNAAGGNGGNGGLFGTGGTGGAGTNFGAGGNGGNGGLFGAGGTGGAA
                     GSGGSGITTGGGGHGGNAGLLSLGASGGAGGSGGASSLAGGAGGTGGNGALLFGFRGAGGAGGHGGAALTSIQQGGAGGAGGNGG
                     LLFGSAGAGGAGGSGANALGAGTGGTGGDGGHAGVFGNGGDGGCRRVWRRYRRQRWCRRQRRADRQRRQRRQRRQSRGHARCRRH
                     RRAAARRERTQRLAIAGRPATTRGVEGISCSPQMMP"
     CDS             complement(331730..334243)
                     /gene="PE_PGRS4"
                     /locus_tag="Rv0279c"
```

```
                     /experiment="EXISTENCE: identified in proteomics study"
                     /note="Rv0279c, (MTV035.07c), len: 837 aa. PE_PGRS4, Member of the Mycobacterium
                     tuberculosis PE family, PGRS subfamily of gly-rich proteins (see Brennan and Delogu,
                     2002),similar to many e.g. Z95890|MTCY28_25|Rv0278c from Mycobacterium tuberculosis
                     (914 aa), FASTA scores: opt: 2677, E(): 0, (64.5% identity in 926 aa overlap)."
                     /codon_start=1
                     /transl_table=11
                     /product="PE-PGRS family protein PE_PGRS4"
                     /protein_id="YP_177708.1"
                     /db_xref="GI:57116713 GeneID:886621"
                     /translation="MSFVIAAPEVIAAAATDLASLESSIAAANAAAAANTTALLAAGADEVSTAVAALFGAHGQAYQALSAQAQA
                     FHAQFVQALTSGGGAYAAAEAAATSPLLAPINEFFLANTGRPLIGNGTNGAPGTGANGGDGGWLIGNGGAGGSGAAGVNGGAGGN
                     GGAGGLIGNGGAGGAGGRASTGTGGAGGAGGAAGMLFGAAGVGGPGGFAAAFGATGGAGGAGGNGGLFADGGVGGAGGATDAGTG
                     GAGGSGGNGGLFGAGGTGGPGGFGIFGGGAGGDGGSGGLFGAGGTGGSGGTSIINVGGNGGAGGDAGMLSLGAAGGAGGSGGSNP
                     DGGGGAGGIGGDGGTLFGSGGAGGVCGLGFDAGGAGGAGGKAGLLIGAGGAGGAGGGSFAGAGGTGGAGGAPGLVGNAGNGGNGG
                     ASANGAGAAGGAGGSGVLIGNGGNGGSGGTGAPAGTAGAGGLGGQLLGRDGFNAPASTPLHTLQQQILNAINEPTQALTGRPLIG
                     NGANGTPGTGADGGAGGWLFGNGGNGGHGATGADGGDGGSGGAGGILSGIGGTGGSGGIGTTGQGGTGGTGGAALLIGSGGTGGS
                     GGFGLDTGGAGGRGGDAGLFLGAAGTGGQAALSQNFIGAGGTAGAGGTGGLFANGGAGGAGGFGANGGTGGNGLLFGAGGTGGAG
                     TLGADGGAGGHGGLFGAGGTGGAGGSSGGTFGGNGGSGGNAGLLALGASGGAGGSGGSALNVGGTGGVGGNGGSGGSLFGFGGAG
                     GTGGSSGIGSSGGTGGDGGTAGVFGNGGDGGAGGFGADTGGNSSSVPNAVLIGNGGNGGNGGKAGGTPGAGGTSGLIIGENGLNG
                     L"
     CDS             353351..355126
                     /gene="PE_PGRS5"
                     /locus_tag="Rv0297"
                     /experiment="EXISTENCE: identified in proteomics study"
                     /note="Rv0297, (MTCY63.02), len: 591 aa. PE_PGRS5, Member of the Mycobacterium
                     tuberculosis PE family, PGRS subfamily of gly-rich proteins (see citation below),
                     highly similar to others e.g. Y03A_MYCTU|Q10637 from Mycobacterium tuberculosis (603
                     aa), FASTA scores: opt: 1884, E(): 0,(53.7% identity in 635 aa overlap). This region
                     is a possible MT-complex-specific genomic island (See Becq et al., 2007)."
                     /codon_start=1
                     /transl_table=11
                     /product="PE-PGRS family protein PE_PGRS5"
                     /protein_id="YP_177713.1"
                     /db_xref="GI:57116718 GeneID:885981"
                     /translation="MSFVIAQPEMIAAAAGELASIRSAINAANAAAAAQTTGVMSAAADEVSTAVAALFSSHAQAYQAASAQAAA
                     FHAQVVRTLTVDAGAYASAEAANAGPNMLAAVNAPAQALLGRPLIGNGANGAPGTGQAGGDGGLLFGNGGNGGSGAPGQAGGAGG
                     AAGFFGNGGNGGDGGAGANGGAGGTAGWFFGFGGNGGAGGIGVAGINGGLGGAGGDGGNAGFFGNGGNGGMGGAGAAGVNAVNPG
                     LATPVTPAANGGNGLNLVGVPGTAGGGADGANGSAIGQAGGAGGDGGNASTSGGIGIAQTGGAGGAGGAGGDGAPGGNGGNGGSV
                     EHTGATGSSASGGNGATGGNGGVGAPGGAGGNGGHVSGGSVNTAGAGGKGGNGGTGGAGGPGGHGGSVLSGPVGDSGNGGAGGDG
                     GAGVSATDIAGTGGRGGNGGHGGLWIGNGGDGGAGGVGGVGGAGAAGAIGGHGGDGGSVNTPIGGSEAGDGGKGGLGGDGGGRGI
                     FGQFGAGGAGGAGGVGGAGGAGGTGGGGGNGGAIFNAGTPGAAGTGGDGGVGGTGAAGGKGGAGGSGGVNGATGADGAKGLDGAT
                     GGKGNNGNPG"
```

```
     CDS             581291..583075
                     /gene="PE_PGRS6"
                     /locus_tag="Rv0532"
                     /note="Rv0532, (MTCY25D10.11), len: 594 aa. PE_PGRS6,Member of the Mycobacterium
                     tuberculosis PE family, PGRS subfamily of gly-rich proteins (see citation
                     below),similar to others e.g. Y0DP_MYCTU|Q50615 from Mycobacterium tuberculosis (498
                     aa), FASTA scores: opt: 1703, E(): 0,(58.2% identity in 536 aa overlap)."
                     /codon_start=1
                     /transl_table=11
                     /product="PE-PGRS family protein PE_PGRS6"
                     /protein_id="YP_177736.1"
                     /db_xref="GI:57116752 GeneID:887391"
                     /translation="MSNLLVTPELVAAAAADLAGIGSAIGAANAAAGAPTMALLAAGADEVSAAVAAVFSSYAQQYQALSAAAAA
                     FHDQFVRALAAGAGAYAGAEAANVEQQLLNAINAPTLALLGRPLIGNGADGAAGTGQAGGAGGLLYGNGGNGGSGAAGQAGGAGG
                     AAGLIGHGGTGGAVTGVSTTGGPGGHGGDAGLYGFGGAGGAGGFGQSGAAGGAGGAGGWLYGDGGDGGAGDNGGNESGTGVSAVG
                     GVGGAGGAGGLLFGNGGDGGVGGDGGDGSSTQDSGGDGGAGGAGGAGGWLLGNGGAGGAGGAASIKVATGGLGGDGGDAGLFGFG
                     GDGGWGGRGVDARFGAAGGAAGAGGAGGWLYGDGGAGGVGGVGGAVFSLSSGDGGAGGAGGGGGWLFGNGGDGGAGGGGGGRFGS
                     GSGAGGDGAVGGAGGAGAWFGNGGAGGVGGGGGRGTTAIGGDGGAGGAGGAGGWLYGDGGAGGAGGGGGRGGTGNDGGDGGDGGR
                     GGDAQLLGNGGDGGAGGAGGPAGLALPPGPARPAGAAVPAVRCSAAPARPARTADPWLAPIFARSTLRHSHHLGGIAQTGAVADQ
                     QGQIAGLGRAGRQ"
     CDS             complement(630494..634414)
                     /gene="PE_PGRS7"
                     /locus_tag="Rv0578c"
                     /experiment="EXISTENCE: identified in proteomics study"
                     /inference="protein motif:PROSITE:PS00583"
                     /note="Rv0578c, (MTV039.16c), len: 1306 aa. PE_PGRS7,Member of the Mycobacterium
                     tuberculosis PE family, PGRS subfamily of gly-rich proteins (see citation below),
                     highly similar to many other PGRS proteins e.g. MTCY493.04|Z95844 from Mycobacterium
                     tuberculosis (1329 aa), FASTA scores: opt: 3994, E(): 0, (54.6% identity in 1375 aa
                     overlap). Contains two PS00583 pfkB family of carbohydrate kinases signatures
                     possibly fortuitously."
                     /codon_start=1
                     /transl_table=11
                     /product="PE-PGRS family protein PE_PGRS7"
                     /protein_id="YP_177739.1"
                     /db_xref="GI:57116755 GeneID:887725"
                     /translation="MSFVIATPEMLTTAATDLAKIGSTITAANTAAAAVAKVLPASADEVSVAVAALFGTHAQEYQTVSAQVATF
                     HDRFVQTLSAAASSYVAAEAVNVEQSLLAAVNAPTQALFGRPLIGNGADGSPGTGQAGGPGGILYGNGGNGGSGAPGQRGGAGGA
                     AGLIGNGGNGGAGGVGTTGGAGGHGGAGGWLYGNGGAGGFGGAGAVGGNGGAGGTAGLFGVGGAGGAGGNGIAGVTGTSASTPGG
                     SGTAGGAGGIGGNGGAGGAGGVLMGNGGNGGAGGEGGPGGAGGAGASGAHATNLGADGQAGGNGGNGGAGGTGGVGGPGGGHGLL
                     GLGGSHGAGGAGGSGGDGGAPGDGGNGATGTWGHNLGAGGTGGNGGNPGAGGAGGAGGASVGGSAHGANGAPGTTSTSGGNGGDG
                     GKGADAISSGQTGANGGRGGDGGQVGNGGAGGAGGRGGAGGLGFGSEAPGRPGGAGGTGGAGGNGGTQAGDGGTGGAGGAGGDGG
                     SGGAGSIGFNASAPGAAGSPGGNGGNGGPGGAGGEGGAGGLALAASGQNGSQGAGGDGGAGGNGGTPGNGGHGAAGALGVNGGVG
                     GAGGHGGDPGVGGAGGQGGSGSTPGANGAPGNTPTSGGNGGNGGRGADATGFGQTGASGGRGGDGGLVGNGGAGGAGGNGSKGLP
                     GLGRLGNPGLDGGTGGNGGAGGSGGAWAGNGGTGGAGGTGGVGGTGGSGSDGVNGSSAGADGHPGGTGGVGGTGGKGGDGGDGGA
                     APNGVAGSQGPGGAGGDGGTGGVGGNGGRGIDGADGATAGARGQDGGAGGAGGKGGRGGTGGPGGAGPAGTTGSQGAGGNGGSGG
                     TGGDPGDGGNGANGSVFTNNGIGGNGGNGGNAGPSGAGGSGGAGSTFGATGSSSSIHVNGGNGGNGGNGDHALSGNGAAGGNGGN
                     GGNGSLRGSGGAGGHGGNGGNASRGMGGDGGTGGAGGNAGQIGNGGAGGNGGDGGTGSDGNPGAITGSGGRGGDGGVGGQGGSVA
                     GDGADGGRGGAGGTGGTGLRGTTGATGATGTFDAGADGHGGNGGTGGVGGTGGAGGGGGNGGAGGKALSPTGNNGSQGAGGDGGA
                     GGAGGTGGTGGDGGRGAHGTLFSSLAGTGGTGGNGGTGGTGGTGGAGGAGGTGSTLGATGATGAAGRAGNGGVGGSGGLGSAFGP
                     GGTGGMGGAGGTSTVSAGGDGGRGGFGGDGLDASSGGNGGDGGHGGDGFRTAGAGGRGGDGGKGADPGGLFPIPGAGGKGGTGGT
                     GGTAHLGPLAIIGQSGQPGQFGSPGADGRGGAGGAGGGGGAGGSF"
     CDS             791479..792006
                     /gene="PE_PGRS8"
                     /locus_tag="Rv0742"
                     /note="Rv0742, (MTV041.16), len: 175 aa. PE_PGRS8, Member of the Mycobacterium
                     tuberculosis PE family, PGRS subfamily of gly-rich proteins (see citation below),
                     similar to many Mycobacterium tuberculosis PGRS-type proteins e.g. Z78020|MTCY1A11_25
                     (498 aa), FASTA scores: opt: 766, E(): 6.1e-25, (73.6% identity in 178 aa overlap).
                     Similarity suggests ORF starts with ATA start codon. This region is a possible
                     MT-complex-specific genomic island (See Becq et al., 2007)."
                     /codon_start=1
                     /transl_table=11
                     /product="PE-PGRS family protein PE_PGRS8"
                     /protein_id="YP_177749.1"
                     /db_xref="GI:57116772 GeneID:888645"
                     /translation="MSFVIAAPEAIAAAATDLASIGSTIGAANAAAAANTTAVLAAGADQVSVAIAAAFGAHGQAYQALSAQAAT
                     FHIQFVQALTAGAGSYAAAEAASAASITSPLLDAINAPFLAALGRPLIGNGADGAPGTGAAGGAGGLLFGNGGAGGSGAPGGAGG
                     LLFGNGGAGGPGASGGALG"
```

```
     CDS             794199..796550
                     /gene="PE_PGRS9"
                     /locus_tag="Rv0746"
                     /experiment="EXISTENCE: identified in proteomics study"
                     /note="Rv0746, (MTV041.20), len: 783 aa. PE_PGRS9, Member of the Mycobacterium
                     tuberculosis PE family, PGRS subfamily of gly-rich proteins (see citation below),
                     highly similar to part of MTCY28.25c|Rv1759c|Z95890 antigen wag22 from M.
                     tuberculosis (914 aa), FASTA scores: opt: 2429, E(): 0,(56.9% identity in 873 aa
                     overlap). Also similar to other PE-PGRS family proteins e.g. AL0212|MTV008_46 FASTA
                     score: (48.8% identity in 887 aa overlap); etc. This region is a possible
                     MT-complex-specific genomic island (See Becq et al., 2007)."
                     /codon_start=1
                     /transl_table=11
                     /product="PE-PGRS family protein PE_PGRS9"
                     /protein_id="YP_177750.1"
                     /db_xref="GI:57116773 GeneID:888664"
                     /translation="MSFVLAMPEVLGSAATDLAALGSVLGAADAAAAATTTGIVAAAQDEVSAAIAALFSAHGRAYQVASAQAAA
                     VHAQFVEALSAGAGAYASAEAAGAAVLANPAQSVQQDLLAAVNAQSVALTGRPLIGNGANGAPGTGANGAPGGWLLGNGGAGGSA
                     AAGSGLPGGAGGAAGLFGTGGAGGAGGSSTVGDGEAGGAGGSGGWLLGTGGVGGVGGLGAGAGGAGGVGGAGGLLGAGGHGGAGG
                     LGAVTGGVGGTGGAGGLLAGLLAGPGGAGGTGGRGFLNNGGVGGAGGNAGLLFGAGGTGGSGGAGLGGDGGAGGAGGNTGVLFGN
                     AGSGGTGGFGDTDGGAGGAGGDAGWLGSGGVGGAGGFGETGDGGVGGAGGKAGLLIGNGGAGGAGGQGAVTGGTGGAGGDGVLIG
                     NGGNAGIGGTGPTAGDTGAGGISGLLLGADGFNTPASASPLHTLKQQALAAINAPTQTLTGRPLIGNGTPGAVGSGATGAPGGWL
                     LGDGGAGGSGAAGSGAPGGAGGAAGLWGTGGAGGAGGSSAGGGGAGGAGGAGGWLLGDGGAGGIGGASTVLGGTGGGGGVGGLWG
                     AGGAGGAGGTGLVGGDGGAGGAGGTGGLLAGLIGAGGGHGGTGGLSTNGDGGVGGAGGNAGMLAGPGGAGGAGGDGENLDTGGDG
                     GAGGSAGLLFGSGGAGGAGGFGFLGGDGGAGGNAGLLLSSGGAGGFGGFGTAGGVGGAGGNAGWLGFGGAGGVGGSAGLIGTGGN
                     GGNGGTGANAGSPGTGGAGGLLLGQNGLNGLP"
     CDS             796949..799354
                     /gene="PE_PGRS10"
                     /locus_tag="Rv0747"
                     /experiment="EXISTENCE: identified in proteomics study"
                     /inference="protein motif:PROSITE:PS00012"
                     /note="Rv0747, (MTV041.21), len: 801 aa. PE_PGRS10, Member of the Mycobacterium
                     tuberculosis PE family, PGRS subfamily of gly-rich proteins (see citation below),
                     highly similar to part of MTCY28.25c|Rv1759c|Z95890 antigen wag22 from M.
                     tuberculosis (914 aa), FASTA scores: opt: 2772, E(): 0,(60.9% identity in 941 aa
                     overlap). Also similar to other PE-PGRS family proteins e.g. Z95844|MTCY493_2 FASTA
                     score: (50.2% identity in 815 aa overlap). Contains PS00012 Phosphopantetheine
                     attachment site. This region is a possible MT-complex-specific genomic island (See
                     Becq et al., 2007)."
                     /codon_start=1
                     /transl_table=11
                     /product="PE-PGRS family protein PE_PGRS10"
                     /protein_id="YP_177751.1"
                     /db_xref="GI:57116774 GeneID:888662"
                     /translation="MSWVMVSPELVVAAAADLAGIGSAISSANAAAAVNTTGLLTAGADEVSTAIAALFGAQGQAYQAASAQAAA
                     FYAQFVQALSAGGGAYAAAEAAAVSPLLAPINAQFVAATGRPLIGNGANGAPGTGANGGPGGWLIGNGGAGGSGAPGAGAGGNGG
                     AGGLFGSGGAGGASTDVAGGAGGAGGAGGNAGMLFGAAGVGGVGGFSNGGATGGAGGAGGAGGLFGAGRERGSGGSGNLTGGAGG
                     AGGNAGTLATGDGGAGGTGGASRSGGFGGAGGAGGDAGMFFGSGGSGGAGGISKSVGDSAAGGAGGAPGLIGNGGNGGNGGASTG
                     GGDGGPGGAGGTGVLIGNGGNGGSGGTGATLGKAGIGGTGGVLLGLDGFTAPASTSPLHTLQQDVINMVNDPFQTLTGRPLIGNG
                     ANGTPGTGADGGAGGWLFGNGGNGGQGTIGGVNGGAGGAGGAGGILFGTGGTGGSGGPGATGLGGIGGAGGAALLFGSGGAGGSG
                     GAGAVGGNGGAGGNAGALLGAAGAGGAGGAGAVGGNGGAGGNGGLFANGGAGGPGGFGSPAGAGGIGGAGGNGGLFGAGGTGGAG
                     GGSTLAGGAGGAGGNGGLFGAGGTGGAGSHSTAAGVSGGAGGAGGDAGLLSLGASGGAGGSGGSSLTAAGVVGGIGGAGGLLFGS
                     GGAGGSGGFSNSGNGGAGGAGGDAGLLVGSGGAGGAGASATGAATGGDGGAGGKSGAFGLGGDGGAGGATGLSGAFHIGGKGGVG
                     GSAVLIGNGGNGGNGGNSGNAGKSGGAPGPSGAGGAGGLLLGENGLNGLM"
     CDS             804657..806411
                     /gene="PE_PGRS11"
                     /locus_tag="Rv0754"
                     /experiment="EXISTENCE: identified in proteomics study"
                     /note="Rv0754, (MTV041.28), len: 584 aa. PE_PGRS11, Member of the Mycobacterium
                     tuberculosis PE family, PGRS subfamily of gly-rich proteins (see citation below),
                     similar to others e.g. AL0212|MTV008_46 from Mycobacterium tuberculosis (1660 aa),
                     FASTA score: (48.7% identity in 345 aa overlap); Z80225|MTCY441_4 from Mycobacterium
                     tuberculosis (778 aa), FASTA score: (41.6% identity in 442 aa overlap); etc."
                     /codon_start=1
                     /transl_table=11
                     /product="PE-PGRS family protein PE_PGRS11"
                     /protein_id="YP_177752.1"
                     /db_xref="GI:57116776 GeneID:888695"
                     /translation="MSFVIVARDALAAAAADLAQIGSAVNAGNLAAANPTTAVAAAAADEVSAALAALFGAHAREYQAAAAQAAA
```

YHEQFVHRLSAAATSYAVTEVTIATSLRGALGSAPASVSDGFQAFVYGPIHATGQQWINSPVGEALAPIVNAPTNVLLGRDLIGN
GVTGTAAAPNGGPGGLLFGDGGAGYTGGNGGSAGLIGNGGTGGAGFAGGVGGMGGTGGWLMGNGGMGGAGGVGGNGGAGGQALLF
GNGGLGGAGGAGGVDGAIGRGGWFIGTGGMATIGGGGNGQSIVIDFVRHGQTPGNAAMLIDTAVPGPGLTALGQQQAQAIANALA
AKGPYAGIFDSQLIRTQQTAAPLANLLGMAPQVLPGLNEIHAGIFEDLPQISPAGLLYLVGPIAWTLGFPIVPMLAPGSTDVNGI
VFNRAFTGAVQTIYDASLANPVVAADGNITSVAYSSAFTIGVGTMMNVDNPHPLLLLTHPVPNTGAVVVQGNPEGGWTLVSWDGI
PVGPASLPTALFVDVRELITAPQYAAYDIWESLFTGDPAAVINAVRDGADEVGAAVVQFPHAVADDVIDATGHPYLSGLPIGLPS
LIP"

CDS 881511..881924
/gene="PE_PGRS12"
/locus_tag="Rv0832"
/experiment="EXISTENCE: identified in proteomics study"
/note="Rv0832, (MTV043.24), len: 137 aa. PE_PGRS12, Member of the Mycobacterium tuberculosis PE family, possibly PGRS subfamily of gly-rich proteins (see citation below), highly similar to many others e.g. MTCY1A11.25c|Z78020 (498 aa),FASTA scores: opt: 529, E(): 5.2e-22, (61.8% identity in 136 aa overlap); etc. Appears to have incurred frameshift as next ORF should be continuation; sequence has been checked but no error found."
/codon_start=1
/transl_table=11
/product="PE-PGRS family protein PE_PGRS12"
/protein_id="YP_177759.1"
/db_xref="GI:57116787 GeneID:885236"
/translation="MSYVSVLPATLATAATEVARIGSALSLASAVAAAQTSAVQAAAADEVSAAIAALFSAHGRDFQALSARAAA
FHHEFVQALAAGAGSYAVAEIAAASPLQSLIDVFNAPIQAATGRPLIGNGANGQPGTGAPGGPAGG"

CDS 881921..884170
/gene="PE_PGRS13"
/locus_tag="Rv0833"
/note="Rv0833, (MTV043.25), len: 749 aa. PE_PGRS13, Member of the Mycobacterium tuberculosis PE family, PGRS subfamily of gly-rich proteins (see Brennan and Delogu, 2002), but lacking N-terminal domain (present in preceding ORF),possibly due to frameshift. Similar in part to many others e.g. MTCY28_25|Z95890 (914 aa), FASTA scores: opt: 2726,E(): 0, (60.1% identity in 776 aa overlap); etc."
/codon_start=1
/transl_table=11
/product="PE-PGRS family protein PE_PGRS13"
/protein_id="YP_177760.1"
/db_xref="GI:57116788 GeneID:885391"
/translation="MIGNGGAGGSGAPGAIGGAGGPAGLIGVGGAGGAGGDSAVAGVIGGAGGAGGAALLFGAGGAGGAGGSGGS
GAAGGAGGAGGAGGLFASGGSGGFGGFASTGTGGAGGTGGAGGLFASGGVGGTGGGAGSGGTGGVGGTGGAGGLFASGGAGGAGG
SGGTGGAGGTGGAGGLFGAGGAGGLGGQGNHTGGHGGAGGSAGLLALGDGGAGGAGGAATTGTGGAGGAGGKAGLLFGSGGAGGS
GGAAGTFGDTGNSGGAGGAGGKAGLLFGSGGAGGSGGAGGFANGSTGGAGGAGGGAGLIGNGGNGGSGGTSVATGGAGNGGAGGA
GGGAGLIGNGGNGGSGGMGDAPGGTGVGGIGGLLLGLDGANAPASTNPLHTAQQQALAAVNAPIQAVTGRPLIGNGANGAPGSGA
PGGHGGWLFGGGGTGGSGVSGGAGGDGGAGGILFGAGGAGGAGGAVTGTGATGGSGGAGGGALLFGAGGAGGAGGSSGIGGFAAG
GAGGPGGAGGLFNGGGAGGAGGSGVSGGAGGEGGAGGAGGLFAGGGAGGAGGSGNNVGGAGGAGGVGGLFGAGGAGGSGGGGSVA
GDSGAGGNAGLLAPGLAGGAGGGGGQGFDTGGAGGPGGDAGLLVGSGGVGGAGGFGLTTGGPGAAGGDAGLLFGSGGAGGAGGSG
RTDLGGAGGAGGKAGLIGNGGNGGAGGAGGNGGGDGGPGGAAFGLGNGGNGGNGGTGTSAGSPGAGGAGGSLIGAEGLPGLLP"

CDS complement(884397..887045)
/gene="PE_PGRS14"
/locus_tag="Rv0834c"
/experiment="EXISTENCE: identified in proteomics study"
/note="Rv0834c, (MTV043.26c), len: 882 aa. PE_PGRS14,Member of the Mycobacterium tuberculosis PE family, PGRS subfamily of gly-rich proteins (see Brennan & Delogu 2002),highly similar to many others e.g. MTCY493_4|Z95844 (1329 aa), FASTA scores: opt: 2577, E(): 0, (52.0% identity in 950 aa overlap); etc. Thought to be differentially expressed within host cells (see Triccas et al., 1999)."
/codon_start=1
/transl_table=11
/product="PE-PGRS family protein PE_PGRS14"
/protein_id="YP_177761.1"
/db_xref="GI:57116789 GeneID:885369"
/translation="MSFVIAAPDLVAMATEDLAGIGASLTAANAAAAVPTSGLLAAAGDEVSAAIAALFSSHGQQYQAMSAQAAA
FHARFVQALAGAMGAYAAAEAANASPLQTLEQGLLGAINAPAAALSGRPFIGNGTNGAPGTGEAGGPGGWLLGNGGNGGSGAPGQ
TGGAGGAAGLLGHGGTGGAGGTGASGGKGGTGGWLWGSGGAGGAGGSGGGSGGAGGNALMFGIGGNGGAGGAASGVGNGGVGGAG
GAGGALVAIGGAGGAGGAATTGTGGAGGAGSNALGLFLGLGGSGGQGGDSAMGSGGAGGAGGSGGAASPFGIDIGIGGAGGHGGA
GTNGGAGGAGGAGGSSGTVFALDLSWGGAGGNGGAATTGTGGAGGTGGFAVAPDFIGFGAAYGGAGGLGGAATGAGGTGGTGGVG
AGGFAALGVGVGGAGGAGGAATETGGIGGAGGLGVGLLGGAGGAGGPGGAASAGSGGHGGTGGDALGLIGAGIGGVGGVGGAATD
TGGNGGAGGSGTGLLGGVGGAGGHGGGASVGTGGSGGAGGDGFGFVGAGGNGGNAGTGVGVNGANGGNGGSATGALAAVGGAGAA
GGDATSGTGGFGGAGGSARGLIFALGGAGAAGGDASTGVGGPGGPGGTGTASSPFGIAIAIGGAGAQGGAGTSGATGGAGGDGVF
EGIAVLGLGFGGAAGAGGAATGDGATGGAGGFGGAGAGIANFLGFSVLHGGAGGAGGTATGTGGNGGAGGGGGLSSPVILGIGIG

```
                     GAGGDGGGALGVLGGMGGDGGDGGEAVAVGIAVGGAGGAGGAAPTGNGGAGGNGGDALGLVGVGGNGGNAGTGFGANTGGNGGDT
                     TIVVNGMLAPSTLGYGGNGGNGVNGGAGGTGGKAGVFGAPGQNGLP"
     CDS             complement(924984..926804)
                     /gene="PE_PGRS15"
                     /locus_tag="Rv0872c"
                     /experiment="EXISTENCE: identified in proteomics study"
                     /inference="protein motif:PROSITE:PS00017"
                     /note="Rv0872c, (MTV043.65c), len: 606 aa. PE_PGRS15,Member of the Mycobacterium
                     tuberculosis PE family, PGRS subfamily of gly-rich proteins (see Brennan & Delogu
                     2002),similar to many e.g. MTCY24A1.04c|Z95207 (615 aa), FASTA scores: opt: 2636,
                     E(): 0, (64.6% identity in 619 aa overlap); etc."
                     /codon_start=1
                     /transl_table=11
                     /product="PE-PGRS family protein PE_PGRS15"
                     /protein_id="YP_177763.1"
                     /db_xref="GI:57116793 GeneID:885742"
                     /translation="MSYVLATPEMVAAAANNLAQIGSTLSAANAAALAPTTGVLAAGADEVSAAVASLFSGHAQAYQTLGTQAAA
                     FHERFIQALSTAAGAYGSAEAANASPLQQALNVINAPTQTLLGRPLIGNGTNGAPGTGQAGGPGGLLYGNGGNGGSGGVGQAGGA
                     GGSAGLIGIGGTGGAGGAGAVGGVGGNGGWLYGNGGAGGLGGTGVAGVNGGMGAAGGAGGNAYLFGSGGAGGQGGMGAAGADGVN
                     PTPTGTADAGSTGTDQTLGGNAIGGNGGPGDAGDAMTSGGAGGSGGNAVSTVNGDAVGGEGGKGGEGAYGGAGGAGGSAASIGNA
                     AIGGNGGAGGNAQAPGGVGGAGGEGGDAQVGTNSPSNAEAGNGGSGGNGFDSFASGGTGGAGGTGGAGGRGGLLIGDGGAGGAGG
                     VGGTGGSGAPGGGGGAGGDGGAANTDSAGSSRKAFGGDGGVGGDGASALGTGGEGGIGGQGGNGGAGGLLIGNGGAGGVGGTAGA
                     GGTGGSGGAGGAGGAGGGGTNSGPGAAFGGNGNTGGNGGNGGAPGALGGKGGSGGLIGRAGSDGGVGAGGAGGAGGAGGTGGEGG
                     TGGDGKTTDGNPGMGGSPGSAGQPG"
     CDS             1044329..1047100
                     /gene="PE_PGRS16"
                     /locus_tag="Rv0977"
                     /note="Rv0977, (MTV044.05), len: 923 aa. PE_PGRS16, Member of the Mycobacterium
                     tuberculosis PE family, PGRS subfamily of gly-rich proteins (see citation below),
                     highly similar to other PGRS-type sequences e.g. AL0091|MTV004_1 from Mycobacterium
                     tuberculosis (1125 aa), FASTA score: (45.4% identity in 959 aa overlap);
                     Z80225|MTCY441_4 from Mycobacterium tuberculosis (778 aa), FASTA score: (51.5%
                     identity in 750 aa overlap); etc."
                     /codon_start=1
                     /transl_table=11
                     /product="PE-PGRS family protein PE_PGRS16"
                     /protein_id="YP_177773.1"
                     /db_xref="GI:57116805 GeneID:885264"
                     /translation="MSFVVTAPPVLASAASDLGGIASMISEANAMAAVRTTALAPAAADEVSAAIAALFSSYARDYQTLSVQVTA
                     FHVQFAQTLTNAGQLYAVVDVGNGVLLKTEQQVLGVINAPTQTLVGRPLIGDGTHGAPGTGQNGGAGGILWGNGGNGGSGAPGQP
                     GGRGGDAGLFGHGGHGGVGGPGIAGAAGTAGLPGGNGANGGSGGIGGAGGAGGNGGLLFGNGGAGGQGGSGGLGGSGGTGGAGMA
                     AGPAGGTGGIGGIGGIGGAGGVGGHGSALFGHGGINGDGGTGGMGGQGGAGGNGWAAEGITVGIGEQGGQGGDGGAGGAGGIGGS
                     AGGIGGSQGAGGHGGDGGQGGAGGSGGVGGGGAGAGGDGGAGGIGGTGGNGSIGGAAGNGGNGGRGGAGGMATAGSDGGNGGGGG
                     NGGVGVGSAGGAGGTGGDGGAAGAGGAPGHGYFQQPAPQGLPIGTGGTGGEGGAGGAGGDGGQGDIGFDGGRGGDGGPGGGGGAG
                     GDGSGTFNAQANNGGDGGAGGVGGAGGTGGTGGVGADGGRGGDSGRGGDGGNAGHGGAAQFSGRGAYGGEGGSGGAGGNAGGAGT
                     GGTAGSGGAGGFGGNGADGGNGGNGGNGGFGGINGTFGTNGAGGTGGLGTLLGGHNGNIGLNGATGGIGSTTLTNATVPLQLVNT
                     TEPVVFISLNGGQMVPVLLDTGSTGLVMDSQFLTQNFGPVIGTGTAGYAGGLTYNYNTYSTTVDFGNGLLTLPTSVNVVTSSSPG
                     TLGNFLSRSGAVGVLGIGPNNGFPGTSSIVTAMPGLLNNGVLIDESAGILQFGPNTLTGGITISGAPISTVAVQIDNGPLQQAPV
                     MFDSGGINGTIPSALASLPSGGFVPAGTTISVYTSDGQTLLYSYTTTATNTPFVTSGGVMNTGHVPFAQQPIYVSYSPTAIGTTT
                     FN"
     CDS             complement(1047317..1048312)
                     /gene="PE_PGRS17"
                     /locus_tag="Rv0978c"
                     /experiment="EXISTENCE: identified in proteomics study"
                     /note="Rv0978c, (MTV044.06c), len: 331 aa. PE_PGRS17,Member of the Mycobacterium
                     tuberculosis PE family, PGRS subfamily of gly-rich proteins (see citation below),
                     highly similar to others e.g. Z95387|MTCY1A10_19 from Mycobacterium tuberculosis (461
                     aa), FASTA score: (73.6% identity in 277 aa overlap); etc."
                     /codon_start=1
                     /transl_table=11
                     /product="PE-PGRS family protein PE_PGRS17"
                     /protein_id="YP_177774.1"
                     /db_xref="GI:57116806 GeneID:885077"
                     /translation="MSFVNVAPQLVSTAAADAARIGSAINTANTAAAATTQVLAAAQDEVSTAIAALFGSHGQHYQAISAQVAAY
                     QQRFVLALSQAGSTYAVAEAASATPLQNVLDAINAPVQSLTGRPLIGDGANGIDGTGQAGGNGGWLWGNGGNGGSGAPGQAGGAG
                     GAAGLIGNGGAGGTGGAVSLARAGTAGGAGRGPVGGIGGAGGVGGAGGAAGAVTTITHASFNDPHGVAVNPGGNVYVTNFGSGTV
                     SVINPATNTVTGSPITIGNGPSGVAVSPVTGLVFVTNFDSNTVSVIDPTTNTVTGSPITVGTAPTGVAVNPVTGEVYVTNFAGDT
                     VSVIS"
     CDS             complement(1049034..1050407)
```

```
                     /gene="PE_PGRS18"
                     /locus_tag="Rv0980c"
                     /note="Rv0980c, (MTV044.08c), len: 457 aa. PE_PGRS18,Member of the Mycobacterium
                     tuberculosis PE family, PGRS subfamily of gly-rich proteins (see Brennan & Delogu
                     2002),highly similar to others e.g. Z95387|MTCY1A10_19 from Mycobacterium
                     tuberculosis (461 aa), FASTA score: (66.7% identity in 405 aa overlap);
                     Z95844|MTCY493_2 from Mycobacterium tuberculosis (741 aa), FASTA score: (53.0%
                     identity in 394 aa overlap); etc. Predicted to be an outer membrane protein (See Song
                     et al., 2008)."
                     /codon_start=1
                     /transl_table=11
                     /product="PE-PGRS family protein PE_PGRS18"
                     /protein_id="YP_177775.1"
                     /db_xref="GI:57116809 GeneID:885327"
                     /translation="MSFVNVAPQLVSTAAADAARIGSAINTANTAAAATTQVLAAAHDEVSTAIAALFGSHGQHYQAISAQVAAY
                     QERFVLALSQASSTYAVAEAASATPLQNVLDAINAPVQSLTGRPLIGDGANGIDGTGQAGGNGGWLWGNGGNGGSGAPGQAGGAG
                     GAAGLIGNGGAGGAGGQGLPFEAGANGGAGGAGGWLFGNGGAGGVGGAGGAGTTFGVAGGDGGTGGVGGHGGLIGVGGHGGDGGT
                     GGTGGAVSLARAGTAGGAGGGPAGGIGGAGGVGGAGGAAGAVTTITHASFNDPHGVAVNPGGNIYVTNQGSNTVSVIDPVTNTVT
                     GSITDGNGPSGVAVSPVTGLVFVTNFDSNTVSVIDPNTNTVTGSIPVGTGAYGVAVNPGGNIYVTNQFSNTVSVIDPATNTVTGS
                     PIPVGLDPTGVAVNPVTGVVYVTNSLDDTVSVITGEPARSVCSAAI"
     CDS             complement(1141201..1143204)
                     /gene="PE_PGRS19"
                     /locus_tag="Rv1067c"
                     /experiment="EXISTENCE: identified in proteomics study"
                     /inference="protein motif:PROSITE:PS00583"
                     /note="Rv1067c, (MTV017.20c), len: 667 aa. PE_PGRS19,Member of the Mycobacterium
                     tuberculosis PE family, PGRS subfamily of gly-rich proteins (see Brennan & Delogu
                     2002). Similar to Rv3388|MTV004.46 M. tuberculosis (731 aa), FASTA scores: opt: 2227,
                     E(): 0, (55.6% identity in 710 aa overlap). Contains PS00583 pfkB family of
                     carbohydrate kinases signature 1, probably fortuitous. Predicted to be an outer
                     membrane protein (See Song et al., 2008)."
                     /codon_start=1
                     /transl_table=11
                     /product="PE-PGRS family protein PE_PGRS19"
                     /protein_id="YP_177780.1"
                     /db_xref="GI:57116818 GeneID:887122"
                     /translation="MSFVLVSPSQLMAAAADVAGIGSAISAANAAALAPTSVLAAAGADEVSAAVAALFSAHAGQYQQLGARAAL
                     FHEQFVQALTGAASAYASAEATNVEQQVLGLINAPTQALLGRPLIGNGADGTAANPNGGAGGLLYGNGGNGFSQTTAGLTGGTGG
                     SAGLIGNGGNGGAGGAGANGGAGGNGGWLYGSGGNGGAGGAGPAGAIGAPGVAGGAGGAGGTAGLFGNGGVGGVGGDGGQGGNGA
                     GAGASGTKGGDAGAGGAGGAGGWIHGHGGAGGDGGAGGAGGQASPGAPGPPSQPGGAGGAGGAGGRGGDGGSAGWLSGNGGDAGN
                     GGGGGTAGGAGNGGQFGGDGGTGGTGGTAGAGGNGGRGAVLFGHGGNAGHGGAGGNGAAAGAGGEHVVATAGKGGTGGVGGDGGG
                     GGAGGGGGLLYGNGGAGGAGNSGGDGGTGLNAALGGNGGGGGVGGNAGAGGTGGSAGWLSGNGGAGGSGGSAGAGGAGGKGGDTP
                     NGLAINPGIGGNGGDTGNAGNGGNGGSAARLFGGGGAGGAGGTGSTAGSGGSGGTNPPTGLQAAGGNGGSGHAGGHGGNGGGAGL
                     LGGGGTGGNGGGGGQGGLGAAAGGVDGNGGNGGNGGKGGDAQLVGDGGNGGNGGKGGAGLIAGLDGAGGAGGTRGLIFGNAGTPG
                     Q"
     CDS             complement(1143537..1144928)
                     /gene="PE_PGRS20"
                     /locus_tag="Rv1068c"
                     /note="Rv1068c, (MTV017.21c), len: 463 aa. PE_PGRS20,Member of the Mycobacterium
                     tuberculosis PE family, PGRS subfamily of gly-rich proteins (see Brennan & Delogu
                     2002). Similar to AL021897|MTV017_19 Mycobacterium tuberculosis H37Rv (667 aa), FASTA
                     scores: opt: 1875, E(): 0, (55.0% identity in 667 aa overlap)."
                     /codon_start=1
                     /transl_table=11
                     /product="PE-PGRS family protein PE_PGRS20"
                     /protein_id="YP_177781.1"
                     /db_xref="GI:57116819 GeneID:887123"
                     /translation="MSYMIAVPDMLSSAAGDLASIGSSINASTRAAAAATTRLLPAAADEVSAHIAALFSGHGEGYQAIARQMAA
                     FHDQFTLALTSSAGAYASAEATNVEQQVLGLINAPTQALLGRPLIGNGADGTAANPNGGAGGLLYGNGGNGFSQTTAGLTGGTGG
                     SAGLIGNGGNGGAGGAGANGGAGGNGGWLYGSGGNGGAGGAGPAGAIGAPGVAGGAGGAGGTAGLFGNGGAGGAGGAGGAGGRGG
                     DGGSAGWLSGNGGDAGTGGGGGNAGNGGNGGSAGWLSGNGGTGGGGGTAGAGGQGGNGNSGIDPGNGGQGADTGNAGNGGHGGSA
                     AKLFGDGGAGGAGGMGSTGGTGGGGGFGGGTGGNGGNGHAGGAGGSGGTAGLLGSGGSGGTGGDGGNGGLGAGSGAKGNGGNGGD
                     GGKGGDAQLIGNGGNGGNGGKGGTGLMPGINGTGGAGGSRGQISGNPGTPGQ"
     CDS             1164340..1166643
                     /gene="PE_PGRS21"
                     /locus_tag="Rv1087"
                     /experiment="EXISTENCE: identified in proteomics study"
                     /inference="protein motif:PROSITE:PS00583"
                     /note="Rv1087, (MTV017.40), len: 767 aa. PE_PGRS21, Member of the Mycobacterium
```

```
                     tuberculosis PE family, PGRS subfamily of gly-rich proteins (see citation below).
                     Similar to Rv1090|AL021897|MTV017_43 Mycobacterium tuberculosis H37Rv (853 aa), FASTA
                     scores: opt: 2819, E(): 0, (59.8% identity in 860 aa overlap). Contains PS00583 pfkB
                     family of carbohydrate kinases signature 1 near C -terminus. Predicted to be an outer
                     membrane protein (See Song et al.,2008)."
                     /codon_start=1
                     /transl_table=11
                     /product="PE-PGRS family protein PE_PGRS21"
                     /protein_id="YP_177783.1"
                     /db_xref="GI:57116821 GeneID:887094"
                     /translation="MSFVVVAPEVLAAAASDLAGIGSTLAQANAAALAPTTAVLAAGADEVSAAIASLFGAHGQAYQAVSAQMSA
                     FHAQFMQALTGAGGAYAAAEAVNVSAAQSVEQDLLAAINARFERIFGRPLIGDGANGGPGQDGGPGGLLYGNGGNGGTSTTVGMA
                     GGNGGAAGLIGNGGFGGGGGPGAAGGNGGAGGWLFGNGGAGGAGGLGVAPGVPGGAGGAGGAGGVGGPAGLWGHGGAGGAGGAGV
                     AGAGGFEGTIGAGGAGGVGGAGGVGGAGGAGGWLYGDAGAGGDGGVGGAGGTGGLGNRGGAGGAGGAGGVGGAGGAAGLWGGGGA
                     GGVGGTGGGAGLGAQSVTFSSSLSGLSGGDGGAGGAGGAGGAGGTGGWLYGGGGAAGSGGDGGTGGQGGAGGAGVFSLFGSGGGP
                     GGNGGVGGVGGVGGAGGRAGLFGVGGLGGAGGDAGDSGEGGFGGPGLAGGLFGNPGNGGVGGIGGDAAAGGAGGAGGNGGAGGNG
                     GWLFGNGGAGGSGGDGGAAGRGGAGNLGSAGGINAPAGNPGSGSVGIGGAGGAGGTAGLFGDGGAGGAGGAGAAGGFGGISAATP
                     SAGSEGAMGGAGGVGGNARLLGTGGAGGVGGGGGAGGDGGRGGVATPGGQGGDAGDGGAGGAGGNGGGASGAGGWLLGTGGAGGA
                     GGNGGNGGKAGFSPGPTNFGLNGAGGGGGVGGNGATGPWLFGDGGPTPGSTGAGAAGGHGGDAQLIGNGGHGGAGGTGVPNGSGG
                     AGGLSGLLFGEPGANG"
     CDS             1169249..1171810
                     /gene="PE_PGRS22"
                     /locus_tag="Rv1091"
                     /experiment="EXISTENCE: identified in proteomics study"
                     /note="Rv1091, (MTV017.44), len: 853 aa. PE_PGRS22, Member of the Mycobacterium
                     tuberculosis PE family, PGRS subfamily of gly-rich proteins (see citation below).
                     Similar to Rv1087|AL021897|MTV017_39 Mycobacterium tuberculosis H37Rv (767 aa), FASTA
                     scores: opt: 2819, E(): 0, (60.0% identity in 860 aa overlap). Predicted to be an
                     outer membrane protein (See Song et al., 2008). This region is a possible
                     MT-complex-specific genomic island (See Becq et al.,2007)."
                     /codon_start=1
                     /transl_table=11
                     /product="PE-PGRS family protein PE_PGRS22"
                     /protein_id="YP_177786.1"
                     /db_xref="GI:57116826 GeneID:885258"
                     /translation="MSFVIAAPEALVAVASDLAGIGSALAEANAAALAPTTALLAAGADEVSAAIAALFGAHGQAYQTVSAQASA
                     FHAQFVQALTGGGGAYAAAEAANVSAAQSTDQRLLDLINGPTQALLGRPLIGDGANGGPGQDGGPGGLLYGNGGNGGTSTTAGVA
                     GGNGGAAGLIGNGGAGGGGGAGAAGGNGGAGGWLYGNGGAGGAGGTSVIPGVAGGNGGAGGSAGLWGTGGAGGDGGNGRSGPVNV
                     AGSAGGNGGAGGAAGLFGDAGAGGNGGKGGAGGAAFSINFTAGDGGAGGAGGSGGHALLWGAGGAGGNGGSGGTGGAGGSTAGAG
                     GNGGAGGGGGTGGLLFGNGGAGGHGAAAGNGLAAGNGVSSSGGGGAGGTGGAGGDGGAGGAGGNARLWGVGGAGGAGGDGGAGGA
                     GGKGGSGLSGNANGGAGGDSGRGGTGGAGGEGGAAGLLVGTGGHGGDGGAGGAAVKGGDGGAAAGTGIAGAGGRGGAGGSGGSGG
                     DGGGGAAGPAGWLFGDGGAGGNGGAAAAGGAGGQAGGGGGNGGNGGNGGNGGNGGNGATGGWLYGNGGAGGQGATAGAGGAGANG
                     VSSTNGGGTGGNGGIGGTGGSGGAGGNAGLLGVGGAGGHGASGGAGDRGGAGGTGFISSDGGAGGDGGDGGNGGAGGTGGLLFGA
                     GGNGGPGGSGGAADIGGNGGAGNGGGTDGNGGNGGSGGGAGSGGDGGGAGGNGAWLFGNGGAGGGGGKGGNGAGGGLGGGSFGLP
                     GLNGSGGDGGDGGNGAPGGVLYGNGGAGGQGSSGGIGGPGATGGAGGKGGDGGDAQLIGDGGNGGNGGAGGTGGTPGPGGPGGSG
                     GLGGLLFGQTGTAGVSP"
     CDS             complement(1333695..1335383)
                     /gene="PE_PGRS23"
                     /locus_tag="Rv1243c"
                     /experiment="EXISTENCE: identified in proteomics study"
                     /note="Rv1243c, (MTV006.15c), len: 562 aa. PE_PGRS23,Member of the Mycobacterium
                     tuberculosis PE family, PGRS subfamily of gly-rich proteins (see Brennan & Delogu
                     2002)."
                     /codon_start=1
                     /transl_table=11
                     /product="PE-PGRS family protein PE_PGRS23"
                     /protein_id="YP_177798.1"
                     /db_xref="GI:57116845 GeneID:887109"
                     /translation="MEYLIAAQDVLVAAAADLEGIGSALAAANRAAEAPTTGLLAAGADEVSAAIASLFSGNAQAYQALSAQAAA
                     FHQQFVRALSSAAGSYAAAEAANASPMQAVLDVVNGPTQLLLGRPLIGDGANGGPGQNGGDGGLLYGNGGNGGSSSTPGQPGGRG
                     GAAGLIGNGGAGGAGGPGANGGAGGNGGWLYGNGGLGGNGGAATQIGGNGGNGGHGGNAGLWGNGGAGGAGAAGAAGANGQNPVS
                     HQVTHATDGADGTTGPDGNGTDAGSGSNAVNPGVGGGAGGIGGDGTNLGQTDVSGGAGGDGGDGANFASGGAGGNGGAAQSGFGD
                     AVGGNGGAGGNGGAGGGGGLGGAGGSANVANAGNSIGGNGGAGGNGGIGAPGGAGGAGGNANQDNPPGGNSTGGNGGAGGDGGVG
                     ASADVGGAGGFGGSGGRGGLLLGTGGAGGDGGVGGDGGIGAQGGSGGNGGNGGIGADGMANQDGDGGDGGNGGDGGAGGAGGVGG
                     NGGTGGAGGLFGQSGSPGSGAAGGLGGAGGNGGAGGGGGTGFNPGAPGDPGTQGATGANGQHGLNG"
     CDS             complement(1436860..1438671)
                     /gene="PE_PGRS24"
                     /locus_tag="Rv1325c"
                     /experiment="EXISTENCE: identified in proteomics study"
```

```
                     /note="Rv1325c, (MTCY130.10c), len: 603 aa. PE_PGRS24,Member of the Mycobacterium
                     tuberculosis PE family, PGRS subfamily of ala-, gly-rich proteins (see Brennan &
                     Delogu 2002), similar to many e.g. YQ04_MYCTU|P71933 hypothetical 63.1 kDa
                     glycine-rich protein (778 aa), FASTA scores: E(): 0, (52.3% identity in 724 aa
                     overlap). Predicted to be an outer membrane protein (See Song et al., 2008)."
                     /codon_start=1
                     /transl_table=11
                     /product="PE-PGRS family protein PE_PGRS24"
                     /protein_id="YP_177799.1"
                     /db_xref="GI:57116848 GeneID:886899"
                     /translation="MSFVIAAPETLVRAASDLANIGSTLGAANAAALGPTTELLAAGADEVSAAIASLFAAHGQAYQAVSAQMSA
                     FHAQFVQTFTAGAGAYASAEAAAAAPLEGLLNIVNTPTQLLLGRPLIGNGANGAPGTGQAGGAGGLLYGNGGAGGSGAPGQAGGP
                     GGAAGLFGNGGAGGAGGDGPGNGAAGGAGGAGGLLFGSGGAGGPGGVGNTGTGGLGGDGGAAGLFGAGGIGGAGGPGFNGGAGGA
                     GGRSGLFEVLAAGGAGGTGGLSVNGGTGGTGGTGGGGGLFSNGGAGGAGGFGVSGSAGGNGGTGGDGGIFTGNGGTGGTGGTGTG
                     NQLVGGEGGAGGAGGNAGILFGAGGIGGTGGTGLGAPDPGGTGGKGGVGGIGGAGALFGPGGAGGTGGFGASSADQMAGGIGGSG
                     GSGGAAKLIGDGGAGGTGGDSVRGAAGSGGTGGTGGLIGDGGAGGAGGTGIEFGSVGGAGGAGGNAAGLSGAGGAGGAGGFGETA
                     GDGGAGGNAGLLNGDGGAGGAGGLGIAGDGGNGGKGGKAGMVGNGGDGGAGGASVVANGGVGGSGGNATLIGNGGNGGNGGVGSA
                     PGKGGAGGTAGLLGLNGSPGLS"
     CDS             complement(1518022..1519752)
                     /gene="PE_PGRS25"
                     /locus_tag="Rv1396c"
                     /inference="protein motif:PROSITE:PS00017"
                     /note="Rv1396c, (MTCY21B4.13c), len: 576 aa. PE_PGRS25,Member of the Mycobacterium
                     tuberculosis PE family, PGRS subfamily of gly-rich proteins (see Brennan & Delogu
                     2002),strong similarity to many e.g. glycine rich protein MTCY130.10C|E245019 (603
                     aa), FASTA scores: opt: 1945, E(): 0, (57.5% identity in 619 aa overlap). Contains
                     PS00017 ATP/GTP-binding site motif A, similar to other PGRS-type sequences. This
                     region is a possible MT-complex-specific genomic island (See Becq et al., 2007)."
                     /codon_start=1
                     /transl_table=11
                     /product="PE-PGRS family protein PE_PGRS25"
                     /protein_id="YP_177809.1"
                     /db_xref="GI:57116861 GeneID:886745"
                     /translation="MSFLFAQPEMLGAAATDLASIGSAISTANAAAAAATTRVLAAGADEVSAAVAALFSGHAQTYQALRTQAAA
                     FHQQIVQTLTSTAGAYASAEAANVEQQLLGAINAPTMALLGRPLIGHGADGAPGTGQAGGAGGILYGNGGNGGSGATGQAGGAGG
                     AAGLIGHGGAGGLGGTGASGGAGGAGGWLWGNGGAGGNGGVGVAGDPGGVGGAGGAGGAAGLWGSGGSGGTGGQGGVGGGKSGDG
                     GTGGIGGAGGGGGWLHGDGGAGGHGGQGGTGVSSGGNGGAGGTGGDGRGLSGSGGAGGRGGQTGVGGKVGENNFGGAGGAGGTGG
                     LIGNGGAGGNGGQGAISGAGGAGGNAWLIGDGGAGGNGGDIRGQGGGAGGAGGAGGQLIGNGGTGGAGGTVTSPNGLGGAGGAGG
                     SAGLIGHGGTGGAGGHSAQGPDGNGGIGGAGGAGGNGGQLYGTGGTGGTGGKGGDGFGVFGKGGAGGTGGRGGAAGLIGDAGTGG
                     TGGKGGTAGEDGTGGNGGTGGNGGAAVLIGNGGGGGAGGNGGAGNDGTPGNGGGGGVGGTGGTLFGQPGQPGPPGQPGPA"
     CDS             complement(1564104..1565579)
                     /gene="PE_PGRS26"
                     /locus_tag="Rv1441c"
                     /note="Rv1441c, (MTCY493.13), len: 491 aa. PE_PGRS26,Member of the Mycobacterium
                     tuberculosis PE family, PGRS subfamily of gly-rich proteins (see Brennan & Delogu
                     2002),similar to Y0DP_MYCTU|Q50615 hypothetical glycine-rich 40.8 kDa protein (498
                     aa), fasta scores: opt: 1625, E(): 0,(55.2% identity in 518 aa overlap)."
                     /codon_start=1
                     /transl_table=11
                     /product="PE-PGRS family protein PE_PGRS26"
                     /protein_id="YP_177811.1"
                     /db_xref="GI:57116864 GeneID:886626"
                     /translation="MSNVMVVPGMLSAAAADVASIGAALSAANGAAAPTTAGVLAAGADEVSAAIASLFSGYARDYQALSAQMAR
                     FHQQFVQALTASVGSYAAAEAANASPLQALEQQVLAAINAPTQTLLGRPLIGNGADGLPGQNGGAGGLLWGNGGNGGAGDAAHPN
                     GGNGGDAGMFGNGGAGGAGYSPAAGTGAAGGAGGAGGAGGWLSGNGGAGGNGGTGASGADGGGGLPPVPASPGGNGGGGDAGGAA
                     GMFGTGGAGGTGGDGGAGGAGDSPNSGANGARGGDGGNGAAGGAGGRLFGNGGAGGNGGTAGQGGDGGTALGAGGIGGDGGTGGA
                     GGTGGTAGIGGSSAGAGGAGGDGGAGGTGGGSSMIGGKGGTGGNGGVGGTGGASALTIGNGSSAGAGGAGGAGGTGGTGGYIESL
                     DGKGQAGNGGNGGNGAAGGAGGGGTGAGGNGGAGGNGGDGGPSQGGGNPGFGGDGGTGGPGGVGVPDGIGGANGAQGKHG"
     CDS             complement(1576533..1580522)
                     /gene="PE_PGRS27"
                     /locus_tag="Rv1450c"
                     /note="Rv1450c, (MTCY493.04), len: 1329 aa. PE_PGRS27,Member of the Mycobacterium
                     tuberculosis PE family, PGRS subfamily of gly-rich proteins (see Brennan and
                     Delogu,2002), similar to Y03A_MYCTU|Q10637 hypothetical glycine-rich 49.6 kDa protein
                     (603 aa), fasta scores: opt: 2112, E(): 0, (56.5% identity in 630 aa overlap)."
                     /codon_start=1
                     /transl_table=11
                     /product="PE-PGRS family protein PE_PGRS27"
                     /protein_id="YP_177812.1"
```

```
                     /db_xref="GI:57116865 GeneID:886605"
                     /translation="MSLVIVAPETVAAAALDVARIGSSIGAANAAAAGSTTSVLAAGADEVSAAIATLFGSHAREYQAISTQVAA
                     FHDRFAQTLSAAVGSYVSAEATNAAPLATLEHNVLNALNAPTQALLGRPLIGDGAAGAPGTGQAGGAGGILWGNGGAGGSGAPGQ
                     VGGAGGAAGLFGTGGAGGAGGAGAAGGAGGSGGWLLGNGGVGGAGGQSLLGGATGGAGGNAGLFGVGGTGGPGGPGGPGGVGGTG
                     GAGGLGGTLYGAGGHGGAGGPGPIGGVGGHGGVGGAAGLLGVGGHGGAGGHGAEGVAGAAGEDLSPHGTSGGVGGDAGDGGTGGR
                     GGWLAGAGGAGGAGGVGGTGGAGGAGFSRALIVAGDNGGDPGAGGAGGTGGAGSTIGAHGAAGASPTSGGNGGAGGNGAHFSSGG
                     KAGGNGGAGGAGGLVGNGGAGGAGGNGAPGAPPSGGDPNGGGGGAGGAGGKGGDGGAQAGDGGAGGAGGKGGNGGNGATGATGLN
                     GLGAGADGTDGGKGGNGGAGGGGGAGGQGGKALAATHQDGSMGAGGAGGNGGAGGMGGDGGNGAKGTFDNGGDGVGGNGGNGGSR
                     GIGGAGGIGGAGSTAGADGARGATPTSGGNGGTGGNGANATVAGGAGGAGGKGGNGGLVGNGGAGGKGGDGMAGVAGSSPTTAGE
                     SGTSGQNGGAGGAGGAGGRGGDFGGDGGTGGAGGNGANGANATTPGAKGGDGGHGGPGAQGGNGGQGGPGGLAGNLFGQNGIQGV
                     GGSGGKGGAGGLAGDGGNGANGNFAFGDGNGGHGGNGGNPGAGGQGGSGGAGSTPGAKGAHGFTPTSGGDGGDGGNGGNSQVVGG
                     NGGDGGNGGNGGSAGTGGNGGRGGDGAFGGMSANATNPGENGPNGNPGGNGGAGGAGGAGLNGGNGGAGGNGGLGGFGGNGAAGA
                     NGVAVGAPGQPGGAGGHGGAGGNGGAGGNGGQGVVSDGAGGAGGAGGDGGAPGDGANGGNGQGAGAFAGGGGGRGGDGGNAGNAG
                     AGGPGGTGSTAGKAGPAGSILHDGGNGGHGGHGAASGGNGGPGGHGGNGGNGGTGANGGNGGIGGTGGAGSTGAKGVLGTNEGDG
                     GDGGRGGNGGRGGNGGQGLTGAGGNGGTGGTPGNGGNGGNGASGDLVTSPGDGGGGGRGGDAGRGGDAGLGGSSGPGGTPGDWGT
                     GGTGGTGGTGGQGANGGLTGGRGGTGGNGGNGNTGGTGGAGGTGGTGHNGSQPGMGGNGGAGGFGGNGFAGVGGRGGMGGSGGTG
                     GTGDAGPFGTGTGGTGGHGGQGGGGGFSILLGLGGLGGLGSPGSIATGTAGGAGGGGGFGGLGGGEFV"
     CDS             complement(1581899..1584124)
                     /gene="PE_PGRS28"
                     /locus_tag="Rv1452c"
                     /note="Rv1452c, (MTCY493.02), len: 741 aa. PE_PGRS28,Member of the Mycobacterium
                     tuberculosis PE family, PGRS subfamily of gly-rich proteins (see Brennan and
                     Delogu,2002), similar to Y03A_MYCTU|Q10637 hypothetical glycine-rich 49.6 kDa protein
                     (603 aa), fasta scores: opt: 2090, E(): 0, (56.3% identity in 641 aa overlap)."
                     /codon_start=1
                     /transl_table=11
                     /product="PE-PGRS family protein PE_PGRS28"
                     /protein_id="YP_177813.1"
                     /db_xref="GI:57116866 GeneID:886595"
                     /translation="MSLVIVTPETVAAAASDVARIGSSIGVANSAAAGSTTSVLAAGADEVSAAIATLFGSHAREYQAISTQVAA
                     FHDRFAQTLSAAVGSYVSAEATNAAPLATLEHNVLNALNAPTQALLGRPLIGDGAAGAPGTGQAGGAGGILWGNGGAGGSGAPGQ
                     VGGAGGAAGLFGTGGAGGAGGAGAAGGAGGSGGWLLGNGGVGGAGGQSLLGGATGGAGGNAGLFGVGGTGGPGGPGGPGGVGGTG
                     GAGGLGGTLYGAGGHGGAGGPGPIGGVGGHGGVGGAAGLLGVGGHGGAGGHGAEGVAGAAGEDLSPHGTSGGVGGDAGDGGTGGR
                     GGWLAGAGGAGGAGGVGGTGGAGGAGFSRALIVAGDNGGDGGNGGMGGAGGAGGPGGAGGLISLLGGQGAGGAGGTGGAGGVGGD
                     RGAGGPGNQAFNAGAGGAGGHGGDPGAGGAGGTGGAGSITGAQGAIGATPTSGGNGGAGGNGANATTAGTNGANGGPGGHGGLVG
                     NGGAGGNGANGAAGTNASDSGAVGGKGNSGGNGGQGGAGGDGGTLAGNGGAGGTGGRGADGGLGGSGAEGANATTAGERGQDGGK
                     GGNGGVGGTGGNAVAPGANGGHGGNGGNPGFSGAGGLGGLSGDGVTRAAQGATPDFADTGGKGGNGGNGANAVAPGGTGASGGAG
                     GNAGAGGKGGENIIGDGGGGNGGAGGKGGAGTLLGLTVFGDNGGAGVLGDSTDPDGSGGAGGAGGAGGDPTI"
     CDS             complement(1601504..1602616)
                     /gene="PE_PGRS29"
                     /locus_tag="Rv1468c"
                     /experiment="EXISTENCE: identified in proteomics study"
                     /note="Rv1468c, (MTV007.15c), len: 370 aa. PE_PGRS29,Member of the Mycobacterium
                     tuberculosis PE family, PGRS subfamily of gly-rich proteins (see citation below)."
                     /codon_start=1
                     /transl_table=11
                     /product="PE-PGRS family protein PE_PGRS29"
                     /protein_id="YP_177814.1"
                     /db_xref="GI:57116869 GeneID:886556"
                     /translation="MSFVVANTEFVSGAAGNLARLGSMISAANSAAAAQTTAVAAAGADEVSAAVAALFGAHGQTYQVLSAQAAA
                     FHSQFVQALSGGAQAYAAAEATNFGPLQPLFDVINAPTLALLNRPLIGNGADGTAANPNGQAGGLLIGNGGNGFSPAAGPGGNGG
                     AAGLLGHGGNGGVGALGANGGAGGTGGWLFGNGGAGGNSGGGGGAGGIGGSAVLFGAGGAGGISPNGMGAGGSGGNGGLFFGNGG
                     AGASSFLGGGGAGGRAFLFGDGGAGGAALSAGSAGRGGDAGFFYGNGGAGGSGAGGASSAHGGAGGQAGLFGNGGEGGDGGALGG
                     NGGNGGNAQLIGNGGDGGDGGGAGAPGLGGRGGLLLGLPGANGT"
     CDS             complement(1806205..1809240)
                     /gene="PE_PGRS30"
                     /locus_tag="Rv1651c"
                     /experiment="EXISTENCE: identified in proteomics study"
                     /note="Rv1651c, (MTCY06H11.16c), len: 1011 aa. PE_PGRS30,Member of the Mycobacterium
                     tuberculosis PE family, PGRS subfamily of gly-rich proteins (see citations
                     below),similar to many e.g. Q10637|Y03A_MYCTU hypothetical glycine-rich 49.6 kd
                     protein (603 aa), FASTA scores: opt: 1757, E(): 0, (50.8% identity in 714aa overlap).
                     The transcription of this CDS seems to be activated in macrophages (see Ramakrishnan
                     et al., 2000)."
                     /codon_start=1
                     /transl_table=11
                     /product="PE-PGRS family protein PE_PGRS30"
                     /protein_id="YP_177826.1"
                     /db_xref="GI:57116897 GeneID:885174"
```

```
                     /translation="MSFLLVEPDLVTAAAANLAGIRSALSEAAAAASTPTTALASAGADEVSAAVSRLFGAYGQQFQALNARAAT
                     FHAEFVSLLNGGAAAYTGAEAASVSSMQALLDAVNAPTQTLLGRPLIGNGADGVAGTGSNAGGNGGPGGILYGNGGNGGAGGNGG
                     AAGLIGNGGAGGAGGAGGAGGAGGTGGLLYGNGGAGGNGGSAAAAGGAGGNALLFGNGGNGGSGASGGAAGHAGTIFGNGGN
                     AGAGSGLAGADGGLFGNGGDGGSSTSKAGGAGGNALFGNGGDGGSSTVAAGGAGGNTLVGNGGAGGAGGTSGLTGSGVAGGAGGS
                     VGLWGSGGAGGDGGAATSLLGVGMNAGAGGAGGNAGLLYGNGGAGGAGGNGGDTTVPLFDSGVGGAGGAGGNASLFGNGGTGGVG
                     GKGGTSSDLASATSGAGGAGGAGGVGGLLYGNGGNGGAGGIGGAAINILANAGAGGAGGAAGSSFIGNGGNGGAGGAGGAAALFS
                     SGVGGAGGSGGTALLLGSGGAGGNGGTGGANSGSLFASPGGTGGAGGHGGAGGLIWGNGGAGGNGGNGGTTADGALEGGTGGIGG
                     TGGSAIAFGNGGQGGAGGTGGDHSGGNGIGGKGGASGNGGNAGQVFGDGGTGGTGGAGGAGSGTKAGGTGSDGGHGGNATLIGNG
                     GDGGAGGAGGAGSPAGAPGNGGTGGTGGVLFGQSGSSGPPGAAALAFPSLSSSVPILGPYEDLIANTVANLASIGNTWLADPAPF
                     LQQYLANQFGYGQLTLTALTDATRDFAIGLAGIPPSLQSALQALAAGDVSGAVTDVLGAVVKVFVSGVDASDLSNILLLGPVGDL
                     FPILSIPGAMSQNFTNVVMTVTDTTIAFSIDTTNLTGVMTFGLPLAMTLNAVGSPITTAIAFAESTTAFVSAVQAGNLQAAAAAL
                     VGAPANVANGFLNGEARLPLALPTSATGGIPVTVEVPVGGILAPLQPFQATAVIPVIGPVTVTLEGTPAGGIVPALVNYAPTQLA
                     QAIAP"
     CDS             1938967..1940823
                     /gene="PE_PGRS31"
                     /locus_tag="Rv1768"
                     /experiment="EXISTENCE: identified in proteomics study"
                     /note="Rv1768, (MTCY28.34), len: 618 aa. PE_PGRS31, Member of the Mycobacterium
                     tuberculosis PE family, PGRS subfamily of gly-rich proteins (see Brennan and Delogu,
                     2002), highly similar to Q50615 hypothetical 40.8 kDa protein (498 aa),FASTA scores:
                     opt: 1703, E(): 0, (57.4% identity in 566 aa overlap)."
                     /codon_start=1
                     /transl_table=11
                     /product="PE-PGRS family protein PE_PGRS31"
                     /protein_id="YP_177832.1"
                     /db_xref="GI:57116908 GeneID:885429"
                     /translation="MSYLVVVPELVAAAATDLANIGSSISAANAAAAAPTTALVAAGGDEVSAAIAALFGAHARAYQALSAQAAM
                     FHEQFVRALAAGGNSYAVAEAATAQSVQQDLLNLINAPTQALLGRPLIGNGANGLPGTGQNGGDGGILYGNGGNGGSGGVNQAGG
                     NGGNAGLWGNGGSGGAGGNATTAGRNGFNGGAGGSGGLLWGNGGAGGAGGNGGPAPLVGGVGTTGGAGGNGGGAGLFYGFGGAGG
                     NGGMGGVAPSTGPSMGILPAGGVGGPGGSGGASALAFGSGGVGGAGGLGGPTDGTVQGVGGFGGQGGNGGQSGLLFGNAGAGGAG
                     AAGGAGTGDTESFGGHGGAGGDGGAVGLIGNGGAGGTGSPGAVVGGNGGVGGLGGAGSPGGLLYGTGGAGGNGGPGGDGGTGATV
                     GFAGSGGFGGAGGIAQLFGTGGMGGSGGGIGAGTTTVVPPDVAPVGGTGGNGGRAGLLLGVGGMGGNGGATSVGGTLYAAGGNGG
                     DGGLVWGNGGTGGSGGAGGAGSVGNGGAGGNAALLFGNGGAGGAGGAGGIGAGGAGGFGAVLFGNGGAGGSGAPGGIGAGGNGGN
                     ALLVGNGGNGGAGTGGAAGGAGGSGGLLFGQNGMPGP"
     CDS             complement(1975311..1977230)
                     /gene="PE_PGRS32"
                     /locus_tag="Rv1803c"
                     /experiment="EXISTENCE: identified in proteomics study"
                     /inference="protein motif:PROSITE:PS00583"
                     /note="Rv1803c, (MTV049.25c), len: 639 aa. PE_PGRS32,Member of the Mycobacterium
                     tuberculosis PE family, PGRS subfamily of gly-rich proteins (see citation below).
                     Most similar to Rv1768|MTCY28.34|Z95890 (618 aa), FASTA scores: opt: 1827, E(): 0,
                     (53.5% identity in 664 aa overlap). Contains two PS00583 pfkB family of carbohydrate
                     kinases signatures 1. Predicted to be an outer membrane protein (See Song et al.,
                     2008)."
                     /codon_start=1
                     /transl_table=11
                     /product="PE-PGRS family protein PE_PGRS32"
                     /protein_id="YP_177842.1"
                     /db_xref="GI:57116918 GeneID:885730"
                     /translation="MWTSQMIVAPAFVDAAAKDLATIGSAISRANAEALVPITALLPAGADDVSAAIAALFATHGQAYQELSAHA
                     VAFHEQFVQLMSAGAAQYASAEAANSSPLQIVGQTALDAINSPVQTLTGRPLIGNGANGVAGTGQNGGDGGWLYGNGGNGGSGGT
                     GQNGGNGGSAGLWGSGGNGGQGGAGANGAAGQPGKAGGSGGNGGAGGWIYGHGGHGGAGGNGGNATAPGGASAGFDGGAGGNGGS
                     GGRGGLLFGNGGNGSVGGMGGQGTNDTAGDSAGSGGLGGNGGNGAQGGWLIGNGGQGGDSGAGGGTDSTQTGVMNGASGGSAGIA
                     GNGGDAGLVGNGGAGGNGGNGAAGSALGTTIFGGSGGVGGSGGDGGNGGWLFGSGASGGNGGQGGDAGTNGFAGFGGSAGGGGWV
                     GAVNFGPISVQGFGLFGHGGDGGNGGDVGAGSLSIQFGASGGDGGQGGVLYGNGGNGGNAGSGGGTGFEGSAGQGGAAILIGNGG
                     AGGNGATGGTGVGNIIQEAGGDGSDGGAGGSGGLLFGSGGAGGIGGAGGVGGSGNDGGNGGDGGQGGASGLGIGNGGPGGSGGTG
                     GAGGTGGSAGTGGAGGDGGNAALLIGTGGDGGDGVPPAPGGQGGKGGLIGLPGQNGQP"
     CDS             complement(1987729..1989225)
                     /gene="PE_PGRS33"
                     /locus_tag="Rv1818c"
                     /inference="protein motif:PROSITE:PS00583"
                     /note="Rv1818c, (MTCY1A11.25), len: 498 aa. PE_PGRS33,Member of the Mycobacterium
                     tuberculosis PE family, PGRS subfamily of gly-rich proteins, similar to many.
                     Contains 2 x PS00583 pfkB family of carbohydrate kinases signature 1. Supposedly
                     localised to the cell surface (see citations below)."
                     /codon_start=1
                     /transl_table=11
                     /product="PE-PGRS family protein PE_PGRS33"
```

```
                     /protein_id="YP_177846.1"
                     /db_xref="GI:57116923 GeneID:885551"
                     /translation="MSFVVTIPEALAAVATDLAGIGSTIGTANAAAAVPTTTVLAAAADEVSAAMAALFSGHAQAYQALSAQAAL
                     FHEQFVRALTAGAGSYAAAEAASAAPLEGVLDVINAPALALLGRPLIGNGANGAPGTGANGGDGGILIGNGGAGGSGAAGMPGGN
                     GGAAGLFGNGGAGGAGGNVASGTAGFGGAGGAGGLLYGAGGAGGAGGRAGGGVGGIGGAGGAGGNGGLLFGAGGAGGVGGLAADA
                     GDGGAGGDGGLFFGVGGAGGAGGTGTNVTGGAGGAGGNGGLLFGAGGVGGVGGDGVAFLGTAPGGPGGAGGAGGLFGVGGAGGAG
                     GIGLVGNGGAGGSGGSALLWGDGGAGGAGGVGSTTGGAGGAGGNAGLLVGAGGAGGAGALGGGATGVGGAGGNGGTAGLLFGAGG
                     AGGFGFGGAGGAGGLGGKAGLIGDGGDGGAGGNGTGAKGGDGGAGGGAILVGNGGNGGNAGSGTPNGSAGTGGAGGLLGKNGMNG
                     LP"
     CDS             complement(2014522..2016069)
                     /gene="PE_PGRS34"
                     /locus_tag="Rv1840c"
                     /note="Rv1840c, (MTCY359.33), len: 515 aa. PE_PGRS34,Member of the Mycobacterium
                     tuberculosis PE family, PGRS subfamily of gly-rich proteins (see citation below).
                     Similar to many e.g. Y03A_MYCTU|Q10637 hypothetical glycine-rich 49.6 kDa protein
                     (603 aa), FASTA scores: opt: 1693, E(): 0, (53.1% identity in 612 aa overlap); etc."
                     /codon_start=1
                     /transl_table=11
                     /product="PE-PGRS family protein PE_PGRS34"
                     /protein_id="YP_177847.1"
                     /db_xref="GI:57116924 GeneID:885753"
                     /translation="MSFVVAAPEVVVAAASDLAGIGSAIGAANAAAAVPTMGVLAAGADEVSAAVADLFGAHAQAYQALSAQAAL
                     FHEQFVHAMTAGAGAYAGAEAADAAALDVLNGPFQALFGRPLIGDGANGAPGQPGGPGGLLYGNGGNGGNGGIGQPGGAGGDAGL
                     IGNGGNGGIGGPGATGLAGGAGGVGGLLFGDGGNGGAGGLGTGPVGATGGIGGPGGAAVGLFGHGGAGGAGGLGKAGFAGGAGGT
                     GGTGGLLYGNGGNGGNVPSGAADGGAGGDARLIGNGGDGGSVGAAPTGIGNGGNGGNGGWLYGDGGSGGSTLQGFSDGGTGGNAG
                     MFGDGGNGGFSFFDGNGGDGGTGGTLIGNGGDGGNSVQTDGFLRGHGGDGGNAVGLIGNGGAGGAGSAGTGVFAPGGGSGGNGGN
                     GALLVGNGGAGGSGGPTQIPSVAVPVTGAGGTGGNGGTAGLIGNGGNGGAAGVSGDGTPGTGGNGGYAQLIGDGGDGGPGDSGGP
                     GGSGGTGGTLAGQNGSPGG"
     CDS             2145451..2147127
                     /gene="PE_PGRS35"
                     /locus_tag="Rv1983"
                     /experiment="EXISTENCE: identified in proteomics study"
                     /inference="protein motif:PROSITE:PS00141"
                     /note="Rv1983, (MTCY39.36c), len: 558 aa. PE_PGRS35, Member of the Mycobacterium
                     tuberculosis PE family, PGRS subfamily of gly-rich proteins (see Brennan & Delogu
                     2002). Similar to other PE proteins e.g. Rv0977, etc. Contains PS00141 Eukaryotic and
                     viral aspartyl proteases active site."
                     /codon_start=1
                     /transl_table=11
                     /product="PE-PGRS family protein PE_PGRS35"
                     /protein_id="YP_177854.1"
                     /db_xref="GI:57116933 GeneID:885921"
                     /translation="MSFLVVVPEFLTSAAADVENIGSTLRAANAAAAASTTALAAAGADEVSAAVAALFARFGQEYQAVSAQASA
                     FHQQFVQTLNSASGSYAAAEATIASQLQTAQHDLLGAVNAPTETLLGRPLIGDGAPGTATSPNGGAGGLLYGNGGNGYSATASGV
                     GGGAGGSAGLIGNGGAGGAGGPNAPGGAGGNGGWLLGNGGIGGPGGASSIPGMSGGAGGTGGAAGLLGWGANGGAGGLGDGVGVD
                     RGTGGAGGRGGLLYGGYGVSGPGGDGRTVPLEIIHVTEPTVHANVNGGPTSTILVDTGSAGLVVSPEDVGGILGVLHMGLPTGLS
                     ISGYSGGLYYIFATYTTTVDFGNGIVTAPTAVNVVLLSIPTSPFAISTYFSALLADPTTTPFEAYFGAVGVDGVLGVGPNAVGPG
                     PSIPTMALPGDLNQGVLIDAPAGELVFGPNPLPAPNVEVVGSPITTLYVKIDGGTPIPVPSIIDSGGVTGTIPSYVIGSGTLPAN
                     TNIEVYTSPGGDRLYAFNTNDYRPTVISSGLMNTGFLPFRFQPVYIDYSPSGIGTTVFDHPA"
     CDS             complement(2304255..2305025)
                     /gene="PE_PGRS37"
                     /locus_tag="Rv2126c"
                     /experiment="EXISTENCE: identified in proteomics study"
                     /note="Rv2126c, (MTCY261.22c), len: 256 aa. PE_PGRS37,Possible PE_PGRS pseudogene
                     fragment, similar to the Gly-rich C-terminus of many members of the Mycobacterium
                     tuberculosis PGRS family."
                     /codon_start=1
                     /transl_table=11
                     /product="PE-PGRS family protein PE_PGRS37"
                     /protein_id="YP_177862.1"
                     /db_xref="GI:57116949 GeneID:887791"
                     /translation="MIGDGANGGPGQPGGPGGLLYGNGGHGGAGAAGQDRGAGNSAGLIGNGGAGGAGGNGGIGGAGAPGGLGGD
                     GGKGGFADEFTGGFAQGGRGGFGGNGNTGASGGMGGAGGAGGAGGAGGLLIGDGGAGGAGGIGGAGGVGGGGGAGGTGGGGVASA
                     FGGGNAFGGRGGDGGDGGDGGTGGAGGARGAGGAGGAGGWLSGHSGAHGAMGSGGEGGAGGGGGARGEAGAGGGTSTGTNPGKAG
                     APGTQGDSGDPGPPG"
     CDS             complement(2340293..2341891)
                     /gene="PE_PGRS38"
                     /locus_tag="Rv2162c"
                     /experiment="EXISTENCE: identified in proteomics study"
```

```
                     /note="Rv2162c, (MTCY270.06), len: 532 aa. PE_PGRS38,Member of M. tuberculosis
                     PE_PGRS family (see citations below). FASTA score: Y03A_MYCTU Q 10637 hypothetical
                     glycine-rich 49.6 kDa protein (603 aa) op t: 1798 z-score: 1220.0 E(): 0; (55.4%
                     identity in 590 aa overlap). This region is a possible MT-complex-specific genomic
                     island (See Becq et al., 2007)."
                     /codon_start=1
                     /transl_table=11
                     /product="PE-PGRS family protein PE_PGRS38"
                     /protein_id="YP_177865.1"
                     /db_xref="GI:57116953 GeneID:887300"
                     /translation="MSFVIAAPEVMAAAATDLANIGSSISAASAAAAGPTMGILAAGADEVSVAISALFGSHAQGYQTLSAQLAA
                     YHNQFVRALNAGAGSYASAEAANVQQTLLNAINAPTQTLLGRPLIGNGADGGPGQNGGPGGLLYGNGGNGGAGDTANPNGGNGGS
                     AGLIGNGGAGGAGAATGAGGAGGNGGWLYGNGGPGGAAGLGTAGGVSPAGGAGGAAGLWGHGGAGGAGGSASGAPGAGGAGGDGG
                     RGGLLYGDGGAGGAGGNGSNGVTGVHGGNGGAGGAAGLIGNGGAGGDGGNGGLSNTGASGGAGGAGGAALIGNGGDGGHGGNGGH
                     GNSGGAGGAGGAGGAGGAGGHVGLIGNGGNGGAGGNGGNDNSSTLADAGSGGAGAAGGNGGLFYGNGGVGGRGGNGGFSSAGTSG
                     GDGGIGGAGGIGGLIGSGGGGGDGGNGGQAPTPGNAGDGGAGGNARLIGDGGRGGNGGEGGDGPPGVKGDGGNGGNGGNAVVIGN
                     GGNGGAGGFGIPVGSGGAGGSRGVLFGTPGANGADG"
     CDS             complement(2534720..2535961)
                     /gene="PE_PGRS39"
                     /locus_tag="Rv2340c"
                     /experiment="EXISTENCE: identified in proteomics study"
                     /note="Rv2340c, (MTCY98.09c), len: 413 aa. PE_PGRS39,Member of the Mycobacterium
                     tuberculosis PE_family, PGRS subfamily of gly-rich proteins (see citations
                     below),similar to others eg YI18_MYCTU|Q50615|Rv1818c|MTCY1A11.25 PE-PGRS family
                     protein from Mycobacterium tuberculosis (498 aa), FASTA scores: opt: 710, E():
                     1.4e-22, (41.0% identity in 368 aa overlap); O53884|Rv0872v|MTV043.65c PGRS-family
                     protein from Mycobacterium tuberculosis (606 aa), FASTA scores: opt: 708, E():
                     1.9e-22, (42.4% identity in 389 aa overlap); etc. This region is a possible
                     MT-complex-specific genomic island (See Becq et al.,2007)."
                     /codon_start=1
                     /transl_table=11
                     /product="PE-PGRS family protein PE_PGRS39"
                     /protein_id="YP_177869.1"
                     /db_xref="GI:57116973 GeneID:888961"
                     /translation="MSHVTAAPNVLAASAGELAAIGSTMRAANAAAAAPTAGVLAAGGDDVSAGIAALFGARAQAYQAISAQAAL
                     FHDRFVQILQEGAAAYAMAEAANALPLQKAQGVVSELAQDRTGGTGTGQSRGAGGFGGVGQAGGKGWDGGPIGNGQVGEQHGAGQ
                     LGSTDGNPGVAGAAHGSGVSASHGSGATGAAGVADPGGSGAGVGSAAGNGTGAGSADAVGGAGTGRDIVGSVRGDGGVGMASGDG
                     GLSTGAAGASAEGGLMPGFGGAPWVGGHWGLGGEGHSGAIGGVGEQVAPAVATAPAVSPATTSAVAAESGSTPATKAQAMHATTN
                     PGNAAHQGNPADPGNSARRADGGRDEQLLLLPLTSLRGLRHTLKKLSGLRARNGLLTASGDNASGSGRPWDRDQLLRALGLRPPG
                     HE"
     CDS             2564717..2564902
                     /gene="PE_PGRS40"
                     /locus_tag="Rv2371"
                     /note="Rv2371, (MTCY27.09c), len: 61 aa. PE_PGRS40, Short protein, member of the
                     Mycobacterium tuberculosis PE family, PGRS subfamily of gly-rich proteins (see
                     citation below), highly similar to N-terminal part of others e.g. AAK44356|MT0132
                     PE_PGRS family protein from Mycobacterium tuberculosis strain CDC1551 (561 aa), FASTA
                     scores: opt: 217, E(): 4.9e-08, (69.65% identity in 56 aa overlap); etc."
                     /codon_start=1
                     /transl_table=11
                     /product="PE-PGRS family protein PE_PGRS40"
                     /protein_id="YP_177875.1"
                     /db_xref="GI:57116979 GeneID:885141"
                     /translation="MSLVSVAPELVVTAVPDVARIGSSIGAPDTAAAARPTTSVLAAGADEVSADVVALFGWVAR"
     CDS             2605763..2606848
                     /gene="PE_PGRS41"
                     /locus_tag="Rv2396"
                     /gene_synonym="aprC"
                     /inference="protein motif:PROSITE:PS00583"
                     /note="Rv2396, (MTCY253.25c), len: 361 aa. PE_PGRS41,member of the Mycobacterium
                     tuberculosis PE family, PGRS subfamily of gly-rich proteins (see citation below).
                     Also known as aprC, acid and phagosome regulated protein C,restricted to M.
                     tuberculosis complex (See Abramovitch et al., 2011). Contains PS00583 pfkB family of
                     carbohydrate kinases signature 1. Predicted to be an outer membrane protein (See Song
                     et al., 2008)."
                     /codon_start=1
                     /transl_table=11
                     /product="PE-PGRS family protein PE_PGRS41"
                     /protein_id="YP_177878.1"
```

```
                     /db_xref="GI:57116982 GeneID:885517"
                     /translation="MSFLIASPEALAATATYLTGIGSAISAANAVAAAPTTEILAAGTDEVSTAISALFGAHAQAYQALSAHVAA
                     FHDQFVHTLTAGAGSYMAAEAAAASPLQALQLELLNAINAPTLALLGRPLIGDGTDAAPGSGGAGGAGGILIGNGGTGGASDLAG
                     TGRGGVGGAGGAGGLFGIGGAGGGCGSAVAIGGDGGAGGAGGVFSGGGAGGAGDAIGGSGGAGGTGGLLGGGGGAGGAGGAGGNG
                     GGASNSASIGGDGGSGGAGGMLYGAGGVGGNGGAAVAIGGDGGAGGRAGAIGNGGDGGNGGTSNTPGGSGGDGGNGGNAGLIGNG
                     GNGGNAEIVISGGSVAGTGGNGGLLLGFNGTNGLP"
     CDS             complement(2707680..2709764)
                     /gene="PE_PGRS42"
                     /locus_tag="Rv2487c"
                     /experiment="EXISTENCE: identified in proteomics study"
                     /note="Rv2487c, (MTV008.43c), len: 694 aa. PE_PGRS42,Member of the Mycobacterium
                     tuberculosis PE family, PGRS subfamily of Gly-rich proteins (see citation
                     below),similar to many e.g. AAK47245|MT2919 PE_PGRS family protein from Mycobacterium
                     tuberculosis strain CDC1515 (663 aa),FASTA scores: opt: 2317, E(): 2.3e-84, (58.35%
                     identity in 622 aa overlap); etc."
                     /codon_start=1
                     /transl_table=11
                     /product="PE-PGRS family protein PE_PGRS42"
                     /protein_id="YP_177886.1"
                     /db_xref="GI:57116995 GeneID:887909"
                     /translation="MSLVIATPQLLATAALDLASIGSQVSAANAAAAMPTTEVVAAAADEVSAAIAGLFGAHARQYQALSVQVAA
                     FHEQFVQALTAAAGRYASTEAAVERSLLGAVNAPTEALLGRPLIGNGADGTAPGQPGAAGGLLFGNGGNGAAGGFGQTGGSGGAA
                     GLIGNGGNGGAGGTGAAGGAGGNGGWLWGNGGNGGVGGTSVAAGIGGAGGNGGNAGLFGHGGAGGTGGAGLAGANGVNPTPGPAA
                     STGDSPADVSGIGDQTGGDGGTGGHGTAGTPTGGTGGDGATATAGSGKATGGAGGDGGTAAAGGGGGNGGDGGVAQGDIASAFGG
                     DGGNGSDGVAAGSGGGSGGAGGGAFVHIATATSTGGSGGFGGNGAASAASGADGGAGGAGGNGGAGGLLFGDGGNGGAGGAGGIG
                     GDGATGGPGGSGGNAGIARFDSPDPEAEPDVVGGKGGDGGKGGSGLGVGGAGGTGGAGGNGGAGGLLFGNGGNGGNAGAGGDGGA
                     GVAGGVGGNGGGGGTATFHEDPVAGVWAVGGVGGDGGSGGSSLGVGGVGGAGGVGGKGGASGMLIGNGGNGGSGGVGGAGGVGGA
                     GGDGGNGGSGGNASTFGDENSIGGAGGTGGNGGNGANGGNGGAGGIAGGAGGSGGFLSGAAGVSGADGIGGAGGAGGAGGAGGSG
                     GEAGAGGLTNGPGSPGVSGTEGMAGAPG"
     CDS             complement(2713633..2718615)
                     /gene="PE_PGRS43"
                     /locus_tag="Rv2490c"
                     /experiment="EXISTENCE: identified in proteomics study"
                     /note="Rv2490c, (MTV008.46c), len: 1660 aa. PE_PGRS43,Member of the Mycobacterium
                     tuberculosis PE family,PGRS-subfamily of Gly-rich proteins (see Brennan and Delogu,
                     2002), similar to many e.g. AAK47971|MT3612.1 PE_PGRS family protein from
                     Mycobacterium tuberculosis strain CDC1551 (1715 aa), FASTA scores: opt: 5161, E():
                     1.5e-187, (51.7% identity in 1752 aa overlap); etc."
                     /codon_start=1
                     /transl_table=11
                     /product="PE-PGRS family protein PE_PGRS43"
                     /protein_id="YP_177887.1"
                     /db_xref="GI:57116996 GeneID:887941"
                     /translation="MSYVIATPEMMATAAFDLARIGSQVSAASAVAAMPTTEVVAAGADEVSAGIAALFSAHAQEYQALSAQAAA
                     FHDQFVHTLTAAARWYTATEIANAAAMRVVLGAVNAPTQTLLGRPLIGDGAHGTAPGQPGGAGGLLFGNGGNGAAGAVGQVGGAG
                     GAAGLFGIGGAGGAGGAGAPGGTGGTGGWLAGGGGVGGMGGAGGGAGGAGGNAGLFGNGGAGGAGGAGGGAGGAGGNAGWFGHGG
                     AGGVGGVGAAGANGATPGQDGAAGVAGSDDGAGGDGLAGSDGGDGGAGGVGGNGGRGGWLLGNGGAGGVGGVGGAGGAGAAGGAG
                     GAGATGINGPAGISAAGGDGGAGGNGGAGGNGGVGGAGGAGGSAGLLGYVGRAGDGGAGGGGGLGGAPGDGGAGGNGGSWLAAGD
                     GGAGGHGGDPGLGGAGGAGGASGGAGARAGANGLAAGNDGPVSGGNGGKGGNGAHAPVAGGHGGNGGAGGNGGLVGDGGAGGHGG
                     DGAAGAGYADMTAIFLGSSGTPGEDGGNGGAGGAGGAGGAHAGDGGAGGAGGNGGAGGAGGNGAHGFNAVLVSDGGNGGDGGAGG
                     RGGDGGAGGAGGDAPAGRAGSQGVGGDGGAGGAGGAPGNGGSGGRGDMAFKDGDGGAGGDGGDPGAGGKGGAGGAGATEGVTGAT
                     GATVHSGGNGGKGGNGADATVAGANGGKGGAGGNGGLVGDGGAGGDGGSGAAGANGANVGEDGADGTLSGQPGEGSEANGGQGGV
                     GGGGAGGAGGDGGAGSSALGSGGNGGRGDAGQAGGAGGAGGAGGSVSGDGGPGGKGGAGGAGGAGASGGGGGKGASGADSAE
                     AVGGAGGKGGDGGVGGVGGDGGPGGDGGAGGAAPAGQVGSHGVGGVGGDGGLGGAGGNGGDGGHGSDGGDGGDGGDPGAGGLGGL
                     GGDSGNGTRAASGVDASDHGPGSGGNGGNGGNGAQASVAGGAGGNGGDGGNAGRVGDGGAGGNGGDGAAGANGANSGAPGSDALA
                     LGQPGGNGGQGDAGQAGGAGGAGGAGGAGGSVSGDGGAGGNGGAGGNGGVGASGGAGARGANGIDSIGGTGGAGGGGGDGGAGGV
                     GGHGGDGGVGGAAPSGTVGSHGTGGVGGDGGLGGAGGVGGAGGNGGIGITVGGAGGAGGNGGDPGAGGRGGLGGDSGNGTSAANG
                     VDASKHGPLTGGDGGVGGNGAKAAAAGGDGGQGGDGGNAGLFGDGGAGGDGADGTAAEALGGDGGAGGAGGKGGDAGDIGDGGDG
                     GKGGDGAHGALGGLTVAGGNGGAGGAGGAGGAGGAFLGDGGNGGAGGQGGAGRGGSPGGGGGVGGHGGAGGDAGMNGGGGTGGQG
                     GNGAAGGAGWSPDSDLKGFDGFDGGSGGAGGDGGAGGAGGTQTGDGGDGGAGGLGGAGGVGGNGVDGFDINETTGRDGGDGGDGG
                     YGGWGGAGGNGGAGGSAPAGEVGNRGVGGDGGDGGSGGDAGNGGLGGDGFTYLADFDGEPGGDGGDGGDGGWGRPGGQGGFGSTS
                     GAHGKAGFGAPGGDGGDGGNGGHGGDGNGSFADAGDGGPGGNGGNGGLGGAGRDGGAPGGDGGDGGTGGSGGFGAPPPRSIGGGD
                     GGDGGRGGDGGRGAGGLTSGGVGSSGESGGSGNGRGDPGSGGSGGEGGEGGPSISVNVT"
     CDS             2833930..2835561
                     /gene="PE_PGRS44"
                     /locus_tag="Rv2591"
                     /inference="protein motif:PROSITE:PS00583"
                     /note="Rv2591, (MTCY227.10c), len: 543 aa. PE_PGRS44,Member of the Mycobacterium
                     tuberculosis PE family, PGRS subfamily of gly-rich proteins (see citation below),
```

```
                     highly similar to others e.g. O53845|Rv0834c|MTV043.26c from Mycobacterium
                     tuberculosis (882 aa), FASTA scores: opt: 1813, E(): 5.8e-66, (55.3% identity in 568
                     aa overlap). Equivalent to AAK46982 from Mycobacterium tuberculosis strain CDC1551
                     (505 aa) but longer 38 aa. Contains PS00583 pfkB family of carbohydrate kinases
                     signature 1."
                     /codon_start=1
                     /transl_table=11
                     /product="PE-PGRS family protein PE_PGRS44"
                     /protein_id="YP_177891.1"
                     /db_xref="GI:57117002 GeneID:887992"
                     /translation="MSFVTAAPEMLATAAQNVANIGTSLSAANATAAASTTSVLAAGADEVSQAIARLFSDYATHYQSLNAQAAA
                     FHHSFVQTLNAAGGAYSSAEAANASAQALEQNLLAVINAPAQALFGRPLIGNGANGTAASPNGGDGGILYGNGGNGFSQTTAGVA
                     GGAGGSAGLIGNGGNGGAGGAGAAGGAGGAGGWLLGNGGAGGPGGPTDVPAGTGGAGGAGGDAPLIGWGGNGGPGGFAAFGNGGA
                     GGNGGASGSLFGVGGAGGVGGSSEDVGGTGGAGGAGRGLFLGLGGDGGAGGTSNNNGGDGGAGGTAGGRLFSLGGDGGNGGAGTA
                     IGSNAGDGGAGGDSSALIGYAQGGSGGLGGFGESTGGDGGLGGAGAVLIGTGVGGFGGLGGGSNGTGGAGGAGGTGATLIGLGAG
                     GGGGIGGFAVNVGNGVGGLGGQGGQGAALIGLGAGGAGGAGGATVVGLGGNGGDGGDGGGLFSIGVGGDGGNAGNGAMPANGGNG
                     GNAGVIANGSFAPSFVGFGGNGGNGVNGGTGGSGGILFGANGANGPS"
     CDS             complement(2854236..2855621)
                     /gene="PE_PGRS45"
                     /locus_tag="Rv2615c"
                     /note="Rv2615c, (MTCY01A10.19), len: 461 aa. PE_PGRS45,Member of the Mycobacterium
                     tuberculosis PE family, PGRS subfamily of gly-rich proteins (see citation below),
                     highly similar to many e.g. P71664|Rv1396c|MTCY21B4.13c from Mycobacterium
                     tuberculosis (576 aa), FASTA scores: opt: 1629, E(): 4.8e-58, (56.65% identity in 482
                     aa overlap). Equivalent to AAK47006 from Mycobacterium tuberculosis strain CDC1551
                     (476 aa) but shorter 15 aa."
                     /codon_start=1
                     /transl_table=11
                     /product="PE-PGRS family protein PE_PGRS45"
                     /protein_id="YP_177895.1"
                     /db_xref="GI:57117008 GeneID:888215"
                     /translation="MSFVNVAPQLVSTAAADAARIGSAINTANTAAAATTQVLAAAQDEVSTAIAALFGSHGQHYQAISAQVAAY
                     QQRFVLALSQAGSTYAVAEAASATPLQNVLDAINAPVQSLTGRPLIGDGANGIDGTGQAGGNGGWLWGNGGNGGSGAPGQAGGAG
                     GAAGLIGNGGAGGAGGQGLPFEAGANGGAGGAGGWLFGNGGAGGNGGIGGAGTNLAIGGHGGNGGNAGLIGAGGTGGAGGTGGGE
                     PSAGASGGNGGNGGNGGLLIGNSGDGGAAGNGAGISQNGPASGFGGNGGHAGTTGLIGNGGNGGAGGAGGDVSADFGGVGFGGQG
                     GNGGAGGLLYGNGGAGGNGGAAGSPGSVTAFGGNGGSGGSGGNGGNALIGNAGAGGSAGAGGNGASAGTAGGSGGDGGKGGNGGS
                     VGLIGNGGNGGNGGAGSLFNGAPGFGGPGGSGGASLLGPPGLAGTNGADG"
     CDS             complement(2870741..2873077)
                     /gene="PE_PGRS46"
                     /locus_tag="Rv2634c"
                     /experiment="EXISTENCE: identified in proteomics study"
                     /note="Rv2634c, (MTCY441.04c), len: 778 aa. PE_PGRS46,Member of the Mycobacterium
                     tuberculosis PE family, PGRS subfamily of gly-rich proteins (see citation below),
                     highly similar to many e.g. O53553|YZ08_MYCTU|Rv3508|MTV023.15 from Mycobacterium
                     tuberculosis (1901 aa), FASTA scores: opt: 2553, E(): 2.2e-93, (53.8% identity in 866
                     aa overlap). Equivalent to AAK47026 from Mycobacterium tuberculosis strain CDC1551
                     (788 aa) but shorter 10 aa."
                     /codon_start=1
                     /transl_table=11
                     /product="PE-PGRS family protein PE_PGRS46"
                     /protein_id="YP_177896.1"
                     /db_xref="GI:57117010 GeneID:888573"
                     /translation="MSFVIAVPEALTMAASDLANIGSTINAANAAAALPTTGVVAAAADEVSAAVAALFGSYAQSYQAFGAQLSA
                     FHAQFVQSLTNGARSYVVAEATSAAPLQDLLGVVNAPAQALLGRPLIGNGANGADGTGAPGGPGGLLLGNGGNGGSGAPGQPGGA
                     GGDAGLIGNGGTGGKGGDGLVGSGAAGGVGGRGGWLLGNGGTGGAGGAAGATLVGGTGGVGGATGLIGSGGFGGAGGAAAGVGTT
                     GGVGGSGGVGGVFGNGGFGGAGGLGAAGGVGGAASYFGTGGGGGVGGDGAPGGDGGAGPLLIGNGGVGGLGGAGAAGGNGGAGGM
                     LLGDGGAGGQGGPAVAGVLGGMPGAGGNGGNANWFGSGGAGGQGGTGLAGTNGVNPGSIANPNTGANGTDNSGNGNQTGGNGGPG
                     PAGGVGEAGGVGGQGGLGESLDGNDGTGGKGGAGGTAGTDGGAGGAGGAGGIGETDGSAGGVATGGEGGDGATGGVDGGVGGAGG
                     KGGQGHNTGVGDAFGGDGGIGGDGNGALGAAGGNGGTGGAGGNGGRGGMLIGNGGAGGAGGTGGTGGGGAAGFAGGVGGAGGEGL
                     TDGAGTAEGGTGGLGGLGGVGGTGGMGGSGGVGGNGGAAGSLIGLGGGGGAGGVGGTGGIGGIGGAGGNGGAGGAGTTTGGGATI
                     GGGGGTGGVGGAGGTGGTGGAGGTTGGSGGAGGLIGWAGAAGGTGAGGTGGQGGLGGQGGNGGNGGTGATGGQGGDFALGGNGGA
                     GGAGGSPGGSSGIQGNMGPPGTQGADG"
     CDS             2964550..2966127
                     /gene="PE_PGRS47"
                     /locus_tag="Rv2741"
                     /experiment="EXISTENCE: identified in proteomics study"
                     /note="Rv2741, (MTV002.06), len: 525 aa. PE_PGRS47, Member of the M. tuberculosis PE
                     family, PGRS subfamily of gly-rich proteins (see citation below), highly similar to
                     others e.g. Q10637|YD25_MYCTU|Rv1325c|MT1367|MTCY130.10c hypothetical PE-PGRS family
```

```
                     protein (603 aa), FASTA scores: opt: 1936, E(): 1.1e-71, (56.95% identity in 611 aa
                     overlap). Predicted to be an outer membrane protein (See Song et al., 2008). This
                     region is a possible MT-complex-specific genomic island (See Becq et al.,2007)."
                     /codon_start=1
                     /transl_table=11
                     /product="PE-PGRS family protein PE_PGRS47"
                     /protein_id="YP_177902.1"
                     /db_xref="GI:57117018 GeneID:888339"
                     /translation="MSFVIAAPEFLTAAAMDLASIGSTVSAASAAASAPTVAILAAGADEVSIAVAALFGMHGQAYQALSVQASA
                     FHQQFVQALTAGAYSYASAEAAAVTPLQQLVDVINAPFRSALGRPLIGNGANGKPGTGQDGGAGGLLYGSGGNGGSGLAGSGQKG
                     GNGGAAGLFGNGGAGGAGASNQAGNGGAGGNGGAGGLIWGTAGTGGNGGFTTFLDAAGGAGGAGGAGGLFGAGGAGGVGGAALGG
                     GAQAAGGNGGAGGVGGLFGAGGAGGAGGFSDTGGTGGAGGAGGLFGPGGGSGGVGGFGDTGGTGGDGGSGGLFGVGGAGGHGGFG
                     SAAGGDGGAGGAGGTVFGSGGAGGAGGVATVAGHGGHGGNAGLLYGTGGAGGAGGFGGFGGDGGDGGIGGLVGSGGAGGSGGTGT
                     LSGGRGGAGGNAGTFYGSGGAGGAGGESDNGDGGNGGVGGKAGLVGEGGNGGDGGATIAGKGGSGGNGGNAWLTGQGGNGGNAAF
                     GKAGTGSVGVGGAGGLLEGQNGENGLLPS"
     CDS             3070570..3072417
                     /gene="PE_PGRS48"
                     /locus_tag="Rv2853"
                     /experiment="EXISTENCE: identified in proteomics study"
                     /note="Rv2853, (MTCY24A1.04c), len: 615 aa. PE_PGRS48,Member of the Mycobacterium
                     tuberculosis PE family, PGRS subfamily of gly-rich proteins (see citation below),
                     highly similar to many e.g. O53884|Rv0872c|MTV043.65c from Mycobacterium tuberculosis
                     (606 aa), FASTA scores: opt: 1405, E(): 1.4e-97, (64.6% identity in 619 aa overlap).
                     Equivalent to AAK47245 from Mycobacterium tuberculosis strain CDC1551 (663 aa) but
                     shorter 48 aa."
                     /codon_start=1
                     /transl_table=11
                     /product="PE-PGRS family protein PE_PGRS48"
                     /protein_id="YP_177909.1"
                     /db_xref="GI:57117029 GeneID:888171"
                     /translation="MLYVVASPDLMTAAATNLAEIGSAISTANGAAALPTVEVVAAAADEVSTQIAALFGAHARSYQTLSTQAAA
                     FHSRFVQALTTAAASYASVEAANASPLQVALDVINAPAQTLLGRPLIGNGADGSTPGQAGGPGGLLYGNGGNGAAGGPNQAGGAG
                     GNAGLIGNGGAGGAGGVGAVGGKRGTGGLLFGNGGAGGQGGLGLAGINGGSGGQGGHGGNAILFGQGGAGGPGGTGAMGVAGTNP
                     TPIGTAAPGSDGVNQIGNGGNTDLTGGAGGDGNAGSTTVNGGNGGTGGAARNSSGGTGNSFGGAGGAGGDGANGGDGGAGGEALT
                     EGGATAVSGAGGKGGNAEASGGAGGNGGKGGFAQATTSVTGGNGGNGGNGHDSNAPGGAGGSGGVGGDGGRGGLLAGNGGTGGAG
                     GNGGTGGAGAPGGAGGAGGKADIANSLGDNATVTGGNGGTGGDGGSALGTGGAGGAGGLGGHGGAGGLLIGNGGAGGAGGLGGAG
                     GAGGAGGEGGAGGAGGEAIPGGASTNSAGGDGGAGGTGGNGGDGGAGGAPGLGGAGGAGGWLIGQSGSTGGGGAGGAGGAGGAGG
                     AGGSGGAGGHGDTTSGKNGSSGTAGFDGNPGQPG"
```

```
     CDS             complement(3549875..3551329)
                     /gene="PE_PGRS49"
                     /locus_tag="Rv3344c"
                     /note="Rv3344c, (MTV016.44c), len: 484 aa. PE_PGRS49,Member of the Mycobacterium
                     tuberculosis PE family, PGRS subfamily of gly-, ala-rich proteins (see Brennan and
                     Delogu, 2002). Appears to be a gene fragment, should be in-frame with following ORF,
                     MTV016.45c, frameshift required around 49595 but could not be found on checking BAC
                     and cosmid clones. Similar to many from Mycobacterium tuberculosis strains H37Rv and
                     CDC1551 e.g. O53557|Rv3512|MTV023.19 (1079 aa), FASTA scores: opt: 1595,E(): 1.8e-54,
                     (52.0% identity in 544 aa overlap)."
                     /codon_start=1
                     /transl_table=11
                     /product="PE-PGRS family protein PE_PGRS49"
                     /protein_id="YP_177961.1"
                     /db_xref="GI:57117093 GeneID:888115"
                     /translation="AQASPAAHGGSGGAGGNGGAGSAGNGGAGGAGGNGGAGGNGGGDAGNAGSGGNGGKGGDGVGPGSTGGAG
                     GKGGAGANGGSSNGNARGGNAGNGGHGGAGGSGDTGGAGGAGGQGGFGGTGGSGSGIGGGAGGNGGNGGAGGTGVVLGGKGGDGG
                     NGDHGGPATNPGSGSRGGAGGSGGNGGAGGNATGSGGKGGAGGNGGDGSFGATSGPASIGVTGAPGGNGGKGGAGGSNPNGSGGD
                     GGKGGNGGAGGNGGSIGANSGIVGGSGGAGGAGGAGGNGSLSSGEGGKGGDGGHGGDGVGGNSSVTQGGSGGGGGAGGAGGSGFF
                     GGKGGFGGDGGQGGPNGGGTVGTVAGGGGNGGVGGRGGDGVFAGAGGQGGLGGQGGNGGGSTGGNGGLGGAGGGGGNAPDGGFGG
                     NGGKGGQGGIGGGTQSATGLGGDGGDGGDGGNGGNSGAKAGGAGGKGQAGQPNSGTEPGFGGDGGLGGAGATP"
     CDS             complement(3551049..3555665)
                     /gene="PE_PGRS50"
                     /locus_tag="Rv3345c"
                     /experiment="EXISTENCE: identified in proteomics study"
                     /note="Rv3345c, (MTV004.01c-MTV016.45c), len: 1538 aa. PE_PGRS50, Member of the
                     Mycobacterium tuberculosis PE family, PGRS subfamily of gly-rich proteins (see
                     Brennan and Delogu, 2002). Similar to AAK47791 from strain CDC1551 but with some big
                     gaps (after residues 501 and 1419; and for AAK47791 after residue 991). Similar to
                     many from Mycobacterium tuberculosis strains H37Rv and CDC1551."
                     /codon_start=1
                     /transl_table=11
                     /product="PE-PGRS family protein PE_PGRS50"
                     /protein_id="YP_177962.1"
                     /db_xref="GI:57117094 GeneID:888114"
                     /translation="MVMSLMVAPELVAAAAADLTGIGQAISAANAAAAGPTTQVLAAAGDEVSAAIAALFGTHAQEYQALSARVA
                     TFHEQFVRSLTAAGSAYATAEAANASPLQALEQQVLGAINAPTQLWLGRPLIGDGVHGAPGTGQPGGAGGLLWGNGGNGGSGAAG
                     QVGGPGGAAGLFGNGGSGGSGGAGAAGGVGGSGGWLNGNGGAGGAGGTGANGGAGGNAWLFGAGGSGGAGTNGGVGGSGGFVYGN
                     GGAGGIGGIGGIGGNGGDAGLFGNGGAGGAGAAGLPGAAGLNGGDGSDGGNGGTGGNGGRGGLLVGNGGAGGAGGVGGDGGKGGA
                     GDPSFAVNNGAGGNGGHGGNPGVGGAGGAGGLLAGAHGAAGATPTSGGNGGDGGIGATANSPLQAGGAGGNGGHGGLVGNGGTGG
                     AGGAGHAGSTGATGTALQPTGGNGTNGGAGGHGGNGGNGGAQHGDGGVGGKGGAGGSGGAGGNGFDAATLGSPGADGGMGGNGGK
                     GGDGGKAGDGGAGAAGDVTLAVNQGAGGDGGNGGEVGVGGKGGAGGVSANPALNGSAGANGTAPTSGGNGGNGGAGATPTVAGEN
                     GGAGGNGGHGGSVGNGGAGGAGGNGVAGTGLALNGGNGGNGGIGGNGGSAAGTGGDGGKGGNGGAGANGQDFSASANGANGGQGG
                     NGGNGGIGGKGGDAFATFAKAGNGGAGGNGGNVGVAGQGGAGGKGAIPAMKGATGADGTAPTSGGDGGNGGNGASPTVAGGNGGD
                     GGKGGSGGNVGNGGNGGAGGNGAAGQAGTPGPTSGDSGTSGTDGGAGGNGGAGGAGGTLAGHGGNGGKGGNGGQGGIGGAGERGA
                     DGAGPNANGANGENGGSGGNGGDGGAGGNGGAGGKAQAAGYTDGATGTGGDGGNGGDGGKAGDGGAGENGLNSGAMLPGGGTVGN
                     PGTGGNGGNGGNAGVGGTGGKAGTGSLTGLDGTDGITPNGGNGGNGGNGGKGGTAGNGSGAAGGNGGNGGSGLNGGDAGNGGNGG
                     GALNQAGFFGTGGKGGNGGNGGAGMINGGLGGFGGAGGGGAVDVAATTGGAGGNGGAGGFASTGLGGPGGAGGPGGAGDFASGVG
                     GVGGAGGDGGAGGVGGFGGQGGIGGEGRTGGNGGSGGDGGGGISLGGNGGLGGNGGVSETGFGGAGGNGGYGGPGGPEGNGGLGG
                     NGGAGGNGGVSTTGGDGGAGGKGGNGGDGGNVGLGGDAGSGGAGGNGGIGTDAGGAGGAGGAGGNGGSSKSTTTGNAGSGGAGGN
                     GGTGLNGAGGAGGAGGNAGVAGVSFGNAVGGDGGNGGNGGHGGDGTTGGAGGKGGNGSSGAASGSGVVNVTAGHGGNGGNGGNGG
                     NGSAGAGGQGGAGGSAGNGGHGGGATGGDGGNGGNGGNSGNSTGVAGLAGGAAGAGGNGGGTSSAAGHGGSGGSGGSGTTGGAGA
                     AGGNGGAGAGGGSLSTGQSGGPRRQRWCRWQRRRWLGRQRRRRWCRWQRRCRRQRWRWRCRQRRLRRQWRQGRRRCRPWLHRRRG
                     RQGRRWRQRRFQQRQRSRWQRR"
     CDS             3570834..3572600
                     /gene="PE_PGRS51"
                     /locus_tag="Rv3367"
                     /experiment="EXISTENCE: identified in proteomics study"
                     /note="Rv3367, (MTV004.25), len: 588 aa. PE_PGRS51, Member of the Mycobacterium
                     tuberculosis PE family, PGRS subfamily of gly-rich proteins (see Brennan & Delogu
                     2002). Similar to many from Mycobacterium tuberculosis strains H37Rv and CDC1551 e.g.
                     O50415|Rv3388|MTV004.46 (731 aa), FASTA scores: opt: 1999, E(): 7.2e-72, (55.0%
                     identity in 620 aa overlap); and MTV004_44, MTV043_65, MTV006_15,
                     MTCY63_2,MTCY21B4_13, MTV023_21, MTV008_43, MTCY24A1_4, MTV023_15; etc. Equivalent to
                     AAK47814 from Mycobacterium tuberculosis strain CDC1551 (628 aa) but shorter 37 aa."
                     /codon_start=1
                     /transl_table=11
                     /product="PE-PGRS family protein PE_PGRS51"
                     /protein_id="YP_177965.1"
```

```
                     /db_xref="GI:57117098 GeneID:887404"
                     /translation="MSFVVAVPEALAAAASDVANIGSALSAANAAAAAGTTGLLAAGADEVSAALASLFSGHAVSYQQVAAQATA
                     LHDQFVQALTGAGGSYALTEAANVQQNLLNAINAPTQALLGRPLIGDGAVGTASSPDGQDGGLLFGNGGAGYNSAATPGMAGGNG
                     GNAGLIGNGGTGGSGGAGAAGGAGGSGGWLYGNGGNGGIGGNAIVAGGAGGNGGAGGAAGLWGSGGSGGQGGNGLTGNDGVNPAP
                     VTNPALNGAAGDSNIEPQTSVLIGTQGGDGTPGGAGVNGGNGGAGGDANGNPANTSIANAGAGGNGAAGGDGGANGGAGGAGGQA
                     ASAGSSVGGDGGNGGAGGTGTNGHAGGAGGAGGAGGRGGWLVGNGGNGGNGAAGGNGAIGGTGGAGGVPANQGGNSALGTQPVGG
                     DGGDGGNGGTGGTGGRGGDGGSGGAGGASGWLMGNGGNGGNGGTGGSGGVGGNGGIGGDGAGGGNATSTSSIPFDAHGGNGGAGG
                     DAGHGGTGGDGGDGGHAGTGGRGGLLAGQHANSGNGGGGGTGGAGGTHGTPGSGNAGGTGTGNADSTNGGPGSDGLGGDAFNGSR
                     GTDGNPG"
     CDS             3593919..3596114
                     /gene="PE_PGRS52"
                     /locus_tag="Rv3388"
                     /experiment="EXISTENCE: identified in proteomics study"
                     /note="Rv3388, (MTV004.46), len: 731 aa. PE_PGRS52, Member of the M. tuberculosis PE
                     family, PGRS subfamily of gly-rich proteins (see citation below), similar to many
                     PE-family proteins from Mycobacterium tuberculosis strains H37Rv and CDC1551 e.g.
                     O53553|YZ08_MYCTU|RV3508|MTV023.15 (1901 aa), FASTA scores: opt: 2380, E(): 3.6e-87,
                     (53.8% identity in 773 aa overlap); and MTV023_21, MTV023_18,MTV023_14, MTV039_16,
                     MTCY441_4. Predicted to be an outer membrane protein (See Song et al., 2008)."
                     /codon_start=1
                     /transl_table=11
                     /product="PE-PGRS family protein PE_PGRS52"
                     /protein_id="YP_177968.1"
                     /db_xref="GI:57117101 GeneID:888151"
                     /translation="MSFVIANPEMLAAAATDLAGIRSAISAATAAAAAPTIQVAAAGADEVSLAISALFGQHAQAYQALSAQATI
                     FHDQFVQALTSGGNLYAAAESHTVEQMVLNAINAPTQTLFGRPLIGDGANGTAENPDGQNGGLLFGNGGNGFTQTTAGVAGGNGG
                     SAGLIGNGGAGGGGGAGAAGGLGGNGGWLYGNGGAGGIGGAGTGTGGHGGAGGAGGRAWLWGTGGAGGAGGDGGWLFGDGGAGGT
                     GGNGGSGFNSLTSSVGGAGGAGGHAGLFGAGGTGGTGGIGGQNTETGPAASNGGAGGAGGGGGYLVGDGGAGGTGGAGGKNSSGG
                     ATLTGGTGGTGGAGGAAGWLYGSGGAGGAGGAGGLNNAGGATGGTGGTGGAGGSGAWLYGNGGAAGAGGNGGNNTSAGTGGVGAS
                     GGTGGNAGLIGAGGHGGAGGAGGNQTGGVGNGGAGGNGGAGGAGGQLYGNGGDGGNGGAGGANIAGGNGSDGGAAGHGGAGGSAR
                     LIGAGGHGGDGGAGGNTAGRRADAIAGTGGDGGNGGNGGLLSGNAGAGGHGGAGGSSTATTTGTPPTGATGGNGGNGGAGGTAG
                     FTGSGGIGGNGGAGGTGGNAGVALSVGSTGGLGGNGGSGGLGGGGGSLFGNGGAGGVGATGGNGGSGIGPASVGGNGGKGGVGAA
                     GGLAGQIGNGGSGGSGGAGGNGGTGDTAGNGGNGGAGAVGGNAQLIGNGGNGGGGGNGGTGADGT"
     CDS             3715886..3720031
                     /gene="PE_PGRS53"
                     /locus_tag="Rv3507"
                     /experiment="EXISTENCE: identified in proteomics study"
                     /inference="protein motif:PROSITE:PS00583"
                     /note="Rv3507, (MTV023.14), len: 1381 aa. PE_PGRS53, Member of the Mycobacterium
                     tuberculosis PE protein family, PGRS subfamily of gly-rich proteins (see citation
                     below),similar to others from Mycobacterium tuberculosis strains H37Rv and CDC1551
                     e.g. O06810|Rv1450c|MTCY493.04 (1329 aa),FASTA scores: opt: 2173, E(): 1.4e-135,
                     (51.15% identity in 1412 aa overlap). Equivalent to AAK47970 from Mycobacterium
                     tuberculosis strain CDC1551 (1384 aa) but with some minor differences between the
                     proteins. Contains two PS00583 pfkB family of carbohydrate kinases signatures 1."
                     /codon_start=1
                     /transl_table=11
                     /product="PE-PGRS family protein PE_PGRS53"
                     /protein_id="YP_177978.1"
                     /db_xref="GI:57117114 GeneID:888256"
                     /translation="MSFVLVSPETVAAVATDLKRIGASLAHENASAAASTTAVVSAAADEVSTAVAALFSQHAQGYQAAAAQVAA
                     FHSRFVQALTAGAGAYAFAEAANASPLQSAMGAVSASAQTLLSRPLIGNGANATTPGGNGGDGGWLFGSGGNGAPGAAGQSGGNG
                     GSAGLWGNGGAGGAGGSGGAAGGNGGNGGWLFGAGGTGGIGGTGAPGAMGGTGGNGGNGALLIGGGGLGGAGGMGGTGGGTGGTG
                     GNGGNGALLIGAGGVGGAGGIGGQGTGAGGAAGAGGTGGNGGAGGLFMNGGDGGAGGQGGDGAAGDAAASAGGTGGKGGQGGDGG
                     TGGAGGAGPVLFGHGGAGGMGGQGGTGGMGGAGGDGTTVIAAGTGGEGGTGGAAGAGGAAGARGALTSGGLAGGVGAGGTGGTGG
                     TGGNGADAAAVVGFGANGDPGFAGGKGGNGGIGGAAVTGGVAGDGGTGGKGGTGGAGGAGNDAGSTGNPGGKGGDGGIGGAGGAG
                     GAAGTGNGGHAGNTGDGGDGGTGGNGGNGTGGVNGADNTLNPDTPGGAGEPGGAGGAGGAGGAAGGPGGTGGTGGNGGNGGNGGN
                     GGNGGNGGNGGNAGNNSTNAPVGGEGGAGGDGGAGGAGGAANGGTAGSQGTGGVGGDGGAGGNGGGGKAGTGNSGNFGVDGEAGF
                     SGGAGGNGGVGGAAGANGGTGGSGGNGGDGGAGGIGGAGGNGIPGTGTEPAGGTGAKGGDGGDGGAGGAGGNAGGAGGQGGNAGQ
                     GGAGGAGGNAVIPGDGVGKAPHGDAGGSGGDGGKGGQGGSGGTGGSGAPIGGGAGGTGGSGGHAGKGGAGGIGAQGTTITVPGNG
                     GNAGDGGNGGNAGAGGNGGSGDFGGNTTSGASGSGGNGGNAGTAGSGGAGGTGGTGLSGGNGGNGGNGGNGGDGGNGAHGTVGAQ
                     FVPATSLPTPNGGAGGNGGTGSNGGAPGPAGAPGPTTGGNAGSQGIGGDGGNGGDGGKGGDGADAVNVVFMPTEPQAATGTAGSA
                     GDPTGGNGGPGTPGSPMVAPPPPTPITQVQQGGDGGAGGTGSTNANDGTATGGKGGEGGVGSILGGPGGNGGTGGNASATGTNGV
                     ANAGNGGKGGDGGQFGAGGNGGAGGSVTDGSAGSTAGNGGNGGNATNGTIAGQPAGGNGSAGGKGGDGGNIAAGATGTAGNGGNG
                     GNGNDGAVNAGTGGSGGNGGNAGGGGANGGDGGAGGAGGAGGRGGKGIDGGFGGDGGNGGSNNGTGAGGNGGNGGTGGVGSVGAA
                     GGDGGNGGTGGFAGFGGTAGNGGSGGTGGAGGDGGTGGDGGNGVIAGGGGTGGNGGASGAGGAGGTGGFAGNGNAGGNGGTGGAS
                     EDGDNGNAGSGATGGTGGNGGTGGDGGAAGLGGVA"
     CDS             3720322..3726027
                     /gene="PE_PGRS54"
```

```
                     /locus_tag="Rv3508"
                     /experiment="EXISTENCE: identified in proteomics study"
                     /inference="protein motif:PROSITE:PS00583"
                     /note="Rv3508, (MTV023.15), len: 1901 aa. PE_PGRS54, Member of the Mycobacterium
                     tuberculosis PE family, PGRS subfamily of gly-rich proteins (see Brennan & Delogu
                     2002), similar to others from Mycobacterium tuberculosis strains H37Rv and CDC1551
                     e.g. downstream O53559|Rv3514|MTV023.21 (1489 aa),FASTA scores: opt: 6598, E(): 0,
                     (71.05% identity in 1533 aa overlap). Equivalent to AAK47971 from Mycobacterium
                     tuberculosis strain CDC1551 (1384 aa) but shorter 13 aa and with some minor
                     differences between the proteins. Contains five PS00583 pfkB family of carbohydrate
                     kinases signatures 1."
                     /codon_start=1
                     /transl_table=11
                     /product="PE-PGRS family protein PE_PGRS54"
                     /protein_id="YP_177979.1"
                     /db_xref="GI:57117115 GeneID:888270"
                     /translation="MSFVLIAPEFVTAAAGDLTNLGSSISAANASAASATTQVLAAGADEVSARIAALFGGFGLEYQAISAQVAA
                     YHQRFVQALSTGAGAYASAEAAAAEQIVLGVINAPTQALLGRPLIGDGANATTPGGAGGAGGLLFGNGGAGAAGAPGQAGGPGGP
                     AGLWGNGGPGGAGGSGGGTGGAGGAGGWLFGVGGAGGVGGAGGGTGGAGGPGGLIWGGGGAGGVGGAGGGTGGAGGRAELLFGAG
                     GAGGAGTDGGPGATGGTGGHGGVGGDGGWLAPGGAGGAGGQGGAGGAGSDGGALGGTGGTGGTGGAGGAGGRGALLLGAGGQGGL
                     GGAGGQGGTGGAGGDGVLGGVGGTGGKGGVGGVAGLGGAGGAAGQLFSAGGAAGAVGVGGTGGQGGAGGAGAAGADAPASTGLTG
                     GTGFAGGAGGVGGQGGNAIAGGINGSGGAGGTGGQGGAGGMGGSGADNASGIGADGGAGGTGGNAGAGGAGGAAGTGGTGGVVGA
                     AGKAGIGGTGGQGGAGGAGSAGTDATATGATGGTGFSGGAGGAGGAGGNTGVGGTNGSGGQGGTGGAGGAGGAGGVGADNPTGIG
                     GTGGTGGKGGAGGAGGQGGSSGAGGTNGSGGAGGTGGQGGAGGAGGAGADNPTGIGGAGGTGGTGGAAGAGGAGGAIGTGGTGGA
                     VGSVGNAGIGGTGGTGGVGGAGGAGAAAAAGSSATGGAGFAGGAGGEGGAGGNSGVGGTNGSGGAGGAGGKGGTGGAGGSGADNP
                     TGAGFAGGAGGTGGAAGAGGAGGATGTGGTGGVVGATGSAGIGGAGGRGGDGGDGASGLGLGLSGFDGGQGGQGGAGGSAGAGGI
                     NGAGGAGGNGGDGGDGATGAAGLGDNGGVGGDGGAGGAAGNGGNAGVGLTAKAGDGGAAGNGGNGGAGGAGGAGDNNFNGGQGGA
                     GGQGGQGGLGGASTTSINANGGAGGNGGTGGKGGAGGAGTLGVGGSGGTGGDGGDAGSGGGGGFGGAAGKAGGGGNGGRGGDGGD
                     GASGLGLGLSGFDGGQGGQGGAGGSAGAGGINGAGGAGGNGGDGGDGATGAAGLGDNGGVGGDGGAGGAAGNGGNAGVGLTAKAG
                     DGGAAGNGGNGGAGGAGGAGDNNFNGGQGGAGGQGGQGGLGGASTTSINANGGAGGNGGTGGKGGAGGAGTLGVGGSGGTGGDGG
                     DAGSGGGGGFGGAAGKAGGGGNGGVGGDGGEGASGLGLGLSGFDGGQGGQGGAGGSAGAGGINGAGGAGGTGGAGGDGAPATLIG
                     GPDGGDGGQGGIGGDGGNAGFGAGVPGDGGDGGNAGFGAGVPGDGGIGGTGGAGGAGGAGADGDPSIDGGQGGAGGHGGQGGKGG
                     LNSTGLASAASGDGGNGGAGGAGGNGGDGDGFIGGSGGTGGTGGDAGVGGLANTGGTAGNAGIGGAGGRGGDGGAGDSGALSQDG
                     NGFAGGQGGQGGVGGNAGAGGINGAGGTGGTGGAGGDGQNGTTGVASEGGAGGQGGDGGQGGIGGAGGNAGFGAGVPGDGGIGGT
                     GGAGGAGGAGADGDPSIDGGQGGAGGHGGQGGKGGLNSTGLASAASGDGGNGGAGGAGGNGGDGDGFIGGSGGTGGTGGDAGVGG
                     LANTGGTAGNAGIGGAGGRGGDGGAGDSGALSQDGNGFAGGQGGQGGVGGNAGAGGINGAGGTGGTGGAGGDGQNGTTGVASEGG
                     AGGQGGDGGQGGIGGAGGNAGFGAGVPGDGGIGGTGGAGGAGGAGADGDPSIDGGQGGAGGHGGQGGKGGLNSTGLASAASGDGG
                     NGGAGGAGGNGGAGGLGGGGTGGTNGNGGLGGGGNGGAGGAGGTPTGSGTEGTGGDGGDAGAGGNGGSATGVGNGGNGGDGGN
                     GGDGGNGAPGGFGGGAGAGGLGGSGAGGGTDGDDGNGGSPGTDGS"
     CDS             3728934..3731078
                     /gene="PE_PGRS55"
                     /locus_tag="Rv3511"
                     /inference="protein motif:PROSITE:PS00583"
                     /note="Rv3511, (MTV023.18), len: 714 aa. PE_PGRS55, Member of the Mycobacterium
                     tuberculosis PE family, PGRS subfamily of gly-rich proteins (see Brennan and Delogu,
                     2002),similar to others from Mycobacterium tuberculosis strains H37Rv and CDC1551
                     e.g. AAK47974|MT3615.3 (1217 aa) FASTA scores: opt: 2563, E(): 1.5e-94, (59.65%
                     identity in 773 aa overlap); and upstream O53553|Rv3508|MTV023.15 (1901 aa),FASTA
                     scores: opt: 2455, E(): 3.9e-90, (60.4% identity in 737 aa overlap); etc. Contains
                     PS00583 pfkB family of carbohydrate kinases signature 1."
                     /codon_start=1
                     /transl_table=11
                     /product="PE-PGRS family protein PE_PGRS55"
                     /protein_id="YP_177980.1"
                     /db_xref="GI:57117116 GeneID:888273"
                     /translation="MSFVLISPEVVSAAAGDLANVGSTISAANKAAAAATTQVLAAGADEVSARIAALFGMYGLEYQAISAQVAA
                     YHQQFVQTLRTGAASYMLAEATNVEQNLLNLINAPTQTLLGRPLIGDGANATTPGGAGGDGGLLFGSGGNGAPGAPGQAGGAGGS
                     AGLLGNGGSGGAGGTGAPGGNGGNAGWLYGRGGVGGAGGIGGGTGGAGGHAWLFGHGGTGGIGGGPGGNGGWLLGNGGHGGAGGI
                     GGGSGGAGGNGGWLLGNGGIGGAGGTGGGAGGTGGNAAWLLGGGGTGGAGGIGGGNGGHGGNGGWLLGNGGNGGLGGDGDGGTGG
                     GHGGNGGNPGWLLGTAGGGGNGGAGSTGTAGGGSGGTGGDGGTGGRGGLLMGAGAGGHGGTGGAGGAGVNGGGAGGAGGAGGNGG
                     AGGQAALLFGRGGTGGAGGYGGDGGGGGDGFDGTMAGLGGTGGSGGTGGDGGAPGNGGAGGAGQLLSHSGVAGASGKGGAGGTGG
                     NGGAGSAGADAPAGSGAMGSTGFAGGAGGDGGNGGGSGASQGNGGNGGNGGTGGKGGTGGAGMNSLDPLLAAQDGGQGGTGGTGG
                     NAGAGGTGFTQGADGNAGNGGDGGVGGNGGNGADNTTTAAAGTTGGAGGAGGAGGTGGAAGTGTGGQQGNGGNGGNGGTGGKGGT
                     GGAGMNSLDPLLAAQDGGQGGTGGTGGNAGAGGTGFTPRRRRQRRQRR"
     CDS             3731041..3734280
                     /gene="PE_PGRS56"
                     /locus_tag="Rv3512"
                     /experiment="EXISTENCE: identified in proteomics study"
                     /note="Rv3512, (MTV023.19), len: 1079 aa. PE_PGRS56, Member of the Mycobacterium
```

```
                     tuberculosis PE family, PGRS subfamily of gly-rich proteins (see citation below),
                     similar to others from Mycobacterium tuberculosis strains H37Rv and CDC1551 e.g.
                     AAK47974|MT3615.3 (1217 aa) FASTA scores: opt: 3688, E(): 4.5e-130, (53.95% identity
                     in 1136 aa overlap); and downstream O53559|Rv3514|MTV023.21 (1489 aa), FASTA scores:
                     opt: 3611, E(): 3.6e-127, (53.15% identity in 1195 aa overlap); etc. Frameshifted
                     PGRS protein, could be continuation of upstream MTV023.18, but no error could be
                     found."
                     /codon_start=1
                     /transl_table=11
                     /product="PE-PGRS family protein PE_PGRS56"
                     /protein_id="YP_177981.1"
                     /db_xref="GI:57117117 GeneID:888306"
                     /translation="PQGADGNAGNGGDGGVGGNGGNGADNTTTAAAGTTGGAGGAGGAGGTGGTGGAAGTGTGGQQGNGGNGGNG
                     GTGGKGGTGGDGALAGSSGGAGGKGGNGGDAGKAGTGSAPGTAGTGGDGGKGGNGGIGAAGTTGPVGTGASGGTGGSGGAGGTGG
                     DGGAANGGTAGAGGAGGNGGKGGDGGAGVTSSTAGNSGGAGGSGGKGGDAGAGGAGATPGANGIAGNGGDGGDGAAGAVGISGAT
                     GAGDGGHGGTGAAGGNGGTGGAGGSGIDGVGGGTGGTGGNGGNGAIGGAGGDAGGSGNSGGNGGIGGKGGNAGAGGAAGSNGGTV
                     GANGTGGDGGNGGAAGAATAGSNGGAGTGSAGGNGGTGGRGGSGGAGGDGIGGVGGGKGGNGADGEVGGAGGAGGSGPNTSPGGN
                     GGQGGQGGSGGAGGAAGAGGAGGGANGTAGNGGQGGAGGTGGAGAASSATNGGSGGAGGTGGDGGSGGAGGTGGAGGTGGAAGDG
                     GQGGQGGAGGGAGGQGGAGGAGGTGGNGGNITGGTAGTAGAAGNGGAAGKGGAGGQGGTGGGTGGQGGAGGDGGAGGTGGDRTVG
                     GGTVPAGSGGQGGNAGGGGAGGQGGADGGSGGDGGDAGTGGNGGNGGNRNSGNGTGGAGGNGGGANGGAGGAGGSGGGTGGNGG
                     AGGDAGDAGNGGNGNGTGNGGNGGNGGIAGMGGNGGAGTGSGNGGNGGSGGNGGNAGMGGNSGTGSGDGGAGGNGGAAGTGGTGG
                     DGGLTGTGGTGGSGGTGGDGGNGGNGADNTANMTAQAGGDGGNGGDGGFGGGAGAGGGGLTAGANGTGGQGGAGGDGGNGAIGGH
                     GPLTDDPGGNGGTGGNGGTGGTGGAGIGSLGGGTGGDGGNGGNGGTGGEGGEVGGAGGTGGAAGNGGDGGTGGTGGGDGGAGGTG
                     GTGGTGGLGDPRVGGSGGDGGTGGSGGAAGNGGNGGNAGAGGNGNGGTGGAGGIGGTGGNGGDAEPGVPPGAGGAGGAGTTGGKG
                     GTGGNGSGTGSGGTGGDGGTGGGGGNGGTGWNGGKGDTGSGGGAGDGGKAPAGGTGGAGGDGGAGGKGGSGGV"
     CDS             3735111..3739580
                     /gene="PE_PGRS57"
                     /locus_tag="Rv3514"
                     /experiment="EXISTENCE: identified in proteomics study"
                     /inference="protein motif:PROSITE:PS00583"
                     /note="Rv3514, (MTV023.21), len: 1489 aa. PE_PGRS57, Member of the Mycobacterium
                     tuberculosis PE family, PGRS subfamily of gly-rich proteins (see citation below),
                     similar to others from Mycobacterium tuberculosis strains H37Rv and CDC1551 e.g.
                     AAK47971 (1715 aa) FASTA scores: opt: 6940,E(): 0, (67.0% identity in 1713 aa
                     overlap); and upstream O53553|YZ08_MYCTU|Rv3508|MTV023.15 (1901 aa), FASTA scores:
                     opt: 6598,E(): 0, (71.05% identity in 1533 aa overlap). Contains two PS00583 pfkB
                     family of carbohydrate kinases signatures 1."
                     /codon_start=1
                     /transl_table=11
                     /product="PE-PGRS family protein PE_PGRS57"
                     /protein_id="YP_177982.1"
                     /db_xref="GI:57117118 GeneID:888294"
                     /translation="MSFVLIAPEFVTAAAGDLTNLGSSISAANASAASATTQVLAAGADEVSARIAALFGGFGLEYQAISAQVAA
                     YHQRFVQALSTGAGAYASAEAAAAEQIVLGVINAPTQALLGRPLIGDGANATTPGGAGGAGGLLFGNGGAGAAGAPGQAGGPGGP
                     AGLWGNGGPGGAGGSGGGTGGAGGAGGWLFGVGGAGGVGGAGGGTGGAGGPGGLIWGGGGAGGVGGAGGGTGGAGGRAELLFGAG
                     GAGGAGTDGGPGATGGTGGHGGVGGDGGWLAPGGAGGAGGQGGAGGAGSDGGALGGTGGTGGTGGAGGAGGRGALLLGAGGQGGL
                     GGAGGQGGTGGAGGDGVLGGVGGTGGKGGVGGVAGLGGAGGAAGQLFSASGAAGNAGVGGAGGQGGDGGAGGAGADADQPGATGG
                     TGFAGGAGGAGGAGGSSGAGGTNGSGGAGGQGGAGGAGGAGADNPTGIGGTGGDGGTGGAAGAGGAGGAAGTGGTGGMIGTTGNA
                     GVGGAGGQGGDGGAGGAGADADQPGATGGTGFAGGAGGAGGAGGSSGAGGTNGSGGAGGTGGQGGAGGAGGAGADNPTGIGGTGG
                     DGGTGGAAGAGGAGGAAGTGGTGGMIGTTGNAGVGGAGGQGGDGGAGGAGADADQPGATGGTGFAGGAGGAGKAGGSSSAGGTNS
                     SGSAGGTGRQSGTGGAGGAGADNPTGIGGTGGDGGTGGAAGAGGAGGAAGTGGTGGMIGTTGNAGVGGAGGSSGAGGTNGSGGAG
                     GTDGQGGAGGAGGAGADNPTGIGGTGGDGGTGGAAGAGGAGGAAGTGGTGGMIGTTGNAGVGGAGGQGGDGGAGGAGADADQPGA
                     TGGTGFAGGAGGAGGSGGSSCAGGTNGSGGAGGTCGQVVAGGAGISFSNGSNGGTGGTGGVGGTGGDGGNAGTGAGDPGKGGTGG
                     TGGTGGSGGAGGSGGANFNGGTGGTGGTGGKGGLNTDGLSSATSGTGGTGGTGGKGGTGGAGDDSAGGTGGTGGAGGNAGAGGLA
                     NTGGTAGNAGIGGDGGQGGNGGQGDSGSGLGGQPGFAGGAGGKGGAGGSSGAGGTNGSGGAGGAGGQGGAGGAGISFSNGSNGGT
                     GGTGGVGGTGGDGGNAGTGAGDPGKGGTGGTGGTGGSGGAGGSGGANFNGGTGGTGGTGGTGGKGGMGGIAGDGGPGGDGGNAGV
                     GGKGGTNGNGGSGGTGGTGGAGGNAGAGGLANTGGTAGNAGIGGDGGQGGNGGQGDSGSGLGGQPGFAGGPGGKGGAGGNAGTGG
                     TNGSGAGGAGGQGGAGGAGISFSNGSNGGTGGTGGVGGTGGDGGNAGTGAGDPGKGGTGGTGGTGGSGGAGGSGGANFNGGTGGT
                     GGTGGTGGKGGMGGIAGDGGPGGDGGNAGVGGKGGTNGNGGSGGTGGTGGPGGSGGAPTGSGTGGKGGAGGDGGDGADGGAATGV
                     GDGGDGGNGGNGGNGGTGVGSPGGLGGAGGTGGLGGAGAGGGADGDDGDDGQPGNNGS"
     CDS             complement(3814652..3816406)
                     /gene="PE_PGRS58"
                     /locus_tag="Rv3590c"
                     /experiment="EXISTENCE: identified in proteomics study"
                     /note="Rv3590c, (MTV024.08c, MTCY6F7.04), len: 584 aa. PE_PGRS58, Member of the
                     Mycobacterium tuberculosis PE family, PGRS subfamily of gly-rich proteins (see
                     citation below), highly similar to e.g. O53439|Rv1091|MTV017.44 (853 aa), FASTA
                     scores: opt: 2005, E(): 1.4e-70, (54.95% identity in 646 aa overlap)."
                     /codon_start=1
```

```
                     /transl_table=11
                     /product="PE-PGRS family protein PE_PGRS58"
                     /protein_id="YP_177993.1"
                     /db_xref="GI:57117129 GeneID:887874"
                     /translation="MSFVIVAPEALMSVASEVAGIGSALNAANAAAAAPTTGVLAAAADEVSAAMAALFGAHAQEYQRLSAQAAG
                     FHAQFVQALNAGVNSYASAEAANASPLQAVEQQVLGLINGPAQTLLGRPLIGNGADGAPGTGQPGGPGGLLWGNGGNGGSGVAGV
                     GGPGGSGGAAGLFGHGGNGGAGGSNAAGAGGVGGAGGAGWLVGNGGAGGFGGVGTTVSGNGGAGGAAGAFGNGGVGGAGGAAVIG
                     GLPGNGGAGGNAGLIGAGGDGGVGGVGAPGTNGMNPPPNQTSQAANGSPGANNGAGSGGAGLPGNPGAVPGRAGGAGGLGGSGSD
                     TSEGPVTGGNGGNGGDGGPGAPGGNGAPGGIGVNTGTGWAYGGNGGNGGDGGAGARGGDGGNGGNGLALNGGNGIGGNGGAGGRG
                     GTGAAGGNGGIGGGATGTLTFFGSGGDGGPGGAGANTAGTGGVGGVGGAGGQGGLLFGDGGNGGAGGAGGIGGTGASGGAGGKGG
                     SGLVGGDGGNGGAGGAGGNGGKGGAGGAGGGAGMFSQPGVHGAGGTGGQGGAGGAGGAGGAAGAGTVVAGNPGDPGGFGAAGADG
                     LPG"
     CDS             complement(3819979..3821298)
                     /gene="PE_PGRS59"
                     /locus_tag="Rv3595c"
                     /experiment="EXISTENCE: identified in proteomics study"
                     /note="Rv3595c, (MTCY07H7B.27), len: 439 aa. PE_PGRS59,Member of the Mycobacterium
                     tuberculosis PE family, PGRS subfamily of gly-rich proteins (see citation
                     below),similar to many e.g. O53439|Rv1091|MTV017.44 (853 aa),FASTA scores: opt: 1644,
                     E(): 1.2e-57, (58.75% identity in 492 aa overlap)."
                     /codon_start=1
                     /transl_table=11
                     /product="PE-PGRS family protein PE_PGRS59"
                     /protein_id="YP_177994.1"
                     /db_xref="GI:57117130 GeneID:885464"
                     /translation="MSFVIAVPEFLSAAATDLANLGSTISAANAAASIPTTGVLAAGADDVSAAIAALFGAHAQAYQTISAQAAT
                     FHAQFVQTLSAGAGAYANAEAANVQQSLLNAINAPTQALLGRPLIGDGADGTAPGQNGGAGGLLYGNGGNGAAGVNAGIAGGSGG
                     AAGLIGNGGSGGAGGAGAAGGSGGQGGLLYGNGGAGGNGGAATIPGGNGGAGGAGGNAWLFGNGGAGGLGAAGAAGAAGVNPLTV
                     PAGQGSMGNNGEPGGPGQPGTEFGQTGGTGGTGGTGLSVGGTGGTGGTGGTGGAGGSGGRGGLLVGDGGAGGIGGTGGEGGIGAR
                     GGTGGQGGMGGAGQPGVGGDAGDGGNGGIGGDGGAGGDGGAGGAGGAGGLFGVSGSSGLGGAAGSGGNGGGGGEPGVAGSPGVGP
                     AGRGGDGNLGQFGPEGAPGQPGQPGQPG"
     CDS             3875638..3875952
                     /gene="PE_PGRS60"
                     /locus_tag="Rv3652"
                     /note="Rv3652, (MTV025.001A), len: 104 aa. PE_PGRS60,Member of the Mycobacterium
                     tuberculosis PE family, PGRS subfamily of gly-rich proteins (see Brennan and
                     Delogu,2002), similar at N-terminal end with many e.g.
                     P56877|Y278_MYCTU|Rv0278c|MTV035.06c (957 aa) FASTA scores: opt: 242, E(): 3e-09,
                     (77.35% identity in 53 aa overlap). Originally annotated as the first part of a
                     PE-PGRS family protein (Rv3653/PE_PGRS61 being the second part) but more similar to a
                     PE family protein. Length extended since first submission (+50 aa)."
                     /codon_start=1
                     /transl_table=11
                     /product="PE-PGRS family-related protein PE_PGRS60"
                     /protein_id="YP_178001.1"
                     /db_xref="GI:57117139 GeneID:886260"
                     /translation="MSYVIAAPEALVAAATDLATLGSTIGAANAAAAGSTTALLTAGADEVSAAIAAYSECTARPIRHSVRGRRR
                     SMSGSCRPWPQVGAPMRPPRPPASRRCRARSIC"
     CDS             3875946..3876533
                     /gene="PE_PGRS61"
                     /locus_tag="Rv3653"
                     /note="Rv3653, (MTV025.001B), len: 195 aa. PE_PGRS61,Member of the Mycobacterium
                     tuberculosis PE family, PGRS subfamily of gly-rich proteins (see Brennan and
                     Delogu,2002), highly similar to the C-termini of members of the Mycobacterium
                     tuberculosis PE family, PGRS subfamily of gly-rich proteins, e.g. MTCY1A11_25,
                     MTCY28_25, MTCY130_10,MTCY1A10_19, MTCY21B4_13, MTCI418B_6,MTCY28_34,
                     MTV004_1,MTCY441_4; etc. Originally annotated as the second part of a PE-PGRS family
                     protein (Rv3652/PE_PGRS60 being the first part). Start shortened since first
                     submission (-50 aa)."
                     /codon_start=1
                     /transl_table=11
                     /product="PE-PGRS family-related protein PE_PGRS61"
                     /protein_id="YP_178002.1"
                     /db_xref="GI:57117140 GeneID:886259"
                     /translation="MLNAPTQALLGRPLVGNGANGAPGTGANGGDGGILFGSGGAGGSGAAGMAGGNGGAAGLFGNGGAGGAGGS
                     ATAGAAGAGGNGGAGGLLFGTAGAGGNGGLSLGLGVAGGAGGAGGSGGSDTAGHGGTGGAGGLLFGAGEDGTTPGGNGGAGGVAG
                     LFGDGGNGGNAGVGTPAGNVGAGGTGGLLLGQDGMTGLT"
     CDS             4057395..4058909
                     /gene="PE_PGRS62"
```

```
/locus_tag="Rv3812"
/experiment="EXISTENCE: identified in proteomics study"
/note="Rv3812, (MTV026.17, MTCY409.18c), len: 504 aa. PE_PGRS62, Member of the
Mycobacterium tuberculosis PE family, PGRS subfamily of gly-rich proteins (see
citations below), similar to many e.g. P96828|Rv0151c|MTCI5.25c (588 aa), FASTA
scores: opt: 389, E(): 6.2e-14, (29.2% identity in 473 aa overlap); MTCY7H7B_27;
MTCY493_24; MTCY441_4; MTCY39_36; MTCY1A11_4; MTCY359_33; MTCY130_10; MTCY98_9; etc.
The transcription of this CDS seems to be activated in macrophages (see Ramakrishnan
et al., 2000)."
/codon_start=1
/transl_table=11
/product="PE-PGRS family protein PE_PGRS62"
/protein_id="YP_178019.1"
/db_xref="GI:57117161 GeneID:886143"
/translation="MSFVVTVPEAVAAAAGDLAAIGSTLREATAAAAGPTTGLAAAAADDVSIAVSQLFGRYGQEFQTVSNQLAA
FHTEFVRTLNRGAAAYLNTESANGGQLFGQIEAGQRAVSAAAAAAPGGAYGQLVANTATNLESLYGAWSANPFPFLRQIIANQQV
YWQQIAAALANAVQNFPALVANLPAAIDAAVQQFLAFNAAYYIQQIISSQIGFAQLFATTVGQGVTSVIAGWPNLAAELQLAFQQ
LLVGDYNAAVANLGKAMTNLLVTGFDTSDVTIGTMGTTISVTAKPKLLGPLGDLFTIMTIPAQEAQYFTNLMPPSILRDMSQNFT
NVLTTLSNPNIQAVASFDIATTAGTLSTFFGVPLVLTYATLGAPFASLNAIATSAETIEQALLAGNYLGAVGALIDAPAHALDGF
LNSATVLDTPILVPTGLPSPLPPTVGITLHLPFDGILVPPHPVTATISFPGAPVPIPGFPTTVTVFGTPFMGMAPLLINYIPQQL
ALAIKPAA"
```

```
     gene            complement(2217083..2218387)
                     /gene="PE_PGRS36"
                     /locus_tag="Rv2098c"
                     /pseudogene="unknown"
                     /db_xref="GeneID:888312"
     misc_feature    complement(2217083..2218387)
                     /gene="PE_PGRS36"
                     /locus_tag="Rv2098c"
                     /note="Rv2098c, (MTCY49.38c), len: 434 aa. PE_PGRS36,Member of the Mycobacterium
                     tuberculosis PE family, PGRS subfamily of gly-rich proteins (see citation below).
                     Frameshifted near N-terminus (see Rv2099c|PE21)."
                     /pseudogene="unknown"
     misc_feature    complement(2218387..2218560)
                     /gene="PE21"
                     /locus_tag="Rv2099c"
                     /note="Rv2099c, (MTCY49.39c), len: 58 aa. PE21, Member of the Mycobacterium
                     tuberculosis PE family (see Brennan and Delogu, 2002); 5'-end of
                     Rv2098c|PE_PGRS36|MTCY49.38c, then frameshifts. Sequence has been checked, no errors
                     found."
                     /pseudogene="unknown"
```

```
     CDS             complement(173205..174782)
                     /gene="PE2"
                     /locus_tag="Rv0152c"
                     /experiment="EXISTENCE: identified in proteomics study"
                     /note="Rv0152c, (MTCI5.26c), len: 525 aa. PE2, Member of the Mycobacterium
                     tuberculosis PE family (see citation below), similar to ORF downstream
                     Z92770|MTCI5_25 (588 aa),FASTA scores: opt: 1492, E(): 0, (54.7% identity in 464 aa
                     overlap); and to many other PE family type members. Predicted to be an outer membrane
                     protein (See Song et al.,2008)."
                     /codon_start=1
                     /transl_table=11
                     /product="PE family protein PE2"
                     /protein_id="YP_177696.1"
                     /db_xref="GI:57116695 GeneID:886838"
                     /translation="MRCRPPSRNRSAHTARNTRPCSLKSRRFTVRFHQTLAAAANSYADAEAAIASTRQNQLAVPAAAPTPAAAA
                     MIPPFPANLTTLFFGPTGIPLPPPSMLTPPIRCRSVRRALQAVFTPEELYPLTGVRSLVLNTSVEEGLTILHDAIMVELATTGNA
                     VTVFGWSQSAIIASLEMQRFTAMGGAAPSASDLNFVLVGNEMNPNGGMLARFPDLTLPTLDLTFYGATPSDTIYPTAIYTLEYDG
                     FADFSRYPLNFISDLNAVAGITFVHTKYLDLTPAQVEGATKLPTSPGYTGVTDYYIIRTENRPLLQPLRAVPVIGDPLADLIQPN
                     LKVIVNLGYGDPNYGYSTSYADVRTPFGLWPNVPPQVIADALAAGTQEGILDFTADLQALSAQPLTLPQIQLPQPADLVAAVAAA
                     PTPAEVVNTLARIISTNYAVLLPTVDIALALVTTLPLYTTQLFVRQLAAGNLINAIGYPLAATVGLGTIDSGRRGIAHPPRGGLG
                     HRSKHRGPRHLTDSRRHRRPPTTVYRPRQ"
     CDS             complement(181319..182725)
                     /gene="PE3"
                     /locus_tag="Rv0159c"
                     /note="Rv0159c, (MTV032.02c), len: 468 aa. PE3, Member of the Mycobacterium
                     tuberculosis PE family (see citation below), similar to many other PE proteins e.g.
                     O06828 from Mycobacterium tuberculosis (528 aa), FASTA scores: opt: 1163, E(): 0,
                     (45.8% identity in 467 aa overlap). Also highly similar to upstream MTV032_3, and to
                     MTCI5_25,MTCI5_26, MTV049_ 21, MTCY1A10_26, etc."
                     /codon_start=1
                     /transl_table=11
                     /product="PE family protein PE3"
                     /protein_id="YP_177697.1"
                     /db_xref="GI:57116696 GeneID:886826"
                     /translation="MSYVIAAPEMLATTAADVDGIGSAIRAASASAAGPTTGLLAAAADEVSSAAAALFSEYARECQEVLKQAAA
                     FHGEFTRALAAAGAAYAQAEASNTAAMSGTAGSSGALGSVGMLSGNPLTALMMGGTGEPILSDRVLAIIDSAYIRPIFGPNNPVA
                     QYTPEQWWPFIGNLSLDQSIAQGVTLLNNGINAELQNGHDVVVFGYSQSAAVATNEIRALMALPPGQAPDPSRLAFTLIGNINNP
                     NGGVLERYVGLYLPFLDMSFNGATPPDSPYQTYMYTGQYDGYAHNPQYPLNILSDLNAFMGIRWVHNAYPFTAAEVANAVPLPTS
                     PGYTGNTHYYMFLTQDLPLLQPIRAIPFVGTPIAELIQPDLRVLVDLGYGYGYADVPTPASLFAPINPIAVASALATGTVQGPQA
                     ALVSIGLLPQSALPNTYPYLPSANPGLMFNFGQSSVTELSVLSGALGSVARLIPPIA"
     CDS             complement(182817..184325)
                     /gene="PE4"
                     /locus_tag="Rv0160c"
                     /experiment="EXISTENCE: identified in proteomics study"
                     /note="Rv0160c, (MTV032.03c), len: 502 aa. PE4, Member of the Mycobacterium
                     tuberculosis PE family (see citation below), similar to many other PE proteins e.g.
                     Z92770|MTCI5_26c from Mycobacterium tuberculosis (525 aa),FASTA scores: opt: 816,
                     E(): 0, (41.4% identity in 367 aa overlap); C-terminal region of
                     O06801|RV1768|MTCY28.34 from Mycobacterium tuberculosis (618 aa), FASTA scores: opt:
                     417, E(): 6.7e-18, (53.5% identity in 142 aa overlap). Also highly similar to
                     downstream ORF MTV032_2."
                     /codon_start=1
                     /transl_table=11
                     /product="PE family protein PE4"
                     /protein_id="YP_177698.1"
                     /db_xref="GI:57116697 GeneID:886825"
                     /translation="MSHLVTAPDMLATAAAHVDEIASTLRAANAAAAGPTCNLLAAAGDEVSAATAALFSAYGREYQAVVKQAAA
                     FHSEFTRTLEAAGNAYAHAEAANAARVSHALDTINAPIRTLLGRAPLSPNGSSGAGGLPAIAQLAAESPITALIMGGTNNPLPDP
                     EYVTDINKAFIQTLFPGAVSQGLFTPEQFWPVTPDLGNLTFNQSVTEGVALLNTAVNNQLALDNKVVAFGYSQSATIINNYINSL
                     MAMGSPNPDDISFVMIGSGNNPVGGLLARFPGFYIPFLDVPFNGATPANSPYPTHIYTAQYDGIAHAPQFPLRILSDINAFMGYF
                     YVHNTYPELMATQVDNAVPLPTSPGYTGNTQYYMFLTQDLPLLQPIRDIPYAGPPIADLFQPQLRVLVDLGYADYGPGGNYADIP
                     TPAGLFSIPNPFAVTYYLIKGSLQAPYGAIVEIGVEAGLIGPEWFPDSYPWVPSINPGLNFYFGQPQVTLLSLMSGGLGNILHLI
                     PPPVFT"
     CDS             334840..335148
                     /gene="PE5"
                     /locus_tag="Rv0285"
                     /experiment="EXISTENCE: identified in proteomics study"
                     /note="Rv0285, (MTV035.13), len: 102 aa. PE5, Member of the Mycobacterium
                     tuberculosis PE family (see Brennan & Delogu 2002), similar to others e.g.
```

```
                     AL0212|MTV012_37 from Mycobacterium tuberculosis (105 aa), FASTA scores: opt: 497,
                     E(): 2.6e-24, (80.4% identity in 102 aa overlap); Z80108|MTCY21B4.03 from
                     Mycobacterium tuberculosis (102 aa), FASTA scores: opt: 413, E(): 3.7e-19, (66.7%
                     identity in 102 aa overlap); etc. A core mycobacterial gene; conserved in
                     mycobacterial strains (See Marmiesse et al.,2004)."
                     /codon_start=1
                     /transl_table=11
                     /product="PE family protein PE5"
                     /protein_id="YP_177710.1"
                     /db_xref="GI:57116715 GeneID:886608"
                     /translation="MTLRVVPEGLAAASAAVEALTARLAAAHASAAPVITAVVPPAADPVSLQTAAGFSAQGVEHAVVTAEGVEE
                     LGRAGVGVGESGASYLAGDAAAAATYGVVGG"
     CDS             complement(371926..372441)
                     /gene="PE6"
                     /locus_tag="Rv0335c"
                     /experiment="EXISTENCE: identified in proteomics study"
                     /note="Rv0335c, (MTCY279.02c), len: 171 aa. PE6, Member of the Mycobacterium
                     tuberculosis PE family (see Brennan & Delogu 2002); contains short region of
                     similarity to part of the unique N-terminus of the Mycobacterium tuberculosis PGRS
                     family of Glycine-rich proteins e.g. Y03A_MYCTU|Q10637 hypothetical glycine-rich 49.6
                     kd protein (603 aa), FASTA scores: opt: 219, E(): 1.1e-08, (51.5% identity in 66 aa
                     overlap)."
                     /codon_start=1
                     /transl_table=11
                     /product="PE family protein PE6"
                     /protein_id="YP_177717.1"
                     /db_xref="GI:57116722 GeneID:886527"
                     /translation="MRSMGFLHRACRAPSSLPAPLMARPGRSVLARPAATPPGPLCATTRPRPPQGNQPPASRISNFPPKRHKTR
                     VLAAAEDEVSAAVAALISAHGRRHHSLNNQAAAFHGQFAQNLNVGAGSCASAETTADAPTQALLGPADRQRRQRRAVRQWLVRWA
                     AHPGRATRGFHNHRQ"
     CDS             complement(955423..955722)
                     /gene="PE7"
                     /locus_tag="Rv0916c"
                     /gene_synonym="MTB10"
                     /note="Rv0916c, (MTCY21C12.10c), len: 99 aa. PE7 (alternate gene name: MTB10). Member
                     of the Mycobacterium tuberculosis PE family (see citations below), similar to many
                     e.g. Rv1788 from Mycobacterium tuberculosis (99 aa), FASTA scores: opt: 321, E():
                     1.3e-11, (53.5% identity in 99 aa overlap); etc."
                     /codon_start=1
                     /transl_table=11
                     /product="PE family protein PE7"
                     /protein_id="YP_177766.1"
                     /db_xref="GI:57116796 GeneID:885167"
                     /translation="MSFVTIQPVVLAAATGDLPTIGTAVSARNTAVCAPTTGVLPPAANDVSVLTAARFTAHTKHYRVVSKPAAL
                     VHGMFVALPAATADAYATTEAVNVVATG"
     CDS             complement(1090310..1091137)
                     /gene="PE8"
                     /locus_tag="Rv1040c"
                     /note="Rv1040c, (MTCY10G2.09), len: 275 aa. PE8, Member of the Mycobacterium
                     tuberculosis PE family (see citation below), most similar to AL008967|MTV002_34
                     Mycobacterium tuberculosis H37Rv (275 aa), FASTA scores: opt: 1111, E(): 0, (68.6%
                     identity in 283 aa overlap). This region is a possible MT-complex-specific genomic
                     island (See Becq et al., 2007)."
                     /codon_start=1
                     /transl_table=11
                     /product="PE family protein PE8"
                     /protein_id="YP_177779.1"
                     /db_xref="GI:57116814 GeneID:888533"
                     /translation="MSFLKTVPEELTAAAAQLGTIGAAMAAQNAAAAAPTTAIAPAALDEVSALQAALFTAYGTFYQQVSAEAQA
                     MHDMFVNTLGISAGTYGVTESLNSSAAASPLSGITGEASAIIQATTGLFPPELSGGIGNILNIGAGNWASATSTLIGLAGGGLLP
                     AEEAAEAASALGGEAALGELGALGAAEAALGEAGIAAGLGSASAIGMLSVPPAWAGQATLVSTTSTLPGAGWTAAAPQAAAGTFI
                     PGMPGVASAARNSAGFGAPRYGVKPIVMPKPATV"
     CDS             1136574..1137008
                     /gene="PE9"
                     /locus_tag="Rv1088"
                     /experiment="EXISTENCE: identified in proteomics study"
                     /note="Rv1088, (MTV017.41), len: 144 aa. PE9, Member of Mycobacterium tuberculosis PE
                     family (see citation below),similar to many others e.g. Z96071|MTCI418B_6
                     Mycobacterium tuberculosis cosmid (487 aa), FASTA scores: opt: 318, E(): 7.3e-14,
```

```
                     (60.9% identity in 87 aa overlap) - except it appears to be frameshifted around codon
                     84. No error to account for frameshift could be found."
                     /codon_start=1
                     /transl_table=11
                     /product="PE family protein PE9"
                     /protein_id="YP_177784.1"
                     /db_xref="GI:57116823 GeneID:887096"
                     /translation="MSYMIATPAALTAAATDIDGIGSAVSVANAAAVAATTGVLAAGGDEVLAAIARLFNANAEEYHALSAQVAA
                     FQTLFVRTLTGGCGVFRRRRGRQCVTAAEHRAAGAGRRQRRRRSGDGQWRLRQQRHFGCGGQPEFRQHSEHRR"
     CDS             1136830..1137192
                     /gene="PE10"
                     /locus_tag="Rv1089"
                     /note="Rv1089, (MTV017.42), len: 120 aa. PE10, Member of the Mycobacterium
                     tuberculosis PE family of glycine-rich proteins (see citation below). Partial ORF
                     that appears to be frameshifted continuation of Rv1088|MTV017.41. Sequence has been
                     checked and appears correct. Similar to Z95555|MTCY06F7_4 Mycobacterium tuberculosis
                     cosmid (401 aa), FASTA scores: opt:126, E(): 2, (29.6% identity in 125 aa overlap).
                     This region is a possible MT-complex-specific genomic island (See Becq et al., 2007)."
                     /codon_start=1
                     /transl_table=11
                     /product="PE family protein PE10"
                     /protein_id="YP_177785.1"
                     /db_xref="GI:57116824 GeneID:887090"
                     /translation="SFAGAEAANASQLQSIARQVRGAVNAVAGQVTGNGGSGNSGTSAAAANPNSDNTASIADRGTSAIMTTASA
                     TASSTGVDGGIAATYAVASQWDGGYVANYTITQFGRDFDDRLAVAIHFA"
     CDS             complement(1218356..1219282)
                     /gene="PE12"
                     /locus_tag="Rv1172c"
                     /experiment="EXISTENCE: identified in proteomics study"
                     /note="Rv1172c, (MTV005.08c), len: 308 aa. PE12, Member of the Mycobacterium
                     tuberculosis PE family of proteins (see Brennan & Delogu 2002), e.g.
                     P71748|Z81368|MTCY253.25C (361 aa), FASTA scores: opt: 483, E(): 7.8e-22, (46.4%
                     identity in 192 aa overlap)."
                     /codon_start=1
                     /transl_table=11
                     /product="PE family protein PE12"
                     /protein_id="YP_177793.1"
                     /db_xref="GI:57116839 GeneID:885988"
                     /translation="MSFVFAAPEALAAAAADMAGIGSTLNAANVVAAVPTTGVLAAAADEVSTQVAALLSAHAQGYQQLSRQMMT
                     AFHDQFVQALRASADAYATAEASAAQTMVNAVNAPARALLGHPLISADASTGGGSNALSRVQSMFLGTGGSSALGGSAAANAAAS
                     GALQLQPTGGASGLSAVGALLPRAGAAAAAALPALAAESIGNAIKNLYNAVEPWVQYGFNLTAWAVGWLPYIGILAPQINFFYYL
                     GEPIVQAVLFNAIDFVDGTVTFSQALTNIETATAASINQFINTEINWIRGFLPPLPPISPPGFPSLP"
     CDS             1255604..1255903
                     /gene="PE13"
                     /locus_tag="Rv1195"
                     /experiment="EXISTENCE: identified in proteomics study"
                     /note="Rv1195, (MTCI364.07), len: 99 aa. PE13, Member of Mycobacterium tuberculosis
                     PE family (see Brennan & Delogu 2002), e.g. Y0DP_MYCTU|Q50615 hypothetical
                     glycine-rich 40.8 kd protein (498 aa), FASTA scores: opt: 307, E(): 1.4e-12, (56.3%
                     identity in 96 aa overlap), similar to MTCY21C12.10c (99 aa), FASTA scores: opt:295,
                     E(): 1.9e-11,(51.5% identity in 97 aa overlap)."
                     /codon_start=1
                     /transl_table=11
                     /product="PE family protein PE13"
                     /protein_id="YP_177794.1"
                     /db_xref="GI:57116840 GeneID:886044"
                     /translation="MSFVMAYPEMLAAAADTLQSIGATTVASNAAAAAPTTGVVPPAADEVSALTAAHFAAHAAMYQSVSARAAA
                     IHDQFVATLASSASSYAATEVANAAAAS"
     CDS             complement(1272718..1273050)
                     /gene="PE14"
                     /locus_tag="Rv1214c"
                     /experiment="EXISTENCE: identified in proteomics study"
                     /note="Rv1214c, (MTCI364.26c), len: 110 aa. PE14, Member of Mycobacterium
                     tuberculosis PE family (see citation below),appears to be frameshifted but sequence
                     appears to be correct. The 5'-end is atypical as first 9 aa appear to be missing."
                     /codon_start=1
                     /transl_table=11
                     /product="PE family protein PE14"
                     /protein_id="YP_177797.1"
```

```
                     /db_xref="GI:57116843 GeneID:888362"
                     /translation="MLASAATDLAGIGSALSAANAAAAAPTTAMLAACADEVSAVVASLFARHAQAYQALSLQATAFHQQFVQAL
                     TGAGGAYAAAEAVNAAVAQSVQQDVLNVINAPTQALFDR"
     CDS             1472197..1472501
                     /gene="PE15"
                     /locus_tag="Rv1386"
                     /experiment="EXISTENCE: identified in proteomics study"
                     /note="Rv1386, (MTCY21B4.03), len: 102 aa. PE15, Member of Mycobacterium tuberculosis
                     PE family (see Brennan & Delogu 2002), similar to many e.g. G913039 ORF 3' of PGRS
                     tandem repeat (polymorphic GC-rich sequence) (100 aa), FASTA scores: opt: 149, E():
                     0.0013, (31.5% identity in 92 aa overlap); also similar to Q49943|U1756A (99 aa)
                     (34.7% identity in 95 aa overlap) and G466937|U1620K (100 aa) (36.2% identity in 69
                     aa overlap). A core mycobacterial gene; conserved in mycobacterial strains (See
                     Marmiesse et al., 2004). Predicted to be an outer membrane protein (See Song et al.,
                     2008)."
                     /codon_start=1
                     /transl_table=11
                     /product="PE family protein PE15"
                     /protein_id="YP_177805.1"
                     /db_xref="GI:57116857 GeneID:886757"
                     /translation="MTLRVVPESLAGASAAIEAVTARLAAAHAAAAPFIAAVIPPGSDSVSVCNAVEFSVHGSQHVAMAAQGVEE
                     LGRSGVGVAESGASYAARDALAAASYLSGG"
     CDS             1513768..1515354
                     /gene="PE16"
                     /locus_tag="Rv1430"
                     /note="Rv1430, (MTCY493.24c), len: 528 aa. PE16, Member of the Mycobacterium
                     tuberculosis PE family of proteins (see citation below), e.g. Y0D4_MYCTU|Q50594
                     (55.9% identity in 127 aa overlap). The C-terminus shows similarity to
                     Q49633|LEPB1170_F3_112 hypothetical Mycobacterium leprae protein (391 aa), FASTA
                     scores: opt: 342, E(): 1.2e-13,(29.8% identity in 292 aa overlap). Possible TMhelix
                     aa 500-522."
                     /codon_start=1
                     /transl_table=11
                     /product="PE family protein PE16"
                     /protein_id="YP_177810.1"
                     /db_xref="GI:57116862 GeneID:886652"
                     /translation="MSFVFAVPEMVAATASDLASLGAALSEATAAAAIPTTQVLAAAADEVSAAIAELFGAHGQEFQALSAQASA
                     FHDRFVRALSAAAGWYVDAEAANAALVDTAATGASELGSGGRTALILGSTGTPRPPFDYMQQVYDRYIAPHYLGYAFSGLYTPAQ
                     FQPWTGIPSLTYDQSVAEGAGYLHTAIMQQVAAGNDVVVLGFSQGASVATLEMRHLASLPAGVAPSPDQLSFVLLGNPNNPNGGI
                     LARFPGLYLQSLGLTFNGATPDTDYATTIYTTQYDGFADFPKYPLNILADVNALLGIYYSHSLYYGLTPEQVASGIVLPVSSPDT
                     NTTYILLPNEDLPLLQPLRGIVPEPLLDLIEPDLRAIIELGYDRTGYADVPTPAALFPVHIDPIAVPPQIGAAIGGPLTALDGLL
                     DTVINDQLNPVVTSGIYQAGAELSVAAAGYGAPAGVTNAIFIGQQVLPILVEGPGALVTADTHYLVDAIQDLAAGDLSGFNQNLQ
                     LIPATNIALLVFAAGIPAVAAVAILTGQDFPV"
     CDS             1752304..1753236
                     /gene="PE17"
                     /locus_tag="Rv1646"
                     /experiment="EXISTENCE: identified in proteomics study"
                     /note="Rv1646, (MTCY06H11.11), len: 310 aa. PE17, Member of the Mycobacterium
                     tuberculosis PE family of proteins (see citation below), similar to many e.g.
                     YW36_MYCTU|Q10873 hypothetical 53.7 kd protein cy39.36c (558 aa), FASTA scores, opt:
                     411, E(): 1.3e-15, (34.4% identity in 320 aa overlap)."
                     /codon_start=1
                     /transl_table=11
                     /product="PE family protein PE17"
                     /protein_id="YP_177825.1"
                     /db_xref="GI:57116896 GeneID:885486"
                     /translation="MSFLTVAPDMVTAAAGNLESVGSALNEAAAAAAPATVGLAAPAADRVSAVVAAMLGAYARDFQGISAQIAG
                     FHNQFVGALRGGAAAYASAEAANVQQTVVNAVNAPAQALLGHPLIGPETVGSSAAAVSFGFGPLLLAGSDPLLAVPFSYPASLPT
                     PFGPVTMTLNGSFDPLTQQVVFDSGSLTAPAPFVYGLGAVGPALTTMTALQNSGTAFSGAVQSGNLLGAAGALLQAPGNAVTGFL
                     FGQTAISQSIPGPSNLGYESVGISVPVGGLLAPLQPVTVTLTPTSGMPTAIQLSGTQFGGLLPALLNGF"
     CDS             1911521..1911820
                     /gene="PE18"
                     /locus_tag="Rv1788"
                     /note="Rv1788, (MTV049.10), len: 99 aa. PE18, Member of the Mycobacterium
                     tuberculosis PE family of gly-, ala-rich proteins (see citation below), similar to
                     Z93777|MTCI364.07 Mycobacterium tuberculosis cosmid (99 aa), FASTA scores: opt: 414,
                     E(): 3.6e-20, (72.4% identity in 98 aa overlap)."
                     /codon_start=1
                     /transl_table=11
```

```
                     /product="PE family protein PE18"
                     /protein_id="YP_177834.1"
                     /db_xref="GI:57116910 GeneID:885895"
                     /translation="MSFVTTQPEALAAAAGSLQGIGSALNAQNAAAATPTTGVVPAAADEVSALTAAQFAAHAQIYQAVSAQAAA
                     IHEMFVNTLQMSSGSYAATEAANAAAAG"
     CDS             1912713..1913012
                     /gene="PE19"
                     /locus_tag="Rv1791"
                     /note="Rv1791, (MTV049.13), len: 99 aa. PE19, Member of the Mycobacterium
                     tuberculosis PE family, but no glycine rich C-terminus (see Brennan & Delogu 2002),
                     highly similar to Z93777|MTCI364.07 M.tuberculosis cosmid (99 aa) opt: 430 E():
                     2.4e-21, (75.5% identity in 98 aa overlap)."
                     /codon_start=1
                     /transl_table=11
                     /product="PE family protein PE19"
                     /protein_id="YP_177837.1"
                     /db_xref="GI:57116913 GeneID:885445"
                     /translation="MSFVTTQPEALAAAAANLQGIGTTMNAQNAAAAAPTTGVVPAAADEVSALTAAQFAAHAQMYQTVSAQAAA
                     IHEMFVNTLVASSGSYAATEAANAAAAG"
     CDS             1924329..1924628
                     /gene="PE20"
                     /locus_tag="Rv1806"
                     /experiment="EXISTENCE: identified in proteomics study"
                     /note="Rv1806, (MTV049.28), len: 99 aa. PE20, Member of the Mycobacterium
                     tuberculosis PE family of gly-, ala-rich proteins (see citation below), most similar
                     to Rv1788|MTV049.10|AL022021 (99 aa), FASTA scores: opt: 334,E(): 4.7 e-15, (59.8%
                     identity in 97 aa overlap). This region is a possible MT-complex-specific genomic
                     island (See Becq et al., 2007)."
                     /codon_start=1
                     /transl_table=11
                     /product="PE family protein PE20"
                     /protein_id="YP_177843.1"
                     /db_xref="GI:57116919 GeneID:885537"
                     /translation="MAFVLVCPDALAIAAGQLRHVGSVIAARNAVAAPATAELAPAAADEVSALTATQFNFHAAMYQAVGAQAIA
                     MNEAFVAMLGASADSYAATEAANIIAVS"
     CDS             2226235..2226531
                     /gene="PE22"
                     /locus_tag="Rv2107"
                     /note="Rv2107, (MTCY261.03), len: 98 aa. PE22, Member of mycobacterial PE family (see
                     citation below). A core mycobacterial gene; conserved in mycobacterial strains (See
                     Marmiesse et al., 2004)."
                     /codon_start=1
                     /transl_table=11
                     /product="PE family protein PE22"
                     /protein_id="YP_177858.1"
                     /db_xref="GI:57116945 GeneID:887811"
                     /translation="MSFVNVDPFGMLAAAATLESLGSHMAVSNAAVASVTTKVPPPAADYVSKKLSLFFSSHGQQYQVQAARGTA
                     FHRKLVRTLANGALAYEEVEIANNEGF"
     CDS             2455083..2456231
                     /gene="PE23"
                     /locus_tag="Rv2328"
                     /experiment="EXISTENCE: identified in proteomics study"
                     /note="Rv2328, (MTCY3G12.06), len: 382 aa. PE23, Member of the Mycobacterium
                     tuberculosis PE family (see citation below), similar to others e.g. Q9L8K5|MAG24-1
                     PE-PGRS homolog from Mycobacterium marinum (638 aa), FASTA scores: opt: 495, E():
                     6.6e-18, (34.65% identity in 401 aa overlap); etc."
                     /codon_start=1
                     /transl_table=11
                     /product="PE family protein PE23"
                     /protein_id="YP_177867.1"
                     /db_xref="GI:57116969 GeneID:888111"
                     /translation="MQFLSVIPEQVESAAQDLAGIRSALSASYAAAAGPTTAVVSAAEDEVSTAIASIFGAYGRQCQVLSAQASA
                     FHDEFVNLLKTGATAYRNTEFANAQSNVLNAVNAPARSLLGHPSAAESVQNSAPTLGGGHSTVTAGLAAQAGRAVATVEQQAAAA
                     VAPLPSAGAGLAQVVNGVVTAGQGSAAKLATALQSAAPWLAKSGGEFIVAGQSALTGVALLQPAVVGVVQAGGTFLTAGTSAATG
                     LGLLTLAGVEFSQGVGNLALASGTAATGLGLLGSAGVQLFSPAFLLAVPTALGGVGSLAIAVVQLVQGVQHLSLVVPNVVAGIAA
                     LQTAGAQFAQGVNHTMLAAQLGAPGIAVLQTAGGHFAQGIGHLTTAGNAAVTVLIS"
     CDS             2553766..2554485
                     /gene="PE24"
                     /locus_tag="Rv2408"
```

```
                     /note="Rv2408, (MTCY253.12c), len: 239 aa. Possibly PE24, a member of PE family (see
                     citation below), similar to AAK46440|MT2159 from Mycobacterium tuberculosis strain
                     CDC1551 (491 aa) FASTA scores: opt: 269, E(): 5.4e-08,(38.45% identity in 156 aa
                     overlap) and AAK45466|MT1209 from Mycobacterium tuberculosis strain CDC1551 (308
                     aa),FASTA scores: opt: 265, E(): 6.3e-08, (36.0% identity in 197 aa overlap)."
                     /codon_start=1
                     /transl_table=11
                     /product="Possible PE family-related protein PE24"
                     /protein_id="YP_177880.1"
                     /db_xref="GI:57116986 GeneID:885511"
                     /translation="MLIARPDILCSRGPEAMRAKAADLDLAAAAKTVGVQPAADQVAAAIAAILLSHAQIYQDISTQMAAFHDQL
                     VENRTADSTSYASAEANAQQSLLNAMDAPSWQQRRETVGEVGLPADPAGSGTATAAVAAATTARAGSRSAAQATVAPIGGLKLRR
                     ESALSQPGDLHHHVEVGDALPRVDPFQRGNVGVVAAYTHTDVLLGDLIVIGGVVVPPSTGPGLNPGMAAPVYRLSHHGITLRV"
     CDS             complement(2575131..2575430)
                     /gene="PE25"
                     /locus_tag="Rv2431c"
                     /experiment="EXISTENCE: identified in proteomics study"
                     /note="Rv2431c, (MTCY428.15), len: 99 aa. PE25, Member of the Mycobacterium
                     tuberculosis PE family (see Brennan & Delogu 2002), similar to others e.g.
                     AAK47158|MT2839 from Mycobacterium tuberculosis (275 aa) FASTA scores: opt: 194,E():
                     2.5e-06, (40.0% identity in 95 aa overlap); etc."
                     /codon_start=1
                     /transl_table=11
                     /product="PE family protein PE25"
                     /protein_id="YP_177882.1"
                     /db_xref="GI:57116989 GeneID:885703"
                     /translation="MSFVITNPEALTVAATEVRRIRDRAIQSDAQVAPMTTAVRPPAADLVSEKAATFLVEYARKYRQTIAAAAV
                     VLEEFAHALTTGADKYATAEADNIKTFS"
     CDS             2675881..2677359
                     /gene="PE26"
                     /locus_tag="Rv2519"
                     /note="Rv2519, (MTV009.04), len: 492 aa. PE26, Member of the M. tuberculosis PE
                     family (see citation below), highly similar to many e.g.
                     Q50630|YP91_MYCTU|Rv2591|MT2668.1|MTCY 227.10c (543 aa),FASTA scores: opt: 848, E():
                     3e-30, (39.55% identity in 445 aa overlap)."
                     /codon_start=1
                     /transl_table=11
                     /product="PE family protein PE26"
                     /protein_id="YP_177888.1"
                     /db_xref="GI:57116998 GeneID:888172"
                     /translation="MSRLIVAPDWLASAAAEVQSIGSALSAANAAAAAPTTLLVAAAEDEVSAAAAALFANYGREYQTLSVRFAS
                     LDQQFAQALNSAAASYQTAEATGASLVQTATQGVLGVINAPTEFMFGRSLIGDGADGTAASPIGEPGGILYGDGGNGYSQTTPGA
                     VGGAGGSAGFIGNGGAGGAGGPGAGGGTGGLGGWLWGNNGAAGTGDPVNVAVPLRVENNFPLVNLLVNRGPTVPILLDTGSSSLV
                     IPFWKIGWQNLGLPTGFDVVHYGNGVSIVYADVPTTVDFGGGAATTPTSVHVGILPYPRNLDSLVLIASGGAFGPNGNGILGIGP
                     NVGSYAVSGPGNVVTTDLPGQLNEGTLIDIPGGYMQFGPNTGTPITSVTGAPITVLNVQIGGYDPNGGYWSLPSIFDSGGNHGTL
                     PAVILGTGQTTGYAPPGTVISISIHDNQTLLYQYTTTASNSPVVTADPRLNTGLTPFLLGPVYISNNPSGVGTVVFNYPPP"
     CDS             complement(2908393..2909220)
                     /gene="PE27"
                     /locus_tag="Rv2769c"
                     /experiment="EXISTENCE: identified in proteomics study"
                     /note="Rv2769c, (MTV002.34c), len: 275 aa. PE27, Member of the Mycobacterium
                     tuberculosis PE family (see citation below), highly similar to many (notably in
                     N-terminal part) e.g. P96361|Rv1040c|MTCY10G2.09 from Mycobacterium tuberculosis (275
                     aa), FASTA scores: opt: 1111, E(): 5.9e-52, (68.55% identity in 283 aa overlap)."
                     /codon_start=1
                     /transl_table=11
                     /product="PE family protein PE27"
                     /protein_id="YP_177907.1"
                     /db_xref="GI:57117023 GeneID:888461"
                     /translation="MSFLTTQPEELAAAAGKLETIGSAMVAQNAAAAAPTTTGVIPAAADEISVLQAPLFTAYGTLYQQVSAEAA
                     AVYDLFVKTLGVSAGTYAATEAANSSAAASPLSGIASILGSTPGKVPSWISDIANIFNIGAGNWASAASDLLGLASGGLLPAAEE
                     AALEEGLEGAGLSELGAAEAAVGEAPIAAGLGAAPLAAGLSRASSIGALSVPPSWAGQANLVSSTSTLQGAGWTTAAPHGAAGTV
                     IPGMPGLASATRSSAGFGAPRYGAKPIVVPKPAV"
     CDS             complement(3203035..3203121)
                     /gene="PE27A"
                     /locus_tag="Rv3018A"
                     /note="Rv3018A, len: 28 aa. PE27A, Member of Mycobacterium tuberculosis PE family
                     (see Brennan and Delogu, 2002), most similar to Rv0285 (102 aa), FASTA scores: opt:
                     147, E(): 3.5e-05, (92.85% identity in 28 aa overlap); etc."
```

```
                     /codon_start=1
                     /transl_table=11
                     /product="PE family protein PE27A"
                     /protein_id="YP_007411731.1"
                     /db_xref="GI:448817858 GeneID:3205087"
                     /translation="MTLSVVPEGLAAASAAVEALTARLAAAH"
     CDS             complement(3205385..3205699)
                     /gene="PE29"
                     /locus_tag="Rv3022A"
                     /note="Rv3022A, len: 104 aa. PE29, Member of the Mycobacterium tuberculosis PE family
                     (see Brennan and Delogu, 2002), similar to many others e.g. Rv0285|AL021930_12 from
                     Mycobacterium tuberculosis (102 aa), FASTA scores: opt: 497, E(): 3e-21, (80.39%
                     identity in 102 aa overlap); etc."
                     /codon_start=1
                     /transl_table=11
                     /product="PE family protein PE29"
                     /protein_id="YP_177685.1"
                     /db_xref="GI:57117050 GeneID:3205088"
                     /translation="MTLRVVPEGLAAASAAVEALTARLAAAHAGAAPAITAVVAPAADPVSLQSAVGFSALGSEHAAIAGEGVEE
                     LGRSGVAVGESGIGYAAGDAVAAATYLVSGGSL"
     CDS             3673360..3673656
                     /gene="PE31"
                     /locus_tag="Rv3477"
                     /experiment="EXISTENCE: identified in proteomics study"
                     /note="Rv3477, (MTCY13E12.30), len: 98 aa. PE31, Member of the Mycobacterium
                     tuberculosis PE family (see Brennan & Delogu 2002), similar to
                     O53941|Rv1791|MTV049.13 (99 aa),FASTA scores: opt: 373, E(): 4.3e-18, (64.65%
                     identity in 99 aa overlap); MTCI364.07; MTCY21C12.10c; MTCY1A11.25c; MTC1A11.04;
                     MTCY359.33; etc."
                     /codon_start=1
                     /transl_table=11
                     /product="PE family protein PE31"
                     /protein_id="YP_177975.1"
                     /db_xref="GI:57117110 GeneID:888474"
                     /translation="MSFTAQPEMLAAAAGELRSLGATLKASNAAAAVPTTGVVPPAADEVSLLLATQFRTHAATYQTASAKAAVI
                     HEQFVTTLATSASSYADTEAANAVVTG"
     CDS             complement(3809929..3810228)
                     /gene="PE32"
                     /locus_tag="Rv3622c"
                     /note="Rv3622c, (MTCY15C10.30), len: 99 aa. PE32, Member of the Mycobacterium
                     tuberculosis PE family (see Brennan and Delogu, 2002), but no glycine rich C-terminus
                     present. Similar to others e.g. O53938|Rv1788|MTV049.10 (99 aa),FASTA scores: opt:
                     376, E(): 7.1e-17, (65.6% identity in 96 aa overlap)."
                     /codon_start=1
                     /transl_table=11
                     /product="PE family protein PE32"
                     /protein_id="YP_177999.1"
                     /db_xref="GI:57117136 GeneID:885712"
                     /translation="MSIMHAEPEMLAATAGELQSINAVARAGNAAVAGPTTGVVPAAADLVSLLTASQFAAHAQLYQAISAEAMA
                     VQEQLATTLGISAGSYAATEAANAATIA"
     CDS             3839263..3839547
                     /gene="PE33"
                     /locus_tag="Rv3650"
                     /note="Rv3650, (MTCY15C10.02c), len: 94 aa. PE33, Short protein, member of the
                     Mycobacterium tuberculosis PE family (see Brennan and Delogu, 2002), but without the
                     repetitive gly-rich region, similar to the N-terminal part of many e.g.
                     O53809|Rv0746|MTV041.20 PGRS-family protein (783 aa),FASTA scores: opt: 363, E():
                     2.1e-15, (76.55% identity in 81 aa overlap)."
                     /codon_start=1
                     /transl_table=11
                     /product="PE family protein PE33"
                     /protein_id="YP_178000.1"
                     /db_xref="GI:57117138 GeneID:885832"
                     /translation="MSFVIAAPEALDSAATDLVVLGSTLGAATAAAAAQTTGIVAAAHDEVSAAIAALFSAHGQAYQAASAQAAA
                     FHTRFIRARSRHPQQETTCRRVR"
     CDS             complement(3942123..3942458)
                     /gene="PE34"
                     /locus_tag="Rv3746c"
                     /note="Rv3746c, (MTV025.094c), len: 111 aa. PE34, Probable member of the
```

```
                     Mycobacterium tuberculosis PE family (see citation below), but without the
                     glycine-rich C-terminal part, similar to N-termini of many e.g.
                     O69737|Rv3872|MTV027.07 (99 aa) FASTA scores: opt: 306,E(): 1e-13, (50.5% identity in
                     99 aa overlap); O53215|Rv2490c|MTV008.46 (1660 aa) FASTA scores: opt: 125,E(): 0.99,
                     (34.25% identity in 111 aa overlap). Also weakly similar to MTV008_46; MTCI418B_6;
                     MTCY130_1; MTY25D10_11; MTCY1A11_25; MTCY21B4_13; MTCY21B4_27; MTCY493_2; MTCY28_25;
                     etc."
                     /codon_start=1
                     /transl_table=11
                     /product="Probable PE family protein PE34 (PE family-related protein)"
                     /protein_id="YP_178011.1"
                     /db_xref="GI:57117152 GeneID:885764"
                     /translation="MQSMSFDPAVADIGSQVVNNAFQGLQAGAVAWVSLSSLLPAGAEEVSAWAVTAFTTAATGLLALNQAAQEE
                     LRKAGEVFTAIARMYSDADVRAAACLLEAIPRPGQTLARE"
     CDS             4095182..4095481
                     /gene="PE35"
                     /locus_tag="Rv3872"
                     /experiment="EXISTENCE: identified in proteomics study"
                     /note="Rv3872, (MTV027.07), len: 99 aa. PE35, Some similarity to Mycobacterium
                     tuberculosis conserved PE family proteins (see Brennan & Delogu 2002), e.g.
                     O69713|Rv3746c|MTV025.094c (111 aa), FASTA scores: opt: 306, E(): 5.5e-13, (50.5%
                     identity in 99 aa overlap). Equivalent to AAK48354 from Mycobacterium tuberculosis
                     strain CDC1551 (112 aa) but shorter 14 aa."
                     /codon_start=1
                     /transl_table=11
                     /product="PE family-related protein PE35"
                     /protein_id="YP_178021.1"
                     /db_xref="GI:57117163 GeneID:886191"
                     /translation="MEKMSHDPIAADIGTQVSDNALHGVTAGSTALTSVTGLVPAGADEVSAQAATAFTSEGIQLLASNASAQDQ
                     LHRAGEAVQDVARTYSQIDDGAAGVFAE"
     CDS             complement(4117892..4118125)
                     /gene="PE36"
                     /locus_tag="Rv3893c"
                     /note="Rv3893c, (MTCY15F10.19), len: 77 aa. PE36, Member of the Mycobacterium
                     tuberculosis PE family of conserved proteins (see citation below), similar to others
                     e.g. O53690|Rv0285|MTV035.13 from Mycobacterium tuberculosis (102 aa), FASTA scores:
                     opt: 136, E(): 0.042, (35.6% identity in 73 aa overlap)."
                     /codon_start=1
                     /transl_table=11
                     /product="PE family protein PE36"
                     /protein_id="YP_178025.1"
                     /db_xref="GI:57117167 GeneID:886213"
                     /translation="MVWSVQPEAVLASAAAESAISAETEAAAAGAAPALLSTTPMGGDPDSAMFSAALNACGASYLGVVAEHASQ
                     RGLFAG"
```

```
     misc_feature    complement(3186871..3187947)
                     /gene="PPE47"
                     /locus_tag="Rv3021c"
                     /note="Rv3021c, (MTV012.35c), len: 358 aa. PPE47, Member of Mycobacterium
                     tuberculosis PPE family. Should be continuation of upstream ORF MTV012.36c but is
                     frameshifted due to missing base at 36448 in v012. Sequence has been checked but no
                     error apparent. Very similar to neighbouring ORF O53265|MTV012.32c|Rv3018c from
                     Mycobacterium tuberculosis (434 aa), FASTA scores: opt: 1714, E(): 6.6e-770, (78.3%
                     identity in 355 aa overlap) and AAK47430|MT3101 (strongly in the N-terminal part)
                     (310 aa),FASTA scores: opt: 897, E(): 4.5e-37, (66.95% identity in 227 aa overlap)."
                     /pseudogene="unknown"
                     /db_xref="PSEUDO:CCP45828.1"
     misc_feature    complement(3187935..3188173)
                     /gene="PPE48"
                     /locus_tag="Rv3022c"
                     /note="Rv3022c, (MTV012.36c), len: 81 aa. PPE48, Member of M. tuberculosis PPE family
                     with frameshift due to missing bp in codon 82. The ORF continues in downstream
                     MTV012.35c. The sequence has been checked and no errors were detected. Identical to
                     neigbouring ORF O53265|Rv3018c|MTV012.32c (434 aa), FASTA scores: opt: 526, E():
                     6.2e-26, (100.0% identity in 81 aa overlap); and O69706|Rv739c|MTV025.087c (77
                     aa),FASTA scores: opt: 392, E(): 3.4e-18, (72.7% identity in 77 aa overlap)."
                     /pseudogene="unknown"
```

```
     CDS             105324..106715
                     /gene="PPE1"
                     /locus_tag="Rv0096"
                     /experiment="EXISTENCE: identified in proteomics study"
                     /note="Rv0096, (MTCY251.15), len: 463 aa. PPE1, Member of the Mycobacterium
                     tuberculosis PPE family, similar to many. A core mycobacterial gene; conserved in
                     mycobacterial strains (See Marmiesse et al., 2004)."
                     /codon_start=1
                     /transl_table=11
                     /product="PPE family protein PPE1"
                     /protein_id="YP_177690.1"
                     /db_xref="GI:57116689 GeneID:886938"
                     /translation="MAIPPEVHSGLLSAGCGPGSLLVAAQQWQELSDQYALACAELGQLLGEVQASSWQGTAATQYVAAHGPYLA
                     WLEQTAINSAVTAAQHVAAAAAYCSALAAMPTPAELAANHAIHGVLIATNFFGINTVPIALNEADYVRMWLQAADTMAAYQAVAD
                     AATVAVPSTQPAPPIRAPGGDAADTRLDVLSSIGQLIRDILDFIANPYKYFLEFFEQFGFSPAVTVVLALVALQLYDFLWYPYYA
                     SYGLLLLPFFTPTLSALTALSALIHLLNLPPAGLLPIAAALGPGDQWGANLAVAVTPATAAVPGGSPPTSNPAPAAPSSNSVGSA
                     SAAPGISYAVPGLAPPGVSSGPKAGTKSPDTAADTLATAGAARPGLARAHRRKRSESGVGIRGYRDEFLDATATVDAATDVPAPA
                     NAAGSQGAGTLGFAGTAPTTSGAAAGMVQLSSHSTSTTVPLLPTTWTTDAEQ"
     CDS             complement(177543..179309)
                     /gene="PE1"
                     /locus_tag="Rv0151c"
                     /experiment="EXISTENCE: identified in proteomics study"
                     /note="Rv0151c, (MTCI5.25c), len: 588 aa. PE1, Member of the Mycobacterium
                     tuberculosis PE family (see citation below), with N-terminal region similar to others
                     e.g. MTV032_2 PE_PGRS family from Mycobacterium tuberculosis (468 aa), FASTA scores:
                     opt: 1125, E(): 0, (46.3% identity in 456 aa overlap); MTCY493_24 from Mycobacterium
                     tuberculosis FASTA score: (42.5% identity in 558 aa overlap). Also similar to
                     upstream ORF MTCI5.26c FASTA score: (54.7% identity in 464 aa overlap). Also shows
                     similarity to C-terminal part of some PPE family proteins e.g. MTV049_21 from
                     Mycobacterium tuberculosis FASTA score: (41.5% identity in 591 aa overlap)."
                     /codon_start=1
                     /transl_table=11
                     /product="PE family protein PE1"
                     /protein_id="YP_177695.1"
                     /db_xref="GI:57116694 GeneID:886857"
                     /translation="MAPFGFTPKARHNRGVALRSTYRLDGWVMGPVDKEGWGLSYVFAQPSVLAAAATDLAGIGSAINQATAAVA
                     APTTGLAAAAADEVSTALATLFGAYGQQFQAISAQVAAFHNEFTQRLAAAANAFVNAEATNTSALVQEATAGLFKPTSPPVLPPM
                     FNQNTAIIMGGTGSPIPTPSYVNAITTLFIDPVVSNPVVKALVTPEELYPITGVKSLPFQTSVQLGLQILDGAIWEQINAGNHVT
                     VFGYSQSAVIASLEMQHLISLGPNAPSPSQLNFILIGNEMNPNGGILARIPGLNVTTLGLPFYGATPDNPYPTTTYTLEYDGFAD
                     FPRYPLNVLSDINAVFGILTVHTTYADLTPAQIASATQLPTQGTTSNTYYIIETEHLPLLAPLRAIPVIGPPLAALVEPNLEVIV
                     NLGYGDPRFGYSTSPANVPTPFGLFPDVPASVVADALVAGTQQGVNDFMVELPAALNTLPQTPMPAFPPYVPTLLPPPPPPQPAT
                     LINIADTFASVVSTGYSILLPTADLGLAFVTILPAYDLTLFVNQLAAGNLRAAIELPLAATIGLAALGGMIEFIAIVVTLADITQ
                     QLQSFSI"
     CDS             complement(307877..309547)
                     /gene="PPE2"
                     /locus_tag="Rv0256c"
                     /experiment="EXISTENCE: identified in proteomics study"
                     /note="Rv0256c, (MTV034.22c), len: 556 aa. PPE2, Member of the M. tuberculosis PPE
                     family, similar to many e.g. Rv0280, Rv0286, etc. Equivalent to Z98756|MLCB2492.30
                     from Mycobacterium leprae (572 aa), FASTA scores: opt: 1837,E(): 0, (62.9% identity
                     in 461 aa overlap). A core mycobacterial gene; conserved in mycobacterial strains
                     (See Marmiesse et al., 2004)."
                     /codon_start=1
                     /transl_table=11
                     /product="PPE family protein PPE2"
                     /protein_id="YP_177704.1"
                     /db_xref="GI:57116708 GeneID:886684"
                     /translation="MTAPIWMASPPEVHSALLSSGPGPGPLLVSAEGWHSLSIAYAETADELAALLAAVQAGTWDGPTAAVYVAA
                     HTPYLAWLVQASANSAAMATRQETAATAYGTALAAMPTLAELGANHALHGVLMATNFFGINTIPIALNESDYARMWIQAATTMAS
                     YQAVSTAAVAAAPQTTPAPQIVKANAPTAASDEPNQVQEWLQWLQKIGYTDFYNNVIQPFINWLTNLPFLQAMFSGFDPWLPSLG
                     NPLTFLSPANIAFALGYPMDIGSYVAFLSQTFAFIGADLAAAFASGNPATIAFTLMFTTVEAIGTIITDTIALVKTLLEQTLALL
                     PAALPLLAAPLAPLTLAPASAAGGFAGLSGLAGLVGIPPSAPPVIPPVAAIAPSIPTPTPTPAPAPAPTAVTAPTPPPGPPPPPV
                     TAPPPVTGAGIQSFGYLVGDLNSAAQARKAVGTGVRKKTPEPDSAEAPAAAAAPEEQVQPQRRRRPKIKQLGRGYEYLDLDPETG
                     HDPTGSPQGAGTLGFAGTTHKASPGQVAGLITLPNDAFGGSPRTPMMPGTWDTDSATRVE"
     CDS             339364..340974
                     /gene="PPE3"
                     /locus_tag="Rv0280"
                     /experiment="EXISTENCE: identified in proteomics study"
                     /note="Rv0280, (MTV035.08), len: 536 aa. PPE3, Member of the Mycobacterium
```

tuberculosis PPE family, similar to others e.g. Z80108|MTCY21B4_4|Rv0453 from Mycobacterium tuberculosis (539 aa), FASTA scores: opt: 1131, E(): 0,(51.7% identity in 540 aa overlap)."
/codon_start=1
/transl_table=11
/product="PPE family protein PPE3"
/protein_id="YP_177709.1"
/db_xref="GI:57116714 GeneID:886619"
/translation="MTLWMASPPEVHSALLSSGPGPGSVLSAAGVWSSLSAEYAAVADELIGLLGAVQTGAWQGPSAAAYVAAHA
PYLAWLMRASETSAEAAARHETVAAAYTTAVAAMPTLVELAANHTLHGVLVATNFFGINTIPIALNEADYARMWTQAASTMATYQ
AVAEAAVASAPQTTPAPPILAAEAADDDHDHDHDHGGEPTPLDYLVAEILRIISGGRLIWDPAEGTMNGIPFEDYTDAAQPIWWV
VRAIEFSKDFETFVQELFVNPVEAFQFYFELLLFDYPTHIVQIVEALSQSPQLLAVALGSVISNLGAVTGFAGLSGLAGMQPAAI
PALAPVAAAPSTLPAVAMAPTMAAPGAAVASAAAPASAPAASTVASATPAPPPAPGAAGFGYPYAIAPPGIGFGSGMSASASAQR
KAPQPDSAAAAAAAAAVRDQARARRRRRVTRRGYGDEFMDMNIDVDPDWGPPPGEDPVTSTVASDRGAGHLGFAGTARREAVADA
AGMTTLAGDDFGDGPTTPMVPGSWDPDRDAPGSAEPGDRG"

CDS 349935..351476
/gene="PPE4"
/locus_tag="Rv0286"
/note="Rv0286, (MTV035.14), len: 513 aa. PPE4, Member of the Mycobacterium tuberculosis PPE family, similar to others e.g. AL0212|MTV012_32 from Mycobacterium tuberculosis (434 aa), FASTA scores: opt: 958, E(): 0,(43.5% identity in 522 aa overlap)."
/codon_start=1
/transl_table=11
/product="PPE family protein PPE4"
/protein_id="YP_177711.1"
/db_xref="GI:57116716 GeneID:886607"
/translation="MAAPIWMASPPEVHSALLSNGPGPGSLVAAATAWSQLSAEYASTAAELSGLLGAVPGWAWQGPSAEWYVAA
HLPYVAWLTQASADAAGAAAQHEAAAAAYTTALAAMPTLAELAANHVIHTVLVATNFFGINTIPITLNEADYVRMWLQAAAVMGL
YQAASGAALASAPRTVPAPTVMNPGGGAASTVGAVNPWQWLLALLQQLWNAYTGFYGWMLQLIWQFLQDPIGNSIKIIIAFLTNP
IQALITYGPLLFALGYQIFFNLVGWPTWGMILSSPFLLPAGLGLGLAAIAFLPIVLAPAVIPPASTPLAAAAVAAGSVWPAVSMA
VTGAGTAGAATPAAGAAPSAGAAPAPAAPATASFAYAVGGSGDWGPSLGPTVGGRGGIKAPAATVPAAAAAAATRGQSRARRRRR
SELRDYGDEFLDMDSDSGFGPSTGDHGAQASERGAGTLGFAGTATKERRVRAVGLTALAGDEFGNGPRMPMVPGTWEQGSNEPEA
PDGSGRGGGDGLPHDSK"

CDS complement(366150..372764)
/gene="PPE5"
/locus_tag="Rv0304c"
/experiment="EXISTENCE: identified in proteomics study"
/note="Rv0304c, (MTCY63.9c), len: 2204 aa. PPE5, Member of the Mycobacterium tuberculosis PE family (PPE, MPTR),similar to others e.g. Z95324|MTY13E10_16 from Mycobacterium tuberculosis (1443 aa), FASTA scores: E(): 0,(50.6% identity in 1403 aa overlap); Y04H_MYCTU|Q10778 from Mycobacterium tuberculosis (734 aa), FASTA scores: opt: 989, E(): 0, (42.3% identity in 522 aa overlap). This region is a possible MT-complex-specific genomic island (See Becq et al., 2007)."
/codon_start=1
/transl_table=11
/product="PPE family protein PPE5"
/protein_id="YP_177714.1"
/db_xref="GI:57116719 GeneID:886592"
/translation="MNLVSTTSGMSGFLNVGALGSGVANVGNTISGIYNVGTSDLSTPAVNSGLANIGTNIAGLLRDGAGTAAIN
LGLANHGNLNVGFASLGGFNFGGATIGHNNVGIGNTGIFDVGLANLGSYNIGFGNLGDDNLGFGNFGSYNIGFGNVGNDNLGFAN
AGGGNIGFANTGSNNVGFGNTGSNNVGIGLTGNGQIGFGSFNSGSGNIGLFNSGSNNIGFFNSGSGNFGIANSGSFNTGIGNTGN
TNTGLFNSGDVNTGAFNPGSFNTGSFNTGSFNTGGFNPGNTNTGYLNIGNYNTGIANTGDVDTGAFITGNYSNGLFLSGDYQGLV
GLNLVIDMPLPISLGVNIPIDIPITASAGNITLMGVTIPPTGDIVLSSIAGQRAHFGPIITIPNITVVGPTTTVAIGGPNTAITIT
GGGAIRIPLISIPAAPGFGNSTTNPSSGFFNTGAGGASGFGNFGGANSGFWNLASATSGASGLLNVGALGSGLANVGTTVSGFYN
TSTSDLATPAFNSGLANISTSIAGLLRDSTGTMVLNLGLANHGTLNVGIANLGDYNIGFANLGSANFGSANIGGNNIGGANTGIF
DIGLANLGSYNIGFGNFGDDNLGFGNLGSYNVGFGNLGNDNLGFANTGSNNIGFANTGSNNIGIGLTGDGQIGFGSLNSGSGNIG
LFNSGSGNIGFFNSGNGNVGIGNTGTANFGLGNTGSTNTGFFNSGDVNTGIGNTGSFNTGSFNPGDSNTGDFNPGSYNTGLGNTG
DVDTGAFISGSYSNGFLWSGNYQGLIGLHAALAIPEIALTFGVDIPIHIPINIDAGVVTLQGFSIVAAENNIDFTPIIIPTINIT
LPTAAITVGGPTTSIGITASAGIGSITIPIIDIPATSGFGNSTTSPSSGFFNSGAGSASGFLNVVAGASGISGYLNVGALGSGVT
NVGHTVSGFYNASALDLVTPAFASGLMRDGMGTMTLNLGLANLGSNNAGFGNTGIFDVGVANLGNYNIGFGNFGDDNLGFANLGS
YNIGVANTGSNNIGFANTGSNNIGIGLTGTGQIGIGALNSGSGNIGLFNSGDGNIGFFNSGTGNFGIGNTGTGNFGIGNSGSTST
GLFNSGDGNTGGFNPGNFNTGNFNTGSFNTGGFNAGNTNTGHFNTGNYNTGIANTGDVSTGAFISGNYSNGILWRGDYQGLIGYS
YALTIPEIPAHLDVNIPIDIPITGSFTDLVVDNFTIPIIGFESFAFSFHIHTEPDIGPIIVPSFVLSVPTFAIAVGGPTTAINIS
ATAGLGPITIPIIDIPAAPGIGNSTTSPSSGFFNTGAGTASGFGNVGGNTSGLWNLASAASGVSGLLNVGALGSGVANVGNTISG
IYNTSPLDLGTPAFGSGLANIAGLLQGGAGTTILDLAGLGNLNVGLANLGGSNFGIGNTGIFNVGFANVGNHNIGLANLGNYSVG
FANSGNYHIGIANTGSANIGFANTGSGNIGIGLTGTGQIGFGSFNSGSHNIGLFNSGDGNVGFFNSGTGNVGIGNTGTANFGIAN
SGSFNTGLGNTGSTNTGLFNPGNVNTGVGNTGSINTGSINTGSFNTGSTNTGSFNLGDHNTGSFNSGDYNTGYFNAGDYNTGVAN
TGNVNTGAFISGNYSNGFFWRGDYQGLIGLSTTITIPEIPYRYDLSVPIDIPITGTVVATTPNSFTIPGFQIRVLLGPAAVLVNE

```
                     MIGPITIDVNQVIAIDSPIQQTISMVGTGGFGPIPIGISIGGTPGFGNSTTGPSSGFFHTGAGHVSGFGNFGAGNMSGSGNFGAG
                     NSGFFNAGGLGNSGLLNFGALQSGLANLGNTISGVYNTSTLDLATPAFGSGIANIGANLAGLFLDNTGNLTLNFGVANQGGLNAG
                     IGNLGSVNIGFVNTGDSNLGIGNLGDLNFGGVNIGGNNIGIANTGIFDIGLANLGSYNIGLANLGDDNLGFGNAGSYNIGFANFG
                     SDNLGFANTGSYNIGFANTGNNNIGVGLTGNGQIGIGSLNSGSNNIGLFNSGSGNIGFFNSGTGNVGIFNTGTGNFGLANSGGFN
                     TGIGNAGSTNTGVFNPGDLNTGSFNPGSFNTGGFNPGSGNTGYLNTGDYNTGVANTGDVDTGAFITGSYSNGFLVSGDYQGLIGL
                     PLLGIPVTPGYFNLTGGPSSGFFNSGAGSVSGFVNSGAGLSGYLNTGALGSGVANVGNTISGWLNASALDLATPGFLSGIGNFGT
                     NLAGFFRG"
     CDS             complement(372820..375711)
                     /gene="PPE6"
                     /locus_tag="Rv0305c"
                     /experiment="EXISTENCE: identified in proteomics study"
                     /note="Rv0305c, (MTCY63.10c), len: 963 aa. PPE6, Member of the Mycobacterium
                     tuberculosis PE family (PPE, MPTR),similar to others e.g. Y04H_MYCTU|Q10778 from
                     Mycobacterium tuberculosis (734 aa), FASTA scores: opt: 1340, E(): 0,(40.9% identity
                     in 815 aa overlap)."
                     /codon_start=1
                     /transl_table=11
                     /product="PPE family protein PPE6"
                     /protein_id="YP_177715.1"
                     /db_xref="GI:57116720 GeneID:885978"
                     /translation="MDFVVSAPEVNSLRMYLGAGSGPMLAAAAAWDGLADELAVAASWFGSVTSGLADAAWRGPAAVAMARAVAP
                     YLGWLISATAQAEQAAAQARVAVATFEAARAATVHPAIVAANRAVLVSLVSSNLLGFNAPAIAATEAAYERMWAQDVAAMVGYHA
                     GASAAVSALMPFTQQLKKLAGLSERLTSAAAAAAGPPSAAGFNLGLANVGANNVGNGNVGVFNVGFGNLGSYNLGFANLGSDNLG
                     LANLGGHNIGFANTGSNNVGFGNTGSNNVGIGLTGNGQIGFGSFNSGSHNIGLFNSGSGNVGLFNSGTGNFGIGNSGTGNFGLGN
                     TGSTNTGWFNTGDVNTGGFNPGSYNTGNFNTGNYNTGSFNAGNYNTGYFNTGDYNTGVANTGNVNTGAFIAGNYSNGVLWRGDYQ
                     GLIGADIALEIPAIPINAQLFSMPIHQVMVMPGSVMTIPGMRLPFTSIVPFVVYYGPVELPQSTLTLPTVTITVGGPTTTIDGNL
                     TGMVGGVSIPLIKIPAAPGFGNSTTSPSSGFFNAGAGTASGFGNFGGGASGFWNLASATSGLSGFGNVGALGSGVANVGNTISGL
                     YNTSTSNLATPAFNSGLLHHSVGTMTLNFGLANVGGNNVGGANAGIFNVGLANLGDYNIGFGNLGGDNLGFAHAGSYNIGFANTG
                     SNNLGFANTGDNNIGFANIGSNNIGIGLTGSGQIGFGSLNSGSHNIGLFNSGDGNIGLFNSGSGNFGIGNAGTGNWGIGNSGAGN
                     FGIGNAGSTNTGLFNSGDLNTGSLNPGSYNTGSVNTGSVNTGGFNAGNYNTGYFNTGDLQHRHGEHRQYQHRRFHLRQPQQRPSV
                     AGRQPGSDRPRHRRRHSRNPDCERRREYPDSHTDHRQLHGHRIQRARSSTEHSRHCYFFRTRRYRPLHRPSDTDNRSHTCGHGGW
                     THYRDQYRRHCGRRRHQHPDYPYSSDSRLRQLDRRTVVGLLQ"
     CDS             complement(424269..424694)
                     /gene="PPE7"
                     /locus_tag="Rv0354c"
                     /experiment="EXISTENCE: identified in proteomics study"
                     /note="Rv0354c, (MTCY13E10.14c), len: 141 aa. PPE7, Member of the Mycobacterium
                     tuberculosis PPE family, similar to others e.g. MTCY63_9 from Mycobacterium
                     tuberculosis (2411 aa), FASTA scores: E(): 3.6e-11, (47.6% identity in 103 aa
                     overlap). Possible continuation of ORF upstream, but no sequence error apparent."
                     /codon_start=1
                     /transl_table=11
                     /product="PPE family protein PPE7"
                     /protein_id="YP_177720.1"
                     /db_xref="GI:57116725 GeneID:886498"
                     /translation="MSVCVIYIPFKGCVKHVSVTIPITTEHLGPYEIDASTINPDQPIDTAFTQTLDFAGSGTVGAFPFGFGWQQ
                     SPGFFNSTTTPSSGFFNSGAGGASGFLNDAAAAVSGLGNVFTETSGFFNAGGVGIRASKTSATCCRAGRT"
     CDS             complement(424777..434679)
                     /gene="PPE8"
                     /locus_tag="Rv0355c"
                     /experiment="EXISTENCE: identified in proteomics study"
                     /note="Rv0355c, (MTCY13E10.15c, MTCY13E10.16c,MTCY13E10.17c), len: 3300 aa. PPE8,
                     Member of the Mycobacterium tuberculosis PPE family, similar to others e.g.
                     AL009198|MTV004_5 from Mycobacterium tuberculosis (3716 aa), FASTA scores: opt: 2906,
                     E(): 0, (40.9% identity in 3833 aa overlap); MTV004_3 FASTA scores: (39.0% identity
                     in 3531 aa overlap); etc. Gene contains large number of clustered Major Polymorphic
                     Tandem Repeats (MPTR). Related to MTCY13E10.16c, E(): 0; MTCY13E10.17c, E(): 0;
                     MTCY48.17,E(): 0; MTCY98.0034c, E(): 0; MTCY03C7.23 E(): 0; MTCY98.0031c, E(): 0;
                     MTCY31.06c, E(): 5.6e-17; MTCY359.33,E(): 2.3e-16. Nucleotide position 426909 in the
                     genome sequence has been corrected, A:C resulting in W2591G."
                     /codon_start=1
                     /transl_table=11
                     /product="PPE family protein PPE8"
                     /protein_id="YP_177721.2"
                     /db_xref="GI:448824742 GeneID:886491"
                     /translation="MSFAVLPPEINSARLYVGAGLAPMLDAAAAWDGLADELGSAAASFSAVTAGLAGSSWLGAASTAMTGAAAP
                     YLGWLSAAAAQAQQAATQTRLAAAAFEAALAATVHPAIISANRALFVSLVVSNLLGQNAPAIAATEAAYEQMWAQDVAAMFGYHA
                     GASAAVSALTPFGQALPTVAGGGALVSAAAAQVTTRVFRNLGLANVGEGNVGNGNVGNFNLGSANIGNGNIGSGNIGSSNIGFGN
                     VGPGLTAALNNIGFGNTGSNNIGFGNTGSNNIGFGNTGDGNRGIGLTGSGLLGFGGLNSGTGNIGLFNSGTGNVGIGNSGTGNWG
```

IGNSGNSYNTGFGNSGDANTGFFNSGIANTGVGNAGNYNTGSYNPGNSNTGGFNMGQYNTGYLNSGNYNTGLANSGNVNTGAFIT
GNFNNGFLWRGDHQGLIFGSPGFFNSTSAPSSGFFNSGAGSASGFLNSGANNSGFFNSSSGAIGNSGLANAGVLVSGVINSGNTV
SGLFNMSLVAITTPALISGFFNTGSNMSGFFGGPPVFNLGLANRGVVNILGNANIGNYNILGSGNVGDFNILGSGNLGSQNILGS
GNVGSFNIGSGNIGVFNVGSGSLGNYNIGSGNLGIYNIGFGNVGDYNVGFGNAGDFNQGFANTGNNNIGFANTGNNNIGIGLSGD
NQQGFNIASGWNSGTGNSGLFNSGTNNVGIFNAGTGNVGIANSGTGNWGIGNPGTDNTGILNAGSYNTGILNAGDFNTGFYNTGS
YNTGGFNVGNTNTGNFNVGDTNTGSYNPGDTNTGFFNPGNVNTGAFDTGDFNNGFLVAGDNQGQIAIDLSVTTPFIPINEQMVID
VHNVMTFGGNMITVTEASTVFPQTFYLSGLFFFGPVNLSASTLTVPTITLTIGGPTVTVPISIVGALESRTITFLKIDPAPGIGN
STTNPSSGFFNSGTGGTSGFQNVGGGSSGVWNSGLSSAIGNSGFQNLGSLQSGWANLGNSVSGFFNTSTVNLSTPANVSGLNNIG
TNLSGVFRGPTGTIFNAGLANLGQLNIGSANLGDFNLGSGNVGSFNVFSGNQGSYNIGPANLGNYNIGFANLGNYNIGFGNAGDF
NQGFANTGNNNIGFANTGNNNIGIGLSGDNQQGFNFAGGWNSGTANIGLFNSGTNNVGIGNSGTGNWGIGNSGSGNTGIGNTGST
NTGFFNTGIVNTGVANAGSYNTGWYNTGDTNTGIANLGDFNTGFYNTGNFSTGFANQGDIATGAFITGDMGNGAFWRGDQQGLFS
AGYRVHVPEIPAHVTVEVPVNIPITASFTNTVYSGITLEQINFGFTIDIAGIPLLAGAISKAVLPPITGTGPAITVNIGDPGGST
AIRIPATASVGPFDVTFVNIAATTGFFNATTDPSSGFFNGGPGTVSGIANIGANISGFQNVANSATSGFNNYGSLQSGLANLGDT
VSGVFNTGIGAPANVSGMFNIGSNLAGFFHDQATGMSMFNLGLGNIGQFNVGFSNVGDSNAGLANIGSFNLGSGNLGSFNVFGGN
QGSYNIGPANLGNYNIGLGNLGSYNFGFGNAGDFNLGFANTGNNNIGFANTGNNNIGIGLSGDNQQGFNFAGGWNSGSGNSGLFN
SGTNNIGLFNSGTGNIGIGNSGTGNWGIANTGDTNTGIFNTGDVNTGLLNAGNVNTGIFNTGHYNTGSFNAGSFNTAGFNPGSYN
TGYLNTGSYNTGLANSGDVNTGGFITGNYSNGFWWRGDYQGLAGISQTITVPDTAVPVKLHVPIFLDIPVTGTLGTFTVHGFRFP
EITGDIFLIGIPFNAATLDAFSFPNISIVLPNIGINLGSGPDPLIDIAGTGGLLPIKIPLIDIPAAPGFGNSTTTPSSGFFNAGT
GTVSGVGNVGSNSSGFFNLTSGSSGISGVQNFGELISGGFNFGNTVSGLVNASTLGLSMPANLSGGGNVGATVAGFVNNTQILNL
GFGNVGSGNVGHGNIGDSNVGLGNLGNANVGHGNIGSFNVFSGNRGSYNIGPANLGNYNIGLGNLGSYNFGFGNAGDFNLGFANS
GSNNIGFANTGNNNIGIGLSGHNQQGFGSWNSGTANTGLFNSGTNNIGLFNSGTGNIGIGNSGIGNTGIGNPGVGNTGLGNSGTG
NWGLWNPGTGNMGVANVGTYNTGGYNVGSTNTGIANVGIANTGSYNTGSTNTGSFNDGDFNTGFYNTGDYNTGFYNTGDVNTGAF
IGGNFSNGAFWQSDHQGQWGAHYAITVPQIPLLNFSLNIPVNIPIHLDFGTLAVNGFQIPAITLRALGVTHFSVGPIIVPRIAGT
LPVIDINIGDPGGSSSIPITITSGAGPVVIPLLDIPPAPGFGNSTTGPSSGFFNSGTGSSSGFGNVGANNSGFWNTAFAGIGNSG
LQNFGSLQSGWANLGNTVSGFYNTSAADFATPANLSGLSNVGADLTGVLRGPNGSTFNAGLANLGQFNVGSANLGSANLGSANLG
SANLGNSNVGFGNIGNANIGGANIGDFNVGIANTGPGLTAAVNNIGIGNTGNYNIGVGNTGNYNIGFGNTGNNNIGIGLSGDNQI
GFGPLNAGIANMGLFNLGDNNFGMANAGNFNQGIANTGNNNIGLFNTGNNNVGIGLTGDGLSGFSSLNSGAGNTGFFNSGTANTG
LFNSGTGNTGLFNSGTGNVGIGNMGTGGFGVGLSGDSQVGIGGTNSGSFNIGLFNSGTGNVGIGNSGTGNVGIGNTGTGNTGIGN
SGNYNTGLLNAGLVNTGIANPGNHNTGLFNIGTFNTGIANPGHYNTGSYNTGSYNTGMANAGDYGTGAFITGSMNNGLLWRADRQ
GLLAANYTITIERPAAFLNVDIPVNIPITGDITNVSIPAITFPRIDASGSVDIGILSGTVLAPVGPITLHGGDASAPLDTPIEID
FGPSPAINLNIGKPDGSTVINIVGGAGAGPISIPIIDLRPAPGFFNATTGPSSGFLNWGAGSASGLLNFGNNSGLYNFATSSMGN
SGFQNYGSLQSGWANLGNSISGIYNTGLGAPANVSGLLNIGTNLAGWLQNGPTETTFSVGLANLGFWNLGSANIGNYNLGSANIG
VYNLGSANIGDFNLGSANIGDFNLGSANIGSSNIGFGNVGPGLTAAIGNIGFGNTGNGNIGIGNTGTGNIGFGNTGNGNIGIGLT
GDTMTGFGGWNSGTGNIGLFNSGTGNIGFGNSGTGNWGIGNSGDYNTGIGNTGSTNSGFFNTGLVNTGIGNSGDYNTGLFNAGNT
NTGSFNPGDYNTGGFNPGNYNTGYFNPGNSNTGIANSGDVNTGAFNSGNYSNGFFWRGDYQGLGGFAYQSAVSEIPWSYDRFQH"

CDS complement(467459..468001)
/gene="PPE9"
/locus_tag="Rv0388c"
/note="Rv0388c, (MTV036.23c), len: 180 aa. PPE9, Member of the Mycobacterium tuberculosis PPE family, highly similar to others e.g. MTCY10G2_10|Z92539 from Mycobacterium tuberculosis (391 aa), FASTA scores: opt: 667, E(): 0,(58.3% identity in 180 aa overlap) but much shorter."
/codon_start=1
/transl_table=11
/product="PPE family protein PPE9"
/protein_id="YP_177724.1"
/db_xref="GI:57116729 GeneID:886439"
/translation="MDFGALPPEINSARIYSGPGSRPLMQAAAAWQRLANELTATAASYSSVISGLTGDDWLGPSALSMAAAAVP
YVAWMRATAASAEQAAAQAVAAANAYESAYAATVPPTVIAANRRTMLSLVQTNVFGQNTPAIATSETHYGEMWAHDILAMDGYAG
ASGAASQLRRSPATGDHQRGRVAE"

CDS complement(530751..532214)
/gene="PPE10"
/locus_tag="Rv0442c"
/experiment="EXISTENCE: identified in proteomics study"
/note="Rv0442c, (MTV037.06c), len: 487 aa. PPE10, Member of the Mycobacterium tuberculosis PPE family, nearly identical to hypothetical protein from Mycobacterium tuberculosis (strain Erdman) and to AN5S46909_1 protein fragment from Mycobacterium bovis (302 aa); P42611|YHS6_MYCTU hypothetical 50.6 kDa protein (517 aa), FASTA scores: opt: 3144, E(): 0, (98.4 identity in 492 aa overlap); and S46909|S46909_1 (302 aa), FASTA scores: opt: 1897, E(): 0,(98.0% identity in 302 aa overlap). Nucleotide position 532097 in the genome sequence has been corrected, T:C resulting in K40E."
/codon_start=1
/transl_table=11
/product="PPE family protein PPE10"
/protein_id="YP_177726.2"
/db_xref="GI:448824744 GeneID:886340"
/translation="MTSPHFAWLPPEINSALMFAGPGSGPLIAAATAWGELAEELLASIASLGSVTSELTSGAWLGPSAAAMMAV
ATQYLAWLSTAAAQAEQAAAQAMAIATAFEAALAATVQPAVVAANRGLMQLLAATNWFGQNAPALMDVEAAYEQMWALDVAAMAG

```
                     YHFDASAAVAQLAPWQQVLRNLGIDIGKNGQINLGFGNTGSGNIGNNNIGNNNIGSGNTGTGNIGSGNTGSGNLGLGNLGDGNIG
                     FGNTGSGNIGFGITGDHQMGFGGFNSGSGNIGFGNSGTGNVGLFNSGSGNIGIGNSGSLNSGIGTSGTINAGLGSAGSLNTSFWN
                     AGMQNAALGSAAGSEAALVSSAGYATGGMSTAALSSGILASALGSTGGLQHGLANVLNSGLTNTPVAAPASAPVGGLDSGNPNPG
                     SGSAAAGSGANPGLRSPGTSYPSFVNSGSNDSGLRNTAVREPSTPGSGIPKSNFYPSPDRESAYASPRIGQPVGSE"
     CDS             543174..544730
                     /gene="PPE11"
                     /locus_tag="Rv0453"
                     /experiment="EXISTENCE: identified in proteomics study"
                     /note="Rv0453, (MTV037.17), len: 518 aa. PPE11, Member of the Mycobacterium
                     tuberculosis PPE family, similar to many e.g. AL0212|MTV012_32 from Mycobacterium
                     tuberculosis (434 aa), FASTA scores: opt: 882, E(): 7e-31, (41.8% identity in 514 aa
                     overlap)."
                     /codon_start=1
                     /transl_table=11
                     /product="PPE family protein PPE11"
                     /protein_id="YP_177727.1"
                     /db_xref="GI:57116732 GeneID:886317"
                     /translation="MTSALIWMASPPEVHSALLSSGPGPGPVLAAATGWSSLGREYAAVAEELGALLAAVQAGVWQGPSAESFAA
                     ACLPYLSWLTQASADCAAAAARLEAVTAAYAAALVAMPTLAELAANHATHGAMVATNFFGINTIPIAVNEADYVRMWLQAATTMA
                     TYQAVADSAVRSIPDSVPPPRILKSNAQSQHSSSNNSGGADPVDDFIAEILKIITGGRVIWDPEAGTVNGLPYDAYTNPGTLMWW
                     IARSLELLQDFQEFAKLLFTNPVKAFQFLVDLILFDWPTHMLQLATWLAENPQLLVAALTPAISGLGAVSGLAGLTGLVPQPPVV
                     PAPAPDAVVPTVLPLAGTATPTTAPASAPAAGAAPGPPAGTATATSASVPTSAGGFPPYLVGSGPGIDFDAGTPAGSRRAQPAAD
                     NVTAVAAAQVSARHQARRRRRAAAKERGNADEFVDMDSGPAIPPSGERDAWASNSGVGGLGFAGTASNETVAAPAGLTTLADDEF
                     QCGPRMPMLPGAWDLGTWDRGD"
     CDS             complement(848103..850040)
                     /gene="PPE12"
                     /locus_tag="Rv0755c"
                     /experiment="EXISTENCE: identified in proteomics study"
                     /note="Rv0755c, (MTV041.29), len: 645 aa. PPE12, Member of the Mycobacterium
                     tuberculosis PPE family, highly similar to others e.g. Z82098|MTCY3C7_23 from
                     Mycobacterium tuberculosis (582 aa), FASTA scores: (56.1% identity in 636 aa
                     overlap); Z92774|MTCY6G11_5 from Mycobacterium tuberculosis (552 aa), FASTA scores:
                     (55.8% identity in 590 aa overlap); etc. Predicted to be an outer membrane protein
                     (See Song et al., 2008)."
                     /codon_start=1
                     /transl_table=11
                     /product="PPE family protein PPE12"
                     /protein_id="YP_177753.1"
                     /db_xref="GI:57116777 GeneID:888708"
                     /translation="MVGFAWLPPETNSLRMYLGAGSRPLLAAAGAWDGLAEELHAAASSFGSVTSELAGGAWQGPASAAMANAAG
                     PYASWLTAAGAQAELAARQARAAAGAFEEALAGVVHPAVVQANRVRTWLLAVSNVFGQNAPAIAAMESTYEQMWAQDVAVMAGYH
                     AASSAAAAQLASWQPALPNINLGVGNIGNLNVGNGNTGDYNLGNGNLGNANFGGGNGSAFHGQISSFNVGSGNIGNFNLGSGNGN
                     VGIGPSSFNVGSGNIGNANVGGGNSGDNNFGFGNFGNANIGIGNAGPNMSSPAVPTPGNGNVGIGNGGNGNFGGGNTGNANIGLG
                     NVGDGNVGFGNSGSYNFGFGNTGNNNIGIGLTGSNQIGFGGLNSGSGNIGFGNSGTGNIGFFNSGSGNFGVGNSGVTNTGVANSG
                     NINTGFGNSGFINTGFGNALSVNTGFGNSGQANTGIGNAGDFNTGNFNGGIINTGSFNSGAFNSGSFNGGDANSGFLNSGLTNTG
                     FANSGNINTGGFNAGNLNTGFGNTTDGLGENSGFGNAGSGNSGFNNSGRGNSGAQNVGNLQISGFANSGQSVTGYNNSVSVTSGF
                     GNKGTGLFSGFMSGFGNTGFLQSGFGNLEANPDNNSATSGFGNSGKQDSGGFNSIDFVSGFFHR"
     CDS             complement(976872..978203)
                     /gene="PPE13"
                     /locus_tag="Rv0878c"
                     /note="Rv0878c, (MTCY31.06c), len: 443 aa. PPE13, Member of the Mycobacterium
                     tuberculosis PPE family, highly similar to many e.g. P4261|YHS6_MYCTU (517 aa), FASTA
                     scores: opt: 1044, E(): 0, (47.4% identity in 397 aa overlap); MTV014_3,MTCI65_2,
                     MTCY98_24, MTCY3C7_23, MTCY48_17, MTV004_5,MTV004_3, etc. Predicted to be an outer
                     membrane protein (See Song et al., 2008)."
                     /codon_start=1
                     /transl_table=11
                     /product="PPE family protein PPE13"
                     /protein_id="YP_177764.1"
                     /db_xref="GI:57116794 GeneID:885617"
                     /translation="MNFMVLPPEVNSARIYAGAGPAPMLAAAVAWDGLAAELGMAAASFSLLISGLTAGPGSAWQGPAAAAMAAA
                     AAPYLSWLNAATARAEGAAAGAKAAAAVYEAARAATAHPALVAANRNQLLSLVLSNLFGQNLPAIAATEASYEQLWAQDVAAMVG
                     YHGGASTVASQLTPWQQLLSVLPPVVTAAPAGAVGVPAALAIPALGVENIGVGNFLGIGNIGNNNVGSGNTGDYNFGIGNIGNAN
                     LGNGNIGNANLGSGNAGFFNFGNGNDGNTNFGSGNAGFLNIGSGNEGSGNLGFGNAGDDNTGWGNSGDTNTGGFNSGDLNTGIGS
                     PVTQGVANSGFGNTGTGHSGFFNSGNSGSGFQNLGNGSSGFGNASDTSSGFQNAGTALTRASSTWADSPRAWPIRAPSRLQVWRT
                     RATTARECSIRVIISRVSSTGAPPQKKVGNSG"
     CDS             complement(1020058..1021329)
                     /gene="PPE14"
                     /locus_tag="Rv0915c"
```

```
                     /gene_synonym="MTB41"
                     /inference="protein motif:PROSITE:PS00626"
                     /note="Rv0915c, (MTCY21C12.09c), len: 423 aa. PPE14 (alternate gene name: MTB41).
                     Member of the Mycobacterium tuberculosis PPE family (see citation below), highly
                     similar to many e.g. Rv1807 from Mycobacterium tuberculosis (403 aa), FASTA scores:
                     opt: 966, E(): 4.4e-30, (45.7% identity in 392 aa overlap); etc. Contains PS00626
                     Regulator of chromosome condensation (RCC1) signature 2."
                     /codon_start=1
                     /transl_table=11
                     /product="PPE family protein PPE14"
                     /protein_id="YP_177765.1"
                     /db_xref="GI:57116795 GeneID:885069"
                     /translation="MDFGLLPPEVNSSRMYSGPGPESMLAAAAAWDGVAAELTSAAVSYGSVVSTLIVEPWMGPAAAAMAAAATP
                     YVGWLAATAALAKETATQARAAAEAFGTAFAMTVPPSLVAANRSRLMSLVAANILGQNSAAIAATQAEYAEMWAQDAAVMYSYEG
                     ASAAASALPPFTPPVQGTGPAGPAAAAAATQAAGAGAVADAQATLAQLPPGILSDILSALAANADPLTSGLLGIASTLNPQVGSA
                     QPIVIPTPIGELDVIALYIASIATGSIALAITNTARPWHIGLYGNAGGLGPTQGHPLSSATDEPEPHWGPFGGAAPVSAGVGHAA
                     LVGALSVPHSWTTAAPEIQLAVQATPTFSSSAGADPTALNGMPAGLLSGMALASLAARGTTGGGGTRSGTSTDGQEDGRKPPVVV
                     IREQPPPGNPPR"
     CDS             complement(1161297..1162472)
                     /gene="PPE15"
                     /locus_tag="Rv1039c"
                     /experiment="EXISTENCE: identified in proteomics study"
                     /note="Rv1039c, (MTCY10G2.10), len: 391 aa. PPE15, Member of the Mycobacterium
                     tuberculosis PPE family of glycine-rich proteins, most similar to
                     Rv2768c|AL008967|MTV002_33 Mycobacterium tuberculosis H37Rv (394 aa), FASTA scores:
                     opt: 1721, E(): 0, (70.4% identity in 398 aa overlap)."
                     /codon_start=1
                     /transl_table=11
                     /product="PPE family protein PPE15"
                     /protein_id="YP_177778.1"
                     /db_xref="GI:57116813 GeneID:888477"
                     /translation="MDFGALPPEINSARMYAGAGAGPMMAAGAAWNGLAAELGTTAASYESVITRLTTESWMGPASMAMVAAAQP
                     YLAWLTYTAEAAAHAGSQAMASAAAYEAAYAMTVPPEVVAANRALLAALVATNVLGINTPAIMATEALYAEMWAQDALAMYGYAA
                     ASGAAGMLQPLSPPSQTTNPGGLAAQSAAVGSAAATAAVNQVSVADLISSLPNAVSGLASPVTSVLDSTGLSGIIADIDALLATP
                     FVANIINSAVNTAAWYVNAAIPTAIFLANALNSGAPVAIAEGAIEAAEGAASAAAAGLADSVTPAGLGASLGEATLVGRLSVPAA
                     WSTAAPATTAGATALEGSGWTVAAEEAGPVTGMMPGMASAAKGTGAYAGPRYGFKPTVMPKQVVV"
     CDS             complement(1262272..1264128)
                     /gene="PPE16"
                     /locus_tag="Rv1135c"
                     /note="Rv1135c, (MTCI65.02c), len: 618 aa. PPE16, Member of the Mycobacterium
                     tuberculosis PPE family of glycine-rich proteins. Similar to Rv2356c (59.6% identity
                     in 627 aa overlap); etc.. Predicted to be an outer membrane protein (See Song et al.,
                     2008)."
                     /codon_start=1
                     /transl_table=11
                     /product="PPE family protein PPE16"
                     /protein_id="YP_177790.1"
                     /db_xref="GI:57116833 GeneID:885131"
                     /translation="MSFLVLPPEVNSALMFAGAGSGPTLAAAAAWDGLAAELGQAANSFSSATAALADTAWQGPAATAMAAAAAP
                     YASWLSTAATRALSAAAQAKAAAAVYEAARAATVDPLLVAANRHQLVSLVLSNLFGQNAPAIAATEAAYEQLWAADVAAMVSYHS
                     GASAVAAQLAPWAQAVRALPNPTAPALASGPAALAIPALGIGNTGIGNIFSIGNIGDYNLGNGNTGNANLGSGNTGQANLGSGNT
                     GFFNFGSGNTANTNFGSGNLGNLNLGSGNDGNGNFGLGNIGDGNRGSGNVGSFNFGTANAGSFNVGSANHGSPNVGFANLGNNNL
                     GIANLGNNNLGIANLGNNNIGIGLTGDNMIGIGALNSGIGNLGFGNSGNNNIGLFNSGNNNIGFFNSGDSNFGFFNSGDTNTGFG
                     NAGFTNTGFGNAGSGNFGFGNAGNNNFGFGNSGFENMGVGNSGAYNTGSFNSGTLNTGDLNSGDFNTGWANSGDINTGGFHSGDL
                     NTGFGSPVDQPVMNSGFGNIGTGNSGFNNSGDANSGFQNTNTGAFFIGHSGLLNSGGGQHVGISNSGTGFNTGLFNTGFNNTGIG
                     NSATNAAFTTTSGVANSGDNSSGGFNAGNDQSGFFDG"
     CDS             complement(1298764..1299804)
                     /gene="PPE17"
                     /locus_tag="Rv1168c"
                     /note="Rv1168c, (MTV005.04c), len: 346 aa. PPE17, Member of the Mycobacterium
                     tuberculosis PPE family of glycine-rich proteins, similar to many e.g.
                     E332789|Z98268|MTCI125.27C (385 aa), FASTA scores: opt: 504, E(): 0, (36.6% identity
                     in 388 aa overlap)."
                     /codon_start=1
                     /transl_table=11
                     /product="PPE family protein PPE17"
                     /protein_id="YP_177791.1"
                     /db_xref="GI:57116836 GeneID:885990"
                     /translation="MDFTIFPPEFNSLNIQGSARPFLVAANAWKNLSNELSYAASRFESEINGLITSWRGPSSTIMAAAVAPFRA
```

WIVTTASLAELVADHISVVAGAYEAAHAAHVPLPVIETNRLTRLALATTNIFGIHTPAIFALDALYAQYWSQDGEAMNLYATMAA
AAARLTPFSPPAPIANPGALARLYELIGSVSETVGSFAAPATKNLPSKLWTLLTKGTYPLTAARISSIPVEYVLAFVEGSNMGQM
MGNLAMRSLTPTLKGPLELLPNAVRPAVSATLGNADTIGGLSVPPSWVADKSITPLAKAVPTSAPGGPSGTSWAQLGLASLAGGA
VGAVAARTRSGVILRSPAAG"

CDS 1339349..1340524
/gene="PPE18"
/locus_tag="Rv1196"
/gene_synonym="mtb39a"
/experiment="EXISTENCE: identified in proteomics study"
/note="Rv1196, (MTCI364.08), len: 391 aa. PPE18 (alternate gene name: mtb39a). Member of the Mycobacterium tuberculosis PPE family of glycine-rich proteins, highly similar to others e.g. Y07P_MYCTU|Q11031 hypothetical 40.0 kDa protein cy02b10.25c (396 aa), FASTA scores: opt: 2124,E(): 0, (85.1% identity in 397 aa overlap). Note that expression of Rv1196 was demonstrated in lysates by immunodetection (see Dillon et al., 1999)."
/codon_start=1
/transl_table=11
/product="PPE family protein PPE18"
/protein_id="YP_177795.1"
/db_xref="GI:57116841 GeneID:886073"
/translation="MVDFGALPPEINSARMYAGPGSASLVAAAQMWDSVASDLFSAASAFQSVVWGLTVGSWIGSSAGLMVAAAS
PYVAWMSVTAGQAELTAAQVRVAAAAYETAYGLTVPPPVIAENRAELMILIATNLLGQNTPAIAVNEAEYGEMWAQDAAAMFGYA
AATATATATLLPFEEAPEMTSAGGLLEQAAAVEEASDTAAANQLMNNVPQALQQLAQPTQGTTPSSKLGGLWKTVSPHRSPISNM
VSMANNHMSMTNSGVSMTNTLSSMLKGFAPAAAAQAVQTAAQNGVRAMSSLGSSLGSSGLGGGVAANLGRAASVGSLSVPQAWAA
ANQAVTPAARALPLTSLTSAAERGPGQMLGGLPVGQMGARAGGGLSGVLRVPPRPYVMPHSPAAG"

CDS complement(1532443..1533633)
/gene="PPE19"
/locus_tag="Rv1361c"
/gene_synonym="mtb39b"
/experiment="EXISTENCE: identified in proteomics study"
/inference="protein motif:PROSITE:PS00501"
/note="Rv1361c, (MTCY02B10.25c), len: 396 aa. PPE19 (alternate gene name: mtb39b). Member of the Mycobacterium tuberculosis PPE family of glycine-rich proteins, highly similar to many e.g. Rv1196|MTCI364.08|PPE18, FASTA scores: E(): 0, (84.9% identity in 397 aa overlap); MTCY274.23c (42.3% identity in 416 aa overlap); etc. Contains PS00501 Signal peptidases I serine active site. Note that expression of Rv1361c was demonstrated in lysates by immunodetection (see Dillon et al., 1999). A core mycobacterial gene; conserved in mycobacterial strains (See Marmiesse et al., 2004)."
/codon_start=1
/transl_table=11
/product="PPE family protein PPE19"
/protein_id="YP_177801.1"
/db_xref="GI:57116852 GeneID:886819"
/translation="MVDFGALPPEINSARMYAGPGSASLVAAAKMWDSVASDLFSAASAFQSVVWGLTTGSWIGSSAGLMVAAAS
PYVAWMSVTAGQAELTAAQVRVAAAAYETAYGLTVPPPVIAENRAELMILIATNLLGQNTPAIAVNEAEYGEMWAQDAAAMFGYA
ATAATATEALLPFEDAPLITNPGGLLEQAVAVEEAIDTAAANQLMNNVPQALQQLAQPTKSIWPFDQLSELWKAISPHLSPLSNI
VSMLNNHVSMTNSGVSMASTLHSMLKGFAPAAAAQAVETAAQNGVQAMSSLGSQLGSSLGSSGLGAGVAANLGRAASVGSLSVPQA
WAAANQAVTPAARALPLTSLTSAAQTAPGHMLGGLPLGQLTNSGGGFGGVSNALRMPPRAYVMPRVPAAG"

CDS 1561769..1563388
/gene="PPE20"
/locus_tag="Rv1387"
/experiment="EXISTENCE: identified in proteomics study"
/inference="protein motif:PROSITE:PS00343"
/note="Rv1387, (MTCY21B4.04), len: 539 aa. PPE20, Member of Mycobacterium tuberculosis PPE family of proteins, similar to many e.g. Y05F_MYCTU|Q10892 hypothetical 46.9 kd protein cy251.15 (463 aa), FASTA scores: E(): 4.2e-26, (37.7% identity in 531 aa overlap); similar also to MTCY274.23c (37.5% identity in 168 aa overlap). Contains PS00343 Gram-positive cocci surface proteins 'anchoring' hexapeptide. A core mycobacterial gene; conserved in mycobacterial strains (See Marmiesse et al., 2004)."
/codon_start=1
/transl_table=11
/product="PPE family protein PPE20"
/protein_id="YP_177806.1"
/db_xref="GI:57116858 GeneID:886784"
/translation="MTEPWIAFPPEVHSAMLNYGAGVGPMLISATQNGELSAQYAEAASEVEELLGVVASEGWQGQAAEAFVAAY
MPFLAWLIQASADCVEMAAQQHVVIEAYTAAVELMPTQVELAANQIKLAVLVATNFFGINTIPIAINEAEYVEMWVRAATTMATY
STVSRSALSAMPHTSPPPLILKSDELLPDTGEDSDEDGHNHGGHSHGGHARMIDNFFAEILRGVSAGRIVWDPVNGTLNGLDYDD
YVYPGHAIWWLARGLEFFQDGEQFGELLFTNPTGAFQFLLYVVVVDLPTHIAQIATWLGQYPQLLSAALTGVIAHLGAITGLAGL

```
                     SGLSAIPSAAIPAVVPELTPVAAAPPMLAVAGVGPAVAAPGMLPASAPAPAAAAGATAAGPTPPATGFGGFPPYLVGGGGPGIGF
                     GSGQSAHAKAAASDSAAAESAAQASARAQARAARRGRSAAKARGHRDEFVTMDMGFDAAAPAPEHQPGARASDCGAGPIGFAGTV
                     RKEAVVKAAGLTTLAGDDFGGGPTMPMMPGTWTHDQGVFDEHR"
     CDS             complement(1751297..1753333)
                     /gene="PPE21"
                     /locus_tag="Rv1548c"
                     /experiment="EXISTENCE: identified in proteomics study"
                     /note="Rv1548c, (MTCY48.17), len: 678 aa. PPE21, Member of the Mycobacterium
                     tuberculosis PPE family, similar to several e.g. YHS6_MYCTU|P42611 hypothetical 50.6
                     kDa protein in hsp65 3' region (517 aa), FASTA scores: opt:1142, E(): 0, (40.6%
                     identity in 616 aa overlap); also similar to MTCY31.06c (54.9% identity in 381 aa
                     overlap). Predicted to be an outer membrane protein (See Song et al.,2008)."
                     /codon_start=1
                     /transl_table=11
                     /product="PPE family protein PPE21"
                     /protein_id="YP_177817.1"
                     /db_xref="GI:57116882 GeneID:886384"
                     /translation="MNFSVLPPEINSALMFAGAGPGPMLAAASAWTGLAGDLGSAAASFSAVTSQLATGSWQGPASAAMTGVAAS
                     YARWLTTAAAQAEQAAGQAQAAVSAFEAALAATVHPGAVSANRGRLRSLVASNLLGQNAPAIAAVEAVYEQMWAADVAAMLGYHG
                     EASAVALSLTPFTPSPSAAATPGGAVIIAGFPFLDLGNVTIGGFNLASGNLGLGNLGSFNPGSANTGSVNLGNANIGDLNLGSGN
                     IGSYNLGGGNTGDLNPDSGNTGTLNWGSGNIGSYNLGGGNLGSYNLGSGNTGDTNFGGGNTGNLNVGGGNTGNSNFGFGNTGNVN
                     FGNGNTGDTNFGSGNLGSGNIGFGNKGSHNIGFGNSGNNNIGFGLTGDNQIGFGALNSGSGNLGFGNSGNGNIGFFNSGNNNIGM
                     GNSGNGVGALSVEFGSSAERSSGFGNSGELSTGIGNSGQLSTGWFNSATTSTGWFNSGTTNTGWFNSGTTNTGIGNSGGNLVTGS
                     MGLFNSGHTNTGSFNAGSMNTGDFNSGNVNTGYFNSGNINTGFFNSGDLNTGLFNSVNQPVQNSGWLHTGTNNSGYANAGTFNSG
                     FDNNARDEHAEFVTGNSGLANVGNYNAGIINVGDHLSGFRNSVPTITGTANISGFVNAGTSISGFFNFGSLMSGFANFDDEVSGY
                     LNGDSRASGWIH"
     CDS             complement(1931497..1932654)
                     /gene="PPE22"
                     /locus_tag="Rv1705c"
                     /experiment="EXISTENCE: identified in proteomics study"
                     /note="Rv1705c, (MTCI125.27c), len: 385 aa. PPE22, Member of the Mycobacterium
                     tuberculosis PPE family of glycine-rich proteins, similar to many e.g.
                     YX23_MYCTU|Q10813 hypothetical 41.1 kDa protein cy274.2 3 (404 aa), fasta scores:
                     opt: 819, E(): 0, (46.2% identity in 413 aa overlap)."
                     /codon_start=1
                     /transl_table=11
                     /product="PPE family protein PPE22"
                     /protein_id="YP_177827.1"
                     /db_xref="GI:57116898 GeneID:885068"
                     /translation="MDFGALPPEVNSGRMYCGPGSAPMVAAASAWNGLAAELSVAAVGYERVITTLQTEEWLGPASTLMVEAVAP
                     YVAWMRATAIQAEQAASQARAAAAAYETAFAAIVPPPLIAANRARLTSLVTHNVFGQNTASIAATEAQYAEMWAQDAMAMYGYAG
                     SSATATKVTPFAPPPNTTSPSAAATQLSAVAKAAGTSAGAAQSAIAELIAHLPNTLLGLTSPLSSALTAAATPGWLEWFINWYLP
                     ISQLFYNTVGLPYFAIGIGNSLITSWRALGWIGPEAAEAAAAAPAAVGAAVGGTGPVSAGLGNAATIGKLSLPPNWAGASPSLAP
                     TVGSASAPLVSDIVEQPEAGAAGNLLGGMPLAGSGTGTGGAGPRYGFRVTVMSRPPFAG"
     CDS             complement(1932694..1933878)
                     /gene="PPE23"
                     /locus_tag="Rv1706c"
                     /experiment="EXISTENCE: identified in proteomics study"
                     /note="Rv1706c, (MTCI125.28c), len: 394 aa. PPE23, Member of the Mycobacterium
                     tuberculosis PPE family of glycine-rich proteins, similar to many e.g.
                     YX23_MYCTU|Q10813 hypothetical 41.1 kDa protein cy274.23 (404 aa), fasta scores: opt:
                     841, E(): 3.9e-31, (46.8% identity in 408 aa overlap)."
                     /codon_start=1
                     /transl_table=11
                     /product="PPE family protein PPE23"
                     /protein_id="YP_177828.1"
                     /db_xref="GI:57116899 GeneID:885070"
                     /translation="MTLDVPVNQGHVPPGSVACCLVGVTAVADGIAGHSLSNFGALPPEINSGRMYSGPGSGPLMAAAAAWDGLA
                     AELSSAATGYGAAISELTNMRWWSGPASDSMVAAVLPFVGWLSTTATLAEQAAMQARAAAAAFEAAFAMTVPPPAIAANRTLLMT
                     LVDTNWFGQNTPAIATTESQYAEMWAQDAAAMYGYASAAAPATVLTPFAPPPQTTNATGLVGHATAVAALRGQHSWAAAIPWSDI
                     QKYWMMFLGALATAEGFIYDSGGLTLNALQFVGGMLWSTALAEAGAAEAAAGAGGAAGWSAWSQLGAGPVAASATLAAKIGPMSV
                     PPGWSAPPATPQAQTVARSIPGIRSAAEAAETSVLLRGAPTPGRSRAAHMGRRYGRRLTVMADRPNVG"
     CDS             complement(1981614..1984775)
                     /gene="PPE24"
                     /locus_tag="Rv1753c"
                     /experiment="EXISTENCE: identified in proteomics study"
                     /note="Rv1753c, (MTCY28.16c), len: 1053 aa. PPE24, Member of the Mycobacterium
                     tuberculosis PPE family of Gly-,Asn-rich proteins, similar to many e.g.
                     YF48_MYCTU|Q10778 hypothetical protein cy48.17 (678 aa), FASTA scores: opt: 1360,
```

E(): 0, (48.9% identity in 550 aa overlap). Note that the Gly-, Asn-rich sequence is interrupted by six near-perfect 26 aa repeats, a unique region, and another,more degenerate region of five 25 aa repeats before resuming at the C-terminus. The end of the first Gly-, Asn-rich region and the start of the first set of repeats shows some similarity to Q50577|AT10S from Mycobacterium tuberculosis (170 aa) (40.2% identity in 189 aa overlap)."
/codon_start=1
/transl_table=11
/product="PPE family protein PPE24"
/protein_id="YP_177830.1"
/db_xref="GI:57116904 GeneID:885544"
/translation="MNFSVLPPEINSALIFAGAGPEPMAAAATAWDGLAMELASAAASFGSVTSGLVGGAWQGASSSAMAAAAAP
YAAWLAAAAVQAEQTAAQAAAMIAEFEAVKTAVVQPMLVAANRADLVSLVMSNLFGQNAPAIAAIEATYEQMWAADVSAMSAYHA
GASAIASALSPFSKPLQNLAGLPAWLASGAPAAAMTAAAGIPALAGGPTAINLGIANVGGGNVGNANNGLANIGNANLGNYNFGS
GNFGNSNIGSASLGNNNIGFGNLGSNNVGVGNLGNLNTGFANTGLGNFGFGNTGNNNIGIGLTGNNQIGIGGLNSGTGNFGLFNS
GSGNVGFFNSGNGNFGIGNSGNFNTGGWNSGHGNTGFFNAGSFNTGMLDVGNANTGSLNTGSYNMGDFNPGSSNTGTFNTGNANT
GFLNAGNINTGVFNIGHMNNGLFNTGDMNNGVFYRGVGQGSLQFSITTPDLTLPPLQIPGISVPAFSLPAITLPSLNIPAATTPA
NITVGAFSLPGLTLPSLNIPAATTPANITVGAFSLPGLTLPSLNIPAATTPANITVGAFSLPGLTLPSLNIPAATTPANITVGAF
SLPGLTLPSLNIPAATTPANITVGAFSLPGLTLPSLNIPAATTPANITVSGFQLPPLSIPSVAIPPVTVPPITVGAFNLPPLQIP
EVTIPQLTIPAGITIGGFSLPAIHTQPITVGQIGVGQFGLPSIGWDVFLSTPRITVPAFGIPFTLQFQTNVPALQPPGGGLSTFT
NGALIFGEFDLPQLVVHPYTLTGPIVIGSFFLPAFNIPGIDVPAINVDGFTLPQITTPAITTPEFAIPPIGVGGFTLPQITTQEI
ITPELTINSIGVGGFTLPQITTPPITTPPLTIDPINLTGFTLPQITTPPITTPPLTIDPINLTGFTLPQITTPPITTPPLTIEPI
GVGGFTTPPLTVPGIHLPSTTIGAFAIPGGPGYFNSSTAPSSGFFNSGAGGNSGFGNNGSGLSGWFNTNPAGLLGGSGYQNFGGL
SSGFSNLGSGVSGFANRGILPFSVASVVSGFANIGTNLAGFFQGTTS"

CDS 2025301..2026398
/gene="PPE25"
/locus_tag="Rv1787"
/note="Rv1787, (MTV049.09), len: 365 aa. PPE25, Member of the Mycobacterium tuberculosis PPE family of glycine-rich proteins, similar to Z74024|MTCY274.24 Mycobacterium tuberculosis cosmid (404 aa), FASTA scores: opt: 837, E(): 0, (52.0% identity in 406 aa overlap)."
/codon_start=1
/transl_table=11
/product="PPE family protein PPE25"
/protein_id="YP_177833.1"
/db_xref="GI:57116909 GeneID:885827"
/translation="MDFGALPPEINSGRMYCGPGSGPMLAAAAAWDGVAVELGLAATGYASVIAELTGAPWVGAASLSMVAAATP
YVAWLSQAAARAEQAGMQAAAAAAAYEAAFVMTVPPPVITANRVLVMTLIATNFFGQNSAAIAVAEAQYAEMWAQDAVAMYGYAA
ASASASRLIPFAAPPKTTNSAGVVAQVAAVAAMPGLLQRLSSAASVSWSNPNDWWLVRLLGSITPTERTTIVRLLGQSYFATGMA
QFFASIAQQLTFGPGGTTAGSGGAWYPTPQFAGLGASRAVSASLARANKIGALSVPPSWVKTTALTESPVAHAVSANPTVGSSHG
PHGLLRGLPLGSRITRRSGAFAHRYGFRHSVVARPPSAG"

CDS 2026790..2027971
/gene="PPE26"
/locus_tag="Rv1789"
/experiment="EXISTENCE: identified in proteomics study"
/note="Rv1789, (MTV049.11), len: 393 aa. PPE26, Member of the Mycobacterium tuberculosis PPE family of glycine-rich proteins, highly similar to others e.g.Z98268|MTCI125.26 Mycobacterium tuberculosis cosmid (385 aa), FASTA score: opt: 1283, E(): 0, (62.7% identity in 408 aa overlap)."
/codon_start=1
/transl_table=11
/product="PPE family protein PPE26"
/protein_id="YP_177835.1"
/db_xref="GI:57116911 GeneID:885333"
/translation="MDFGALPPEVNSVRMYAGPGSAPMVAAASAWNGLAAELSSAATGYETVITQLSSEGWLGPASAAMAEAVAP
YVAWMSAAAAQAEQAATQARAAAAAFEAAFAATVPPPLIAANRASLMQLISTNVFGQNTSAIAAAEAQYGEMWAQDSAAMYAYAG
SSASASAVTPFSTPPQIANPTAQGTQAAAVATAAGTAQSTLTEMITGLPNALQSLTSPLLQSSNGPLSWLWQILFGTPNFPTSIS
ALLTDLQPYASFFYNTEGLPYFSIGMGNNFIQSAKTLGLIGSAAPAAVAAAGDAAKGLPGLGGMLGGGPVAAGLGNAASVGKLSV
PPVWSGPLPGSVTPGAAPLPVSTVSAAPEAAPGSLLGGLPLAGAGGAGAGPRYGFRPTVMARPPFAG"

CDS 2028425..2029477
/gene="PPE27"
/locus_tag="Rv1790"
/experiment="EXISTENCE: identified in proteomics study"
/note="Rv1790, (MTV049.12), len: 350 aa. PPE27, Member of the Mycobacterium tuberculosis PPE family of glycine-rich protein, similar to Z74024|MTCY274.24 Mycobacterium tuberculosis cosmid (404 aa), FASTA scores: opt: 849, E(): 0, (50.0% identity in 406 aa overlap)."
/codon_start=1
/transl_table=11

```
                     /product="PPE family protein PPE27"
                     /protein_id="YP_177836.1"
                     /db_xref="GI:57116912 GeneID:885859"
                     /translation="MDFGALPPEINSGRMYCGPGSGPMLAAAAAWDGVAVELGLAATGYASVIAELTGAPWVGAASLSMVAAATP
                     YVAWLSQAAARAEQAGMQAAAAAAAYEAAFVMTVPPPVITANRVLVMTLIATNFFGQNSAAIAVAEAQYAEMWAQDAVAMYGYAA
                     ASASASASRLIPFAAPPKTTNSAGVVAQAVASVSWPNPNDWWLVRLLGSITPTERTTIVRLLGQSYLATGMARFLTSIAQQLTFGPG
                     GTTAGSGGAWYPTPQFAGLGAGPAVSASLARAEPVGRLSVPPSWAVAAPAFAEKPEAGTPMSVIGEASSCGQGGLLRGIPLARAG
                     RRTGAFAHRYGFRHSVITRSPSAG"
     CDS             2039453..2041420
                     /gene="PPE28"
                     /locus_tag="Rv1800"
                     /note="Rv1800, (MTV049.22), len: 655 aa. PPE28, Member of the Mycobacterium
                     tuberculosis PPE family of glycine-rich proteins, C-terminal very similar to parts of
                     PE proteins e.g. Z92770|MTCI5.25|Rv0151c (588 aa), FASTA scores: opt: 1269, E(): 0,
                     (41.5% identity in 591 aa overlap). Predicted to be an outer membrane protein (See
                     Song et al., 2008)."
                     /codon_start=1
                     /transl_table=11
                     /product="PPE family protein PPE28"
                     /protein_id="YP_177839.1"
                     /db_xref="GI:57116915 GeneID:885465"
                     /translation="MLPNFAVLPPEVNSARVFAGAGSAPMLAAAAAWDDLASELHCAAMSFGSVTSGLVVGWWQGSASAAMVDAA
                     ASYIGWLSTSAAHAEGAAGLARAAVSVFEEALAATVHPAMVAANRAQVASLVASNLFGQNAPAIAALESLYECMWAQDAAAMAGY
                     YVGASAVATQLASWLQRLQSIPGAASLDARLPSSAEAPMGVVRAVNSAIAANAAAAQTVGLVMGGSGTPIPSARYVELANALYMS
                     GSVPGVIAQALFTPQGLYPVVVIKNLTFDSSVAQGAVILESAIRQQIAAGNNVTVFGYSQSATISSLVMANLAASADPPSPDELS
                     FTLIGNPNNPNGGVATRFPGISFPSLGVTATGATPHNLYPTKIYTIEYDGVADFPRYPLNFVSTLNAIAGTYYVHSNYFILTPEQ
                     IDAAVPLTNTVGPTMTQYYIIRTENLPLLEPLRSVPIVGNPLANLVQPNLKVIVNLGYGDPAYGYSTSPPNVATPFGLFPEVSPV
                     VIADALVAGTQQGIGDFAYDVSHLELPLPADGSTMPSTAPGSGTPVPPLSIDSLIDDLQVANRNLANTISKVAATSYATVLPTAD
                     IANAALTIVPSYNIHLFLEGIQQALKGDPMGLVNAVGYPLAADVALFTAAGGLQLLIIISAGRTIANDISAIVP"
     CDS             2042001..2043272
                     /gene="PPE29"
                     /locus_tag="Rv1801"
                     /note="Rv1801, (MTV049.23), len: 423 aa. PPE29, Member of the Mycobacterium
                     tuberculosis PPE family of glycine-rich proteins, most similar to
                     AL022021|MTV049.29|Rv1808 (409 aa), FASTA scores: opt: 1229, E(): 0, (55.2% identity
                     in 422 aa overlap)."
                     /codon_start=1
                     /transl_table=11
                     /product="PPE family protein PPE29"
                     /protein_id="YP_177840.1"
                     /db_xref="GI:57116916 GeneID:885491"
                     /translation="MDFGLLPPEINSGRMYTGPGPGPMLAAATAWDGLAVELHATAAGYASELSALTGAWSGPSSTSMASAAAPY
                     VAWMSATAVHAELAGAQARLAIAAYEAAFAATVPPPVIAANRAQLMVLIATNIFGQNTPAIMMTEAQYMEMWAQDAAAMYGYAGS
                     SATASRMTAFTEPPQTTNHGQLGAQSSAVAQTAATAAGGNLQSAFPQLLSAVPRALQGLALPTASQSASATPQWVTDLGNLSTFL
                     GGAVTGPYTFPGVLPPSGVPYLLGIQSVLVTQNGQGVSALLGKIGGKPITGALAPLAEFALHTPILGSEGLGGGSVSAGIGRAGL
                     VGKLSVPQGWTVAAPEIPSPAAALQATRLAAAPIAATDGAGALLGGMALSGLAGRAAAGSTGHPIGSAAAPAVGAAAAAVEDLAT
                     EANIFVIPAMDD"
     CDS             2043384..2044775
                     /gene="PPE30"
                     /locus_tag="Rv1802"
                     /experiment="EXISTENCE: identified in proteomics study"
                     /note="Rv1802, (MTV049.24), len: 463 aa. PPE30, Member of the Mycobacterium
                     tuberculosis PPE family of glycine-rich proteins, most similar to
                     AL022021|MTV049.30|Rv1809 (468 aa), FASTA scores: opt: 1238, E(): 0, (51.0% identity
                     in 471 aa overlap)."
                     /codon_start=1
                     /transl_table=11
                     /product="PPE family protein PPE30"
                     /protein_id="YP_177841.1"
                     /db_xref="GI:57116917 GeneID:885542"
                     /translation="MDFGVLPPEINSGRMYAGPGSGPMLAAAAAWDGLATELQSTAADYGSVISVLTGVWSGQSSGTMAAAAAPY
                     VAWMSATAALAREAAAQASAAAAAYEAAFAATVPPPVVAANRAELAVLAATNIFGQNTGAIAAAEARYAEMWAQDAAAMYGYAGS
                     SSVATQVTPFAAPPPTTNAAGLATQGVAVAQAVGASAGNARSLVSEVLEFLATAGTNYNKTVASLMNAVTGVPYASSVYNSMLGL
                     GFAESKMVLPANDTVISTIFGMVQFQKFFNPVTPFNPDLIPKSALGAGLGLRSAISSGLGSTAPAISAGASQAGSVGGMSVPPSW
                     AAATPAIRTVAAVFSSTGLQAVPAAAISEGSLLSQMALASVAGGALGGAAARATGGFLGGGRVTAVKKSLKDSDSPDKLRRVVAH
                     MMEKPESVQHWHTDEDGLDDLLAELKKKPGIHAVHMAGGNKAEIAPTISESG"
     CDS             2048398..2049597
                     /gene="PPE31"
                     /locus_tag="Rv1807"
```

```
                     /experiment="EXISTENCE: identified in proteomics study"
                     /note="Rv1807, (MTV049.29), len: 399 aa. PPE31, Member of the Mycobacterium
                     tuberculosis PPE family of glycine-rich proteins, most similar to
                     Rv1789|MTV049.11|AL022021 (393 aa), FASTA scores: opt: 1169, E(): 0, (49.5% identity
                     in 412 aa overlap)."
                     /codon_start=1
                     /transl_table=11
                     /product="PPE family protein PPE31"
                     /protein_id="YP_177653.1"
                     /db_xref="GI:57116920 GeneID:885072"
                     /translation="LDFATLPPEINSARMYSGAGSAPMLAAASAWHGLSAELRASALSYSSVLSTLTGEEWHGPASASMTAAAAP
                     YVAWMSVTAVRAEQAGAQAEAAAAAYEAAFAATVPPPVIEANRAQLMALIATNVLGQNAPAIAATEAQYAEMWSQDAMAMYGYAG
                     ASAAATQLTPFTEPVQTTNASGLAAQSAAIAHATGASAGAQQTTLSQLIAAIPSVLQGLSSSTAATFASGPSGLLGIVGSGSSWL
                     DKLWALLDPNSNFWNTIASSGLFLPSNTIAPFLGLLGGVAAADAAGDVLGEATSGGLGGALVAPLGSAGGLGGTVAAGLGNAATV
                     GTLSVPPSWTAAAPLASPLGSALGGTPMVAPPPAVAAGMPGMPFGTMGGQGFGRAVPQYGFRPNFVARPPAAG"
     CDS             2049921..2051150
                     /gene="PPE32"
                     /locus_tag="Rv1808"
                     /experiment="EXISTENCE: identified in proteomics study"
                     /inference="protein motif:PROSITE:PS00343"
                     /note="Rv1808, (MTV049.30), len: 409 aa. PPE32, Member of the Mycobacterium
                     tuberculosis PPE family of glycine-rich proteins, most similar to
                     Rv1800|MTV049.22|AL022021 (655 aa), FASTA scores: opt: 1225, E(): 0, (55.1% identity
                     in 423 aa overlap). Contains PS00343 Gram-positive cocci surface proteins 'anchoring'
                     hexapeptide. Nucleotide position 2050913 in the genome sequence has been
                     corrected,A:G resulting in E331E."
                     /codon_start=1
                     /transl_table=11
                     /product="PPE family protein PPE32"
                     /protein_id="YP_177844.1"
                     /db_xref="GI:57116921 GeneID:885590"
                     /translation="MDFGALPPEINSGRMYAGPGSGPLLAAAAAWDALAAELYSAAASYGSTIEGLTVAPWMGPSSITMAAAVAP
                     YVAWISVTAGQAEQAGAQAKIAAGVYETAFAATVPPPVIEANRALLMSLVATNIFGQNTPAIAATEAHYAEMWAQDAAAMYGYAG
                     SSATASQLAPFSEPPQTTNPSATAAQSAVVAQAAGAAASSDITAQLSQLISLLPSTLQSLATTATATSASAGWDTVLQSITTILA
                     NLTGPYSIIGLGAIPGGWWLTFGQILGLAQNAPGVAALLGPKAAAGALSPLAPLRGGYIGDITPLGGGATGGIARAIYVGSLSVP
                     QGWAEAAPVMRAVASVLPGTGAAPALAAEAPGALFGEMALSSLAGRALAGTAVRSGAGAARVAGGSVTEDVASTTTIIVIPAD"
     CDS             2051282..2052688
                     /gene="PPE33"
                     /locus_tag="Rv1809"
                     /experiment="EXISTENCE: identified in proteomics study"
                     /note="Rv1809, (MTV049.31), len: 468 aa. PPE33, Member of the Mycobacterium
                     tuberculosis PPE family of glycine-rich proteins, most similar to
                     RV1802AL022021|MTV049.23 (463 aa), FASTA scores: opt: 1238, E(): 0, (51.2% identity
                     in 471 aa overlap). Alternative nucleotide at position 2051746 (T->C; A155A) has been
                     observed."
                     /codon_start=1
                     /transl_table=11
                     /product="PPE family protein PPE33"
                     /protein_id="YP_177845.1"
                     /db_xref="GI:57116922 GeneID:885555"
                     /translation="MDFGLQPPEITSGEMYLGPGAGPMLAAAVAWDGLAAELQSMAASYASIVEGMASESWLGPSSAGMAAAAAP
                     YVTWMSGTSAQAKAAADQARAAVVAYETAFAAVVPPPQIAANRSQLISLVATNIFGQNTAAIAATEAEYGEMWAQDTMAMFGYAS
                     SSATASRLTPFTAPPQTTNPSGLAGQAAATGQATALASGTNAVTTALSSAAAQFPFDIIPTLLQGLATLSTQYTQLMGQLINAIF
                     GPTGATTYQNVFVTAANVTKFSTWANDAMSAPNLGMTEFKVFWQPPPAPEIPKSSLGAGLGLRSGLSAGLAHAASAGLGQANLVG
                     DLSVPPSWASATPAVRLVANTLPATSLAAAPATQIPANLLGQMALGSMTGGALGAAAPAIYTGSGARARANGGTPSAEPVKLEAV
                     IAQLQKQPDAVRHWNVDKADLDGLLDRLSKQPGIHAVHVSNGDKPKVALPDTQLGSH"
     CDS             complement(2162932..2167311)
                     /gene="PPE34"
                     /locus_tag="Rv1917c"
                     /experiment="EXISTENCE: identified in proteomics study"
                     /inference="protein motif:PROSITE:PS00879"
                     /note="Rv1917c, (MTV050.01c-MTCY180.01), len: 1459 aa. PPE34, Member of the
                     Mycobacterium tuberculosis PPE family of glycine-rich proteins, MPTR subfamily (see
                     citation below). Similar to MTCY28.16, MTCY13E10.17, MTCY63.10,MTV004.05, MTCY98.24,
                     MTCY6G11.05, etc. C-terminus is identical to Q50471. Unknown Mycobacterium
                     tuberculosis protein (693 aa), FASTA results: opt: 2635, E(): 0, (99.7% identity in
                     391 aa overlap). Start changed since original submission (+23 aa). Thougth to be
                     surface exposed,cell-wall associated."
                     /codon_start=1
```

```
                     /transl_table=11
                     /product="PPE family protein PPE34"
                     /protein_id="YP_177655.1"
                     /db_xref="GI:57116928 GeneID:885362"
                     /translation="MNFSTLPPEINSALIFGGAGSEPMSAAAVAWDQLAMELASAAASFNSVTSGLVGESWLGPSSAAMAAAVAP
                     YLGWLAAAAAQAQRSATQAAALVAEFEAVRAAMVQPALVAANRSDLVSLVFSNFFGQNAPAIAAIEAAYEQMWAIDVSVMSAYHA
                     GASAVASALTPFTAPPQNLTDLPAQLAAAPAAVVTAAITSSKGVLANLSLGLANSGFGQMGAANLGILNLGSLNPGGNNFGLGNV
                     GSNNVGLGNTGNGNIGFGNTGNGNIGFGLTGDNQQGFGGWNSGTGNIGLFNSGTGNIGIGNTGTGNFGIGNSGTSYNTGIGNTGQ
                     ANTGFFNAGIANTGIGNTGNYNTGSFNLGSFNTGDFNTGSSNTGFFNPGNLNTGVGNTGNVNTGGFNSGNYSNGFFWRGDYQGLI
                     GFSGTLTIPAAGLDLNGLGSVGPITIPSITIPEIGLGINSSGALVGPINVPPITVPAIGLGINSTGALVGPINIPPITLNSIGLE
                     LSAFQVINVGSISIPASPLAIGLFGVNPTVGSIGPGSISIQLGTPEIPAIPPFFPGFPPDYVTVSGQIGPITFLSGGYSLPAIPL
                     GIDVGGGLGPFTVFPDGYSLPAIPLGIDVGGGLGPFTVFPDGYSLPAIPLGIDVGGGLGPFTVFPDGYSLPAIPLGIDVGGAIGP
                     LTTPPITIPSIPLGIDVSGSLGPINIPIEIAGTPGFGNSTTTPSSGFFNSGTGGTSGFGNVGSGGSGFWNIAGNLGNSGFLNVGP
                     LTSGILNFGNTVSGLYNTSTLGLATSAFHSGVGNTDSQLAGFMRNAAGGTLFNFGFANDGTLNLGNANLGDYNVGSGNVGSYNFG
                     SGNIGNGSFGFGNIGSNNFGFGNVGSNNLGFANTGPGLTEALHNIGFGNIGGNNYGFANIGNGNIGFGNTGTGNIGIGLTGDNQV
                     GFGALNSGSGNIGFFNSGNGNIGFFNSGNGNVGIGNSGNYNTGLGNVGNANTGLFNTGNVNTGIGNAGSYNTGSYNAGDTNTGDL
                     NPGNANTGYLNLGDLNTGWGNIGDLNTGALISGSYSNGILWRGDYQGLIGYSDTLSIPAIPLSVEVNGGIGPIVVPDITIPGIPL
                     SLNALGGVGPIVVPDITIPGIPLSLNALGGVGPIVVPDITIPGIPLSLNALGGVGPIVVPDITIPGIPLSLNALGGVGPIVVPDI
                     TIPGIPLSLNALGGVGPITVPGVPISRIPLTINIRIPVNITLNELPFNVAGIFTGYIGPIPLSTFVLGVTLAGGTLESGIQGFSV
                     NPFGLNIPLSGATNAVTIPGFAINPFGLNVPLSGGTSPVTIPGFAINPFGLNVPLSGGTSPVTIPGFTIPGSPLNLTANGGLGPI
                     NIPINITSAPGFGNSTTTPSSGFFNSGDGSASGFGNVGPGISGLWNQVPNALQGGVSGIYNVGQLASGVANLGNTVSGFNNTSTV
                     GHLTAAFNSGVNNIGQMLLGFFSPGAGP"
     CDS             complement(2167649..2170612)
                     /gene="PPE35"
                     /locus_tag="Rv1918c"
                     /experiment="EXISTENCE: identified in proteomics study"
                     /note="Rv1918c, (MTV050.02c), len: 987 aa. PPE35, Member of the Mycobacterium
                     tuberculosis PPE family of glycine-rich proteins. Similar to MTCY28.16|Z95890
                     Mycobacterium tuberculosis cosmid (1053 aa), FASTA scores: opt: 3404,E(): 0, (65.6%
                     identity in 1058 aa overlap). Also similar to MTV004.05, MTY13E10.17, MTV014.03,
                     MTCY3C7.23,MTCY6G11.05, MTCY48.17, MTV004.03, MTCY31.07, MTCY4C12.36,MTCY180.01, etc."
                     /codon_start=1
                     /transl_table=11
                     /product="PPE family protein PPE35"
                     /protein_id="YP_177850.1"
                     /db_xref="GI:57116929 GeneID:885506"
                     /translation="MHYSVLPPEINSALIFAGAGSGPMLAAASAWDGLATELASAAVSFGSVTAGLVGGSWQGRSSVAMAAAAAP
                     YAGWLAAAATQAEQAATQAQVMVAEFEAVRLAMVQPALVAANRSGLISLVISNLFGQNAPAIAAAEAAYEEMWALDVSAMAAYHS
                     GASAVAVALPAFALPLRLPAGLAAGPAAVVTALTTAVGMPTFAGRAIAASLGLANVGGGNLGNANNGLGNIGNANLGNNNLGSGN
                     FGSFNIGSANLGGNNIGIGNAGANNFGLANLGNLNTGFANAGIGNFGIANTGNNNIGNGLTGNNQIGIGGLNSGNGNVGLFNAGS
                     ANIGFFNSGNGNFGIGNSGNFSTGLFNPGHGNTGFLNAGSFNTGMFDVGNANTGSFNVGHYNFGAFNPGPSNTGTFNTGGANTGW
                     FNTGSINTGAFNIGDMNNGLFNTGDMNNGVFYRGVGQGSLQFAITSPDLTLPSLEIPGISVPAFSLPAITLPSLTIPAVTTPANV
                     TVGAFDLPGLTVPSLTIPAAMTPANITVGAFDLPGLTVPSLTIPATTTPANITVGAFNLPQLSIPSVTVPPITIPAGTALGAFNL
                     PTLSIPSVTVPPITIPAGTTVGGFTLPTIHTPLISTPQISIGGFSTPGIATQANSGVINLPTFSLNGITITNLVVFIPNNITALQ
                     TNMPGVFPQIGGFANTPPAFINTGTITVGGGQINGVGFSIGAINVTPFTLPNVVIQPWSLGGISVDGFTLPEISTQEFTTPALTI
                     SPIGVGALSLPDITTQQFTTPELTIDPITLGGFTLPQLSIPAITTPAFTIDPIALGGFTLPQIMTPEITTPPFAIDPIGLSGFTL
                     PQVNIPEITTPEFTIQPVGLAAFTTPALTIASIHLPSTTMGGFAIPAGPGYFNSSATPSLGFFNAGIGGNSGFGNSGSGLSGWFN
                     TSPVGLLAGSGYQNYGGLISGFSNLGSGISGFANTGTLPFAVTSLVSGLANIGNNLSGLFFQSTTP"
     CDS             2367711..2368442
                     /gene="PPE36"
                     /locus_tag="Rv2108"
                     /note="Rv2108, (MTCY261.04), len: 243 aa. PPE36, Member of the Mycobacterium
                     tuberculosis PE family: N-terminus is similar to N-terminal region of Mycobacterium
                     tuberculosis PPE family proteins. A core mycobacterial gene; conserved in
                     mycobacterial strains (See Marmiesse et al., 2004)."
                     /codon_start=1
                     /transl_table=11
                     /product="PPE family protein PPE36"
                     /protein_id="YP_177859.1"
                     /db_xref="GI:57116946 GeneID:887814"
                     /translation="MPNFWALPPEINSTRIYLGPGSGPILAAAQGWNALASELEKTKVGLQSALDTLLESYRGQSSQALIQQTLP
                     YVQWLTTTAEHAHKTAIQLTAAANAYEQARAAMVPPAMVRANRVQTTVLKAINWFGQFSTRIADKEADYEQMWFQDALVMENYWE
                     AVQEAIQSTSHFEDPPEMADDYDEAWMLNTVFDYHNENAKEEVIHLVPDVNKERGPIELVTKVDKEGTIRLVYDGEPTFSYKEHP
                     KF"
     CDS             2381071..2382492
                     /gene="PPE37"
                     /locus_tag="Rv2123"
                     /gene_synonym="irg2"
                     /note="Rv2123, (MTCY261.19), len: 473 aa. PPE37 (alternate gene name: irg2), member
```

of the Mycobacterium tuberculosis PPE family of proteins but the C-terminus is not repetitive (see citation below)."
/codon_start=1
/transl_table=11
/product="PPE family protein PPE37"
/protein_id="YP_177861.1"
/db_xref="GI:57116948 GeneID:888710"
/translation="MTFPMWFAVPPEVPSAWLSTGMGPGPLLAAARAWHALAAQYTEIATELASVLAAVQASSWQGPSADRFVVA
HQPFRYWLTHAATVATAAAAAHETAAAGYTSALGGMPTLAELAANHAMHGALVTTNFFGVNTIPIALNEADYLRMWIQAATVMSH
YQAVAHESVAATPSTPPAPQIVTSAASSAASSSFPDPTKLILQLLKDFLELLRYLAVELLPGPLGDLIAQVLDWFISFVSGPVFT
FLAYLVLDPLIYFGPFAPLTSPVLLPAGLTGLAGLGAVSGPAGPMVERVHSDGPSRQSWPAATGVTLVGTNPAALVTTPAPAPTT
SAAPTAPSTPGSSAAQGLYAVGGPDGEGFNPIAKTTALAGVTTDAAAPAAKLPGDQAQSSASKATRLRRRLRQHRFEFLADDGRL
TMPNTPEMADVAAGNRGLDALGFAGTIPKSAPGSATGLTHLGGGFADVLSQPMLPHTWDGSD"
CDS complement(2632923..2634098)
/gene="PPE38"
/locus_tag="Rv2352c"
/experiment="EXISTENCE: identified in proteomics study"
/note="Rv2352c, (MTCY98.21c), len: 391 aa. PPE38, Member of Mycobacterium tuberculosis PPE_family, highly similar to many e.g. Q10778|MTCY48.17|Y04H_MYCTU (734 aa), FASTA scores: opt: 713, E(): 2.8e-27, (37.7% identity in 430 aa overlap); Q10540|MTCY31.06c, Q11031|MTCY02B10.25c,Q10813|MTCY274.23c, P42611|MTV037.06C, P71868|MTCY03C7.23,P95248|MTCY98.22c, P71869|MTCY03C7.24c, etc."
/codon_start=1
/transl_table=11
/product="PPE family protein PPE38"
/protein_id="YP_177870.1"
/db_xref="GI:57116974 GeneID:888959"
/translation="MILDFSWLPPEINSARIYAGAGSGPLFMAAAAWEGLAADLRASASSFDAVIAGLAAGPWSGPASVAMAGAA
APYVGWLSAAAGQAELSAGQATAAATAFEAALAATVHPAAVTANRVLLGALVATNILGQNTPAIAATEFDYVEMWAQDVGAMVGY
HAGAAAVAETLTPFSVPPLDLAGLASQAGAQLTGMATSVSAALSPIAEGAVEGVPAVVAAAQSVAAGLPVDAALQVGQAAAYPAS
MLIGPMMQLAQMGTTANTAGLAGAEAAGLAAADVPTFAGDIASGTGLGGAGGLGAGMSAELGKARLVGAMSVPPTWEGSVPARMA
SSAMAGLGAMPAEVPAAGGPMGMMPMPMGMGGAGAGMPAGMMGRGGANPHVVQARPSVVPRVGIG"
CDS complement(2634528..2635592)
/gene="PPE39"
/locus_tag="Rv2353c"
/note="Rv2353c, (MTCY98.22c), len: 354 aa. PPE39, Member of Mycobacterium tuberculosis PPE family, highly similar to many e.g. near ORF P95249|Rv2356c|MTCY98.25 from Mycobacterium tuberculosis (615 aa), FASTA scores: opt: 1566, E(): 3.2e-69, (66.1% identity in 349 aa overlap); Q10778|MTCY48.17, Q10540|MTCY31.06c, E241779|MTCY98,Q10813|MTCY274.23c, P71868|MTCY03C7.23,P71869|MTCY03C7.24c, P42611|MTV037.06C, E64997|MTCY98,Q10707|MTCY49.38C, P71657|MTCY02B10.25c, etc. Note that the ATG and RBS appear to be provided by the IR of neighbouring IS6110. Predicted to be an outer membrane protein (See Song et al., 2008)."
/codon_start=1
/transl_table=11
/product="PPE family protein PPE39"
/protein_id="YP_177871.1"
/db_xref="GI:57116975 GeneID:886003"
/translation="MPGRFRNFGSQNLGSGNIGSTNVGSGNIGSTNVGSGNIGDTNFGNGNNGNFNFGSGNTGSNNIGFGNTGSG
NFGFGNTGNNNIGIGLTGDGQIGIGGLNSGSGNIGFGNSGTGNVGLFNSGTGNVGFGNSGTANTGFGNAGNVNTGFWNGGSTNTG
LANAGAGNTGFFDAGNYNFGSLNAGNINSSFGNSGDGNSGFLNAGDVNSGVGNAGDVNTGLGNSGNINTGGFNPGTLNTGFFSAM
TQAGPNSGFFNAGTGNSGFGHNDPAGSGNSGIQNSGFGNSGYVNTSTTSMFGGNSGVLNTGYGNSGFYNAAVNNTGIFVTGVMSS
GFFNFGTGNSGLLVSGNGLSGFFKNLFG"
CDS complement(2637688..2639535)
/gene="PPE40"
/locus_tag="Rv2356c"
/experiment="EXISTENCE: identified in proteomics study"
/note="Rv2356c, (MTCY98.25), len: 615 aa. PPE40, Member of Mycobacterium tuberculosis PPE_family, highly similar to others e.g. Q10778|MTCY48.17|YF48_MYCTU hypothetical PPE-family protein (678 aa), FASTA scores: opt: 1888, E(): 1.9e-78, (54.4% identity in 667 aa overlap); Q10540|MTCY31.06c, E241779|MTCY98, P42611|MTV037.06c,Q10813|MTCY274.23c, P71657|MTCY02B10.25c, MTCY03C7.23,P71869|MTCY03C7.24c, etc. Predicted to be an outer membrane protein (See Song et al., 2008)."
/codon_start=1
/transl_table=11
/product="PPE family protein PPE40"
/protein_id="YP_177872.1"

```
                     /db_xref="GI:57116976 GeneID:888950"
                     /translation="MVNFSVLPPEINSGRMFFGAGSGPMLAAAAAWDGLAAELGLAAESFGLVTSGLAGGSGQAWQGAAAAAMVV
                     AAAPYAGWLAAAAARAGGAAVQAKAVAGAFEAARAAMVDPVVVAANRSAFVQLVLSNVFGQNAPAIAAAEATYEQMWAADVAAMV
                     GYHGGASAAAAALAPWQQAVPGLSGLLGGAANAPAAAAQGAAQGLAELTLNLGVGNIGSLNLGSGNIGGTNVGSGNVGGTNLGSG
                     NYGSLNWGSGNTGTGNAGSGNTGDYNPGSGNFGSGNFGSGNIGSLNVGSGNFGTLNLANGNNGDVNFGGGNTGDFNFGGGNNGTL
                     NFGFGNTGSGNFGFGNTGNNNIGIGLTGDGQIGIGGLNSGTGNIGFGNSGNNNIGFFNSGDGNIGFFNSGDGNTGFGNAGNINTG
                     FWNAGNLNTGFGSAGNGNVGIFDGGNSNSGSFNVGFQNTGFGNSGAGNTGFFNAGDSNTGFANAGNVNTGFFNGGDINTGGFNGG
                     NVNTGFGSALTQAGANSGFGNLGTGNSGWGNSDPSGTGNSGFFNTGNGNSGFSNAGPAMLPGFNSGFANIGSFNAGIANSGNNLA
                     GISNSGDDSSGAVNSGSQNSGAFNAGVGLSGFFR"
     CDS             complement(2727336..2727920)
                     /gene="PPE41"
                     /locus_tag="Rv2430c"
                     /experiment="EXISTENCE: identified in proteomics study"
                     /note="Rv2430c, (MTCY428.16), len: 194 aa. PPE41, Member of the Mycobacterium
                     tuberculosis PPE family similar to others e.g. AAK46014|Rv1745|MT1745 from
                     Mycobacterium tuberculosis (385 aa) FASTA scores: opt: 389, E(): 1.2e-17, (35.95%
                     identity in 192 aa overlap); etc."
                     /codon_start=1
                     /transl_table=11
                     /product="PPE family protein PPE41"
                     /protein_id="YP_177881.1"
                     /db_xref="GI:57116988 GeneID:885945"
                     /translation="MHFEAYPPEVNSANIYAGPGPDSMLAAARAWRSLDVEMTAVQRSFNRTLLSLMDAWAGPVVMQLMEAAKPF
                     VRWLTDLCVQLSEVERQIHEIVRAYEWAHHDMVPLAQIYNNRAERQILIDNNALGQFTAQIADLDQEYDDFWDEDGEVMRDYRLR
                     VSDALSKLTPWKAPPPIAHSTVLVAPVSPSTASSRTDT"
     CDS             2935046..2936788
                     /gene="PPE42"
                     /locus_tag="Rv2608"
                     /note="Rv2608, (MTCY01A10.25c), len: 580 aa. PPE42, Member of the Mycobacterium
                     tuberculosis PPE family, highly similar to many e.g. O06828|Rv1430|MTCY493.24c from
                     Mycobacterium tuberculosis (528 aa), FASTA scores: opt: 1004, E(): 5.9e-48, (56.05%
                     identity in 307 aa overlap)."
                     /codon_start=1
                     /transl_table=11
                     /product="PPE family protein PPE42"
                     /protein_id="YP_177893.1"
                     /db_xref="GI:57117005 GeneID:888204"
                     /translation="MNFAVLPPEVNSARIFAGAGLGPMLAAASAWDGLAEELHAAAGSFASVTTGLAGDAWHGPASLAMTRAASP
                     YVGWLNTAAGQAAQAAGQARLAASAFEATLAATVSPAMVAANRTRLASLVAANLLGQNAPAIAAAEAEYEQIWAQDVAAMFGYHS
                     AASAVATQLAPIQEGLQQQLQNVLAQLASGNLGSGNVGVGNIGNDNIGNANIGFGNRGDANIGIGNIGDRNLGIGNTGNWNIGIG
                     ITGNGQIGFGKPANPDVLVVGNGGPGVTALVMGGTDSLLPLPNIPLLEYAARFITPVHPGYTATFLETPSQFFPFTGLNSLTYDV
                     SVAQGVTNLHTAIMAQLAAGNEVVVFGTSQSATIATFEMRYLQSLPAHLRPGLDELSFTLTGNPNRPDGGILTRFGFSIPQLGFT
                     LSGATPADAYPTVDYAFQYDGVNDFPKYPLNVFATANAIAGILFLHSGLIALPPDLASGVVQPVSSPDVLTTYILLPSQDLPLLV
                     PLRAIPLLGNPLADLIQPDLRVLVELGYDRTAHQDVPSPFGLFPDVDWAEVAADLQQGAVQGVNDALSGLGLPPPWQPALPRLF"
     CDS             complement(3076894..3078078)
                     /gene="PPE43"
                     /locus_tag="Rv2768c"
                     /experiment="EXISTENCE: identified in proteomics study"
                     /note="Rv2768c, (MTV002.33c), len: 394 aa. PPE43, Member of the Mycobacterium
                     tuberculosis PPE family, highly similar to many e.g. upstream ORF
                     O33312|Rv2770c|MTV002.35c (402 aa), FASTA scores: opt: 1135, E(): 6.1e-51, (62.15%
                     identity in 391 aa overlap); and P96362|Rv1039c|MTCY10G2.10 from M. tuberculosis (391
                     aa), FASTA scores: opt: 1721,E(): 6.8e-81, (70.35% identity in 398 aa overlap).
                     Equivalent to AAK47157 from Mycobacterium tuberculosis strain CDC1551 (462 aa) but
                     shorter 68 aa."
                     /codon_start=1
                     /transl_table=11
                     /product="PPE family protein PPE43"
                     /protein_id="YP_177906.1"
                     /db_xref="GI:57117022 GeneID:887765"
                     /translation="MDFGALPPEINSTRMYAGAGAAPLMAAGATWNGLAVELSTTASSVESVIMQLTTEQWLGPASMSMVVAAQP
                     YLAWLTYTAESAAHAAAQAMASAAAFEAAFAMTVPPAEVAANRALLAALVATNVLGQNTPAIMATEAHYGEMWAQDALAMYGYAA
                     SSAAAGRLNPLITPSQTANMAGLAGQAAAVSHAAAASTVQQVGLGSLISNLPNAVMGFASPLTSAADAAGLGGIIQDIEELLGIT
                     FVQNAINGAVNTTAWFVMATIPNAVFLGHAFAALNPATVTAAADAVPAAAAAAGLAHTVTPVGVGGASLTASLGEASSVGGLSVP
                     AGWSTAAPAMTSGTTALEGSGWAVPEEAGPVAAMPGMAGISGAAKGAGAYAGPRYGFKPIVMPKQVVV"
     CDS             complement(3079309..3080457)
                     /gene="PPE44"
                     /locus_tag="Rv2770c"
                     /experiment="EXISTENCE: identified in proteomics study"
```

```
                     /note="Rv2770c, (MTV002.35c), len: 382 aa. PPE44, Member of the Mycobacterium
                     tuberculosis PPE family, highly similar to many e.g. downstream ORF
                     O33310|Rv2768c|MTV002.33c from M. tuberculosis (394 aa), FASTA scores: opt: 1135,
                     E(): 2.2e-53, (62.15% identity in 391 aa overlap); and P96362|Rv1039c|MTCY10G2.10
                     from Mycobacterium tuberculosis (391 aa), FASTA scores: opt: 1010, E(): 1e-46,
                     (55.95% identity in 395 aa overlap). Equivalent to AAK47159 from Mycobacterium
                     tuberculosis strain CDC1551 (402 aa) but shorter 20 aa. Start changed since first
                     submission (-20 aa)."
                     /codon_start=1
                     /transl_table=11
                     /product="PPE family protein PPE44"
                     /protein_id="YP_177677.1"
                     /db_xref="GI:57117024 GeneID:888456"
                     /translation="MDFGALPPEVNSARMYGGAGAADLLAAAAAWNGIAVEVSTAASSVGSVITRLSTEHWMGPASLSMAAAVQP
                     YLVWLTCTAESSALAAAQAMASAAAFETAFALTVPPAEVVANRALLAELTATNILGQNVSAIAATEARYGEMWAQDASAMYGYAA
                     ASAVAARLNPLTRPSHITNPAGLAHQAAAVGQAGASAFARQVGLSHLISDVADAVLSFASPVMSAADTGLEAVRQFLNLDVPLFV
                     ESAFHGLGGVADFATAAIGNMTLLADAMGTVGGAAPGGGAAAAVAHAVAPAGVGGTALTADLGNASVVGRLSVPASWSTAAPATA
                     AGAALDGTGWAVPEEDGPIAVMPPAPGMVVAANSVGADSGPRYGVKPIVMPKHGLF"
     CDS             complement(3200794..3202020)
                     /gene="PPE45"
                     /locus_tag="Rv2892c"
                     /experiment="EXISTENCE: identified in proteomics study"
                     /note="Rv2892c, (MTCY274.23c), len: 408 aa. PPE45, Member of the Mycobacterium
                     tuberculosis PPE family, highly similar to many e.g.
                     O06386|Rv3621c|MTCY15C10.31|MTCY07H7B. 01 from M. tuberculosis (413 aa), FASTA
                     scores: opt: 957, E(): 6.2e-46, (44.7% identity in 423 aa overlap)."
                     /codon_start=1
                     /transl_table=11
                     /product="PPE family protein PPE45"
                     /protein_id="YP_177913.1"
                     /db_xref="GI:57117034 GeneID:887824"
                     /translation="MDFGVLPPEINSGRMYAGPGSGPMMAAAAAWDSLAAELGLAAGGYRLAISELTGAYWAGPAAASMVAAVTP
                     YVAWLSATAGQAEQAGMQARAAAAAYELAFAMTVPPPVVVANRALLVALVATNFFGQNTPAIAATEAQYAEMWAQDAAAMYAYAG
                     SAAIATELTPFTAAPVTTSPAALAGQAAATVSSTVPPLATTAAVPQLLQQLSSTSLIPWYSALQQWLAENLLGLTPDNRMTIVRL
                     LGISYFDEGLLQFEASLAQQAIPGTPGGAGDSGSSVLDSWGPTIFAGPRASPSVAGGGAVGGVQTPQPYWYWALDRESIGGSVSA
                     ALGKGSSAGSLSVPPDWAARARWANPAAWRLPGDDVTALRGTAENALLRGFPMASAGQSTGGGFVHKYGFRLAVMQRPPFAG"
     CDS             complement(3376939..3378243)
                     /gene="PPE46"
                     /locus_tag="Rv3018c"
                     /note="Rv3018c, (MTV012.32c), len: 434 aa. PPE46, Member of PPE family but lacks Gly,
                     Ala rich repeats at C-terminal domain, closest to MTCY261.19. See citation below.
                     Also very similar to following ORF MTV012.35c. Nearly identical in parts to
                     Mycobacterium tuberculosis protein erroneously described as dihydrofolate reductase
                     (X59271|MTFOLA_1) P31500|DYR_MYCTU (214 aa), FASTA scores: opt: 972, E(): 4.4e-42,
                     (80.0% identity in 195 aa overlap); and Z97559|MTCY261_19 from Mycobacterium
                     tuberculosis cosmid (473 aa), FASTA scores: opt: 806, E(): 0; (38.8% identity in 479
                     aa overlap); and O53268|MTV012.35c from Mycobacterium tuberculosis (358 aa), FASTA
                     scores: opt: 1714, E(): 3.3e-79, (78.3% identity in 355 aa overlap)."
                     /codon_start=1
                     /transl_table=11
                     /product="PPE family protein PPE46"
                     /protein_id="YP_177918.1"
                     /db_xref="GI:57117045 GeneID:888940"
                     /translation="MTAPVWLASPPEVHSALLSAGPGPGSLQAAAAGWSALSAEYAAVAQELSVVVAAVGAGVWQGPSAELFVAA
                     YVPYVAWLVQASADSAAAAGEHEAAAAGYVCALAEMPTLPELAANHLTHAVLVATNFFGINTIPIALNEADYVRMWVQAATVMSA
                     YEAVVGAALVATPHTGPAPVIVKPGANEASNAVAAATITPFPWHEIVQFLEETFAAYDQYLSALLSELPAVAWVWFQLFVDILGF
                     NIIGFIITLASNAQLLTEFAINASYVAVGLLYAIAGVIDIVVEWVIGNLFGVVPLLGGPLLGALAAAVVPGVAGLAGVAGLAALP
                     AVGAAAGAPAALVGSVAPVSGGVVSPQARLVSAVEPAPASTSVSVLASDRGAGALGFVGTAGKESVGQPAGLTVLADEFGDGAPV
                     PMLPGSWGPDLVGVAGDGGLVSV"
     CDS             complement(3490476..3491651)
                     /gene="PPE49"
                     /locus_tag="Rv3125c"
                     /experiment="EXISTENCE: identified in proteomics study"
                     /note="Rv3125c, (MTCY164.35c), len: 391 aa. PPE49, Member of the Mycobacterium
                     tuberculosis PPE family, similar to other e.g. P95247|Rv2352c|MTCY98.21c (391 aa),
                     FASTA scores: opt: 1576, E(): 3.8e-72, (62.55% identity in 398 aa overlap),
                     MTCY98.0029c, MTCY03A2.22c, MTCY10G2.10,MTCY02B10.25c, MTCI364.08, M TCY21C12.09c,
                     MTCY48.17. This region is a possible MT-complex-specific genomic island (See Becq et
                     al., 2007)."
```

```
                     /codon_start=1
                     /transl_table=11
                     /product="PPE family protein PPE49"
                     /protein_id="YP_177932.1"
                     /db_xref="GI:57117062 GeneID:888892"
                     /translation="MVLGFSWLPPEINSARMFAGAGSGPLFAAASAWEGLAADLWASASSFESVLAALTTGPWTGPASMSMAAAA
                     SPYVGWLSTVASQAQLAAIQARAAATAFEAALAATVHPTAVTANRVSLASLIAANVLGQNTPAIAATEFDYLEMWAQDVAAMVGY
                     HAGAKSVAATLAPFSLPPVSLAGLAAQVGTQVAGMATTASAAVTPVVEGAMASVPTVMSGMQSLVSQLPLQHASMLFLPVRILTS
                     PITTLASMARESATRLGPPAGGLAAANTPNPSGAAIPAFKPLGGRELGAGMSAGLGQAQLVGSMSVPPTWQGSIPISMASSAMSG
                     LGVPPNPVALTQAAGAAGGGMPMMLMPMSISGAGAGMPGGLMDRDGAGWHVTQARLTVIPRTGVG"
     CDS             3501334..3501732
                     /gene="PPE50"
                     /locus_tag="Rv3135"
                     /note="Rv3135, (MTCY03A2.23c), len: 132 aa. PPE50, Member of the Mycobacterium
                     tuberculosis Ala-, Gly-rich PPE family, similar to P95190|Rv3136|MTCY03A2.22c (380
                     aa),FASTA scores: opt: 494, E(): 6.7e-25, (57.25% identity in 131 aa overlap) (next
                     ORF downstream), MTY21C12_9,MTCY3C7_24, MTCI125_27, MTV049_12, MTV049_9,
                     MTV049_11,MTCY274_24 etc."
                     /codon_start=1
                     /transl_table=11
                     /product="PPE family protein PPE50"
                     /protein_id="YP_177934.1"
                     /db_xref="GI:57117064 GeneID:888153"
                     /translation="MDYAFLPPEINSARMYSGPGPNSMLVAAASWDALAAELASAAENYGSVIARLTGMHWWGPASTSMLAMSAP
                     YVEWLERTAAQTKQTATQARAAAAAFEQAHAMTVPPALVTGIRGAIVVETASASNTAGTPP"
     CDS             3501794..3502936
                     /gene="PPE51"
                     /locus_tag="Rv3136"
                     /experiment="EXISTENCE: identified in proteomics study"
                     /note="Rv3136, (MTCY03A2.22c), len: 380 aa. PPE51, Member of the Mycobacterium
                     tuberculosis Ala-, Gly-rich PPE family, similar to Q9AGF0|Ov2770c Rv2770c-like
                     protein from M. microti (397 aa), FASTA scores: opt: 917, E(): 9e-41,(46.15% identity
                     in 388 aa overlap); O33312|Rv2770c|MTV002.35c, MTV002_36, MTCI125_26,MTCY10G2_10,
                     MTCI364_8, MTV049_28, MTV049_29, etc."
                     /codon_start=1
                     /transl_table=11
                     /product="PPE family protein PPE51"
                     /protein_id="YP_177935.1"
                     /db_xref="GI:57117065 GeneID:888835"
                     /translation="MDFALLPPEVNSARMYTGPGAGSLLAAAGGWDSLAAELATTAEAYGSVLSGLAALHWRGPAAESMAVTAAP
                     YIGWLYTTAEKTQQTAIQARAAALAFEQAYAMTLPPPVVAANRIQLLALIATNFFGQNTAAIAATEAQYAEMWAQDAAAMYGYAT
                     ASAAAALLTPFSPPRQTTNPAGLTAQAAAVSQATDPLSLLIETVTQALQALTIPSFIPEDFTFLDAIFAGYATVGVTQDVESFVA
                     GTIGAESNLGLLNVGDENPAEVTPGDFGIGELVSATSPGGGVSASGAGGAASVGNTVLASVGRANSIGQLSVPPSWAAPSTRPVS
                     ALSPAGLTTLPGTDVAEHGMPGVPGVPVAAGRASGVLPRYGVRLTVMAHPPAAG"
     CDS             complement(3510088..3511317)
                     /gene="PPE52"
                     /locus_tag="Rv3144c"
                     /experiment="EXISTENCE: identified in proteomics study"
                     /note="Rv3144c, (MTCY03A2.14), len: 409 aa. PPE52, Member of the Mycobacterium
                     tuberculosis PPE family, Gly-,Ala-rich, similar to others e.g.
                     P71868|Rv3533c|MTCY03C7.23 (582 aa), FASTA scores: opt: 1007, E(): 5.2e-35, (56.2%
                     identity in 306 aa overlap); and MTV014_3, MTCY6G11_5,MTCY98.0034c, MTCY31.06c,
                     MTCY48.17, MTCY98.0029c,MTCY03C7.17c, etc. Nucleotide position 3510642 in the genome
                     sequence has been corrected, T:C resulting in S226G."
                     /codon_start=1
                     /transl_table=11
                     /product="PPE family protein PPE52"
                     /protein_id="YP_177936.2"
                     /db_xref="GI:448824800 GeneID:887930"
                     /translation="MSFVVLPPEINSLRMFIGAGTAPMLAAAAAWDGLAEELGTAAQSFASVTAGLAGQAWQGPAALAMAAAAAP
                     YAGWLTAAAAQSAGAAGQARAVASIFEAAQAATVLPAAVAANRDAFVQLVMTNLFGQNAPLIAAAEGVYEEMWAADVAAMSGYYS
                     GASAIAAQVVPWASLLQRFPGLGAGATGATGGESVGTGATGGESVGTGGGESVGTGGATASGGGVGYVGGGVASAGLAAGDPAHG
                     SVGQGNFGGGDVGAGDVVASSATSAHAGVVSPGFIGAPLALAALGQMARGGTNSAPGTATESARAPEPAASAPPEAVVEVPELEV
                     PAMGVLPTVDPKVAAKAAPLSTTRVGQSAGSGIPESTLRTAQGQQASETSAAEETAPSLRPEAAAGQLRPRVRKDPKIQMRGG"
     CDS             complement(3527391..3529163)
                     /gene="PPE53"
                     /locus_tag="Rv3159c"
                     /experiment="EXISTENCE: identified in proteomics study"
                     /note="Rv3159c, (MTV014.03c), len: 590 aa. PPE53, Member of the Mycobacterium
```

```
                     tuberculosis PPE_family of Gly-, Asn-rich proteins. Highly similar to
                     P71868|Rv3533c|MTCY03C7.23 (582 aa), FASTA scores: opt: 2289, E(): 3.2e-98, (63.5%
                     identity in 600 aa overlap); and also similar to MTCY48_17,MTV041_29, MTCY6G11_5,
                     MTCY98_24, etc. Predicted to be an outer membrane protein (See Song et al., 2008)."
                     /codon_start=1
                     /transl_table=11
                     /product="PPE family protein PPE53"
                     /protein_id="YP_177937.1"
                     /db_xref="GI:57117067 GeneID:888794"
                     /translation="MNYSVLPPEINSLRMFTGAGSAPMLAASVAWDRLAAELAVAASSFGSVTSGLAGQSWQGAAAAAMAAAAAP
                     YAGWLAAAAARAAGASAQAKAVASAFEAARAATVHPMLVAANRNAFVQLVLSNLFGQNAPAIAAAEAMYEQMWAADVAAMVGYHG
                     GASAAAAQLSSWSIGLQQALPAAPSALAAAIGLGNIGVGNLGGGNTGDYNLGSGNSGNANVGSGNSGNANVGSGNDGATNLGSGN
                     IGNTNLGSGNVGNVNLGSGNRGFGNLGNGNFGSGNLGSGNTGSTNFGGGNLGSFNLGSGNIGSSNIGFGNNGDNNLGLGNNGNNN
                     IGFGLTGDNLVGIGALNSGIGNLGFGNSGNNNIGFFNSGNNNVGFFNSGNNNFGFGNAGDINTGFGNAGDTNTGFGNAGFFNMGI
                     GNAGNEDMGVGNGGSFNVGVGNAGNQSVGFGNAGTLNVGFANAGSINTGFANSGSINTGGFDSGDRNTGFGSSVDQSVSSSGFGN
                     TGMNSSGFFNTGNVSAGYGNNGDVQSGINNTNSGGFNVGFYNSGAGTVGIANSGLQTTGIANSGTLNTGVANTGDHSSGGFNQGS
                     DQSGFFGQP"
     CDS             complement(3729364..3736935)
                     /gene="PPE54"
                     /locus_tag="Rv3343c"
                     /experiment="EXISTENCE: identified in proteomics study"
                     /note="Rv3343c, (MTV016.43c), len: 2523 aa. PPE54, Member of the Mycobacterium
                     tuberculosis PPE family, MPTR subgroup of Gly-, Asn-rich proteins. Most similar to
                     O50379|Rv3350c|MTV004.07c|MTV004_5 from Mycobacterium tuberculosis strain H37Rv (3716
                     aa), FASTA scores: opt: 4672, E(): 4e-211, (44.2% identity in 3174 aa overlap); and
                     also similar to MTV004_3, MTCY63_9, MTY13E10_17,MTY13E10_16, MTCY180_1, MTV050_1,
                     MTCY3C7_23, MTV014_3,MTCY63_10; etc."
                     /codon_start=1
                     /transl_table=11
                     /product="PPE family protein PPE54"
                     /protein_id="YP_177960.1"
                     /db_xref="GI:57117092 GeneID:888033"
                     /translation="MSFVVMPPEINSLLIYTGAGPGPLLAAAAAWDELAAELGSAAAAFGSVTSGLVGGIWQGPSSVAMAAAAAP
                     YAGWLSAAAASAESAAGQARAVVGVFEAALAETVDPFVIAANRSRLVSLALSNLFGQNTPAIAAAEFDYELMWAQDVAAMLGYHT
                     GASAAAEALAPFGSPLASLAAAAEPAKSLAVNLGLANVGLFNAGSGNVGSYNVGAGNVGSYNVGGGNIGGNNVGLGNVGWGNFGL
                     GNSGLTPGLMGLGNIGFGNAGSYNFGLANMGVGNIGFANTGSGNFGIGLTGDNLTGFGGFNTGSGNVGLFNSGTGNVGFFNSGTG
                     NWGVFNSGSYNTGIGNSGIASTGLFNAGGFNTGVVNAGSYNTGSFNAGEANTGGFNPGSVNTGWLNTGDINTGVANSGDVNTGAF
                     ISGNYSNGVLWRGDYQGLLGFSSGANVLPVIPLSLDINGGVGAITIEPIHILPDIPININETLYLGPLVVPPINVPAISLGVGIP
                     NISIGPIKINPITLWPAQNFNQTITLAWPVSSITIPQIQQVALSPSPIPTTLIGPIHINTGFSIPVTFSYSTPALTLFPVGLSIP
                     TGGPLTLTLGVTAGTEAFTIPGFSIPEQPLPLAINVIGHINALSTPAITIDNIPLNLHAIGGVGPVDIVGGNVPASPGFGNSTTA
                     PSSGFFNTGAGGVSGFGNVGAHTSGWFNQSTQAMQVLPGTVSGYFNSGTLMSGIGNVGTQLSGMLSGGALGGNNFGLGNIGFDNV
                     GFGNAGSSNFGLANMGIGNIGLANTGNGNIGIGLSGDNLTGFGGFNSGSENVGLFNSGTGNVGFFNSGTGNLGVFNSGSHNTGFF
                     LTGNNINVLAPFTPGTLFTISEIPIDLQVIGGIGPIHVQPIDIPAFDIQITGGFIGIREFTLPEITIPAIPIHVTGTVGLEGFHV
                     NPAFVLFGQTAMAEITADPVVLPDPFITIDHYGPPLGPPGAKFPSGSFYLSISDLQINGPIIGSYGGPGTIPGPFGATFNLSTSS
                     LALFPAGLTVPDQTPVTVNLTGGLDSITLFPGGLAFPENPVVSLTNFSVGTGGFTVFPQGFTVDRIPVDLHTTLSIGPFPFRWDY
                     IPPTPANGPIPAVPGGFGLTSGLFPFHFTLNGGIGPISIPTTTVVDALNPLLTVTGNLEVGPFTVPDIPIPAINFGLDGNVNVSF
                     NAPATTLLSGLGITGSIDISGIQITNIQTQPAQLFMSVGQTLFLFDFRDGIELNPIVIPGSSIPITMAGLSIPLPTVSESIPLNF
                     SFGSPASTVKSMILHEILPIDVSINLEDAVFIPATVLPAIPLNVDVTIPVGPINIPIITEPGSGNSTTTTSDPFSGLAVPGLGVG
                     LLGLFDGSIANNLISGFNSAVGIVGPNVGLSNLGGGNVGLGNVGDFNLGAGNVGGFNVGGGNIGGNNVGLGNVGFGNVGLANSGL
                     TPGLMGLGNIGFGNAGSYNFGLANMGVGNIGFANTGSGNFGIGLTGDNLTGFGGFNTGSGNVGLFNSGTGNVGFFNSGTGNWGVF
                     NSGSYNTGIGNSGIASTGLFNAGGFNTGVVNAGSYNTGSFNAGQANTGGFNPGSVNTGWLNTGDINTGVANSGDVNTGAFISGNY
                     SNGAFWRGDYQGLLGFSYRPAVLPQTPFLDLTLTGGLGSVVIPAIDIPAIRPEFSANVAIDSFTVPSIPIPQIDLAATTVSVGLG
                     PITVPHLDIPRVPVTLNYLFGSQPGGPLKIGPITGLFNTPIGLTPLALSQIVIGASSSQGTITAFLANLPFSTPVVTIDEIPLLA
                     SITGHSEPVDIFPGGLTIPAMNPLSINLSGGTGAVTIPAITIGEIPFDLVAHSTLGPVHILIDLPAVPGFGNTTGAPSSGFFNSG
                     AGGVSGFGNVGAMVSGGWNQAPSALLGGGSGVFNAGTLHSGVLNFGSGMSGLFNTSVLGLGAPALVSGLGSVGQQLSGLLASGTA
                     LHQGLVLNFGLADVGLGNVGLGNVGDFNLGAGNVGGFNVGGGNIGGNNVGLGNVGWGNFGLGNSGLTPGLMGLGNIGFGNAGSYN
                     FGLANMGVGNIGFANTGSGNFGIGLTGDNLTGFGGFNTGSGNVGLFNSGTGNVGFFNSGTGNWGVFNSGSYNTGIGNSGIASTGL
                     FNAGGFNTGVVNAGSYNTGSFNAGQANTGGFNPGSVNTGWLNTGDINTGVANSGDVNTGAFISGNYSNGAFWRGDYQGLLGFSYT
                     STIIPEFTVANIHASGGAGPIIVPSIQFPAIPLDLSATGHIGGFTIPPVSISPITVRIDPVFDLGPITVQDITIPALGLDPATGV
                     TVGPIFSSGSIIDPFSLTLLGFINVNVPAIQTAPSEILPFTVLLSSLGVTHLTPEITIPGFHIPVDPIHVELPLSVTIGPFVSPE
                     ITIPQLPLGLALSGATPAFAFPLEITIDRIPVVLDVNALLGPINAGLVIPPVPGFGNTTAVPSSGFFNIGGGGGLSGFHNLGAGM
                     SGVLNAISDPLLGSASGFANFGTQLSGILNRGADISGVYNTGALGLITSALVSGFGNVGQQLAGLIYTGTGP"
     CDS             complement(3743711..3753184)
                     /gene="PPE55"
                     /locus_tag="Rv3347c"
                     /experiment="EXISTENCE: identified in proteomics study"
                     /note="Rv3347c, (MTV004.03c), len: 3157 aa. PPE55, Member of the Mycobacterium
                     tuberculosis PPE family, Gly-, Ala-,Asn-rich protein. Similar to many from
                     Mycobacterium tuberculosis strains H37Rv and CDC1551, e.g. O50379|Rv3350c|MTV004.07c
```

(3716 aa), FASTA scores: opt: 6497, E(): 0, (61.65% identity in 3756 aa overlap); and
other upstream ORFs MTV004_5, MTY13E10_15, MTCY28_16,MTCY63_9, MTY13E10_17,
MTCY180_1; etc. Predicted possible vaccine candidate (See Zvi et al., 2008)."
/codon_start=1
/transl_table=11
/product="PPE family protein PPE55"
/protein_id="YP_177963.1"
/db_xref="GI:57117095 GeneID:888120"
/translation="MNFPVLPPEINSVLMYSGAGSSPLLAAAAAWDGLAEELGSAAVSFGQVTSGLTAGVWQGAAAAAMAAAAAP
YAGWLGSVAAQAVAVAGQARAAVAAFEAALAATVDPAAVAVNRMAMRALAMSNLLGQNAAAIAAVEAEYELMWAADVAAMAGYHS
GASAAAAALPAFSPPAQALGGGVGAFLNALFAGPAKMLRLNAGLGNVGNYNVGLGNVGIFNLGAANVGAQNLGAANAGSGNFGFG
NIGNANFGFGNSGLGLPPGMGNIGLGNAGSSNYGLANLGVGNIGFANTGSNNIGIGLTGDNLTGIGGLNSGTGNLGLFNSGTGNI
GFFNSGTGNFGVFNSGSYNTGVGNAGTASTGLFNVGGFNTGVANVGSYNTGSFNAGNTNTGGFNPGNVNTGWLNTGNTNTGIANS
GNVNTGAFISGNFSNGVLWRGDYEGLWGLSGGSTIPAIPIGLELNGGVGPITVLPIQILPTIPLNIHQTFSLGPLVVPDIVIPAF
GGGTAIPISVGPITISPITLFPAQNFNTTFPVGPFFGLGVVNISGIEIKDLAGNVTLQLGNLNIDTRINQSFPVTVNWSTPAVTI
FPNGISIPNNPLALLASASIGTLGFTIPGFTIPAAPLPLTIDIDGQIDGFSTPPITIDRIPLNLGASVTVGPILINGVNIPATPG
FGNTTTAPSSGFFNSGDGGVSGFGNFGAGSSGWWNQAQTEVAGAGSGFANFGSLGSGVLNFGSGVSGLYNTGGLPPGTPAVVSGI
GNVGEQLSGLSSAGTALNQSLIINLGLADVGSVNVGFGNVGDFNLGAANIGDLNVGLGNVGGGNVGFGNIGDANFGLGNAGLAAG
LAGVGNIGLGNAGSGNVGFGNMGVGNIGFGNTGTNNLGIGLTGDNQTGIGGLNSGAGNIGLFNSGTGNVGLFNSGTGNFGLFNSG
SFNTGIGNGGTGSTGLFNAGNFNTGVANPGSYNTGSFNVGDTNTGGFNPGSINTGWFNTGNANTGVANSGNVDTGALMSGNFSNG
ILWRGNFEGLFGLNVGITIPEFPIHWTSTGGIGPIIIPDTTILPPIHLGLTGQANYGFAVPDIPIPAIHIDFDGAADAGFTAPAT
TLLSALGITGQFRFGPITVSNVQLNPFNVNLKLQFLHDAFPNEFPDPTISVQIQVAIPLTSATLGGLALPLQQTIDAIELPAISF
SQSIPIDIPPIDIPASTINGISMSEVVPIDVSVDIPAVTITGTRIDPIPLNFDVLSSAGPINISIIDIPALPGFGNSTELPSSGF
FNTGGGGGSGIANFGAGVSGLLNQASSPMVGTLSGLGNAGSLASGVLNSGVDISGMFNVSTLGSAPAVISGFGNLGNHVSGVSID
GLLAMLTSGGSGGSGQPSIIDAAIAELRHLNPLNIVNLGNVGSYNLGFANVGDVNLGAGNLGNLNLGGGNLGGQNLGLGNLGDGN
VGFGNLGHGNVGFGNSGLGALPGIGNIGLGNAGSNNVGFGNMGLGNIGFGNTGTNNLGIGLTGDNQTGFGGLNSGAGNLGLFNSG
TGNIGFFNTGTGNWGLFNSGSYNTGIGNSGTGSTGLFNAGSFNTGLANAGSYNTGSLNAGNTNTGGFNPGNVNTGWFNAGHTNTG
GFNTGNVNTGAFNSGSFNNGALWTGDHHGLVGFSYSIEITGSTLVDINETLNLGPVHIDQIDIPGMSLFDIHELVNIGPFRIEPI
DVPAVVLDIHETMVIPPIVFLPSMTIGGQTYTIPLDTPPAPAPPPFRLPLLFVNALGDNWIVGASNSTGMSGGFVTAPTQGILIH
TGPSSATTGSLALTLPTVTIPTITTSPIPLKIDVSGGLPAFTLFPGGLNIPQNAIPLTIDASGVLDPITIFPGGFTIDPLPLSLA
LNISVPDSSVPIIIVPPTPGFGNATATPSSGFFNSGAGGVSGFGNFGAGSSGWWNQAHAALAGAGSGVLNVGTLNSGVLNVGSGI
SGLYNTAIVGLGTPALVSGAGNVGQQLSGVLAAGTALTQSPIINLGLADVGNYNLGLGNVGDFNLGAANLGDLNLGLGNIGNANV
GFGNIGHGNVGFGNSGLGAALGIGNIGLGNAGSTNVGLANMGVGNIGFANTGTNNLGIGLTGDNQTGIGGLNSGAGNIGLFNSGT
GNIGFFNSGTGNWGLFNSGSFNTGIGNSGTGSTGLFNAGGFTTGLANAGSYNTGSFNVGDTNTGGFNPGSINTGWFNTGNANTGI
ANSGNVDTGALMSGNFSNGILWRGNYEGLFSYSYSLDVPRITILDAHFTGAFGPVVVPPIPVLAINAHLTGNAAMGAFTIPQIDI
PALNPNVTGSVGFGPIAVPSVTIPALTAARAVLDMAASVGATSEIEPFIVWTSSGAIGPTWYSVGRIYNAGDLFVGGNIISGIPT
LSTTGPVHAVFNAASQAFNTPALNIHQIPLGFQVPGSIDAITLFPGGLTFPANSLLNLDVFVGTPGATIPAITFPEIPANADGEL
YVIAGDIPLINIPPTPGIGNTTTVPSSGFFNTGAGGGSGFGNFGANMSGWWNQAHTALAGAGSGIANVGTLHSGVLNLGSGLSGI
YNTSTLPLGTPALVSGLGNVGDHLSGLLASNVGQNPITIVNIGLANVGNGNVGLGNIGNLNLGAANIGDVNLGFGNIGDVNLGFG
NIGGGNVGFGNIGDANFGFGNSGLAAGLAGMGNIGLGNAGSGNVGWANMGLGNIGFGNTGTNNLGIGLTGDNQSGIGGLNSGTGN
IGLFNSGTGNIGFFNSGTANFGLFNSGSYNTGIGNSGVASTGLVNAGGFNTGVANAGSYNTGSFNAGDTNTGGFNPGSTNTGWFN
TGNANTGVANAGNVNTGALITGNFSNGILWRGNYEGLAGFSFGYPIPLFPAVGADVTGDIGPATIIPPIHIPSIPLGFAAIGHIG
PISIPNIAIPSIHLGIDPTFDVGPITVDPITLTIPGLSLDAAVSEIRMTSGSSSGFKVRPSFSFFAVGPDGMPGGEVSILQPFTV
APINLNPTTLHFPGFTIPTGPIHIGLPLSLTIPGFTIPGGTLIPQLPLGLGLSGGTPPFDLPTVVIDRIPVELHASTTIGPVSLP
IFGFGGAPGFGNDTTAPSSGFFNTGGGGGSGFSNSGSGMSGVLNAISDPLLGSASGFANFGTQLSGILNRGAGISGVYNTGTLGL
VTSAFVSGFMNVGQQLSGLLFAGTGP"

CDS complement(3755952..3767102)
/gene="PPE56"
/locus_tag="Rv3350c"
/experiment="EXISTENCE: identified in proteomics study"
/note="Rv3350c, (MTV004.07c), len: 3716 aa. PPE56, Member of the Mycobacterium
tuberculosis PPE family of Gly-, Ala-,Asn-rich proteins, similar to many
Mycobacterium tuberculosis proteins from strains H37Rv and CDC1551, e.g.
O50378|Rv3347c|MTV004.03c (3157 aa), FASTA scores: opt: 6497, E(): 0, (61.65%
identity in 3756 aa overlap); MTCY28_16, MTV050_2, MTY13E10_17, MTCY63_10,
MTCY180_1,MTCY63_9, MTV050_1, MTV014_3, MTY13E10_15; etc."
/codon_start=1
/transl_table=11
/product="PPE family protein PPE56"
/protein_id="YP_177964.1"
/db_xref="GI:57117096 GeneID:888113"
/translation="MEFPVLPPEINSVLMYSGAGSSPLLAAAAAWDGLAEELGSAAVSFGQVTSGLTAGVWQGAAAAAMAAAAAP
YAGWLGSVAAAAEAVAGQARVVVGVFEAALAATVDPALVAANRARLVALAVSNLLGQNTPAIAAAEAEYELMWAADVAAMAGYHS
GASAAAAALPAFSPPAQALGGGVGAFLTALFASPAKALSLNAGLGNVGNYNVGLGNVGVFNLGAGNVGGQNLGFGNAGGTNVGFG
NLGNGNVGFGNSGLGAGLAGLGNIGLGNAGSSNYGFANLGVGNIGFGNTGTNNVGVGLTGNHLTGIGGLNSGTGNIGLFNSGTGN
VGFFNSGTGNFGVFNSGNYNTGVGNAGTASTGLFNAGNFNTGVVNVGSYNTGSFNAGDTNTGGFNPGGVNTGWLNTGNTNTGIAN
SGNVNTGAFISGNFNNGVLWVGDYQGLFGVSAGSSIPAIPIGLVLNGDIGPITIQPIPILPTIPLSIHQTVNLGPLVVPDIVIPA
FGGGIGIPINIGPLTITPITLFAQQTFVNQLPFPTFSLGKITIPQIQTFDSNGQLVSFIGPIVIDTTIPGPTNPQIDLTIRWDTP
PITLFPNGISAPDNPLGLLVSVSISNPGFTIPGFSVPAQPLPLSIDIEGQIDGFSTPPITIDRIPLTVGGGVTIGPITIQGLHIP

```
                     AAPGVGNTTTAPSSGFFNSGAGGVSGFGNVGAGSSGWWNQAPSALLGAGSGVGNVGTLGSGVLNLGSGISGFYNTSVLPFGTPAA
                     VSGIGNLGQQLSGVSAAGTTLRSMLAGNLGLANVGNFNTGFGNVGDVNLGAANIGGHNLGLGNVGDGNLGLGNIGHGNLGFANLG
                     LTAGAAGVGNVGFGNAGINNYGLANMGVGNIGFANTGTGNIGIGLVGDHRTGIGGLNSGIGNIGLFNSGTGNVGFFNSGTGNFGI
                     GNSGRFNTGIGNSGTASTGLFNAGSFSTGIANTGDYNTGSFNAGDTNTGGFNPGGINTGWFNTGHANTGLANAGTFGTGAFMTGD
                     YSNGLLWRGGYEGLVGVRVGPTISQFPVTVHAIGGVGPLHVAPVPVPAVHVEITDATVGLGPFTVPPISIPSLPIASITGSVDLA
                     ANTISPIRALDPLAGSIGLFLEPFRLSDPFITIDAFQVVAGVLFLENIIVPGLTVSGQILVTPTPIPLTLNLDTTPWTLFPNGFT
                     IPAQTPVTVGMEVANDGFTFFPGGLTFPRASAGVTGLSVGLDAFTLLPDGFTLDTVPATFDGTILIGDIPIPIIDVPAVPGFGNT
                     TTAPSSGFFNTGGGGGSGFANVGAGTSGWWNQGHDVLAGAGSGVANAGTLSSGVLNVGSGISGWYNTSTLGAGTPAVVSGIGNLG
                     QQLSGFLANGTVLNRSPIVNIGWADVGAFNTGLGNVGDLNWGAANIGAQNLGLGNLGSGNVGFGNIGAGNVGFANSGPAVGLAGL
                     GNVGLSNAGSNNWGLANLGVGNIGLANTGTGNIGIGLVGDYQTGIGGLNSGSGNIGLFNSGTGNVGFFNTGTGNFGLFNSGSFNT
                     GIGNSGTGSTGLFNAGNFNTGIANPGSYNTGSFNVGDTNTGGFNPGDINTGWFNTGIMNTGTRNTGALMSGTDSNGMLWRGDHEG
                     LFGLSYGITIPQFPIRITTTGGIGPIVIPDTTILPPLHLQITGDADYSFTVPDIPIPAIHIGINGVVTVGFTAPEATLLSALKNN
                     GSFISFGPITLSNIDIPPMDFTLGLPVLGPITGQLGPIHLEPIVVAGIGVPLEIEPIPLDAISLSESIPIRIPVDIPASVIDGIS
                     MSEVVPIDASVDIPAVTITGTTISAIPLGFDIRTSAGPLNIPIIDIPAAPGFGNSTQMPSSGFFNTGAGGGSGIGNLGAGVSGLL
                     NQAGAGSLVGTLSGLGNAGTLASGVLNSGTAISGLFNVSTLDATTPAVISGFSNLGDHMSGVSIDGLIAILTFPPAESVFDQIID
                     AAIAELQHLDIGNALALGNVGGVNLGLANVGEFNLGAGNVGNINVGAGNLGGSNLGLGNVGTGNLGFGNIGAGNFGFGNAGLTAG
                     AGGLGNVGLGNAGSGSWGLANVGVGNIGLANTGTGNIGIGLTGDYRTGIGGLNSGTGNLGLFNSGTGNIGFFNTGTGNFGLFNSG
                     SYSTGVGNAGTASTGLFNAGNFNTGLANAGSYNTGSLNVGSFNTGGVNPGTVNTGWFNTGHTNTGLFNTGNVNTGAFNSGSFNNG
                     ALWTGDYHGLVGFSFSIDIAGSTLLDLNETLNLGPIHIEQIDIPGMSLFDVHEIVEIGPFTIPQVDVPAIPLEIHESIHMDPIVL
                     VPATTIPAQTRTIPLDIPASPGSTMTLPLISMRFEGEDWILGSTAAIPNFGDPFPAPTQGITIHTGPGPGTTGELKISIPGFEIP
                     QIATTRFLLDVNISGGLPAFTLFAGGLTIPTNAIPLTIDASGALDPITIFPGGYTIDPLPLHLALNLTVPDSSIPIIDVPPTPGF
                     GNTTATPSSGFFNSGAGGVSGFGNVGSNLSGWWNQAASALAGSGSGVLNVGTLGSGVLNVGSGVSGIYNTSVLPLGTPAVLSGLG
                     NVGHQLSGVSAAGTALNQIPILNIGLADVGNFNVGFGNVGDVNLGAANLGAQNLGLGNVGTGNLGFANVGHGNIGFGNSGLTAGA
                     AGLGNTGFGNAGSANYGFANQGVRNIGLANTGTGNIGIGLVGDNLTGIGGLNSGAGNIGLFNSGTGNIGFFNSGTGNFGIGNSGS
                     FNTGIGNSGTGSTGLFNAGSFNTGVANAGSYNTGSFNAGDTNTGGFNPGTINTGWFNTGHTNTGIANSGNVGTGAFMSGNFSNGL
                     LWRGDHEGLFSLFYSLDVPRITIVDAHLDGGFGPVVLPPIPVPAVNAHLTGNVAMGAFTIPQIDIPALTPNITGSAAFRIVVGSV
                     RIPPVSVIVEQIINASVGAEMRIDPFEMWTQGTNGLGITFYSFGSADGSPYATGPLVFGAGTSDGSHLTISASSGAFTTPQLETG
                     PITLGFQVPGSVNAITLFPGGLTFPATSLLNLDVTAGAGGVDIPAITWPEIAASADGSVYVLASSIPLINIPPTPGIGNSTITPS
                     SGFFNAGAGGGSGFGNFGAGTSGWWNQAHTALAGAGSGFANVGTLHSGVLNLGSGVSGIYNTSTLGVGTPALVSGLGNVGHQLSG
                     LLSGGSAVNPVTVLNIGLANVGSHNAGFGNVGEVNLGAANLGAHNLGFGNIGAGNLGFGNIGHGNVGVGNSGLTAGVPGLGNVGL
                     GNAGGNNWGLANVGVGNIGLANTGTGNIGIGLTGDYQTGIGGLNSGAGNLGLFNSGAGNVGFFNTGTGNFGLFNSGSFNTGVGNS
                     GTGSTGLFNAGSFNTGVANAGSYNTGSFNVGDTNTGGFNPGSINTGWLNAGNANTGVANAGNVNTGAFVTGNFSNGILWRGDYQG
                     LAGFAVGYTLPLFPAVGADVSGGIGPITVLPPIHIPPIPVGFAAVGGIGPIAIPDISVPSIHLGLDPAVHVGSITVNPITVRTPP
                     VLVSYSQGAVTSTSGPTSEIWVKPSFFPGIRIAPSSGGGATSTQGAYFVGPISIPSGTVTFPGFTIPLDPIDIGLPVSLTIPGFT
                     IPGGTLIPTLPLGLALSNGIPPVDIPAIVLDRILLDLHADTTIGPINVPIAGFGGAPGFGNSTTLPSSGFFNTGAGGGSGFSNTG
                     AGMSGLLNAMSDPLLGSASGFANFGTQLSGILNRGAGISGVYNTGALGVVTAAVVSGFGNVGQQLSGLLFTGVGP"
     CDS             3842239..3842769
                     /gene="PPE57"
                     /locus_tag="Rv3425"
                     /note="Rv3425, (MTCY78.04c), len: 176 aa. PPE57, Member of the M. tuberculosis PPE
                     family, similar to many e.g. O06246|Rv3429|MTCY77.01 (178 aa), FASTA scores: opt:
                     781,E(): 7e-44, (69.9% identity in 176 aa overlap); and downstream
                     Q50702|YY26_MYCTU|Rv3426|MTCY78.03c (232 aa),FASTA scores: opt: 517, E(): 1.2e-26,
                     (68.0% identity in 125 aa overlap); MTV049_11, MTCY428_16, MTV049_22,MTV049_30,
                     MTCY261_4; etc."
                     /codon_start=1
                     /transl_table=11
                     /product="PPE family protein PPE57"
                     /protein_id="YP_177971.1"
                     /db_xref="GI:57117105 GeneID:887635"
                     /translation="MHPMIPAEYISNIIYEGPGADSLFFASGQLRELAYSVETTAESLEDELDELDENWKGSSSDLLADAVERYL
                     QWLSKHSSQLKHAAWVINGLANAYNDTRRKVVPPEEIAANREERRRLIASNVAGVNTPAIADLDAQYDQYRARNVAVMNAYVSWT
                     RSALSDLPRWREPPQIYRGG"
     CDS             3843036..3843734
                     /gene="PPE58"
                     /locus_tag="Rv3426"
                     /experiment="EXISTENCE: identified in proteomics study"
                     /note="Rv3426, (MTCY78.03c), len: 232 aa. PPE58, Member of the M. tuberculosis PPE
                     family, similar to many e.g. the downstream O06246|Rv3429|MTCY77.01 (178 aa), FASTA
                     scores: opt: 555, E(): 6.5e-26, (72.0% identity in 125 aa overlap); and upstream
                     Q50703|YY25_MYCTU|Rv3425|MTCY78.04c (176 aa),FASTA scores: opt: 517, E(): 1.1e-23,
                     (68.0% identity in 125 aa overlap); MTV049_30, MTCY3C7_24, MTCY428_16,MTCY3A2_22;
                     etc."
                     /codon_start=1
                     /transl_table=11
                     /product="PPE family protein PPE58"
                     /protein_id="YP_177972.1"
                     /db_xref="GI:57117106 GeneID:887622"
                     /translation="MHLMIPAEYISNVIYEGPRADSLYAADQRLRQLADSVRTTAESLNTTLDELHENWKGSSSEWMADAALRYL
```

DWLSKHSRQILRTARVIESLVMAYEETLLRVVPPATIANNREEVRRLIASNVAGGKHSSNRRPRGTIRAVPGRKYPSNGPLSKLD
PICAIEAAPMAGAAADPQERVGPRGRRGLAGQQQCRGRPGPSLRCSHDTPRFQMNQAFHTMVNMLLTCFACQEKPR"

CDS 3847165..3847701
/gene="PPE59"
/locus_tag="Rv3429"
/note="Rv3429, (MTCY77.01), len: 178 aa. PPE59, Member of the M. tuberculosis PPE family, similar to many e.g. the upstream Q50703|YY25_MYCTU|Rv3425|MTCY78.04c (176 aa),FASTA scores: opt: 781, E(): 1.9e-44, (69.9% identity in 176 aa overlap); and Q50702|YY26_MYCTU|Rv3426|MTCY78.03c (232 aa), FASTA scores: opt: 555, E(): 1.7e-29, (72.0% identity in 125 aa overlap) (but diverges at 3' end); etc."
/codon_start=1
/transl_table=11
/product="PPE family protein PPE59"
/protein_id="YP_177973.1"
/db_xref="GI:57117107 GeneID:887630"
/translation="MHPMIPAEYISNIIYEGPGADSLSAAAEQLRLMYNSANMTAKSLTDRLGELQENWKGSSSDLMADAAGRYL
DWLTKHSRQILETAYVIDFLAYVYEETRHKVVPPATIANNREEVHRLIASNVAGVNTPAIAGLDAQYQQYRAQNIAVMNDYQSTA
RFILAYLPRWQEPPQIYGGGGG"

CDS 3894426..3895607
/gene="PPE60"
/locus_tag="Rv3478"
/gene_synonym="mtb39c"
/experiment="EXISTENCE: identified in proteomics study"
/note="Rv3478, (MTCY13E12.31), len: 393 aa. PPE60 (alternate gene name: mtb39c). Member of the M. tuberculosis PPE family, highly similar to others e.g. Q11031|YD61_MYCTU|Rv1361c|MT1406|MTCY02B10.25c (396 aa),FASTA scores: opt: 2165, E(): 1.1e-109, (85.35% identity in 396 aa overlap); MTCI364.08; MTCY10G2.10; MTCY03A2.22c; MTCY274.23c; MTCY164.34c; MTCY98.0029c; etc. Note that expression of Rv3478 was demonstrated in lysates by immunodetection (see Dillon et al., 1999)."
/codon_start=1
/transl_table=11
/product="PE family protein PPE60"
/protein_id="YP_177976.1"
/db_xref="GI:57117111 GeneID:888047"
/translation="MVDFGALPPEINSARMYAGPGSASLVAAAKMWDSVASDLFSAASAFQSVVWGLTVGSWIGSSAGLMAAAAS
PYVAWMSVTAGQAQLTAAQVRVAAAAYETAYRLTVPPPVIAENRTELMTLTATNLLGQNTPAIEANQAAYSQMWGQDAEAMYGYA
ATAATATEALLPFEDAPLITNPGGLLEQAVAVEEAIDTAAANQLMNNVPQALQQLAQPAQGVVPSSKLGGLWTAVSPHLSPLSNV
SSIANNHMSMMGTGVSMTNTLHSMLKGLAPAAAQAVETAAENGVWAMSSLGSQLGSSLGSSGLGAGVAANLGRAASVGSLSVPPA
WAAANQAVTPAARALPLTSLTSAAQTAPGHMLGGLPLGHSVNAGSGINNALRVPARAYAIPRTPAAG"

CDS 3969343..3970563
/gene="PPE61"
/locus_tag="Rv3532"
/note="Rv3532, (MTCY03C7.24c), len: 406 aa. PPE61, Member of the Mycobacterium tuberculosis PPE protein family,similar to many, e.g. O53956|Rv1807|MTV049.29 (403 aa),FASTA scores: opt: 954, E(): 1.1e-43, (44.1% identity in 417 aa overlap)"
/codon_start=1
/transl_table=11
/product="PPE family protein PPE61"
/protein_id="YP_177984.1"
/db_xref="GI:57117120 GeneID:888370"
/translation="MFMDFAMLPPEVNSTRMYSGPGAGSLWAAAAAWDQVSAELQSAAETYRSVIASLTGWQWLGPSSVRMGAAV
TPYVEWLTTTAAQARQTATQITAAATGFEQAFAMTVPPPAIMANRAQVLSLIATNFFGQNTAAIAALETQYAEMWEQDATAMYDY
AATSAAARTLTPFTSPQQDTNSAGLPAQSAEVSRATANAGAADGNWLGNLLEEIGILLLPIAPELTPFFLEAGEIVNAIPFPSIV
GDEFCLLDGLLAWYATIGSINNINSMGTGIIGAEKNLGILPELGSAAAAAAPPPADIAPAFLAPLTSMAKSLSDGALRGPGEVSA
AMRGAGTIGQMSVPPAWKAPAVTTVRAFDATPMTTLPGGDAPAAGVPGLPGMPASGAGRAGVVPRYGVRLTVMTRPLSGG"

CDS complement(3970705..3972453)
/gene="PPE62"
/locus_tag="Rv3533c"
/note="Rv3533c, (MTCY03C7.23), len: 582 aa. PPE62, Member of the Mycobacterium tuberculosis PPE protein family,similar to many, e.g. O53309|Rv3159c|MTV014.03c (590 aa) FASTA scores: opt: 2289, E(): 2.3e-95, (63.5% identity in 600 aa overlap). Predicted to be an outer membrane protein (See Song et al., 2008)."
/codon_start=1
/transl_table=11
/product="PPE family protein PPE62"
/protein_id="YP_177985.1"
/db_xref="GI:57117121 GeneID:888385"
/translation="MNYAVLPPELNSLRMFTGAGSAPMLAAAVAWDGLAAELGSAASSFGSVTSDLASQAWQGPAAAAMAAAAAP
YAGWLSAAAARAAGAAAQAKAVASAFEAARAATVHPLLVAANRNAFAQLVMSNWFGLNAPLIAAVEGAYEQMWAADVAAMVGYHS

```
                     GASAAAEQLVPFQQALQQLPNLGIGNIGNANLGGGNTGDLNTGNGNIGNTNLGSGNRGDANLGSGNIGNSNVGGGNVGNGNFGSG
                     NGRAGLPGSGNVGNGNLGNSNLGSGNTGNSNVGFGNTGNNNVGTGNAGSGNIGAGNTGSSNWGFGNNGIGNIGFGNTGNGNIGFG
                     LTGNNQVGIGGLNSGSGNIGLFNSGTNNVGFFNSGNGNLGIGNSSDANVGIGNSGATVGPFVAGHNTGFGNSGSLNTGMGNAGGV
                     NTGFGNGGAINLGFGNSGQLNAGSFNAGSINTGNFNSGQGNTGDFNAGVRNTGWSNSGLTNTGAFNAGSLNTGFGAVGTGSGPNS
                     GFGNAGTNNSGFFNTGVGSSGFQNGGSNNSGLQNAVGTVIAAGFGNTGAQTVGIANSGVLNSGFFNSGVHNSGGFNSENQRSGFG
                     N"
     CDS             3978059..3979498
                     /gene="PPE63"
                     /locus_tag="Rv3539"
                     /experiment="EXISTENCE: identified in proteomics study"
                     /note="Rv3539, (MTCY03C7.17c), len: 479 aa. PPE63, Member of the Mycobacterium
                     tuberculosis PPE protein family,similar to many e.g. O53949|Rv1800|MTV049.22 (655
                     aa),FASTA scores: opt: 914, E(): 7.3e-47, (37.55% identity in 490 aa overlap); etc."
                     /codon_start=1
                     /transl_table=11
                     /product="PPE family protein PPE63"
                     /protein_id="YP_177987.1"
                     /db_xref="GI:57117123 GeneID:888438"
                     /translation="MADFLTLSPEVNSARMYAGGGPGSLSAAAAAWDELAAELWLAAASFESVCSGLADRWWQGPSSRMMAAQAA
                     RHTGWLAAAATQAEGAASQAQTMALAYEAAFAATVHPALVAANRALVAWLAGSNVFGQNTPAIAAAEAIYEQMWAQDVVAMLNYH
                     AVASAVGARLRPWQQLLHELPRRLGGEHSDSTNTELANPSSTTTRITVPGASPVHAATLLPFIGRLLAARYAELNTAIGTNWFPG
                     TTPEVVSYPATIGVLSGSLGAVDANQSIAIGQQMLHNEILAATASGQPVTVAGLSMGSMVIDRELAYLAIDPNAPPSSALTFVEL
                     AGPERGLAQTYLPVGTTIPIAGYTVGNAPESQYNTSVVYSQYDIWADPPDRPWNLLAGANALMGAAYFHDLTAYAAPQQGIEIAA
                     VTSSLGGTTTTYMIPSPTLPLLLPLKQIGVPDWIVGGLNNVLKPLVDAGYSQYAPTAGPYFSHGNLVW"
     CDS             3997980..3999638
                     /gene="PPE64"
                     /locus_tag="Rv3558"
                     /note="Rv3558, (MTCY06G11.05), len: 552 aa. PPE64, Member of the Mycobacterium
                     tuberculosis PPE family of glycine-rich proteins, similar to many e.g.
                     P71868|Rv3533c|MTCY03C7.23 (582 aa), FASTA scores: opt: 1908, E(): 1.7e-83, (58.5%
                     identity in 583 aa overlap). Predicted to be an outer membrane protein (See Song et
                     al.,2008)."
                     /codon_start=1
                     /transl_table=11
                     /product="PPE family protein PPE64"
                     /protein_id="YP_177988.1"
                     /db_xref="GI:57117124 GeneID:887822"
                     /translation="MAHFSVLPPEINSLRMYLGAGSAPMLQAAAAWDGLAAELGTAASSFSSVTTGLTGQAWQGPASAAMAAAAA
                     PYAGFLTTASAQAQLAAGQAKAVASVFEAAKAAIVPPAAVAANREAFLALIRSNWLGLNAPWIAAVESLYEEYWAADVAAMTGYH
                     AGASQAAAQLPLPAGLQQFLNTLPNLGIGNQGNANLGGGNTGSGNIGNGNKGSSNLGGGNIGNNNIGSGNRGSDNFGAGNVGTGN
                     IGFGNQGPIDVNLLATPGQNNVGLGNIGNNNMGFGNTGDANTGGGNTGNGNIGGGNTGNNNFGFGNTGNNNIGIGLTGNNQMGIN
                     LAGLLNSGSGNIGIGNSGTNNIGLFNSGSGNIGVFNTGANTLVPGDLNNLGVGNSGNANIGFGNAGVLNTGFGNASILNTGLGNA
                     GELNTGFGNAGFVNTGFDNSGNVNTGNGNSGNINTGSWNAGNVNTGFGIITDSGLTNSGFGNTGTDVSGFFNTPTGPLAVDVSGF
                     FNTASGGTVINGQTSGIGNIGVPGTLFGSVRSGLNTGLFNMGTAISGLFNLRQLLG"
     CDS             complement(4060648..4061889)
                     /gene="PPE65"
                     /locus_tag="Rv3621c"
                     /experiment="EXISTENCE: identified in proteomics study"
                     /note="Rv3621c, (MTCY15C10.31, MTCY07H7B.01), len: 413 aa. PPE65, Member of the
                     Mycobacterium tuberculosis PPE family,ala-, gly-rich proteins, similar to many e.g.
                     Q10813|YS92_MYCTU|Rv2892c|MT2959|MTCY274.23c (408 aa) FASTA scores: opt: 955, E():
                     1.8e-42, (44.45% identity in 423 aa overlap)."
                     /codon_start=1
                     /transl_table=11
                     /product="PPE family protein PPE65"
                     /protein_id="YP_177998.1"
                     /db_xref="GI:57117135 GeneID:885097"
                     /translation="MLDFAQLPPEVNSALMYAGPGSGPMLAAAAAWEALAAELQTTASTYDALITGLADGPWQGSSAASMVAAAT
                     PQVAWLRSTAGQAEQAGSQAVAAASAYEAAFFATVPPPEIAANRALLMALLATNFLGQNTAAIAATEAQYAEMWAQDAAAMYGYA
                     GASAAATQLSPFNPAAQTINPAGLASQAASVGQAVSGAANAQALTDIPKALFGLSGIFTNEPPWLTDLGKALGLTGHTWSSDGSG
                     LIVGGVLGDFVQGVTGSAELDASVAMDTFGKWVSPARLMVTQFKDYFGLAHDLPKWASEGAKAAGEAAKALPAAVPAIPSAGLSG
                     VAGAVGQAASVGGLKVPAVWTATTPAASPAVLAASNGLGAAAAAEGSTHAFGGMPLMGSGAGRAFNNFAAPRYGFKPTVIAQPPA
                     GG"
     CDS             complement(4189285..4190232)
                     /gene="PPE66"
                     /locus_tag="Rv3738c"
                     /note="Rv3738c, (MTV025.086c), len: 315 aa. PPE66, Member of the Mycobacterium
                     tuberculosis PPE family, highly similar to many e.g. O53265|Rv3018c|MTV012.32c (434
                     aa),FASTA scores: opt: 464, E(): 2.2e-17, (47.05% identity in 338 aa overlap).
```

Probably a continuation of the upstream ORF MTV025.87c|Rv3739c|PPE67. At position 97470-72 a stop codon is present which interrupts a possibly longer ORF,observed in related ORFs MTV012_32 or MTCY21B4_4. The sequence has been checked and no errors were detected. A similar situation, but with a frameshift separating the ORFs is found in MTV012_36/MTV012_35. Sequence similarity is also seen with MTCY251_15; MTCY261_19; MLCB2492_30 from Mycobacterium leprae; MTCY10G2_10; MTY21C12_9; MTCI125_26; MTCY164_36; MTCY6A4_1."
/codon_start=1
/transl_table=11
/product="PPE family protein PPE66"
/protein_id="YP_178009.1"
/db_xref="GI:57117150 GeneID:886262"
/translation="MTTAYASALAAMPTLTELAANHTSHAVLLGTNFFGINTIPIALNEADYARMWIQAATTMSIYEGTSDAALASAPQTTPAPVLFNGGAGVASALPAISAATLDPASIIGIIIEILIQLFLISLEILFAIVAYTIIIVLILPLVIFAYAIVFAVLAIIFGPPLLVIASPFVLTGSVIAVPTSLSTSLSTAVPIGVGQYLADLASADAQAIEVGLKTADVAPVAVRPAAAPPLRESAAVRPEARLVSAVAPAPAGTSASVLASDRGAGVLGFAGTAGKESVGRPAGLTTLAGGEFGGSPSVPMVPASWEQLVGAGEAG"

CDS complement(4190284..4190517)
/gene="PPE67"
/locus_tag="Rv3739c"
/note="Rv3739c, (MTV025.087c), len: 77 aa. PPE67, Member of the Mycobacterium tuberculosis PE family, showing high homology with O53269|Rv3022c|MTV012.36c (82 aa) FASTA scores: opt: 398, E(): 1.2e-19, (74.0% identity in 77 aa overlap); and similar to the N-termini of other PPE proteins e.g. O53265|Rv3018c|MTV012.32c (434 aa) FASTA scores: opt: 398, E(): 4.8e-19, (74.0% identity in 77 aa overlap). ORF ends at the stop codon at position 97470,which is not present in similar ORFs: MTV012_32, or MTCY21B4_4. Sequence homology with MTV012_32, and MTCY21B4_4 continues in the downstream ORF MTV025.086c|Rv3738c|PPE66. Sequence was checked, but no errors were detected. A similar situation, but with a frameshift separating the ORFs, is found in MTV012_36/MTV012_35. Also ORF MTV025.87c shows similarity to MTV03 _14; MTCY6A4_1; MTV035_8; MTV037_17; MLCB2492_30; MTCY261_19; MTCY251_15; MTCY3A2_23; MTCY28_16; etc."
/codon_start=1
/transl_table=11
/product="PPE family protein PPE67"
/protein_id="YP_178010.1"
/db_xref="GI:57117151 GeneID:886257"
/translation="MTAPIWFASPPEVHSALLSAGPGPASLQAAAAEWTSLSAEYASAAQELTAVLAAVQGGAWEGPSAEAYVAAHLPYLA"

```
     CDS             4221604..4222710
                     /gene="PPE68"
                     /locus_tag="Rv3873"
                     /experiment="EXISTENCE: identified in proteomics study"
                     /note="Rv3873, (MTV027.08), len: 368 aa. PPE68, Member of the Mycobacterium
                     tuberculosis PPE family, highly similar to many e.g. O33085|ML0051|MLCB628.14c from
                     Mycobacterium leprae (302 aa), FASTA scores: opt: 656, E(): 2.8e-24,(46.2% identity
                     in 288 aa overlap); and O53691|Rv0286|MTV035.14 (513 aa), FASTA scores: opt: 566,E():
                     7.8e-20, (35.25% identity in 363 aa overlap). A core mycobacterial gene; conserved in
                     mycobacterial strains (See Marmiesse et al., 2004). Predicted possible vaccine
                     candidate (See Zvi et al., 2008)."
                     /codon_start=1
                     /transl_table=11
                     /product="PPE family protein PPE68"
                     /protein_id="YP_178022.1"
                     /db_xref="GI:57117164 GeneID:886201"
                     /translation="MLWHAMPPELNTARLMAGAGPAPMLAAAAGWQTLSAALDAQAVELTARLNSLGEAWTGGGSDKALAAATPM
                     VVWLQTASTQAKTRAMQATAQAAAYTQAMATTPSLPEIAANHITQAVLTATNFFGINTIPIALTEMDYFIRMWNQAALAMEVYQA
                     ETAVNTLFEKLEPMASILDPGASQSTTNPIFGMPSPGSSTPVGQLPPAATQTLGQLGEMSGPMQQLTQPLQQVTSLFSQVGGTGG
                     GNPADEEAAQMGLLGTSPLSNHPLAGGSGPSAGAGLLRAESLPGAGGSLTRTPLMSQLIEKPVAPSVMPAAAAGSSATGGAAPVG
                     AGAMGQGAQSGGSTRPGLVAPAPLAQEREEDDEDDWDEEDDW"
     CDS             complement(4245013..4246212)
                     /gene="PPE69"
                     /locus_tag="Rv3892c"
                     /experiment="EXISTENCE: identified in proteomics study"
                     /note="Rv3892c, (MTCY15F10.20), len: 399 aa. PPE69, Member of the Mycobacterium
                     tuberculosis PPE family of conserved proteins, similar to many e.g.
                     O05298|Rv1196|MTCI364.08 from Mycobacterium leprae (391 aa), FASTA scores: opt:
                     348,E(): 2.2e-08, (26.6% identity in 380 aa overlap)."
                     /codon_start=1
                     /transl_table=11
                     /product="PPE family protein PPE69"
                     /protein_id="YP_178024.1"
                     /db_xref="GI:57117166 GeneID:886227"
                     /translation="MPDPGWAARTPEANDLLLTAGTGVGTHLANQTAWTTLGASHHASGVASAINTAATAASWLGVGSAASALNV
                     TMLNATLHGLAGWVDVKPAVVSTAIAAFETANAAMRPAPECMENRDEWGVDNAINPSVLWTLTPRIVSLDVEYFGVMWPNNAAVG
                     ATYGGVLAALAESLAIPPPVATMGASPAAPAQAAAAVGQAAAEAAAGDGMRSAYQGVQAGSTGAGQSTSAGENFGNQLSTFMQPM
                     QAVMQAAPQALQAPSGLMQAPMSAMQPLQSMVGMFANPGALGMGGAAPGASAASAAGGISAAATEVGAGGGGAALGGGGMPATSF
                     TRPVSAFESGTSGRPVGLRPSGALGADVVRAPTTTVGGTPIGGMPVGHAAGGHRGSHGKSEQAATVRVVDDRR"
```

```
     misc_feature    complement(3295265..3296341)
                     /gene="PPE47"
                     /locus_tag="Rv3021c"
                     /note="Rv3021c, (MTV012.35c), len: 358 aa. PPE47, Member of Mycobacterium
                     tuberculosis PPE family. Should be continuation of upstream ORF MTV012.36c but is
                     frameshifted due to missing base at 36448 in v012. Sequence has been checked but no
                     error apparent. Very similar to neighbouring ORF O53265|MTV012.32c|Rv3018c from
                     Mycobacterium tuberculosis (434 aa), FASTA scores: opt: 1714, E(): 6.6e-770, (78.3%
                     identity in 355 aa overlap) and AAK47430|MT3101 (strongly in the N-terminal part)
                     (310 aa),FASTA scores: opt: 897, E(): 4.5e-37, (66.95% identity in 227 aa overlap)."
                     /pseudogene="unknown"
                     /db_xref="PSEUDO:CCP45828.1"
     misc_feature    complement(3296329..3296571)
                     /gene="PPE48"
                     /locus_tag="Rv3022c"
                     /note="Rv3022c, (MTV012.36c), len: 81 aa. PPE48, Member of M. tuberculosis PPE family
                     with frameshift due to missing bp in codon 82. The ORF continues in downstream
                     MTV012.35c. The sequence has been checked and no errors were detected. Identical to
                     neigbouring ORF O53265|Rv3018c|MTV012.32c (434 aa), FASTA scores: opt: 526, E():
                     6.2e-26, (100.0% identity in 81 aa overlap); and O69706|Rv739c|MTV025.087c (77
                     aa),FASTA scores: opt: 392, E(): 3.4e-18, (72.7% identity in 77 aa overlap)."
                     /pseudogene="unknown"
```

```
     CDS             33582..33794
                     /locus_tag="Rv0031"
                     /note="Rv0031, (MTCY10H4.31), len: 70 aa. Possible remnant of a transposase, showing
                     partial similarity to mycobacterial transposases in a short overlap, e.g.
                     Rv2791c|MTV002_57 (459 aa), FASTA score: (72.2% identity in 36 aa overlap); Rv2885c,
                     Rv2978c, Rv3827c, etc."
                     /codon_start=1
                     /transl_table=11
                     /product="Possible remnant of a transposase"
                     /protein_id="NP_214545.1"
                     /db_xref="GI:15607173 GeneID:887049"
                     /translation="MLARHFGAGRKAHSRAVATLKADIQAWHPAGIQTPKPRCESDVFARIGHTSHPSTRKSRVGPGASEAPLA"
     CDS             649489..650232
                     /locus_tag="Rv0606"
                     /note="Rv0606, (MTCY19H5.16c), len: 247 aa. Possible truncated transposase for
                     IS_1536 element, highly similar to N-terminus of other transposases from
                     Mycobacterium tuberculosis e.g. YX16_MYCTU|Q10809|Rv2885c|MT2953|MTCY274. 16c
                     putative transposase from Mycobacterium tuberculosis (460 aa), FASTA scores: opt:
                     1368, E(): 0, (83.5% identity in 237 aa overlap); Rv2978c, Rv0922, Rv3827c, etc. Also
                     similar to N-terminus of MTV002_57|Rv2792 resolvase from M. tuberculosis (193 aa),
                     FASTA score: (87.4% identity in 238 aa overlap). This region is a possible
                     MT-complex-specific genomic island (See Becq et al., 2007)."
                     /codon_start=1
                     /transl_table=11
                     /product="Possible transposase (fragment)"
                     /protein_id="NP_215120.1"
                     /db_xref="GI:15607746 GeneID:887889"
                     /translation="MPRLEIPNGWCVQAFRFTLDPTAEQAHALARHFGARRKAYNWTVAQLKADIQAWRATGAQTAKPSLRVLRK
                     RWNTVKDEVCVNAETGTVWWPECSKEAYADGIAGAVDAYWNWQQRRAGKRDGKRMGFPRFKKKGRDADRVSFTTGAMRVEPDRRH
                     LTLPVIGCVRTHENTRRIERLIAKDRARVLAITVRRNGTRLDASVRVLVQRPQQPNVELPESRIGVDVGVRRLATVATADGACCP
                     VLVPDG"
     CDS             780007..780321
                     /locus_tag="Rv0741"
                     /note="Rv0741, (MTV041.15), len: 104 aa. Probable truncated transposase for IS1557,
                     showing similarity to transposases and is elements e.g. U63997|EFU63997_1 insertion
                     sequence from Enterococcus faecium (424 aa), FASTA score: (31.0% identity in 87 aa
                     overlap). Very high similarity with the C-terminal part of Z73419|MTCY373_3 2 IS1557
                     from Mycobacterium tuberculosis (444 aa), FASTA score: (86.5% identity in 104 aa
                     overlap). This region is a possible MT-complex-specific genomic island (See Becq et
                     al.,2007)."
                     /codon_start=1
                     /transl_table=11
                     /product="Probable transposase (fragment)"
                     /protein_id="NP_215255.1"
                     /db_xref="GI:15607881 GeneID:888644"
                     /translation="MFSVKGEEGKQALDRWISWARRCRIPVFVELAGGIVRHRQAIDAALDHGLWQGLIESTNTKIRLLTRIAFG
                     FRSPEALIALAMLALGGRRPALPGRTKHPRISQ"
```

```
     CDS             complement(787564..787749)
                     /locus_tag="Rv0755A"
                     /note="Rv0755A, len: 61 aa. Putative transposase (possibly gene fragment), similar to
                     C-terminal part of Q9EZM2|ISMav2|AF286339_1 putative transposase from Mycobacterium
                     paratuberculosis (395 aa), FASTA scores: opt: 284, E(): 5e-13, (83.02% identity in 53
                     aa overlap); and to SCJ11.25c|Q9RI80 possible noncomposite transposon transposase
                     from Streptomyces coelicolor (283 aa)."
                     /codon_start=1
                     /transl_table=11
                     /product="Putative transposase (fragment)"
                     /protein_id="YP_177633.1"
                     /db_xref="GI:57116778 GeneID:3205072"
                     /translation="MKELSVAEQRYQAVLAVISDGLSISQVAEKVGVSRQTLHTWLARYEAEGLDGLRIGTGTAL"
     CDS             826294..826620
                     /locus_tag="Rv0795"
                     /experiment="EXISTENCE: identified in proteomics study"
                     /note="Rv0795, (MTV042.05), len: 108 aa. Putative transposase for IS6110 (fragment),
                     identical to Q50686 insertion element IS6110 (108 aa), FASTA score: (100.0 % identity
                     in 108 aa overlap). The transposase described here may be made by a frame shifting
                     mechanism during translation that fuses Rv0795 and Rv0796, the sequence UUUUAAAG
                     (directly upstream of Rv0796) maybe responsible for such a frameshifting event (see
                     McAdam et al., 1990)."
                     /codon_start=1
                     /transl_table=11
                     /product="Putative transposase for insertion sequence element IS6110 (fragment)"
                     /protein_id="NP_215310.1"
                     /db_xref="GI:15607935 GeneID:885454"
                     /translation="MSGGSSRRYPPELRERAVRMVAEIRGQHDSEWAAISEVARLLGVGCAETVRKWVRQAQVDAGARPGTTTEE
                     SAELKRLRRDNAELRRANAILKTASAFFAAELDRPAR"
     CDS             826569..827555
                     /locus_tag="Rv0796"
                     /experiment="EXISTENCE: identified in proteomics study"
                     /note="Rv0796, (MTV042.06), len: 328 aa. Putative transposase for IS6110. Identical
                     to many other M. tuberculosis IS6110 transposase subunits. The transposase described
                     here may be made by a frame shifting mechanism during translation that fuses Rv0795
                     and Rv0796, the sequence UUUUAAAG (directly upstream of Rv0796) maybe responsible for
                     such a frameshifting event (see McAdam et al., 1990). Start changed since first
                     submission (+ 50 aa)."
                     /codon_start=1
                     /transl_table=11
                     /product="Putative transposase for insertion sequence element IS6110"
                     /protein_id="NP_215311.2"
                     /db_xref="GI:448824749 GeneID:885099"
                     /translation="KDRVGFLRGRARPASTLITRFIADHQGHREGPDGLRWGVESICTQLTELGVPIAPSTYYDHINREPSRREL
                     RDGELKEHISRVHAANYGVYGARKVWLTLNREGIEVARCTVERLMTKLGLSGTTRGKARRTTIADPATARPADLVQRRFGPPAPN
                     RLWVADLTYVSTWAGFAYVAFVTDAYARRILGWRVASTMATSMVLDAIEQAIWTRQQEGVLDLKDVIHHTDRGSQYTSIRFSERL
                     AEAGIQPSVGAVGSSYDNALAETINGLYKTELIKPGKPWRSIEDVELATARWVDWFNHRRLYQYCGDVPPVELEAAYYAQRQRPA
                     AG"
     CDS             827610..828704
                     /locus_tag="Rv0797"
                     /note="Rv0797, (MTCI249B.03c, MTV042.07), len: 364 aa. Putative transposase for
                     IS1547; almost identical to (but 20 aa shorter than) Y13470|MTY13470_2 from
                     Mycobacterium tuberculosis (383 aa). Also similar to other transposases e.g.
                     MAIS1110A _1|Q48909 transposase from Mycobacterium avium (464 aa), FASTA scores: opt:
                     226, E(): 2.4e-08,(30.7% identity in 199 aa overlap). Also slight similarity to
                     Rv2014|MTCY39.03c from Mycobacterium tuberculosis (222 aa), FASTA score: (24.8%
                     identity in 141 aa overlap)."
                     /codon_start=1
                     /transl_table=11
                     /product="Putative transposase for insertion sequence element IS1547"
                     /protein_id="NP_215312.1"
                     /db_xref="GI:15607937 GeneID:885476"
                     /translation="MVVVGTDAHKYSHTFVATDEVGRQLGEKTVKATTAGHATAIMWAREQFGLELIWGIEDCRNMSARLERDLL
                     AAGQQVVRVPTKLMAQTRKSARSRGKSDPIDALAVARAVMRETDLPLATHDETSRELKLLTDRRDVLVAQRTSAINRLRWLVHEL
                     DPERAPAARSLDAAKHQQALRTWLDTQPGLVAELARAELTDIIRLTGEINTLAQRISARVHQVAPALLEIPGCAELTAAKIVGEA
                     AGVTRFKSEAAFACHAAVAPIPVWSGNTAGQMRLSRSGNRQLNAALHRIALTQIRMTDSRGQAYYQRLQDAGKTKRAALRCLKRR
                     LARTVFQALRTVHQPSSEHTQPAAACHRSYCSRSCLSG"
     CDS             858797..859087
                     /locus_tag="Rv0829"
```

```
                     /note="Rv0829, (MTV043.21), len: 96 aa. Possible transposase for IS1605' (fragment),
                     similar to C-terminal end of many mycobacterial transposases and hypothetical
                     proteins e.g. Z74024|MTCY274_16 from Mycobacterium tuberculosis (460 aa), FASTA
                     scores: opt: 668, E(): 6.2e-32, (98.9% identity in 93 aa overlap); MTV002_57|O33333
                     transposase from Mycobacterium tuberculosis ; L07627|SERRY1_1 insertion element
                     IS1136 from Saccharopolyspora erythraea (90 aa), FASTA score: (34.9% identity in 83
                     aa overlap)."
                     /codon_start=1
                     /transl_table=11
                     /product="Possible transposase (fragment)"
                     /protein_id="NP_215344.1"
                     /db_xref="GI:15607969 GeneID:885403"
                     /translation="MGPSSKTCHACRHVQDIGWDEKWQCDGCSITHQRDDNAAINLARYEEPPSVVGPVGAAVKRGADRKTGPGP
                     AGGREARKATGHPAGEQPRDGVLVA"
     CDS             879225..879557
                     /locus_tag="Rv0850"
                     /note="Rv0850, (MTV043.43), len: 110 aa. Putative transposase (fragment), similar in
                     part to others e.g. Q45144|Q4514 transposable element IS31831 (436 aa), FASTA scores:
                     opt: 175, E(): 4.3e-05, (38.6% identity in 57 aa overlap); etc."
                     /codon_start=1
                     /transl_table=11
                     /product="Putative transposase (fragment)"
                     /protein_id="NP_215365.1"
                     /db_xref="GI:15607990 GeneID:885054"
                     /translation="MTRDPHSPDCGREGSYRDTITRPLTDLPVAGYPLVPRVASPRYRCTTPQCGRAVFNQDLANVDQYLVVNQL
                     AHQLIDGSSLIPDADKRWDARRHADMTHHLTSSLKENQS"
     CDS             complement(952685..954004)
                     /locus_tag="Rv0920c"
                     /experiment="EXISTENCE: identified in proteomics study"
                     /inference="protein motif:PROSITE:PS01007"
                     /note="Rv0920c, (MTCY21C12.14c), len: 439 aa. Probable transposase for IS1554, highly
                     similar to others e.g. MTCY441.35|Q45111 transposase from Mycobacterium tuberculosis
                     (419 aa), FASTA scores: opt: 1113, E(): 0,(43.9% identity in 378 aa overlap); etc.
                     Contains transposases mutator family signature (PS01007)."
                     /codon_start=1
                     /transl_table=11
                     /product="Probable transposase"
                     /protein_id="NP_215435.1"
                     /db_xref="GI:15608060 GeneID:885549"
                     /translation="MDAAQVIEPAHAGQDVDEAAVAARELSGAERALVGDLVRQARAEGVALTGPDGLLKALTKTVLEAALQEEM
                     TEHLGYDRHAAAGRGSGNSRNGSRNKKVITDACGQVEIAVPRDRNGTFEPVIVGKRKRRVTDVDRVVLSLYAKGLTTGEIAAHFA
                     DVYGVSVSKDTISRITDRVIEEMQAWWSRPLEKVYAAVFIDAIMVKIRDGQVRNRPVYAAIGVDLDGHKDILGMWAGEGDGESAK
                     FWLAVLTDLRNRGVKDIFFLVCDGLKGLPDSVSAAFPLATVQTCIIHLIRNTFRYASRKYWDKISVDLKPIYTAASAAEARLRYE
                     EFAEKWGKPYPAITRLWDSAWEEFIPFLDYDVEIRRVPCSTNAIESLNARYRRAVRARGHFPNEQSALKTLYLVTRSLDPKGTGQ
                     TKWAVRWKPALNALAITFADRMPAAEER"
     CDS             954873..956525
                     /locus_tag="Rv0922"
                     /experiment="EXISTENCE: identified in proteomics study"
                     /note="Rv0922, (MTCY21C12.16), len: 550 aa. Possible transposase for IS1535, similar
                     to many e.g. YX16_MYCTU|Q10809|MTCY274.16c from Mycobacterium tuberculosis (460 aa),
                     FASTA scores: opt 939, E(): 0,(40.6% identity in 465 aa overlap); etc."
                     /codon_start=1
                     /transl_table=11
                     /product="Possible transposase"
                     /protein_id="NP_215437.1"
                     /db_xref="GI:15608062 GeneID:885564"
                     /translation="MIVRMRSCAQAAKVAEATGGVQLAGKPKPDGTPTFSRYVEIGVDFEAHRPVVESVSVLFELYDGDANSYAA
                     TGGPGAQLPSGWMVTAAKFEVEWPADPQRAGLVRSHFGARRKAFNWGLAQVKADLDAKAADPAHESVDWDLKSLRWAWNRAKDDV
                     APWWAENSKECYSSGLADLAQGLANWKAGKNGTRKGRRVGFPRFKSGRRDPGRVRFTTGTMRIEDDRRTITVPVIGPLRAKENTR
                     RVQRHLVSGRAQILNMTLSQRWGRLFVAVCYALRTPTTRSPLTQPTVRAGMDLGVRTLATVATLDTATGEQTIIEYPNPAPLKAT
                     LVARRRAGRELSRRIPGSHGHRAVKAKLARLDRRCVHLRREAAHQLTTELAGTYGQVVIEDLDVAAMKRSMRRRAFRRSVSDAAM
                     GLVAPQLAYKTAKCSGVLTVADRWFASSQIHHGCTSPDGTPCRLQGKGRIDKHLLCPVTGEVVDRDRNAALNLRDWPDNASRGPV
                     GTTAPSAPGPTTTVGTGHGADTGSSGAGGASVRPRPRRAGRGEAKTQTPQGDAA"
     CDS             complement(1080964..1081353)
                     /locus_tag="Rv1034c"
                     /note="Rv1034c, (MTCY10G2.15), len: 129 aa. Probable IS1560 transposase fragment,
                     similar to part of Rv3387|E1202305|MTV004.45 (225 aa) (65.1% identity in 129 aa
                     overlap)."
                     /codon_start=1
```

```
                     /transl_table=11
                     /product="Probable transposase (fragment)"
                     /protein_id="NP_215550.1"
                     /db_xref="GI:15608174 GeneID:886010"
                     /translation="MQQGNPPDAPQLAPAVAWVKKRAGRTPRTVTADRGYGEAAVDQQLTEVGVKNVLIPRKGKPSQDRRAEEHR
                     KAFRRTIKWRTGCEGRISHLKRGYGWDRGRIGGLEGTRTWVGHGVFAHNLVTISALPA"
     CDS             complement(1081421..1082107)
                     /locus_tag="Rv1035c"
                     /experiment="EXISTENCE: identified in proteomics study"
                     /note="Rv1035c, (MTCY10G2.14), len: 228 aa. Probable IS1560 transposase fragment,
                     similar to parts of Rv3387|E1202305|MTV004.45 (225 aa) (47.8% identity in 67 aa
                     overlap) and Rv3386|E1202304|MTV004.44 (234 aa) (55.1% identity in 127 aa overlap)."
                     /codon_start=1
                     /transl_table=11
                     /product="Probable transposase (fragment)"
                     /protein_id="NP_215551.1"
                     /db_xref="GI:15608175 GeneID:888206"
                     /translation="MPHPTTLMKLTTRCGSAAIDGLNEALLAKAAEAKLLGTNRIRADTTVARANVSYPTDLGLLAKAMRRIAAT
                     GKRIQAAGGAVRTRVGDRSRAAGRRAHAVAAKLRSRAELGRDEARAAVLRFTGELAELAQAAAQEAQQLLDNAKQAVLRAKAKAA
                     ALAARGERDAVAGRRCGGLVRAVNDLTELLNATRQIVAQTRQRVAGITSDGASRRVSLHDGDARPDHQGSAR"
     CDS             complement(1082141..1082479)
                     /locus_tag="Rv1036c"
                     /note="Rv1036c, (MTCY10G2.13), len: 112 aa. Probable IS1560 transposase fragment,
                     similar to part of Rv3386|E1202304|MTV004.44 (234 aa) (82.8% identity in 87 aa
                     overlap)."
                     /codon_start=1
                     /transl_table=11
                     /product="Probable IS1560 transposase (fragment)"
                     /protein_id="NP_215552.1"
                     /db_xref="GI:15608176 GeneID:888227"
                     /translation="MIPGRMVLNWEDGLNALVAEGIEAIVFRTLGDQCWLWESLLPDEVRRLPEELARVDALLDDPAFFAPFVPF
                     FDPRRGRPSTPMEVYLQLMFVKFRYRLGYESLCREVADSIT"
     CDS             complement(1084614..1085477)
                     /locus_tag="Rv1041c"
                     /experiment="EXISTENCE: identified in proteomics study"
                     /note="Rv1041c, (MTCY10G2.08), len: 287 aa. Probable is like-2 transposase, overlaps
                     MTCY10G2.07. Similar to Q00430|X53945 insertion element IS869 hypothetical protein
                     from Agrobacterium tumefaciens (186 aa), FASTA scores: opt: 173, E(): 0.00016, (40.9%
                     identity in 176 aa overlap). Similar to Rv1150, C-terminal part of transposase of
                     putative Mycobacterium tuberculosis is like-1. MTCY10G2.07 and MTCY10G2.08 are
                     frameshifted with respect to Mycobacterium tuberculosis Q50761 transposase, the 10G2
                     cosmid sequence appears to be correct. This region is a possible MT-complex-specific
                     genomic island (See Becq et al., 2007)."
                     /codon_start=1
                     /transl_table=11
                     /product="Probable is like-2 transposase"
                     /protein_id="NP_215557.1"
                     /db_xref="GI:15608181 GeneID:888546"
                     /translation="MRASPADGLAITGLSWKGSRGGSVREVRGGTCPLSSGRGKRCGSAITVGRWMVPATRCSPTLPRCSGWTLR
                     WPRISRSCCRWIPRTCGHTSIRRAPARTRSPQGALSDYKKSADEPDDHAIGRSRGGLTTKIHALTDQREAPVRIRLTAGQAGDNP
                     QLLPLLDDYRHASTEYALGSTDFRLLADKAYSHPSTRAALRSKKIKHTIPERQDQIDRRKAKGSAGGRPPAFDAALYGLRNTVER
                     GFHRLKQWRGIATRYDKYALTYLGGVLLACAVIHARVGTPKLGDTP"
     CDS             complement(1085134..1085541)
                     /locus_tag="Rv1042c"
                     /note="Rv1042c, (MTCY10G2.07), len: 135 aa. Probable is like-2 transposase, similar
                     to Q50761 transposase from Mycobacterium tuberculosis (308 aa), FASTA scores: opt:
                     823, E(): 0, (99.1% identity in 117 aa overlap). Second copy is Rv1149. This region
                     is a possible MT-complex-specific genomic island (See Becq et al.,2007)."
                     /codon_start=1
                     /transl_table=11
                     /product="Probable is like-2 transposase"
                     /protein_id="NP_215558.1"
                     /db_xref="GI:15608182 GeneID:888607"
                     /translation="MTRVGVISDEFWAVVEPLMPSHEGKPGRRFSDHRLILEGIAWRFRTGSPWRDLPAEFGPWQTVWKRHHRWS
                     LDGTCDEVFAHVAAVFGVDAEVAEDIEKLLSVDSTNVRAHQHSAGACSDTLATGGTVGLQEIRR"
     CDS             1089465..1090712
                     /locus_tag="Rv1047"
                     /inference="protein motif:PROSITE:PS01007"
                     /note="Rv1047, (MTCY10G2.02c), len: 415 aa. IS1081 transposase, most similar to
```

```
                     TRA1_MYCBO|P35882 transposase for insertion sequence element (415 aa), FASTA scores:
                     opt: 2675, E(): 0, (99.8% identity in 415 aa overlap). Contains PS01007 Transposases,
                     Mutator family, signature. This region is a possible MT-complex-specific genomic
                     island (See Becq et al., 2007)."
                     /codon_start=1
                     /transl_table=11
                     /product="Probable transposase"
                     /protein_id="NP_215563.1"
                     /db_xref="GI:15608187 GeneID:886060"
                     /translation="MTSSHLIDAEQLLADQLAQASPDLLRGLLSTFIAALMGAEADALCGAGYRERSDERSNQRNGYRHRDFDTR
                     AATIDVAIPKLRQGSYFPDWLLQRRKRAERALTSVVATCYLLGVSTRRMERLVETLGVTKLSKSQVSIMAKELDEAVEAFRTRPL
                     DAGPYTFLAADALVLKVREAGRVVGVHTLIATGVNAEGYREILGIQVTSAEDGAGWLAFFRDLVARGLSGVALVTSDAHAGLVAA
                     IGATLPAAAWQRCRTHYAANLMAATPKPSWPWVRTLLHSIYDQPDAESVVAQYDRVLDALTDKLPAVAEHLDTARTDLLAFTAFP
                     KQIWRQIWSNNPQERLNREVRRRTDVVGIFPDRASIIRLVGAVLAEQHDEWIEGRRYLGLEVLTRARAALTSTEEPAKQQTTNTP
                     ALTT"
     CDS             1097281..1097415
                     /locus_tag="Rv1055"
                     /note="Rv1055, (MTV017.08), len: 44 aa. Possible integrase (fragment); first 49 aa
                     similar to Rv2309c|MTCY3G12_25|Z79702 hypothetical protein (shows similarity to
                     integrases) from Mycobacterium tuberculosis (151 aa), FASTA scores: opt: 291, E():
                     2.2e-16, (74.3% identity in 70 aa overlap); and to L39071|MSGINT_1 integrase from
                     Mycobacterium paratuberculosis (191 aa),FASTA scores: opt: 146, E(): 8.3e-05, (52.1%
                     identity in 48 aa overlap); and to many other integrases or transposases. Shortened
                     since first submission (-34 aa). This region is a possible MT-complex-specific
                     genomic island (See Becq et al., 2007)."
                     /codon_start=1
                     /transl_table=11
                     /product="Possible integrase (fragment)"
                     /protein_id="NP_215571.2"
                     /db_xref="GI:57116817 GeneID:887138"
                     /translation="MTLDRHGHLLNDDLAVWPMRCAKSSRTLRYHCGMRRRNRVGLRA"
     CDS             1187197..1187604
                     /locus_tag="Rv1149"
                     /note="Rv1149, (MTCI65.16), len: 135 aa. Possible transposase. Identical to 117 aa
                     N-terminal region of S21394|X65618 transposase of Mycobacterium tuberculosis (308
                     aa), FASTA scores: opt: 823, E(): 0, (99.1% identity in 117 aa overlap). Second copy
                     is Rv1042c|MTCY10G2.07."
                     /codon_start=1
                     /transl_table=11
                     /product="Possible transposase"
                     /protein_id="NP_215665.1"
                     /db_xref="GI:15608289 GeneID:885164"
                     /translation="MTRVGVISDEFWAVVEPLMPSHEGKPGRRFSDHRLILEGIAWRFRTGSPWRDLPAEFGPWQTVWKRHHRWS
                     LDGTCDEVFAHVAAVFGVDAEVAEDIEKLLSVDSTNVRAHQHSAGACSDTLATGGTVGLQEIRR"
     CDS             complement(1247218..1248465)
                     /locus_tag="Rv1199c"
                     /inference="protein motif:PROSITE:PS00435 protein motif:PROSITE:PS01007"
                     /note="Rv1199c, (MTCI364.11c), len: 415 aa. Possible transposase for IS1081,
                     identical to TRA1_MYCBO|P35882 transposase for insertion sequence element (415 aa);
                     region identical to MTCY441.35 (100.0% identity in 261 aa overlap); and almost
                     identical to MTCY10G2.02c (415 aa) (99.8% identity in 415 aa overlap). Contains
                     PS01007 Transposases, Mutator family, signature, PS00435 Peroxidases proximal
                     heme-ligand signature."
                     /codon_start=1
                     /transl_table=11
                     /product="Possible transposase"
                     /protein_id="NP_215715.1"
                     /db_xref="GI:15608339 GeneID:886092"
                     /translation="MTSSHLIDTEQLLADQLAQASPDLLRGLLSTFIAALMGAEADALCGAGYRERSDERSNQRNGYRHRDFDTR
                     AATIDVAIPKLRQGSYFPDWLLQRRKRAERALTSVVATCYLLGVSTRRMERLVETLGVTKLSKSQVSIMAKELDEAVEAFRTRPL
                     DAGPYTFLAADALVLKVREAGRVVGVHTLIATGVNAEGYREILGIQVTSAEDGAGWLAFFRDLVARGLSGVALVTSDAHAGLVAA
                     IGATLPAAAWQRCRTHYAANLMAATPKPSWPWVRTLLHSIYDQPDAESVVAQYDRVLDALTDKLPAVAEHLDTARTDLLAFTAFP
                     KQIWRQIWSNNPQERLNREVRRRTDVVGIFPDRASIIRLVGAVLAEQHDEWIEGRRYLGLEVLTRARAALTSTEEPAKQQTTNTP
                     ALTT"
     CDS             complement(1372009..1373343)
                     /locus_tag="Rv1313c"
                     /experiment="EXISTENCE: identified in proteomics study"
                     /note="Rv1313c, (MTCY373.33c), len: 444 aa. Possible IS1557 transposase, similar to
                     several transposases e.g. U57649|DBU57649 ORF1 from dibenzofuran-degrading bacterium
```

DPO360 (163 aa), FASTA scores: opt: 767, E(): 0, (67.3% identity in 168 aa overlap); TNPA_BORPA|Q06126 transposase for insertion sequence element IS1001 from Bordetella parapertussis (406 aa), FASTA scores: opt: 254, E(): 3.3e-10, (24.9% identity in 402 aa overlap). Also similar to putative Mycobacterium tuberculosis transposases, Rv3798 and Rv0741."
/codon_start=1
/transl_table=11
/product="Possible transposase"
/protein_id="NP_215829.1"
/db_xref="GI:15608453 GeneID:886922"
/translation="MRNVRLFRALLGVDKRTVIEDIEFEEDDAGDGARVIARVRPRSAVLRRCGRCGRKASWYDRGAGLRQWRSLDWGTVEVFLEAEAPRVNCPTHGPTVVAVPWARHHAGHTYAFDDTVAWLAVACSKTAVCELMRIAWRTVGAIVARVWADTEKRIDRFANLRRIGIDEISYKRHHRYLTVVVDHDSGRLVWAAPGHDKATLGLFFDALGAERAAQITHVSADAADWIADVVTERCPDAIQCADPFHVVAWATEALDVERRRAWNDARAIARTEPKWGRGRPGKNAAPRPGRERARRLKGARYALWKNPEDLTERQSAKLAWIAKTDPRLYRAYLLKESLRHVFSVKGEEGKQALDRWISWAQRCRIPVFVELAARIKRHRVAIDAALDHGLSQGLIESTNTKIRLLTRIAFGFRSPQALIALAMLTLAGHRPTLPGRHNHPQISQ"

CDS complement(1442829..1443815)
/locus_tag="Rv1369c"
/note="Rv1369c, (MTCY02B12.03c), len: 328 aa. Probable transposase subunit for IS6110. Identical to many other M. tuberculosis IS6110 transposase subunits. The transposase described here may be made by a frame shifting mechanism during translation that fuses Rv1368c and Rv1369c, the sequence UUUUAAAG (directly upstream of Rv1369c) maybe responsible for such a frameshifting event (see McAdam et al., 1990). Start changed since first submission (+ 34 aa)."
/codon_start=1
/transl_table=11
/product="Probable transposase"
/protein_id="NP_215885.2"
/db_xref="GI:448824756 GeneID:886789"
/translation="KDRVGFLRGRARPASTLITRFIADHQGHREGPDGLRWGVESICTQLTELGVPIAPSTYYDHINREPSRRELRDGELKEHISRVHAANYGVYGARKVWLTLNREGIEVARCTVERLMTKLGLSGTTRGKARRTTIADPATARPADLVQRRFGPPAPNRLWVADLTYVSTWAGFAYVAFVTDAYARRILGWRVASTMATSMVLDAIEQAIWTRQQEGVLDLKDVIHHTDRGSQYTSIRFSERLAEAGIQPSVGAVGSSYDNALAETINGLYKTELIKPGKPWRSIEDVELATARWVDWFNHRRLYQYCGDVPPVELEAAYYAQRQRPAAG"

CDS complement(1443764..1444090)
/locus_tag="Rv1370c"
/note="Rv1370c, (MTCY02B12.04c), len: 108 aa. Putative transposase for IS6110 (fragment), identical to many other Mycobacterium tuberculosis IS6110 transposase subunits e.g. Q50686|YIA4_MYCTU Insertion element IS6110 hypothetical 12.0 kDa protein (108 aa), fasta scores: E(): 1.4e-43,(100.00% identity in 108 aa overlap). The transposase described here may be made by a frame shifting mechanism during translation that fuses Rv1368c and Rv1369c, the sequence UUUUAAAG (directly upstream of Rv1369c) maybe responsible for such a frameshifting event (see McAdam et al., 1990)."
/codon_start=1
/transl_table=11
/product="Putative transposase for insertion sequence element IS6110 (fragment)"
/protein_id="NP_215886.1"
/db_xref="GI:15608510 GeneID:886791"
/translation="MSGGSSRRYPPELRERAVRMVAEIRGQHDSEWAAISEVARLLGVGCAETVRKWVRQAQVDAGARPGTTTEESAELKRLRRDNAELRRANAILKTASAFFAAELDRPAR"

CDS complement(1863021..1864007)
/locus_tag="Rv1756c"
/note="Rv1756c, (MTCY28.22c), len: 328 aa. Putative Transposase subunit for IS6110. Identical to many other M. tuberculosis IS6110 transposase subunits. The transposase described here may be made by a frame shifting mechanism during translation that fuses Rv1756c and Rv1757c, the sequence UUUUAAAG (directly upstream of Rv1756c) maybe responsible for such a frameshifting event (see McAdam et al., 1990). Start changed since first submission (+ 34 aa)"
/codon_start=1
/transl_table=11
/product="Putative transposase"
/protein_id="NP_216272.2"
/db_xref="GI:448824763 GeneID:885541"
/translation="KDRVGFLRGRARPASTLITRFIADHQGHREGPDGLRWGVESICTQLTELGVPIAPSTYYDHINREPSRRELRDGELKEHISRVHAANYGVYGARKVWLTLNREGIEVARCTVERLMTKLGLSGTTRGKARRTTIADPATARPADLVQRRFGPPAPNRLWVADLTYVSTWAGFAYVAFVTDAYARRILGWRVASTMATSMVLDAIEQAIWTRQQEGVLDLKDVIHHTDRGSQYTSIRFSERLAEAGIQPSVGAVGSSYDNALAETINGLYKTELIKPGKPWRSIEDVELATARWVDWFNHRRLYQYCGDVPPVELEAAYYAQRQRPAAG"

```
     CDS             complement(1863956..1864282)
                     /locus_tag="Rv1757c"
                     /note="Rv1757c, (MTCY28.23c), len: 108 aa. Putative Transposase for IS6110
                     (fragment), identical to many other Mycobacterium tuberculosis IS6110 transposase
                     subunits e.g. Q50686|YIA4_MYCTU Insertion element IS6110 hypothetical 12.0 kDa
                     protein (108 aa), fasta scores: E(): 1.4e-43,(100.00% identity in 108 aa overlap).
                     The transposase described here may be made by a frame shifting mechanism during
                     translation that fuses Rv1756c and Rv1757c, the sequence UUUUAAAG (directly upstream
                     of Rv1756c) maybe responsible for such a frameshifting event (see McAdam et al.,
                     1990)."
                     /codon_start=1
                     /transl_table=11
                     /product="Putative transposase for insertion sequence element IS6110 (fragment)"
                     /protein_id="NP_216273.1"
                     /db_xref="GI:15608895 GeneID:885558"
                     /translation="MSGGSSRRYPPELRERAVRMVAEIRGQHDSEWAAISEVARLLGVGCAETVRKWVRQAQVDAGARPGTTTEE
                     SAELKRLRRDNAELRRANAILKTASAFFAAELDRPAR"
     CDS             1871428..1871754
                     /locus_tag="Rv1763"
                     /note="Rv1763, (MTCY28.29), len: 108 aa. Putative Transposase for IS6110 (fragment),
                     identical to many other Mycobacterium tuberculosis IS6110 transposase subunits e.g.
                     Q50686|YIA4_MYCTU Insertion element IS6110 hypothetical 12.0 kDa protein (108 aa),
                     fasta scores: E(): 1.4e-43,(100.00% identity in 108 aa overlap). The transposase
                     described here may be made by a frame shifting mechanism during translation that
                     fuses Rv1763 and Rv1764, the sequence UUUUAAAG (directly upstream of Rv1764) maybe
                     responsible for such a frameshifting event (see McAdam et al., 1990)."
                     /codon_start=1
                     /transl_table=11
                     /product="Putative transposase for insertion sequence element IS6110 (fragment)"
                     /protein_id="NP_216279.1"
                     /db_xref="GI:15608901 GeneID:885372"
                     /translation="MSGGSSRRYPPELRERAVRMVAEIRGQHDSEWAAISEVARLLGVGCAETVRKWVRQAQVDAGARPGTTTEE
                     SAELKRLRRDNAELRRANAILKTASAFFAAELDRPAR"
     CDS             1871703..1872689
                     /locus_tag="Rv1764"
                     /note="Rv1764, (MTCY28.30), len: 328 aa. Putative Transposase for IS6110 insertion
                     element. Identical to many other M. tuberculosis IS6110 transposase subunits. The
                     transposase described here may be made by a frame shifting mechanism during
                     translation that fuses Rv1763 and Rv1764,the sequence UUUUAAAG (directly upstream of
                     Rv1764) maybe responsible for such a frameshifting event (see McAdam et al., 1990).
                     Start changed since first submission (+ 34 aa)"
                     /codon_start=1
                     /transl_table=11
                     /product="Putative transposase"
                     /protein_id="NP_216280.2"
                     /db_xref="GI:448824764 GeneID:885238"
                     /translation="KDRVGFLRGRARPASTLITRFIADHQGHREGPDGLRWGVESICTQLTELGVPIAPSTYYDHINREPSRREL
                     RDGELKEHISRVHAANYGVYGARKVWLTLNREGIEVARCTVERLMTKLGLSGTTRGKARRTTIADPATARPADLVQRRFGPPAPN
                     RLWVADLTYVSTWAGFAYVAFVTDAYARRILGWRVASTMATSMVLDAIEQAIWTRQQEGVLDLKDVIHHTDRGSQYTSIRFSERL
                     AEAGIQPSVGAVGSSYDNALAETINGLYKTELIKPGKPWRSIEDVELATARWVDWFNHRRLYQYCGDVPPVELEAAYYAQRQRPA
                     AG"
     CDS             complement(1874418..1874633)
                     /locus_tag="Rv1765A"
                     /note="Rv1765A, len: 71 aa. Putative transposase (fragment), similar to part of many
                     transposase genes including IS6110 e.g. P19774|TRA9_MYCTU putative transposase from
                     Mycobacterium tuberculosis (278 aa), FASTA scores: opt: 231, E(): 4.7e-11, (45.35%
                     identity in 75 aa overlap)."
                     /codon_start=1
                     /transl_table=11
                     /product="Putative transposase (fragment)"
                     /protein_id="YP_177652.1"
                     /db_xref="GI:57116906 GeneID:3205098"
                     /translation="MWVADITFVRTWQGFCYTAFVTDVCTRKIVVWAVSATMRTEDLPVQVFNHAVWQSNSDLSELVHHSDPGSQ"
     CDS             2107396..2107875
                     /locus_tag="Rv2013"
                     /note="Rv2013, (MTCY39.04c), len: 159 aa. Transposase,shows similarity to N-terminal
                     part of transposase and insertion element hypothetical proteins. Length changed since
                     first submission (no clear start apparent)."
                     /codon_start=1
```

```
                     /transl_table=11
                     /product="Transposase"
                     /protein_id="NP_216529.2"
                     /db_xref="GI:57116936 GeneID:887546"
                     /translation="MDTLLEAGITVVVISPNQLKNLRGRYGSAGNKDDRFDAFVLADTLRTDRSRLRPLLPDTPATATLRRTCRP
                     RKDLVAHRVALANQLRAHLRVVFPGVVGLFADLDSPISLAFLTFLPRFDCQDRADWLSVKRLAGWLAAAGYCGRAPRPAHRCPAR
                     RHR"
     CDS             2107829..2108419
                     /locus_tag="Rv2014"
                     /note="Rv2014, (MTCY39.03c), len: 196 aa. Transposase,similar to insertion elements;
                     possibly made by frameshifting with respect to Rv2013. Length changed since first
                     submission."
                     /codon_start=1
                     /transl_table=11
                     /product="Transposase"
                     /protein_id="NP_216530.2"
                     /db_xref="GI:57116937 GeneID:887547"
                     /translation="MLHDRLTGAPRGATGDEGAANAHITRAMVAALTSVATQIKTLDAQIAEQLSLHADAHIFTSLPRSGTVRAA
                     RLLAEIGDCRARFPTPESLACLAGVAPSTRQSGKVKHVGFRWAADKQLRDAVCDFAGDSRRANLWAADRYNRAIARGHDHPHAVR
                     ILARAWLYAIWHCWQDGAAYHPANHRALQALLNQDQDRAA"
     CDS             2189758..2190063
                     /locus_tag="Rv2085"
                     /note="Rv2085, (MTCY49.24), len: 101 aa. Conserved hypothetical protein, similar to
                     but shorter than many transposases but we can find no sequence errors to account for
                     the frameshifts. Contains possible helix-turn-helix motif at aa 33 to 54,(+3.11 SD).
                     This region is a possible MT-complex-specific genomic island (See Becq et al.,2007)."
                     /codon_start=1
                     /transl_table=11
                     /product="hypothetical protein"
                     /protein_id="NP_216601.1"
                     /db_xref="GI:15609222 GeneID:888138"
                     /translation="MSDMCDVVSFVGAAERVLRARFRPSPESGPPVHARRCGWSLGISAETLRRWAGQAEVDSGVVAGVSASRSG
                     SVKTSELEQTIEILKVATSFFARKCDPRHR"
     CDS             2210718..2211044
                     /locus_tag="Rv2105"
                     /note="Rv2105, (MTCY261.01), len: 108 aa. Putative transposase for IS6110 (fragment),
                     identical to many other Mycobacterium tuberculosis IS6110 transposase subunits e.g.
                     Q50686|YIA4_MYCTU Insertion element IS6110 hypothetical 12.0 kDa protein (108 aa),
                     fasta scores: E(): 1.4e-43,(100.00% identity in 108 aa overlap). The transposase
                     described here may be made by a frame shifting mechanism during translation that
                     fuses Rv2105 and Rv2106, the sequence UUUUAAAG (directly upstream of Rv2106) maybe
                     responsible for such a frameshifting event (see McAdam et al., 1990)."
                     /codon_start=1
                     /transl_table=11
                     /product="Putative transposase for insertion sequence element IS6110 (fragment)"
                     /protein_id="NP_216621.1"
                     /db_xref="GI:15609242 GeneID:888395"
                     /translation="MSGGSSRRYPPELRERAVRMVAEIRGQHDSEWAAISEVARLLGVGCAETVRKWVRQAQVDAGARPGTTTEE
                     SAELKRLRRDNAELRRANAILKTASAFFAAELDRPAR"
     CDS             2210993..2211979
                     /locus_tag="Rv2106"
                     /note="Rv2106, (MTCY261.02), len: 328 aa. Probable transposase subunit for IS6110.
                     Identical to many other M. tuberculosis IS6110 transposase subunits. The transposase
                     described here may be made by a frame shifting mechanism during translation that
                     fuses Rv2105 and Rv2106, the sequence UUUUAAAG (directly upstream of Rv2106) maybe
                     responsible for such a frameshifting event (see McAdam et al., 1990). Start changed
                     since first submission (+ 16 aa)."
                     /codon_start=1
                     /transl_table=11
                     /product="Probable transposase"
                     /protein_id="NP_216622.2"
                     /db_xref="GI:448824775 GeneID:888398"
                     /translation="KDRVGFLRGRARPASTLITRFIADHQGHREGPDGLRWGVESICTQLTELGVPIAPSTYYDHINREPSRREL
                     RDGELKEHISRVHAANYGVYGARKVWLTLNREGIEVARCTVERLMTKLGLSGTTRGKARRTTIADPATARPADLVQRRFGPPAPN
                     RLWVADLTYVSTWAGFAYVAFVTDAYARRILGWRVASTMATSMVLDAIEQAIWTRQQEGVLDLKDVIHHTDRGSQYTSIRFSERL
                     AEAGIQPSVGAVGSSYDNALAETINGLYKTELIKPGKPWRSIEDVELATARWVDWFNHRRLYQYCGDVPPVELEAAYYAQRQRPA
                     AG"
     CDS             complement(2270591..2271577)
                     /locus_tag="Rv2167c"
```

```
                     /note="Rv2167c, (MTCY270.01), len: 328 aa. Probable IS6110 transposase. Identical to
                     many other M. tuberculosis IS6110 transposase subunits. The transposase described
                     here may be made by a frame shifting mechanism during translation that fuses Rv2167c
                     and Rv2168c, the sequence UUUUAAAG (directly upstream of Rv2167c) maybe responsible
                     for such a frameshifting event (see McAdam et al., 1990). Start changed since first
                     submission (- 18 aa)."
                     /codon_start=1
                     /transl_table=11
                     /product="Probable transposase"
                     /protein_id="NP_216683.2"
                     /db_xref="GI:448824778 GeneID:888197"
                     /translation="KDRVGFLRGRARPASTLITRFIADHQGHREGPDGLRWGVESICTQLTELGVPIAPSTYYDHINREPSRREL
                     RDGELKEHISRVHAANYGVYGARKVWLTLNREGIEVARCTVERLMTKLGLSGTTRGKARRTTIADPATARPADLVQRRFGPPAPN
                     RLWVADLTYVSTWAGFAYVAFVTDAYARRILGWRVASTMATSMVLDAIEQAIWTRQQEGVLDLKDVIHHTDRGSQYTSIRFSERL
                     AEAGIQPSVGAVGSSYDNALAETINGLYKTELIKPGKPWRSIEDVELATARWVDWFNHRRLYQYCGDVPPVELEAAYYAQRQRPA
                     AG"
     CDS             complement(2271526..2271852)
                     /locus_tag="Rv2168c"
                     /note="Rv2168c, (MTV021.01c), len: 108 aa. Putative transposase for IS6110
                     (fragment), identical to many other Mycobacterium tuberculosis IS6110 transposase
                     subunits e.g. Q50686|YIA4_MYCTU Insertion element IS6110 hypothetical 12.0 kDa
                     protein (108 aa), fasta scores: E(): 1.4e-43,(100.00% identity in 108 aa overlap).
                     The transposase described here may be made by a frame shifting mechanism during
                     translation that fuses Rv2167c and Rv2168c, the sequence UUUUAAAG (directly upstream
                     of Rv2167c) maybe responsible for such a frameshifting event (see McAdam et al.,
                     1990)."
                     /codon_start=1
                     /transl_table=11
                     /product="Putative transposase for insertion sequence element IS6110 (fragment)"
                     /protein_id="NP_216684.1"
                     /db_xref="GI:15609305 GeneID:888459"
                     /translation="MSGGSSRRYPPELRERAVRMVAEIRGQHDSEWAAISEVARLLGVGCAETVRKWVRQAQVDAGARPGTTTEE
                     SAELKRLRRDNAELRRANAILKTASAFFAAELDRPAR"
     CDS             complement(2279714..2280379)
                     /locus_tag="Rv2177c"
                     /note="Rv2177c, (MTV021.10c), len: 221 aa. Possible IS1558 transposase (see citation
                     below), similar to several is element proteins and transposases but nearly identical
                     to last 221 residues of MTCY428_23 (333 aa). FASTA scores: Z81451|MTCY428_23
                     Mycobacterium tuberculosis cosmid (333 aa) opt: 1491, E() : 0; 98.6% identity in 221
                     aa overlap."
                     /codon_start=1
                     /transl_table=11
                     /product="Possible transposase"
                     /protein_id="NP_216693.1"
                     /db_xref="GI:15609314 GeneID:888326"
                     /translation="MRSKIPDLQRALEGRFDDHHALMCRLHLAHLDQLDAMIGALDEQIEQLMHPFCARRELIASIPGIGVGASA
                     TVISEIGADPAAWFPSAEHLASWVRLCPGNHESAGKRHHGARRTGNQHLQPVLVECAWAAVRTDGYLREYYRRQVRKFGGFRSPA
                     ANKKAIIAVAHKLIVIIWHVLATGRPYQDLGADYFTTRMDPDKERRRLVAKLEAQGLGVTLEPAA"
     CDS             2390497..2390823
                     /locus_tag="Rv2278"
                     /note="Rv2278, (MTCY339.32c), len: 108 aa. Putative Transposase for IS6110
                     (fragment). Identical to many other M. tuberculosis IS6110 transposase subunits. The
                     transposase described here may be made by a frame shifting mechanism during
                     translation that fuses Rv2278 and Rv2279,the sequence UUUUAAAG (directly upstream of
                     Rv2279) maybe responsible for such a frameshifting event (see McAdam et al., 1990)."
                     /codon_start=1
                     /transl_table=11
                     /product="Putative transposase for insertion sequence element IS6110 (fragment)"
                     /protein_id="NP_216794.1"
                     /db_xref="GI:15609415 GeneID:888602"
                     /translation="MSGGSSRRYPPELRERAVRMVAEIRGQHDSEWAAISEVARLLGVGCAETVRKWVRQAQVDAGARPGTTTEE
                     SAELKRLRRDNAELRRANAILKTASAFFAAELDRPAR"
     CDS             2390772..2391758
                     /locus_tag="Rv2279"
                     /note="Rv2279, (MTCY339.31c), len: 328 aa. Probable IS6110 transposase. Identical to
                     many other M. tuberculosis IS6110 transposase subunits. The transposase described
                     here may be made by a frame shifting mechanism during translation that fuses Rv2278
                     and Rv2279, the sequence UUUUAAAG (directly upstream of Rv2279) maybe responsible for
                     such a frameshifting event (see McAdam et al., 1990). Start changed since first
```

```
                     submission (+ 16 aa)."
                     /codon_start=1
                     /transl_table=11
                     /product="Probable transposase"
                     /protein_id="NP_216795.2"
                     /db_xref="GI:448824782 GeneID:887746"
                     /translation="KDRVGFLRGRARPASTLITRFIADHQGHREGPDGLRWGVESICTQLTELGVPIAPSTYYDHINREPSRREL
                     RDGELKEHISRVHAANYGVYGARKVWLTLNREGIEVARCTVERLMTKLGLSGTTRGKARRTTIADPATARPADLVQRRFGPPAPN
                     RLWVADLTYVSTWAGFAYVAFVTDAYARRILGWRVASTMATSMVLDAIEQAIWTRQQEGVLDLKDVIHHTDRGSQYTSIRFSERL
                     AEAGIQPSVGAVGSSYDNALAETINGLYKTELIKPGKPWRSIEDVELATARWVDWFNHRRLYQYCGDVPPVELEAAYYAQRQRPA
                     AG"
     CDS             2471428..2471754
                     /locus_tag="Rv2354"
                     /note="Rv2354, (MTCY98.23), len: 108 aa. Putative Transposase for IS6110 (fragment).
                     Identical to many other M. tuberculosis IS6110 transposase subunits. The transposase
                     described here may be made by a frame shifting mechanism during translation that
                     fuses Rv2354 and Rv2355,the sequence UUUUAAAG (directly upstream of Rv2355) maybe
                     responsible for such a frameshifting event (see McAdam et al., 1990)."
                     /codon_start=1
                     /transl_table=11
                     /product="Probable transposase for insertion sequence element IS6110 (fragment)"
                     /protein_id="NP_216870.1"
                     /db_xref="GI:15609491 GeneID:888963"
                     /translation="MSGGSSRRYPPELRERAVRMVAEIRGQHDSEWAAISEVARLLGVGCAETVRKWVRQAQVDAGARPGTTTEE
                     SAELKRLRRDNAELRRANAILKTASAFFAAELDRPAR"
     CDS             2471703..2472689
                     /locus_tag="Rv2355"
                     /note="Rv2355, (MTCY98.24), len: 328 aa. Probable IS6110 transposase. Identical to
                     many other M. tuberculosis IS6110 transposase subunits. The transposase described
                     here may be made by a frame shifting mechanism during translation that fuses Rv2354
                     and Rv2355, the sequence UUUUAAAG (directly upstream of Rv2355) maybe responsible for
                     such a frameshifting event (see McAdam et al., 1990). Start changed since first
                     submission (+ 16 aa)."
                     /codon_start=1
                     /transl_table=11
                     /product="Probable transposase"
                     /protein_id="NP_216871.2"
                     /db_xref="GI:448824783 GeneID:888957"
                     /translation="KDRVGFLRGRARPASTLITRFIADHQGHREGPDGLRWGVESICTQLTELGVPIAPSTYYDHINREPSRREL
                     RDGELKEHISRVHAANYGVYGARKVWLTLNREGIEVARCTVERLMTKLGLSGTTRGKARRTTIADPATARPADLVQRRFGPPAPN
                     RLWVADLTYVSTWAGFAYVAFVTDAYARRILGWRVASTMATSMVLDAIEQAIWTRQQEGVLDLKDVIHHTDRGSQYTSIRFSERL
                     AEAGIQPSVGAVGSSYDNALAETINGLYKTELIKPGKPWRSIEDVELATARWVDWFNHRRLYQYCGDVPPVELEAAYYAQRQRPA
                     AG"
     CDS             complement(2552736..2553737)
                     /locus_tag="Rv2424c"
                     /inference="protein motif:PROSITE:PS01159"
                     /note="Rv2424c, (MTCY428.23), len: 333 aa. Probable transposase for IS1558, similar
                     to is element proteins e.g. AL021957|Rv2177c|MTV021_10 from Mycobacterium
                     tuberculosis (221 aa), FASTA scores: opt: 1491, E(): 6.2e-87, (98.6% identity in 221
                     aa overlap); P19780|YIS1_STRCO hypothetical insertion element IS110 from Streptomyces
                     coelicolor (45 aa), FASTA scores: opt: 203, E(): 1.7e-05; (27.3% identity in 238 aa
                     overlap); etc. Contains PS01159 WW/rsp5/WWP domain signature."
                     /codon_start=1
                     /transl_table=11
                     /product="Probable transposase"
                     /protein_id="NP_216940.1"
                     /db_xref="GI:15609561 GeneID:885699"
                     /translation="MQCRAREERPGRKTDLLDAEWLVHLLECGLLRGWLIPPADIKAARDVIRYRRKLVEHRTSKLQRLGNVLQD
                     AGIKADSVASSVTPKSVRAMVEALIDGERRPAVLADLARGSMRSKIPDLQRALEGRFDDHHALMCRLHLAHLDQLDAMIGALDEQ
                     IEQLMHPFCARRELIASIPGIGVGASATVISEIGADPAAWFPSAEHLASWVRLCPGNHESAGKRHHGARRTGNQHLQPVLVECAW
                     AAVRTDGYLREYYRRQVRKFGGFRSPAANKKAITTVAHKLIVIIWHVLATGRPHQDLGADYFTTRMDPDKERRRLVAKLEAQGLG
                     VTLEPAA"
     CDS             complement(2615732..2616718)
                     /locus_tag="Rv2479c"
                     /note="Rv2479c, (MTV008.35c), len: 328 aa. Probable transposase for IS6110. Identical
                     to many other M. tuberculosis IS6110 transposase subunits. The transposase described
                     here may be made by a frame shifting mechanism during translation that fuses Rv2480c
                     and Rv2479c, the sequence UUUUAAAG (directly upstream of Rv2479c) maybe responsible
                     for such a frameshifting event (see McAdam et al., 1990). Start changed since first
```

```
                     submission (- 18 aa)."
                     /codon_start=1
                     /transl_table=11
                     /product="Probable transposase"
                     /protein_id="NP_216995.2"
                     /db_xref="GI:448824787 GeneID:887201"
                     /translation="KDRVGFLRGRARPASTLITRFIADHQGHREGPDGLRWGVESICTQLTELGVPIAPSTYYDHINREPSRREL
                     RDGELKEHISRVHAANYGVYGARKVWLTLNREGIEVARCTVERLMTKLGLSGTTRGKARRTTIADPATARPADLVQRRFGPPAPN
                     RLWVADLTYVSTWAGFAYVAFVTDAYARRILGWRVASTMATSMVLDAIEQAIWTRQQEGVLDLKDVIHHTDRGSQYTSIRFSERL
                     AEAGIQPSVGAVGSSYDNALAETINGLYKTELIKPGKPWRSIEDVELATARWVDWFNHRRLYQYCGDVPPVELEAAYYAQRQRPA
                     AG"
     CDS             complement(2616667..2616993)
                     /locus_tag="Rv2480c"
                     /note="Rv2480c, (MTV008.36c), len: 108 aa. Putative Transposase for IS6110
                     (fragment). Identical to many other M. tuberculosis IS6110 transposase subunits. The
                     transposase described here may be made by a frame shifting mechanism during
                     translation that fuses Rv2480c and Rv2479c, the sequence UUUUAAAG (directly upstream
                     of Rv2479c) maybe responsible for such a frameshifting event (see McAdam et al.,
                     1990)."
                     /codon_start=1
                     /transl_table=11
                     /product="Possible transposase for insertion sequence element IS6110 (fragment)"
                     /protein_id="NP_216996.1"
                     /db_xref="GI:15609617 GeneID:887328"
                     /translation="MSGGSSRRYPPELRERAVRMVAEIRGQHDSEWAAISEVARLLGVGCAETVRKWVRQAQVDAGARPGTTTEE
                     SAELKRLRRDNAELRRANAILKTASAFFAAELDRPAR"
     CDS             complement(2652563..2653810)
                     /locus_tag="Rv2512c"
                     /inference="protein motif:PROSITE:PS00435 protein motif:PROSITE:PS01007"
                     /note="Rv2512c, (MTCY07A7.18c), len: 415 aa. Transposase for IS1081, identical to
                     P35882|TRA1_MYCBO transposase for insertion sequence element IS1081 from
                     Mycobacterium bovis (415 aa), FASTA scores: opt: 2680, E(): 1.9e-162, (100.0%
                     identity in 415 aa overlap). Also highly similar to others from Mycobacterium
                     tuberculosis e.g. P96354|Rv1047|MTCY10G2.02c|Rv3115|MTCY164.25|Rv3023c|MTV01 2.38c
                     (415 aa), FASTA scores: opt: 2675, E(): 3.9e-162, (99.75% identity in 415 aa
                     overlap). Contains PS00435 Peroxidases proximal heme-ligand signature, PS01007
                     Transposases,Mutator family, signature. Belongs to the mutator family of transposase."
                     /codon_start=1
                     /transl_table=11
                     /product="Transposase for insertion sequence element IS1081"
                     /protein_id="NP_217028.1"
                     /db_xref="GI:15609649 GeneID:888515"
                     /translation="MTSSHLIDTEQLLADQLAQASPDLLRGLLSTFIAALMGAEADALCGAGYRERSDERSNQRNGYRHRDFDTR
                     AATIDVAIPKLRQGSYFPDWLLQRRKRAERALTSVVATCYLLGVSTRRMERLVETLGVTKLSKSQVSIMAKELDEAVEAFRTRPL
                     DAGPYTFLAADALVLKVREAGRVVGVHTLIATGVNAEGYREILGIQVTSAEDGAGWLAFFRDLVARGLSGVALVTSDAHAGLVAA
                     IGATLPAAAWQRCRTHYAANLMAATPKPSWPWVRTLLHSIYDQPDAESVVAQYDRVLDALTDKLPAVAEHLDTARTDLLAFTAFP
                     KQIWRQIWSNNPQERLNREVRRRTDVVGIFPDRASIIRLVGAVLAEQHDEWIEGRRYLGLEVLTRARAALTSTEEPAKQQTTNTP
                     ALTT"
     CDS             2787590..2787916
                     /locus_tag="Rv2648"
                     /note="Rv2648, (MTCY441.17A), len: 108 aa. Putative Transposase for IS6110
                     (fragment). Identical to many other M. tuberculosis IS6110 transposase subunits. The
                     transposase described here may be made by a frame shifting mechanism during
                     translation that fuses Rv2648 and Rv2649,the sequence UUUUAAAG (directly upstream of
                     Rv2649) maybe responsible for such a frameshifting event (see McAdam et al., 1990)."
                     /codon_start=1
                     /transl_table=11
                     /product="Probable transposase for insertion sequence element IS6110 (fragment)"
                     /protein_id="NP_217164.1"
                     /db_xref="GI:15609785 GeneID:887828"
                     /translation="MSGGSSRRYPPELRERAVRMVAEIRGQHDSEWAAISEVARLLGVGCAETVRKWVRQAQVDAGARPGTTTEE
                     SAELKRLRRDNAELRRANAILKTASAFFAAELDRPAR"
     CDS             2787865..2788851
                     /locus_tag="Rv2649"
                     /note="Rv2649, (MTCY441.18), len: 328 aa. Probable transposase for IS6110. Identical
                     to many other M. tuberculosis IS6110 transposase subunits. The transposase described
                     here may be made by a frame shifting mechanism during translation that fuses Rv2648
                     and Rv2649, the sequence UUUUAAAG (directly upstream of Rv2649) maybe responsible for
                     such a frameshifting event (see McAdam et al., 1990)."
```

```
                     /codon_start=1
                     /transl_table=11
                     /product="Probable transposase for insertion sequence element IS6110"
                     /protein_id="NP_217165.1"
                     /db_xref="GI:15609786 GeneID:888553"
                     /translation="KDRVGFLRGRARPASTLITRFIADHQGHREGPDGLRWGVESICTQLTELGVPIAPSTYYDHINREPSRREL
                     RDGELKEHISRVHAANYGVYGARKVWLTLNREGIEVARCTVERLMTKLGLSGTTRGKARRTTIADPATARPADLVQRRFGPPAPN
                     RLWVADLTYVSTWAGFAYVAFVTDAYARRILGWRVASTMATSMVLDAIEQAIWTRQQEGVLDLKDVIHHTDRGSQYTSIRFSERL
                     AEAGIQPSVGAVGSSYDNALAETINGLYKTELIKPGKPWRSIEDVELATARWVDWFNHRRLYQYCGDVPPVELEAAYYAQRQRPA
                     AG"
     CDS             2798501..2799304
                     /locus_tag="Rv2666"
                     /inference="protein motif:PROSITE:PS01007"
                     /note="Rv2666, (MTCY441.35), len: 267 aa. Probable transposase (fragment), identical
                     in region of overlap to P35882|TRA1_MYCBO|TRA1_MYCTU transposase for insertion
                     sequence element IS1081 from Mycobacterium tuberculosis or bovis (415 aa). Last 4
                     codons not part of gene. Contains PS01007 Transposases, Mutator family, signature."
                     /codon_start=1
                     /transl_table=11
                     /product="Probable transposase for insertion sequence element IS1081 (fragment)"
                     /protein_id="NP_217182.1"
                     /db_xref="GI:15609803 GeneID:888904"
                     /translation="MTSSHLIDTEQLLADQLAQASPDLLRGLLSTFIAALMGAEADALCGAGYRERSDERSNQRNGYRHRDFDTR
                     AATIDVAIPKLRQGSYFPDWLLQRRKRAERALTSVVATCYLLGVSTRRMERLVETLGVTKLSKSQVSIMAKELDEAVEAFRTRPL
                     DAGPYTFLAADALVLKVREAGRVVGVHTLIATGVNAEGYREILGIQVTSAEDGAGWLAFFRDLVARGLSGVALVTSDAHAGLVAA
                     IGATLPAAAWQRCRTHYAANHGRHNA"
     CDS             complement(2910892..2912271)
                     /locus_tag="Rv2791c"
                     /experiment="EXISTENCE: identified in proteomics study"
                     /note="Rv2791c, (MTV002.56c), len: 459 aa. Probable IS1602 transposase for IS1602
                     element, similar to many e.g. P95117|Rv2978c|MTCY349.09 from Mycobacterium
                     tuberculosis (459 aa), FASTA scores: opt: 2718, E(): 6.3e-165, (86.05% identity in
                     459 aa overlap)."
                     /codon_start=1
                     /transl_table=11
                     /product="Probable transposase"
                     /protein_id="NP_217307.1"
                     /db_xref="GI:15609928 GeneID:888281"
                     /translation="MAKFEIPEGWMVQAFRFTLDPTAEQARALARHFGARRKAYNWTVATLKADIDAWQATGIQTAKPSLRVLRK
                     RWNTVKNDVCVNIETGVVWWPECSKEAYADGIDGAVDAYWNWQNSRSGKRDGKRMGFPRFKKKGRDPDRVTFTTGAMRVEPDRRH
                     LTLPVIGTVRTHENTRRVERLIAKGRSRVLAITVRRNGTRIDASVRVLVQRPQQPKVTDPGSRVGVDVGVRRLATVATADGAVLE
                     RVPNPRPLDAALNELRHVCRARSRCTKGSRRYRERTTEISRLHRRVNDVRTHHLHCLTTHLAKTHGRIVVEGLDAAGMLRQQGLS
                     GARARRRGLSDAALGTPRRHLSYKTGWYGSQLVVADRWFPSSKTCHVCGHVQEIGWAEHWQCDSCSASHQRDDCAAINLARYEDT
                     SSVVGPVGAAVKRGADRKTRPGRAGGREARKGSSRKAAEQPRDGVQVA"
     CDS             complement(2926431..2926832)
                     /locus_tag="Rv2810c"
                     /note="Rv2810c, (MTCY16B7.33), len: 133 aa. Probable transposase for IS1555, similar
                     to C-terminal domain of transposases for defective IS1555 e.g. Q9LCS0|TNPA
                     transposase from Arthrobacter sp. TM1 (435 aa), FASTA scores: opt: 294, E(): 1.8e-13,
                     (55.1% identity in 98 aa overlap); Q50440|TNPA insertion element TNPR and TNPA gene
                     from Mycobacterium smegmatis (413 aa), FASTA scores: opt: 274, E(): 4.7e-12, (56.25%
                     identity in 96 aa overlap); etc. This region is a possible MT-complex-specific
                     genomic island (See Becq et al., 2007)."
                     /codon_start=1
                     /transl_table=11
                     /product="Probable transposase"
                     /protein_id="NP_217326.1"
                     /db_xref="GI:15609947 GeneID:887784"
                     /translation="PLRLQAHTGGPPVALRQETTGGPSPTNDLITEPPRHYKQQTRVRQAPALLTVSAGTGVPVVLEELAKLGRT
                     LWRCRHDVLAYFDHHASNGPTEAINGRLEALCRNALGFRNLTHYRIRSLLHCGNLAQLIHAL"
     CDS             2927508..2928917
                     /locus_tag="Rv2812"
                     /experiment="EXISTENCE: identified in proteomics study"
                     /note="Rv2812, (MTCY16B7.31c), len: 469 aa. Probable transposase for IS1604, similar
                     to putative transposases and hypothetical proteins e.g. Q9EZM2|putative transposase
                     from Mycobacterium paratuberculosis (395 aa), FASTA scores: opt: 329, E(): 3e-13,
                     (27.05% identity in 362 aa overlap); CAC46499 putative transposase protein from
                     Rhizobium meliloti (Sinorhizobium meliloti) (390 aa), FASTA scores: opt: 327, E():
                     3.9e-13, (30.5% identity in 367 aa overlap); etc. Contains possible helix-turn-helix
```

```
                     motif at aa 50-71 (Score 1140, +3.07 SD). This region is a possible
                     MT-complex-specific genomic island (See Becq et al.,2007)."
                     /codon_start=1
                     /transl_table=11
                     /product="Probable transposase"
                     /protein_id="NP_217328.1"
                     /db_xref="GI:15609949 GeneID:888942"
                     /translation="MAVGDDEEKVRAERARAIGLFRYQLIWEAADAAHSTKQRGKMVRELASREHTDPFGRRVRISRQTIDRWIR
                     GWRAGGFDALVPNPRQCTPRTPAEVLELAVALRRENPQRTAAAIRRILRTQLGWAPDERTLQRNFHRLGLTGATTGSAPAVFGRF
                     EAEHPNALWTGDVLHGIRIDLRKTYLFAFLDDHSRLVPGYRWGHAEDTVRLAAALRPALASRGVPNAVYVDNGSPYVDAWLLRAC
                     AKLGVRLVHSTPGRPQGRGKIERFFRTVREQFLVEITGEPDVVGRHYVADLAELNRLFTAWVETVYHRSVHSETGQTPLARWSAG
                     GPIPLPAPETLTEAFLWEEHRRVTKTATVSLHGNRYEIDPALVGRKVELVFDPFDLTRIEVRLAGAPMRRAIPYHIGRHSHPKAK
                     PETPTAPPKPSGIDYAQLIETAHAAELARGVNYTALTGAADQIPGQLDLLTGQEAQPK"
CDS                  complement(2931256..2932242)
                     /locus_tag="Rv2814c"
                     /note="Rv2814c, (MTCY16B7.29), len: 328 aa. Probable transposase subunit for IS6110.
                     Identical to many other M. tuberculosis IS6110 transposase subunits. The transposase
                     described here may be made by a frame shifting mechanism during translation that
                     fuses Rv2814c and Rv2815c, the sequence UUUUAAAG (directly upstream of Rv2814c) maybe
                     responsible for such a frameshifting event (see McAdam et al., 1990). Start changed
                     since first submission (+ 16 aa). This region is a possible MT-complex-specific
                     genomic island (See Becq et al., 2007)."
                     /codon_start=1
                     /transl_table=11
                     /product="Probable transposase"
                     /protein_id="NP_217330.2"
                     /db_xref="GI:448824791 GeneID:887839"
                     /translation="KDRVGFLRGRARPASTLITRFIADHQGHREGPDGLRWGVESICTQLTELGVPIAPSTYYDHINREPSRREL
                     RDGELKEHISRVHAANYGVYGARKVWLTLNREGIEVARCTVERLMTKLGLSGTTRGKARRTTIADPATARPADLVQRRFGPPAPN
                     RLWVADLTYVSTWAGFAYVAFVTDAYARRILGWRVASTMATSMVLDAIEQAIWTRQQEGVLDLKDVIHHTDRGSQYTSIRFSERL
                     AEAGIQPSVGAVGSSYDNALAETINGLYKTELIKPGKPWRSIEDVELATARWVDWFNHRRLYQYCGDVPPVELEAAYYAQRQRPA
                     AG"
CDS                  complement(2932191..2932517)
                     /locus_tag="Rv2815c"
                     /note="Rv2815c, (MTCY16B7.28), len: 108 aa. Putative Transposase for IS6110
                     (fragment). Identical to many other M. tuberculosis IS6110 transposase subunits. The
                     transposase described here may be made by a frame shifting mechanism during
                     translation that fuses Rv2814c and Rv2815c, the sequence UUUUAAAG (directly upstream
                     of Rv2814c) maybe responsible for such a frameshifting event (see McAdam et al.,
                     1990). This region is a possible MT-complex-specific genomic island (See Becq et
                     al.,2007)."
                     /codon_start=1
                     /transl_table=11
                     /product="Probable transposase"
                     /protein_id="NP_217331.1"
                     /db_xref="GI:15609952 GeneID:888511"
                     /translation="MSGGSSRRYPPELRERAVRMVAEIRGQHDSEWAAISEVARLLGVGCAETVRKWVRQAQVDAGARPGTTTEE
                     SAELKRLRRDNAELRRANAILKTASAFFAAELDRPAR"
CDS                  complement(3003008..3004390)
                     /locus_tag="Rv2885c"
                     /experiment="EXISTENCE: identified in proteomics study"
                     /inference="protein motif:PROSITE:PS00017"
                     /note="Rv2885c, (MTCY274.16c), len: 460 aa. Probable transposase for IS1539. Contains
                     PS00017 ATP/GTP-binding site motif A (P-loop)."
                     /codon_start=1
                     /transl_table=11
                     /product="Probable transposase"
                     /protein_id="NP_217401.1"
                     /db_xref="GI:15610022 GeneID:887173"
                     /translation="MMARLKVPEGWCVQAFRFTLNPTQTQAASLARHFGARRKAFNWTVTALKADIKAWRADGTESAKPSLRVLR
                     KRWNTVKDQVCVNAQTGQVWWPECSKEAYADGIAGAVDAYWNWQSCRAGKRAGKTVGVPRFKKKGRDADRVCFTTGAMRVEPDRR
                     HLTLPVIGTIRTYENTRRVERLIAKGRARVLAITVRRNGTRLDASVRVLVQRPQQRRVALPDSRVGVDVGVRRLATVADAEGTVL
                     EQVPNPRPLDAALRGLRRVSRARSRCTKGSRRYCERTTELSRLHRRVNDVRTHHLHVLTTRLAKTHGRIVVEGLDAAGMLRQKGL
                     PGARARRRALSDAALATPRRHLSYKTGWYGSSLVVADRWFPSSKTCHACRHVQDIGWDEKWQCDGCSITHQRDDNAAINLARYEE
                     PPSVVGPVGAAVKRGADRKTGPGPAGGREARKATGHPAGEQPRDGVQVK"
CDS                  3096079..3097320
                     /locus_tag="Rv2943"
                     /note="Rv2943, (MTCY24G1.06c), len: 413 aa. Probable transposase for insertion
                     sequence IS1533, similar to other transposases e.g. P15025|ISTA_ECOLI ista protein
```

(insertion sequence IS21) from Escherichia coli (390 aa), FASTA scores: opt: 268, E(): 5.1e-11, (24.1% identity in 378 aa overlap). Contains potential helix-turn-helix motif at aa 19-40 (Score 1611, +4.67 SD)."
/codon_start=1
/transl_table=11
/product="Probable transposase for insertion sequence element IS1533"
/protein_id="NP_217459.1"
/db_xref="GI:15610080 GeneID:887834"
/translation="MLTVEDWAEIRRLHRAEGLPIKMIARVLGISKNTVKSALESNQQPKYERAPQGSIVDAVEPRIRELLQAYPTMPATVIAERIGWERSIRVLSARVAELRPVYLPPDPASRTTYVAGEIAQCDFWFPPIELPVGFGQTRTAKQLPVLTMVCAYSRWLLAMLLPSRCAEDLFAGWWRLIEALGAVPRVLVWDGEGAIGRWRGGRSELTTECQAFRGTLAAKVLICRPADPEAKGLIERAHDYLERSFLPGRVFASPADFNAQLGAWLALVNTRTRRALGCAPTDRIGADRAAMLSLPPVAPATGWCTSLRLPRDHYVRCDSNDYSVHPGVIGHRVLVRADLERVHVFCDGELVADHERIWAVHQTVSDPAHVEAAKVLRRRHFSAASPVVEPQVQVRSLSDYDDALGVDIDGGVA"

CDS 3097320..3097850
/locus_tag="Rv2943A"
/note="Rv2943A, len: 176 aa. Possible transposase, similar to many e.g. AJ238712|MBO238712_2 putative transposase (IS21-l) from Mycobacterium bovis BCG (266 aa), FASTA scores: opt: 762, E(): 0, (100.0% identity in 118 aa overlap). Possible frameshift after codon 118 i.e. near position 3290056, to fuse with Rv2944."
/codon_start=1
/transl_table=11
/product="Possible transposase"
/protein_id="YP_177680.1"
/db_xref="GI:57117039 GeneID:3205061"
/translation="MPTTKATQRRDVSTEIAYLTRALKAPTLRESVSRLADRARAENWSHEEYLAACLQREVSARESHGGEGRIRAARFPARKSLEEFDFEHARGLKRDTIAHLGTLDFITARDNVVFLGPAWHREDSSCGRPGDTRVSGRSSGAVRHRRRMGSTARRGSPRRAHLRRTHPALPLSAPGG"

CDS 3097405..3098121
/locus_tag="Rv2944"
/inference="protein motif:PROSITE:PS00017"
/note="Rv2944, (MTCY24G1.05c), len: 238 aa. Possible transposase for IS1533, similar to is-element proteins e.g. P15026|ISTB_ECOLI istb protein from Escherichia coli (265 aa), FASTA scores: opt: 475, E (): 1.6e-21, (48.0% identity in 148 aa overlap); Z95436|MTY15C10_14 from Mycobacterium tuberculosis (248 aa), FASTA scores: opt: 784, E(): 0,(87.4% identity in 135 aa overlap). Contains PS00017 ATP/GTP-binding site motif A (P-loop)."
/codon_start=1
/transl_table=11
/product="Possible transposase for insertion sequence element IS1533"
/protein_id="NP_217460.1"
/db_xref="GI:15610081 GeneID:887636"
/translation="MSQCPGWPIAPAPRTGATKNTWPPACSGKCQPGSPMVVRAASAPPASRLGSRWKSSTLSMLVASNATPSHIWAPWISSPPAITSCFWAPPGTGKTHLAVGLAIRACQAGHRVLFATAAEWVARLAEAHHAGRIYAELTRLCRYPLLVVDEVGYIPFEPEAANLFFQLVSSRYERASLIVTSNKAFGRWGEVFGGDDVVAAAMIDRLVHHAEVVALKGDSYRLKDRDLGRVPPAGTTEE"

CDS 3120898..3121287
/locus_tag="Rv2961"
/experiment="EXISTENCE: identified in proteomics study"
/note="Rv2961, (MTCY349.26c), len: 129 aa. Probable transposase, highly similar to C-terminus of O50414|Rv3387|MTV004.45 putative transposase from Mycobacterium tuberculosis (225 aa), FASTA scores: opt: 605, E(): 7.2e-34, (66.65% identity in 129 aa overlap); and similar to others e.g. CAC47401 putative partial transposase for ISRM17 protein from Rhizobium meliloti (Sinorhizobium meliloti) (174 aa), FASTA scores: opt: 183,E(): 2.6e-05, (30.25% identity in 129 aa overlap); etc. This region is a possible MT-complex-specific genomic island (See Becq et al., 2007)."
/codon_start=1
/transl_table=11
/product="Probable transposase"
/protein_id="NP_217477.1"
/db_xref="GI:15610098 GeneID:887316"
/translation="MEHGNPHDAPQLAPAVERITTRAGRPPGTVTADRGYGEKRVEDDLHDLGVRTVAIPRKGRPSQARRAEEQRPSFRRTVKWRTGSEGRISTLKRNYGWNRSCIDGTEGTRIWTRHGILTHNLIKISSLAA"

CDS complement(3141400..3142779)
/locus_tag="Rv2978c"
/experiment="EXISTENCE: identified in proteomics study"
/note="Rv2978c, (MTCY349.09), len: 459 aa. Probable transposase for IS1538, very similar to several other putative transposases from Mycobacterium tuberculosis e.g. YX16_MYCTU|Q10809 (460 aa), FASTA scores: opt: 2613, E(): 0, (83.0% identity in 458 aa overlap); etc. Low level matches to other tranposases."

```
                     /codon_start=1
                     /transl_table=11
                     /product="Probable transposase"
                     /protein_id="NP_217494.1"
                     /db_xref="GI:15610115 GeneID:887390"
                     /translation="MPKFEVPDGWTVQAFRFTLDPTEDQAKALARHFGARRKAYNWTVATLKADIQAWHASGTVTAKPSLRVLRK
                     RWNTVKDDVCVNTETGVAWWPECSKEAYADGIAGAVEAYWNWQTSRAGKRAGKRVGFPRFKRKGRDQDRVSFTTGAMRVEPDRRH
                     LTLPVIGTVRTHENTRRIERLIKAGRARVLAISVRRNGTRLDASVRVLVQRPQQPKVVHPGSRVGVDVGVRRLATVATADGTAIE
                     QVENPRPLGAALRELRHVCRARSRCTKGSRRYRERTTQISRLHRRVNDVRTHHLHVLTTRLAQTHGRIVVEGLDATEMLRQKGLP
                     GARARRRGLSDAALGTPRRHLSYKTVWYGSALVVADRWFPSSKTCHACRHVQDIGWDEQWQCDRCSVVHQRDDCAAINLARYEET
                     SSIVGPVGAAVKRGADRKTGPRPAGGCEARKGSSPKAAEQPRDGVQVA"
     CDS             complement(3187283..3188530)
                     /locus_tag="Rv3023c"
                     /inference="protein motif:PROSITE:PS01007"
                     /note="Rv3023c, (MTV012.38c), len: 415 aa. Probable IS1081 transposase. Contains
                     PS01007 Transposases, Mutator family,signature. Similars to
                     P35882|TRA1_MYCTU|Rv1199c|MTCI364.11c and Rv2512c|MTCY07A7.18c transposases for
                     insertion sequence element IS1081 (415 aa), FASTA scores: opt: 2675, E(): 1.8e-162,
                     (100.0% identity in 415 aa overlap). Belongs to the mutator family of transposase."
                     /codon_start=1
                     /transl_table=11
                     /product="Probable transposase"
                     /protein_id="NP_217539.1"
                     /db_xref="GI:15610160 GeneID:888525"
                     /translation="MTSSHLIDAEQLLADQLAQASPDLLRGLLSTFIAALMGAEADALCGAGYRERSDERSNQRNGYRHRDFDTR
                     AATIDVAIPKLRQGSYFPDWLLQRRKRAERALTSVVATCYLLGVSTRRMERLVETLGVTKLSKSQVSIMAKELDEAVEAFRTRPL
                     DAGPYTFLAADALVLKVREAGRVVGVHTLIATGVNAEGYREILGIQVTSAEDGAGWLAFFRDLVARGLSGVALVTSDAHAGLVAA
                     IGATLPAAAWQRCRTHYAANLMAATPKPSWPWVRTLLHSIYDQPDAESVVAQYDRVLDALTDKLPAVAEHLDTARTDLLAFTAFP
                     KQIWRQIWSNNPQERLNREVRRRTDVVGIFPDRASIIRLVGAVLAEQHDEWIEGRRYLGLEVLTRARAALTSTEEPAKQQTTNTP
                     ALTT"
     CDS             3287359..3288606
                     /locus_tag="Rv3115"
                     /inference="protein motif:PROSITE:PS01007"
                     /note="Rv3115, (MTCY164.25), len: 415 aa. Probable IS1081 transposase, similar to
                     others. Has transposases, mutator family, signature (PS01007). Other copies are
                     MTCY10G2.02c,MTCY441.35, MTCY77.03c. This region is a possible MT-complex-specific
                     genomic island (See Becq et al.,2007)."
                     /codon_start=1
                     /transl_table=11
                     /product="Probable transposase"
                     /protein_id="NP_217631.1"
                     /db_xref="GI:15610252 GeneID:888790"
                     /translation="MTSSHLIDAEQLLADQLAQASPDLLRGLLSTFIAALMGAEADALCGAGYRERSDERSNQRNGYRHRDFDTR
                     AATIDVAIPKLRQGSYFPDWLLQRRKRAERALTSVVATCYLLGVSTRRMERLVETLGVTKLSKSQVSIMAKELDEAVEAFRTRPL
                     DAGPYTFLAADALVLKVREAGRVVGVHTLIATGVNAEGYREILGIQVTSAEDGAGWLAFFRDLVARGLSGVALVTSDAHAGLVAA
                     IGATLPAAAWQRCRTHYAANLMAATPKPSWPWVRTLLHSIYDQPDAESVVAQYDRVLDALTDKLPAVAEHLDTARTDLLAFTAFP
                     KQIWRQIWSNNPQERLNREVRRRTDVVGIFPDRASIIRLVGAVLAEQHDEWIEGRRYLGLEVLTRARAALTSTEEPAKQQTTNTP
                     ALTT"
     CDS             3351468..3351794
                     /locus_tag="Rv3184"
                     /note="Rv3184, (MTV014.28), len: 108 aa. Putative Transposase for IS6110 (fragment).
                     Identical to many other M. tuberculosis IS6110 transposase subunits. The transposase
                     described here may be made by a frame shifting mechanism during translation that
                     fuses Rv3184 and Rv3185,the sequence UUUUAAAG (directly upstream of Rv3185) maybe
                     responsible for such a frameshifting event (see McAdam et al., 1990). This region is
                     a possible MT-complex-specific genomic island (See Becq et al., 2007)."
                     /codon_start=1
                     /transl_table=11
                     /product="Probable transposase for insertion sequence element IS6110 (fragment)"
                     /protein_id="NP_217700.1"
                     /db_xref="GI:15610320 GeneID:888796"
                     /translation="MSGGSSRRYPPELRERAVRMVAEIRGQHDSEWAAISEVARLLGVGCAETVRKWVRQAQVDAGARPGTTTEE
                     SAELKRLRRDNAELRRANAILKTASAFFAAELDRPAR"
     CDS             3351743..3352729
                     /locus_tag="Rv3185"
                     /note="Rv3185, (MTV014.29), len: 328 aa. Probable IS6110 transposase. Identical to
                     many other M. tuberculosis IS6110 transposase subunits. The transposase described
                     here may be made by a frame shifting mechanism during translation that fuses Rv3184
                     and Rv3185, the sequence UUUUAAAG (directly upstream of Rv3185) maybe responsible for
```

```
                     such a frameshifting event (see McAdam et al., 1990). Start changed since first
                     submission (+ 16 aa). This region is a possible MT-complex-specific genomic island
                     (See Becq et al., 2007)."
                     /codon_start=1
                     /transl_table=11
                     /product="Probable transposase"
                     /protein_id="NP_217701.2"
                     /db_xref="GI:448824801 GeneID:887441"
                     /translation="KDRVGFLRGRARPASTLITRFIADHQGHREGPDGLRWGVESICTQLTELGVPIAPSTYYDHINREPSRREL
                     RDGELKEHISRVHAANYGVYGARKVWLTLNREGIEVARCTVERLMTKLGLSGTTRGKARRTTIADPATARPADLVQRRFGPPAPN
                     RLWVADLTYVSTWAGFAYVAFVTDAYARRILGWRVASTMATSMVLDAIEQAIWTRQQEGVLDLKDVIHHTDRGSQYTSIRFSERL
                     AEAGIQPSVGAVGSSYDNALAETINGLYKTELIKPGKPWRSIEDVELATARWVDWFNHRRLYQYCGDVPPVELEAAYYAQRQRPA
                     AG"
     CDS             3352951..3353277
                     /locus_tag="Rv3186"
                     /note="Rv3186, (MTV014.30), len: 108 aa. Putative Transposase for IS6110 (fragment).
                     Identical to many other M. tuberculosis IS6110 transposase subunits. The transposase
                     described here may be made by a frame shifting mechanism during translation that
                     fuses Rv3186 and Rv3187,the sequence UUUUAAAG (directly upstream of Rv3187) maybe
                     responsible for such a frameshifting event (see McAdam et al., 1990). This region is
                     a possible MT-complex-specific genomic island (See Becq et al., 2007)."
                     /codon_start=1
                     /transl_table=11
                     /product="Probable transposase for insertion sequence element IS6110 (fragment)"
                     /protein_id="NP_217702.1"
                     /db_xref="GI:15610322 GeneID:888024"
                     /translation="MSGGSSRRYPPELRERAVRMVAEIRGQHDSEWAAISEVARLLGVGCAETVRKWVRQAQVDAGARPGTTTEE
                     SAELKRLRRDNAELRRANAILKTASAFFAAELDRPAR"
     CDS             3353226..3354212
                     /locus_tag="Rv3187"
                     /note="Rv3187, (MTV014.31), len: 328 aa. Probable IS6110 transposase. Identical to
                     many other M. tuberculosis IS6110 transposase subunits. The transposase described
                     here may be made by a frame shifting mechanism during translation that fuses Rv3186
                     and Rv3187, the sequence UUUUAAAG (directly upstream of Rv3187) maybe responsible for
                     such a frameshifting event (see McAdam et al., 1990). Start changed since first
                     submission (+ 16 aa). This region is a possible MT-complex-specific genomic island
                     (See Becq et al., 2007)."
                     /codon_start=1
                     /transl_table=11
                     /product="Probable transposase"
                     /protein_id="NP_217703.2"
                     /db_xref="GI:448824802 GeneID:887604"
                     /translation="KDRVGFLRGRARPASTLITRFIADHQGHREGPDGLRWGVESICTQLTELGVPIAPSTYYDHINREPSRREL
                     RDGELKEHISRVHAANYGVYGARKVWLTLNREGIEVARCTVERLMTKLGLSGTTRGKARRTTIADPATARPADLVQRRFGPPAPN
                     RLWVADLTYVSTWAGFAYVAFVTDAYARRILGWRVASTMATSMVLDAIEQAIWTRQQEGVLDLKDVIHHTDRGSQYTSIRFSERL
                     AEAGIQPSVGAVGSSYDNALAETINGLYKTELIKPGKPWRSIEDVELATARWVDWFNHRRLYQYCGDVPPVELEAAYYAQRQRPA
                     AG"
     CDS             complement(3357498..3358532)
                     /locus_tag="Rv3191c"
                     /experiment="EXISTENCE: identified in proteomics study"
                     /note="Rv3191c, (MTV014.35c), len: 344 aa. Probable transposase, similar to many
                     especially Q9K2N8 putative transposase from Pseudomonas aeruginosa (338 aa), FASTA
                     scores: opt: 837, E(): 1.3e-43, (42.55% identity in 336 aa overlap); Q9RBF4 insertion
                     sequence IS1088 from Alcaligenes eutrophus (Ralstonia eutropha) (342 aa), FASTA
                     scores: opt: 823, E(): 9.2e-43, (43.05% identity in 337 aa overlap); and Q51379
                     putative transposase from Pseudomonas alcaligenes (338 aa), FASTA scores: opt: 818,
                     E(): 1.8e-42, (42.35% identity in 333 aa overlap). Contains probable helix-turn-helix
                     motif from aa 25 to 46 (Score 1968, +5.89 SD). This region is a possible
                     MT-complex-specific genomic island (See Becq et al., 2007)."
                     /codon_start=1
                     /transl_table=11
                     /product="Probable transposase"
                     /protein_id="NP_217707.1"
                     /db_xref="GI:15610327 GeneID:887628"
                     /translation="MRQISSRYLSEEERINIADLRRSGLSIRKIADQLGRAPSTVSRELRRNSRRDGQYRPFEAHRWAVQRRVRR
                     HRRRIDKNPDLCELIAELLAQRWSPQQIARHLRRKYPDDRSMWLCHESIYQAVYQPQSRLIRPPQVKSPHRGPLRTGRTHRRAHL
                     RPGRRRPRFAQPMLSIHQRPFDPADRSEPGHWEGDLIVGKNQGSAIGTLVERQTRLIRLLHLPTHDAYCLRIAITETMSDLPVTL
                     VRSITWDQGIEMARHIDITADLGAPVYFCDSRSPWQRASNENSNGLLRQYFPKGTSLSTYTPDHLRAVEYEINNRPRQVLGHRSP
                     AELFTALLTSPDHQLLRR"
```

```
     CDS             3510620..3510946
                     /locus_tag="Rv3325"
                     /note="Rv3325, (MTV016.25), len: 108 aa. Putative Transposase for IS6110 (fragment).
                     Identical to many other M. tuberculosis IS6110 transposase subunits. The transposase
                     described here may be made by a frame shifting mechanism during translation that
                     fuses Rv3325 and Rv3326,the sequence UUUUAAAG (directly upstream of Rv3326) maybe
                     responsible for such a frameshifting event (see McAdam et al., 1990). Belongs to the
                     transposase family 8."
                     /codon_start=1
                     /transl_table=11
                     /product="Probable transposase for insertion sequence element IS6110 (fragment)"
                     /protein_id="NP_217842.1"
                     /db_xref="GI:15610461 GeneID:887314"
                     /translation="MSGGSSRRYPPELRERAVRMVAEIRGQHDSEWAAISEVARLLGVGCAETVRKWVRQAQVDAGARPGTTTEE
                     SAELKRLRRDNAELRRANAILKTASAFFAAELDRPAR"
     CDS             3510895..3511881
                     /locus_tag="Rv3326"
                     /note="Rv3326, (MTV016.26), len: 328 aa. Probable transposase for insertion element
                     IS6110. Identical to many other M. tuberculosis IS6110 transposase subunits. The
                     transposase described here may be made by a frame shifting mechanism during
                     translation that fuses Rv3325 and Rv3326,the sequence UUUUAAAG (directly upstream of
                     Rv3326) maybe responsible for such a frameshifting event (see McAdam et al., 1990).
                     Start changed since first submission (+ 16 aa)."
                     /codon_start=1
                     /transl_table=11
                     /product="Probable transposase"
                     /protein_id="NP_217843.2"
                     /db_xref="GI:448824808 GeneID:887563"
                     /translation="KDRVGFLRGRARPASTLITRFIADHQGHREGPDGLRWGVESICTQLTELGVPIAPSTYYDHINREPSRREL
                     RDGELKEHISRVHAANYGVYGARKVWLTLNREGIEVARCTVERLMTKLGLSGTTRGKARRTTIADPATARPADLVQRRFGPPAPN
                     RLWVADLTYVSTWAGFAYVAFVTDAYARRILGWRVASTMATSMVLDAIEQAIWTRQQEGVLDLKDVIHHTDRGSQYTSIRFSERL
                     AEAGIQPSVGAVGSSYDNALAETINGLYKTELIKPGKPWRSIEDVELATARWVDWFNHRRLYQYCGDVPPVELEAAYYAQRQRPA
                     AG"
     CDS             3531115..3531606
                     /locus_tag="Rv3348"
                     /note="Rv3348, (MTV004.04), len: 163 aa. Probable transposase, partially similar to
                     several insertion elements e.g. P19834|YI11_STRCL insertion element IS116
                     hypothetical 44.8 KDA protein (similar to IS900 of Mycobacterium paratuberculosis)
                     from Streptomyces clavuligerus (399 aa), FASTA scores: opt: 146, E(): 0.016,(29.1%
                     identity in 158 aa overlap)."
                     /codon_start=1
                     /transl_table=11
                     /product="Probable transposase"
                     /protein_id="NP_217865.1"
                     /db_xref="GI:15610484 GeneID:888110"
                     /translation="MTAENPGRSRRTLVGIDAAITACHHIAIRDDVGARSIRFSVEPTLAGLRTLTDKLSGYDDIDATVEPTSMT
                     WLPLTIAVENAGDTMHMAGARHCARLRGAIVGKSKSDVIDAEVLTRASEVFDLTPLTLPTPAQLALRRSVIRRAGAVIDANRSWR
                     RLMSLAR"
     CDS             complement(3531643..3532383)
                     /locus_tag="Rv3349c"
                     /experiment="EXISTENCE: identified in proteomics study"
                     /note="Rv3349c, (MTV004.05c), len: 246 aa. Probable transposase pseudogene fragment,
                     similar to part of Q50911|U10634 IS204 putative transposase from nocardia asteroides
                     (377 aa), FASTA scores: opt: 288, E(): 8.3e-11,(48.5% identity in 97 aa overlap); and
                     others."
                     /codon_start=1
                     /transl_table=11
                     /product="Probable transposase"
                     /protein_id="NP_217866.1"
                     /db_xref="GI:15610485 GeneID:888126"
                     /translation="MAIDPAAAYASAIRTPGLLPNAKLVVDHFHVTTLANDALTAVRRRVTWAFHDRRGRKIDPQWANRRRLLTA
                     RERLSDKSFAKMRNRINAVDPRAQILSAWIAKEELRTLLSTVRTGGDPHLARHHLHRFLPGASTRRSPNCSPWPPPLTSHPRSTP
                     SWSPASPTRASVVGEVAEMLGDIDGQCVQVEVPVPERGPAGCGGLDGLGRAGVSATPRVCAAMTAVNVAGRCAGQQADVGPTPQH
                     RCRGR"
     CDS             complement(3559532..3560518)
                     /locus_tag="Rv3380c"
                     /note="Rv3380c, (MTV004.38c), len: 328 aa. Probable transposase subunit for IS6110.
                     Identical to many other M. tuberculosis IS6110 transposase subunits. The transposase
                     described here may be made by a frame shifting mechanism during translation that
```

```
                     fuses Rv3380c and Rv3381c, the sequence UUUUAAAG (directly upstream of Rv3380c) maybe
                     responsible for such a frameshifting event (see McAdam et al., 1990). Start changed
                     since first submission (+ 34 aa)."
                     /codon_start=1
                     /transl_table=11
                     /product="Probable transposase"
                     /protein_id="NP_217897.2"
                     /db_xref="GI:448824811 GeneID:887411"
                     /translation="KDRVGFLRGRARPASTLITRFIADHQGHREGPDGLRWGVESICTQLTELGVPIAPSTYYDHINREPSRREL
                     RDGELKEHISRVHAANYGVYGARKVWLTLNREGIEVARCTVERLMTKLGLSGTTRGKARRTTIADPATARPADLVQRRFGPPAPN
                     RLWVADLTYVSTWAGFAYVAFVTDAYARRILGWRVASTMATSMVLDAIEQAIWTRQQEGVLDLKDVIHHTDRGSQYTSIRFSERL
                     AEAGIQPSVGAVGSSYDNALAETINGLYKTELIKPGKPWRSIEDVELATARWVDWFNHRRLYQYCGDVPPVELEAAYYAQRQRPA
                     AG"
     CDS             complement(3560467..3560793)
                     /locus_tag="Rv3381c"
                     /note="Rv3381c, (MTV004.39c), len: 108 aa. Putative Transposase for IS6110
                     (fragment). Identical to many other M. tuberculosis IS6110 transposase subunits. The
                     transposase described here may be made by a frame shifting mechanism during
                     translation that fuses Rv3380c and Rv3381c, the sequence UUUUAAAG (directly upstream
                     of Rv3380c) maybe responsible for such a frameshifting event (see McAdam et al.,
                     1990)."
                     /codon_start=1
                     /transl_table=11
                     /product="Probable transposase for insertion sequence element IS6110 (fragment)"
                     /protein_id="NP_217898.1"
                     /db_xref="GI:15610517 GeneID:887646"
                     /translation="MSGGSSRRYPPELRERAVRMVAEIRGQHDSEWAAISEVARLLGVGCAETVRKWVRQAQVDAGARPGTTTEE
                     SAELKRLRRDNAELRRANAILKTASAFFAAELDRPAR"
     CDS             3564524..3565228
                     /locus_tag="Rv3386"
                     /experiment="EXISTENCE: identified in proteomics study"
                     /note="Rv3386, (MTV004.44), len: 234 aa. Possible transposase, showing very weak
                     similarity to several is element transposases. Highly similar (but shorter) to
                     P963659|MTCY10G2_13|Rv1036c from Mycobacterium tuberculosis (112 aa), FASTA scores:
                     opt: 507, E(): 8.3e-25, (83.9% identity in 87 aa overlap)."
                     /codon_start=1
                     /transl_table=11
                     /product="Possible transposase"
                     /protein_id="NP_217903.1"
                     /db_xref="GI:15610522 GeneID:888044"
                     /translation="MFRTVGDQASLWESVLPEELRRLPEELARVDALLDDSAFFCPFVPFFDPRMGRPSIPMETYLRLMFLKFRY
                     RLGYESLCREVTDSITWRRFCRIPLEGSVPHPTTLMKLTTRCGEDAVAGLNEALLAKAASEKLLRTNKVRADTTVVEGDVGYPTD
                     TGLLAKAVGSMARTVARIKAADAGSAPLGGSSGPRDRLQAAVTRRAATRSGAGLRAPDHRGASRDRRAGADRGCRGGT"
     CDS             3565218..3565895
                     /locus_tag="Rv3387"
                     /note="Rv3387, (MTV004.45), len: 225 aa. Possible transposase, showing very weak
                     similarity to other is element proteins, and similar to various hypothetical
                     proteins."
                     /codon_start=1
                     /transl_table=11
                     /product="Possible transposase"
                     /protein_id="NP_217904.1"
                     /db_xref="GI:15610523 GeneID:887820"
                     /translation="MVRNAQRAVRRASGRRKAWLRQAINHLEKLIGRTERVVDQARSRLAGVMPDSSSRLVSLHDADARPIRKGR
                     LGKPVEFGYKAQVVDNADGVILDHSVELGNPADAPQLAPAIERISRRTGRPPRAVTADRGCGDASVEDDLHQLGVRNVAIPRKSK
                     PSATRRAFEHRRAFRDKIKWRTGSEGRINHLKRSYGWNRTELTGITGARTWCGHGVFAHNLVKISTLAA"
     CDS             complement(3604891..3605646)
                     /locus_tag="Rv3427c"
                     /experiment="EXISTENCE: identified in proteomics study"
                     /inference="protein motif:PROSITE:PS00017"
                     /note="Rv3427c, (MTCY78.02), len: 251 aa. Possible transposase, similar to other e.g.
                     Q9APG8|ORF2 putative transposase subunit 2 from Pseudomonas putida (251 aa),FASTA
                     scores: opt: 479, E(): 1.8e-21, (34.85% identity in 238 aa overlap). Contains PS00017
                     ATP/GTP-binding site motif A."
                     /codon_start=1
                     /transl_table=11
                     /product="Possible transposase"
                     /protein_id="NP_217944.1"
                     /db_xref="GI:15610563 GeneID:887631"
```

```
                     /translation="MSICDPALRNALRTLKLSGMLDTLDARLAQTRNGDLGHLEFLQALREDEIARRESAALTRRLRRAKFEAQA
                     TFEDFDFTANPKLPGAMLRDLAALRWLDAGESVILHGPVGVGKTHVAQALVHAVARRGGDVRFAKTSRMLSDLAGGHADRSWGQR
                     IREYTKPLVLILDDFAMREHTAMHADDLYELISDRAITGKPLILTSNRAPNNWYGLFPNPVVAESLLDRLINTSHQILMDGPSYR
                     PRKRPGRTTS"
     CDS             complement(3605744..3606976)
                     /locus_tag="Rv3428c"
                     /experiment="EXISTENCE: identified in proteomics study"
                     /note="Rv3428c, (MTCY78.01, len: 410 aa. Possible transposase insertion sequence,
                     similar to others e.g. Q9APG9|ORF1 from Pseudomonas putida (509 aa), FASTA scores:
                     opt: 578, E(): 1.1e-29, (32.45% identity in 376 aa overlap); P55379|Y4BL_RHISN from
                     Rhizobium sp. strain NGR234 (516 aa), FASTA scores: opt: 665, E(): 2.7e-35,(35.3%
                     identity in 391 aa overlap); etc."
                     /codon_start=1
                     /transl_table=11
                     /product="Possible transposase"
                     /protein_id="NP_217945.1"
                     /db_xref="GI:15610564 GeneID:887621"
                     /translation="MATIAQRLRDDHGVAASESSVRRWIATHFAEEVARERVTVPRGPVDAGSEAQIDYGRLGMWFDPATARRVA
                     VWAFVMVLAFSRHLFVRPVIRMDQTAWCACHVAAFEFFDGVPARLVCDNLRTGVDKPDLYDPQINRSYAELASHYATLVDPARAR
                     KPKDKPRVERPMTYVRDSFWKGREFDSLAQMQQAAVTWSTEVAGLRYLRALEGAQPLRMFEAVEQQALIALPPRAFELTSWSIGT
                     VGVDTHLKVGKALYSVPWRLIGQRLHARTAGDVVQIFAGNDVVATHVRRPSGRSTDFSHYPPEKIAFHMRTPTWCRHTAELVGPA
                     SQQVIAEFMRDNAIHHLRSAQGVLGLRDKHGCDRLEAACARAIEVGDPSYRTIKGILVAGTEHAANEPTTSSPASTAGGVPARP"
     CDS             complement(3608171..3609274)
                     /locus_tag="Rv3430c"
                     /experiment="EXISTENCE: identified in proteomics study"
                     /note="Rv3430c, (MTCY77.02c), len: 387 aa. Possible IS1540 transposase, similar to
                     several e.g. Q49592 transposase from Mycobacterium intracellulare (340 aa), FASTA
                     scores: opt: 1377, E(): 1.6e-81, (64.2% identity in 338 aa overlap); similarity is
                     lost at C-terminus due to possible frameshift after aa 297."
                     /codon_start=1
                     /transl_table=11
                     /product="Possible transposase"
                     /protein_id="NP_217947.1"
                     /db_xref="GI:15610566 GeneID:887615"
                     /translation="MIDTAIEEMIPLIGVRAACAATGRAPASYYRAHSKRLSAQSDTFTSTAVTDPSGPRESAQPRALSAAEREH
                     VLAVLNSQRFADMAPAVVYATLLDEGIYLCSESTMYRLLRERGQTGDRRRQATHPAAVKPELVAHQPNSVWSWDITKLRGPAKWS
                     YYYLYVILDIFSRYVVGWMVASRESKVLAERLIAQTLAAQHISADQLTLHADRGSSMSSKPVALLLADLGVTKSHSRPHTSNDNP
                     LSEAQFKTLKYRPDFPKRFESIEAARVHCDRFFGWYNHEHKHSGIGLHTPADVHYGRADQIRRHRATVLDTAYRDHLERIRSQTT
                     RATRATGLQRDQPTTEGGPADSINPRKSCLRNVDRFRPGLLD"
     CDS             complement(3609763..3610608)
                     /locus_tag="Rv3431c"
                     /note="Rv3431c, (MTCY77.03c), len: 281 aa. Possible truncated transposase for IS1552,
                     similar to, but shorter than other transposases e.g. P72303 from Rhodococcus opacus
                     (418 aa), FASTA scores: opt: 1509, E(): 1.2e-91, (80.95% identity in 278 aa overlap);
                     Q9AKV5 from Mycobacterium paratuberculosis (395 aa), FASTA scores: opt: 1115, E():
                     7.8e-66, (63.45% identity in 268 aa overlap); etc."
                     /codon_start=1
                     /transl_table=11
                     /product="Possible transposase (fragment)"
                     /protein_id="NP_217948.1"
                     /db_xref="GI:15610567 GeneID:887608"
                     /translation="MFAELIRAGLQALIEAEATEAIGAGRYERSDGRIVHRNGHRPKTVSTTAGDIEVQIPKLRAGSFFPSLLER
                     RRRIDKALHAVIMEAYVHGVSTRSVDDLVAAMGVQAGVSKSEVSRICAGLDTEIEAFRTRSLTHTEFPYVFCDATFCKVRVGAHV
                     VSQALVVATGVSIDGTREVLGTAVGDSESYEFWREFLASLKARGLTGVHLVISDAHAGLKAAVAQQFSGASWQRCRVHFMRNLYT
                     AVAAKHAPAVTVAVKTIFAHTDPEEVGAQWDRVADPLCQP"
     CDS             3651299..3651625
                     /locus_tag="Rv3474"
                     /note="Rv3474, (MTCY13E12.27), len: 108 aa. Probable transposase subunit for IS6110.
                     Identical to many other M. tuberculosis IS6110 transposase subunits. The transposase
                     described here may be made by a frame shifting mechanism during translation that
                     fuses Rv3474 and Rv3475, the sequence UUUUAAAG (directly upstream of Rv3475) maybe
                     responsible for such a frameshifting event (see McAdam et al., 1990). Belongs to the
                     transposase family 8."
                     /codon_start=1
                     /transl_table=11
                     /product="Possible transposase for insertion element IS6110 (fragment)"
                     /protein_id="NP_217991.1"
                     /db_xref="GI:15610610 GeneID:888097"
                     /translation="MSGGSSRRYPPELRERAVRMVAEIRGQHDSEWAAISEVARLLGVGCAETVRKWVRQAQVDAGARPGTTTEE
```

```
                     SAELKRLRRDNAELRRANAILKTASAFFAAELDRPAR"
     CDS             3651574..3652560
                     /locus_tag="Rv3475"
                     /note="Rv3475, (MTCY13E12.28), len: 328 aa. Probable transposase subunit for IS6110.
                     Identical to many other M. tuberculosis IS6110 transposase subunits. The transposase
                     described here may be made by a frame shifting mechanism during translation that
                     fuses Rv3474 and Rv3475, the sequence UUUUAAAG (directly upstream of Rv3475) maybe
                     responsible for such a frameshifting event (see McAdam et al., 1990). Start changed
                     since first submission (- 18 aa)."
                     /codon_start=1
                     /transl_table=11
                     /product="Possible transposase for insertion element IS6110 [second part]"
                     /protein_id="NP_217992.2"
                     /db_xref="GI:448824814 GeneID:888055"
                     /translation="KDRVGFLRGRARPASTLITRFIADHQGHREGPDGLRWGVESICTQLTELGVPIAPSTYYDHINREPSRREL
                     RDGELKEHISRVHAANYGVYGARKVWLTLNREGIEVARCTVERLMTKLGLSGTTRGKARRTTIADPATARPADLVQRRFGPPAPN
                     RLWVADLTYVSTWAGFAYVAFVTDAYARRILGWRVASTMATSMVLDAIEQAIWTRQQEGVLDLKDVIHHTDRGSQYTSIRFSERL
                     AEAGIQPSVGAVGSSYDNALAETINGLYKTELIKPGKPWRSIEDVELATARWVDWFNHRRLYQYCGDVPPVELEAAYYAQRQRPA
                     AG"
     CDS             3804387..3804734
                     /locus_tag="Rv3636"
                     /note="Rv3636, (MTCY15C10.16c), len: 115 aa. Possible transposase, weakly similar to
                     others e.g. O69924|SC3C8.12 putative transposase from Streptomyces coelicolor (487
                     aa) FASTA scores: opt: 132, E(): 0.12, (33.05% identity in 112 aa overlap); O96916
                     TC1-like transposase from Anopheles gambiae (African malaria mosquito) (332 aa),
                     FASTA scores: opt: 117, E(): 0.84, (30.75% identity in 91 aa overlap);
                     Q9R2U5|IS466A|IS466A-ORF|TNPA|IS469|SCP1.276 transposase (insertion element IS466S
                     transposase) from Streptomyces coelicolor (513 aa), FASTA scores: opt: 114, E(): 2,
                     (30.5% identity in 82 aa overlap); etc. Similar in part to P96288|Rv2943|MTCY24G1.06c
                     hypothetical 45.8 KDA protein from Mycobacterium tuberculosis (413 aa), FASTA scores:
                     opt: 533, E(): 1.4e-28, (74.55% identity in 110 aa overlap). Contains possible
                     helix-turn-helix motif from aa 19-40 (+4.98 SD)."
                     /codon_start=1
                     /transl_table=11
                     /product="Possible transposase"
                     /protein_id="NP_218153.1"
                     /db_xref="GI:15610772 GeneID:885274"
                     /translation="MLSVEDWAEIRRLRRSERLPISEIARVLKISRNTVKSALASDGPPKYQRAAKGSVADEAEPRIRELLAAYP
                     RMPATVIAERIGWWYSIRTLSGRVRELRPLYLPPDPASRDICGR"
     CDS             3805119..3805619
                     /locus_tag="Rv3637"
                     /note="Rv3637, (MTCY15C10.15c), len: 166 aa. Possible transposase. C-terminal end
                     highly similar to Q9RLQ9|ISTA putative transposase a (fragment) from Mycobacterium
                     bovis (102 aa), FASTA scores: opt: 397, E(): 1.4e-19, (58.8% identity in 102 aa
                     overlap). Weakly similar to others e.g. Q9KJ02 putative transposase (fragment) from
                     Polyangium cellulosum (329 aa), FASTA scores: opt: 191, E(): 1.6e-05,(32.1% identity
                     in 134 aa overlap); Q9LCU2|ISTA cointegrase from Pseudomonas aeruginosa (382 aa)
                     FASTA scores: opt: 144, E(): 0.024, (26.8% identity in 123 aa overlap);
                     P15025|ISTA_PSEAE transposase for insertion sequence element IS21 from Pseudomonas
                     aeruginosa (390 aa), FASTA scores: opt: 144, E(): 0.025, (26.85% identity in 123 aa
                     overlap); etc. Also highly similar to C-terminal end of P96288|Rv2943|MTCY24G1.06c
                     hypothetical 45.8 KDA protein from Mycobacterium tuberculosis (413 aa) FASTA scores:
                     opt: 722, E(): 1.5e-40, (63.7% identity in 168 aa overlap)."
                     /codon_start=1
                     /transl_table=11
                     /product="Possible transposase"
                     /protein_id="NP_218154.1"
                     /db_xref="GI:15610773 GeneID:885496"
                     /translation="MPGRVFASPADFNTQLQAWLVRANHRQHRVLGCRPADRIEADTAAMLTLPPVGPSIGWRTSTRLPRDHYVR
                     LDGNDYSVHPVAIGRRIEITADLSRVRVWCGGTLVADHDRIWAKHQTISDPEHVVAAKLLRRKRFDIVGPPHHVEVEQRLLTTYD
                     TVLGLDGPVA"
     CDS             3805619..3806365
                     /locus_tag="Rv3638"
                     /note="Rv3638, (MTCY15C10.14c), len: 248 aa. Possible transposase, highly similar to
                     Q9RLQ8|ISTB ISTB protein from Mycobacterium bovis (266 aa), FASTA scores: opt:
                     784,E(): 4e-46, (78.0% identity in 259 aa overlap); and similar to others e.g.
                     P15026|ISTB_PSEAE insertion sequence IS21 putative ATP-binding protein from
                     Pseudomonas aeruginosa (265 aa), FASTA scores: opt: 420, E(): 2.2e-21, (38.8%
                     identity in 255 aa overlap); Q45619|ISTB_BACST insertion sequence IS5376 putative
```

ATP-binding protein from Bacillus stearothermophilus (251 aa), FASTA scores: opt: 402, E(): 3.6e-20, (34.5% identity in 232 aa overlap); P15026|ISTB_ECOLI ISTB protein from Escherichia coli (265 aa), FASTA scores: opt: 419, E(): 8e-23, (38.8% identity in 255 aa overlap); etc. C-terminus highly similar to C-terminus of P96287|Rv2944|MTCY24G1.05 hypothetical 25.5 KDA protein from Mycobacterium tuberculosis strain H37Rv (alias AAK47343|MT3016 IS1533, ORFB from Mycobacterium tuberculosis strain CDC1551) (238 aa), FASTA scores: opt: 784, E(): 3.6e-46, (87.4% identity in 135 aa overlap)."
/codon_start=1
/transl_table=11
/product="Possible transposase"
/protein_id="NP_218155.1"
/db_xref="GI:15610774 GeneID:885803"
/translation="MAAKTATNSRDVAAELAYLTRALKAPTLRGAIEQLADRARTKTWSYEEFLAACLQREVSARESHGGEGRIRAARFPSRKSLEEFDFDHARGLKRDTIAHLGTLDFVTLAIGIAIRACQAGHRVLFATASQWVDRLAAAHHSGTLQSELIRLARYPLLVVDEVGYIPFEPEAANLFFQLVSSRYERASLIVTSNKPFGRWGEVFGDDVVAAAMIDRLVHHAEVIALKGDSYRIKDRDLGRVPTVTADDQ"

CDS complement(3807155..3808384)
/locus_tag="Rv3640c"
/experiment="EXISTENCE: identified in proteomics study"
/note="Rv3640c, (MTCY15C10.12), len: 409 aa. Probable transposase, highly similar to others e.g. Q48882 transposase from Mycobacterium avium (411 aa) FASTA scores: opt: 1574, E(): 6.2e-93, (59.75% identity in 400 aa overlap); Q9AKV5 putative transposase (fragment) from Mycobacterium paratuberculosis (395 aa), FASTA scores: opt: 1566, E(): 1.9e-92, (60.0% identity in 395 aa overlap); Q48368 transposase from Mycobacterium avium (410 aa), FASTA scores: opt: 1561, E(): 4.1e-92, (59.4% identity in 404 aa overlap); etc."
/codon_start=1
/transl_table=11
/product="Probable transposase"
/protein_id="NP_218157.1"
/db_xref="GI:15610776 GeneID:885324"
/translation="MALPQSALSELLDAFRTGDGVDLIRDAVRLVLQELSELEATERIGAARYERSDTRVTDRNGARSRVLSTQAGDVELRIPKLRKGSFFPAILEPRRRIDQALYAVVMEAYVHGISTRAVDDLVEAMGVETGISKSEVSRICAGLDEIVGAFRTRTLGHIEFPYVYLDATYLNVRNGTGQVVSMAVIVASGIAADGSREILGLDVGDSEDETFWRGFLTSLKGRGLGGVRLVISDQHAGLVKALKRCFQGAGHQRCRVHFARNLLAHVPKDKADMVASMFRMIFSAPDAEAVHATWEGVRDRLAASFPKIGPLMDDARAEVLAFTAFPKAHWQKIWSTNPLERINKEIKRRSRVVGIFPNPAAVIRLVGAVLADMHDEWQASERRYLSEASMALLYPDSDNAVVAAISGGQ"

CDS complement(3941817..3941999)
/locus_tag="Rv3770A"
/note="Rv3770A, len: 60 aa. Probable remnant of a transposase, similar to many e.g. Rv2812|MTCY16B7.31c|Z81331_17 IS1604 putative transposase from Mycobacterium tuberculosis (469 aa), FASTA scores: opt: 204, E(): 1e-07, (80.5% identity in 41 aa overlap). Continuation of Rv3770B."
/codon_start=1
/transl_table=11
/product="Probable remnant of a transposase"
/protein_id="YP_178012.1"
/db_xref="GI:57117153 GeneID:3205079"
/translation="MGSTPWCPNPCQCTLRTPVEVLELAVALRPENPDRTAGAIQRILRAQLAGDRIALRGRGS"

CDS complement(3942014..3942205)
/locus_tag="Rv3770B"
/note="Rv3770B, len: 63 aa. Probable remnant of a transposase, similar to many e.g. Rv2812|MTCY16B7.31c|Z81331_17 IS1604 putative transposase from Mycobacterium tuberculosis (469 aa), FASTA scores: opt: 379, E(): 1.6e-21, (93.55% identity in 62 aa overlap). Continues as Rv3770A."
/codon_start=1
/transl_table=11
/product="Probable remnant of a transposase"
/protein_id="YP_178013.1"
/db_xref="GI:57117154 GeneID:3205080"
/translation="MRAERARAIGLFRYQLIREAADAAHSTKERGKMVRELASREHTDPFGRKVRISRHTIDRWIRN"

CDS 3978929..3980263
/locus_tag="Rv3798"
/note="Rv3798, (MTV026.03), len: 444 aa. Probable transposase for insertion sequence element IS1557, highly similar to Q60255 similar to transposase of ISAE1 from alcaligenes eutrophus H1-4 (fragment) from dibenzofuran-degrading bacterium DPO360 (163 aa) FASTA scores: opt: 767, E(): 3.2e-42, (67.25% identity in 168 aa overlap); and similar to P74920 transposase from Thiobacillus ferrooxidans (404 aa), FASTA scores: opt: 375,E(): 1.1e-16, (27.55% identity in 439 aa overlap); Q48349

transposase from Alcaligenes eutrophus (Ralstonia eutropha) (408 aa), FASTA scores: opt: 324, E(): 2e-13, (3.9% identity in 369 aa overlap); Q9FDC1|TNP transposase from Burkholderia mallei (Pseudomonas mallei) (386 aa) FASTA scores: opt: 282, E(): 9.8e-11, (25.85% identity in 391 aa overlap); etc. C-terminal end identical to O53804|Rv0741|MTV041.15 transposase from Mycobacterium tuberculosis (104 aa), FASTA scores: opt: 582, E(): 1.8e-30, (85.6% identity in 104 aa overlap). Belongs to the transposase family 12."
/codon_start=1
/transl_table=11
/product="Probable transposase"
/protein_id="NP_218315.1"
/db_xref="GI:15610934 GeneID:886268"
/translation="MRNVRLFRALLGVDKRTVIEDIEFEEDDAGDGARVIARVRPRSAVLRRCGRCGRKASWYDRGAGLRQWRSLDWGTVEVFLEAEAPRVNCPTHGPTVVAVPWARHHAGHTYAFDDTVAWLAVACSKTAVCELMRIAWRTVGAIVARVWADTEKRIDRFANLRRIGIDEISYKRHHRYLTVVVDHDSGRLVWAAPGHDKATLGLFFDALGAERAAQITHVSADAADWIADVVTERCPDAIQCADPFHVVAWATEALDVERRRAWNDARAIARTEPKWGRGRPGKNAAPRPGRERARRLKGARYALWKNPEDLTERQSAKLAWIAKTDPRLYRAYLLKESLRHVFSVKGEEGKQALDRWISWAQRCRIPVFVELAARIKRHRVAIDAALDHGLSQGLIESTNTKIRLLTRIAFGFRSPQALIALAMLTLAGHRPTLPGRHNHPQISQ"

CDS complement(4025984..4027210)
/locus_tag="Rv3827c"
/experiment="EXISTENCE: identified in proteomics study"
/note="Rv3827c, (MTCY409.03), len: 408 aa. Possible transposase within IS1537 element, similar to several transposases e.g. O83029|TNPC|DR2324|DR0666|DR0978|DR1381|D R1651|DR1933 transposase from Deinococcus radiodurans(408 aa) FASTA scores: opt: 302, E(): 3.9e-12, (30.75% identity in 358 aa overlap); Q9RXX7|DR0178 putative transposase from Deinococcus radiodurans (409 aa), FASTA scores: opt: 297,E(): 8.2e-12, (31.1% identity in 360 aa overlap); P73816|SLR2062 transposase from Synechocystis sp. strain PCC 6803 (400 aa), FASTA scores: opt: 296, E(): 9.3e-12,(30.05% identity in 353 aa overlap); etc. Highly similar to proteins from Mycobacterium tuberculosis e.g. O33333|Rv2791c|MTV002.56c transposase (459 aa) FASTA scores: opt: 2211, E(): 9.4e-136, (87.75% identity in 367 aa overlap); P95117|Rv2978c|MTCY349.09 hypothetical 51.4 KDA protein (459 aa), FASTA scores: opt: 2165, E(): 9e-133,(85.85% identity in 367 aa overlap); Q10809|YS85_MYCTU|Rv2885c|MT2953|MTCY274.16c hypothetical 51.3 KDA protein (460 aa), FASTA scores: opt: 2127, E(): 2.6e-130, (83.95% identity in 368 aa overlap); O0777|Rv0606|MTCY19H5.16c probable transposase (fragment) (247 aa), FASTA scores: opt: 1405, E(): 9.3e-84, (85.3% identity in 238 aa overlap); etc."
/codon_start=1
/transl_table=11
/product="Possible transposase"
/protein_id="NP_218344.1"
/db_xref="GI:15610963 GeneID:886151"
/translation="MMARFEVPEGWCVQAFRFTLDPTEDQARALARHFGARRKAYNWAVATLKADIEAWRVTGIGTVKPSLRVLRKRWNTVKDEVCVNAETGAVWWPECSKEAYADGIGGAVDAYWNWQNSRSGKREGKTMGFPRFKKKGRDQDRVTFTTGAMRVEPDRRHLTLPVVGTVRTHENTRRIERLIATGRARVLAISVRRNGTRLDASVRVLVQRPQQPNVAQPGSRVGVDVGVRRLATVANEAGAVLEEVPNPRPLDTALKELRYASRARSRCTKGSRRYRERTTEISRLHRRVNDVRTHHLHVLTTRLAQTHGHIVVEGLDAAGMLRQKGLPGARARRRGLSDSALGTPRRHLSYKTGWYGSALVVADRWFPSLSVEPTVRPGLARLVAVKRGREAAAWLPNNPETGCKSRDH"

CDS complement(4027207..4027818)
/locus_tag="Rv3828c"
/inference="protein motif:PROSITE:PS00397"
/note="Rv3828c, (MTCY409.02), len: 203 aa. Possible resolvase within IS1537 element, similar to others e.g. Q97X40|SSO1915 first ORF in transposon ISC1913 from Sulfolobus solfataricus (213 aa), FASTA scores: opt: 275,E(): 1.6e-11, (30.6% identity in 196 aa overlap); Q9V1M0|PAB2076 resolvase related protein from Pyrococcus abyssi (212 aa), FASTA scores: opt: 254, E(): 4.2e-10,(29.95% identity in 197 aa overlap); Q9RMU7|ORFA putative transposase (belongs to the MerR family of transcriptional regulators) from Helicobacter pylori (Campylobacter pylori) (217 aa), FASTA scores: opt: 243, E(): 2.3e-09, (31.8% identity in 154 aa overlap); etc. Also highly similar to proteins from Mycobacterium tuberculosis e.g. O33334|Rv2792c|MTV002.57c resolvase (193 aa), FASTA scores: opt: 970, E(): 1.5e-58, (79.25% identity in 193 aa overlap); O07773|Rv0605|MTCY19H5.17c putative resolvase (202 aa), FASTA scores: opt: 964, E(): 4e-58, (76.25% identity in 202 aa overlap); P95116|Rv2979c|MTCY349.08 hypothetical 21.4 KDA protein (194 aa), FASTA scores: opt: 895, E(): 1.8e-53, (74.75% identity in 194 aa overlap); Q10831|YS86_MYCTU|Rv2886c|MT2954|MTCY274.17c hypothetical 31.9 KDA protein (295 aa), FASTA scores: opt: 826, E(): 1.1e-48, (66.2% identity in 204 aa overlap) (similarity only at C-terminus); etc. Contains PS00397 Site-specific recombinases active site. Possible helix-turn-helix motif from aa 11-32, Score 1305 (+3.63 SD)."
/codon_start=1

```
                     /transl_table=11
                     /product="Possible resolvase"
                     /protein_id="NP_218345.1"
                     /db_xref="GI:15610964 GeneID:886160"
                     /translation="MSVVCCRNRWMNLAVWAERNGVAWVIAYRWFRAGLLPVPAQRVGRLILVNDPAVEESGRGRTLVYARVSSA
                     DQRSDLDRRVARVTAWATSQHLSVDKVVAEGGWALNGHRRKFFALLGDPVVTRIVVEHRDRFCWFGSEYVEAALVAQGRELVVVD
                     LAEVDDDLVGDMTEILTSMCARLYGERAAQNGAKRALAAAVGDAEAA"
     CDS             4043196..4043687
                     /locus_tag="Rv3844"
                     /note="Rv3844, (MTCY01A6.25), len: 163 aa. Possible transposase, identical to
                     P96234|Rv3348|MTV004.04 putative transposase from Mycobacterium tuberculosis. Also
                     some similarity with others e.g. N-terminal part of P19834|YI11_STRCL insertion
                     element IS116 hypothetical 44.8 KDA protein from Streptomyces clavuligerus (399 aa)
                     FASTA scores: opt: 146, E(): 0.017, (29.1% identity in 158 aa overlap)."
                     /codon_start=1
                     /transl_table=11
                     /product="Possible transposase"
                     /protein_id="NP_218361.1"
                     /db_xref="GI:15610980 GeneID:886180"
                     /translation="MTAENPGRSRRTLVGIDAAITACHHIAIRDDVGARSIRFSVEPTLAGLRTLTDKLSGYDDIDATVEPTSMT
                     WLPLTIAVENAGDTMHMAGARHCARLRGAIVGKSKSDVIDAEVLTRASEVFDLTPLTLPTPAQLALRRSVIRRAGAVIDANRSWR
                     RLMSLAR"
     misc_feature    1187573..1188124
                     /locus_tag="Rv1150"
                     /note="Rv1150, (MTCI65.17), len: 183 aa. Possible fragment of transposase
                     (pseudogene). Identical to C-terminal part of S21394 transposase of putative
                     Mycobacterium tuberculosis is element (308 aa), FASTA scores: opt: 959,E(): 0, (99.3%
                     identity in 145 aa overlap). The transposase described here may be made by a -1 frame
                     shifting mechanism during translation that fuses Rv1149|MTCI65.16 and
                     Rv1150|MTCI65.17. No evidence found to account for discrepancy with previously
                     published sequence. Second copy is Rv1041c|MTCY10G2.08."
                     /pseudogene="unknown"
     mobile_element  648644..649283
                     /note="IS1536, len: 1384 nt. Partial copy of insertion sequence IS_1536. This region
                     is a possible MT-complex-specific genomic island (See Becq et al.,2007).
                     /mobile_element_type="insertion sequence:IS1536"
     mobile_element  778868..779069
                     /note="IS1557'-1, len: 517 nt. Region similar to Insertion sequence IS1557 on
                     MTCY373- (IS1557- 1st copy). This region is a possible MT-complex-specific genomic
                     island (See Becq et al., 2007). /mobile_element_type="insertion sequence:IS1557'-1"
     mobile_element  826243..827597
                     /note="IS6110-1, len: 1355 nt. Insertion sequence IS6110.
                     /mobile_element_type="insertion sequence:IS6110-1"
     mobile_element  827610..828701
                     /note="IS1547-1, len: 1092 nt. Insertion sequence IS1547.
                     /mobile_element_type="insertion sequence:IS1547-1"
     mobile_element  858797..859084
                     /note="IS1605', len: 288 nt. Insertion sequence IS1605'.
                     /mobile_element_type="insertion sequence:IS1605'"
     mobile_element  879224..879554
                     /note="IS1606', len: 331 nt. Insertion sequence IS1606'
                     /mobile_element_type="insertion sequence:IS1606'"
     mobile_element  complement(952646..954081)
                     /note="IS1554, len: 1436 nt. Putative Insertion sequence element bounded by 15 bp
                     inverted repeats. /mobile_element_type="insertion sequence:IS1554"
     mobile_element  954249..956548
                     /note="IS1535, len: 2300 nt. Putative Insertion sequence element bounded by 16 bp
                     inverted repeats. /mobile_element_type="insertion sequence:IS1535"
     mobile_element  complement(1080967..1082479)
                     /note="IS1560-1, len: 1513 nt. Insertion sequence IS1560.
                     /mobile_element_type="insertion sequence:IS1560-1"
     mobile_element  complement(1084614..1085591)
                     /note="IS-LIKE-1, len: 978 nt. Insertion sequence, ISLIKE,region identical to cosmid
                     y348, blast score= 4902 (+1) 9377 10354 EM_NEW:MTAD20 Ad000020 Mycobacterium
                     tuberculosis sequence from clone y348. This region is a possible MT-complex-specific
                     genomic island (See Becq et al., 2007). /mobile_element_type="insertion
                     sequence:IS-LIKE-1"
     mobile_element  1089340..1090774
                     /note="IS1081-1, len: 1435 nt. Insertion sequence IS1081,almost identical to
```

```
                 Mycobacterium bovis IS1081 (7157 (-1) 60 14 94 EM_BA:MBBIS1081 X84741 Mycobacterium
                 bovis BCG IS1081 DNA. 4/96. This region is a possible MT-complex-specific genomic
                 island (See Becq et al.,2007). /mobile_element_type="insertion sequence:IS1081-1"
mobile_element   1187147..1188130
                 /note="IS-LIKE-2, len: 984 nt. Insertion sequence element IS-LIKE.
                 /mobile_element_type="insertion sequence:IS-LIKE-2"
mobile_element   complement(1247221..1248649)
                 /note="IS1081-2, len: 1429 nt. Insertion sequence IS1081.
                 /mobile_element_type="insertion sequence:IS1081-2"
mobile_element   complement(1371981..1373489)
                 /note="IS1557-2, len: 1509 nt. Insertion sequence IS1557.
                 /mobile_element_type="insertion sequence:IS1557-2"
mobile_element   complement(1442787..1444141)
                 /note="IS6110-2, len: 1355 nt. Almost identical to Insertion sequence IS986 element.
                 /mobile_element_type="insertion sequence:IS6110-2"
mobile_element   complement(1862979..1864333)
                 /note="IS6110-3, len: 1355 nt. Insertion sequence IS6110.
                 /mobile_element_type="insertion sequence:IS6110-3"
mobile_element   1871377..1872731
                 /note="IS6110-4, len: 1355 nt. Insertion sequence IS6110.
                 /mobile_element_type="insertion sequence:IS6110-4"
mobile_element   complement(1873860..1875089)
                 /note="ISB9', len: 1230 nt. Insertion sequence ISB9, nearly identical to
                 EM_BA:MTU78639. Note that this sequence shows several differences to EM_BA: MTU78639,
                 and the transposase ORFs are extensively frameshifted. Our sequence has been checked
                 and is thought to be correct; the sequence in EM_BA:MTU78639 is from a different
                 isolate of Mycobacterium tuberculosis. /mobile_element_type="insertion sequence:ISB9'"
mobile_element   2107174..2108401
                 /note="IS1607, len: 1228 nt. Vestigial Insertion sequence element, IS1607.
                 /mobile_element_type="insertion sequence:IS1607"
mobile_element   2189673..2191141
                 /note="IS1556, len: 1469 nt. Possible Insertion sequence-like region. This region is
                 a possible MT-complex-specific genomic island (See Becq et al.,2007).
                 /mobile_element_type="insertion sequence:IS1556"
mobile_element   2210667..2212021
                 /note="IS6110-5, len: 1355 nt. Insertion sequence IS6110.
                 /mobile_element_type="insertion sequence:IS6110-5"
mobile_element   complement(2270549..2271903)
                 /note="IS6110-6, len: 1355 nt. Insertion sequence IS6110.
                 /mobile_element_type="insertion sequence:IS6110-6"
mobile_element   complement(2279577..2280380)
                 /note="IS1558-1, len: 804 nt. Insertion sequence IS1558,nearly identical to
                 complement of region 24105 24908 in EM_BA:MTCY428 Z81451 Mycobacterium tuberculosis
                 cosmid Y428. /mobile_element_type="insertion sequence:IS1558-1"
mobile_element   2390446..2391800
                 /note="IS6110-7, len: 1355 nt. Insertion sequence IS6110.
                 /mobile_element_type="insertion sequence:IS6110-7"
mobile_element   2471393..2472731
                 /note="IS6110-8, len: 1355 nt. Insertion sequence IS6110 element that appears to have
                 inserted in 5'-end of MTCY98.031c but is not flanked by expected 3 bp direct repeats
                 of target sequence. /mobile_element_type="insertion sequence:IS6110-8"
mobile_element   complement(2552739..2553737)
                 /note="IS1558-2, len: 999 nt. Insertion sequence IS1558.
                 /mobile_element_type="insertion sequence:IS1558-2"
mobile_element   complement(2615689..2617045)
                 /note="IS6110-9, len: 1357 nt. Insertion sequence IS6110.
                 /mobile_element_type="insertion sequence:IS6110-9"
mobile_element   complement(2652496..2653945)
                 /note="IS1081-3, len: 1450 nt. Insertion sequence IS1081.
                 /mobile_element_type="insertion sequence:IS1081-3"
mobile_element   2787539..2788893
                 /note="IS6110-10, len: 1355 nt. Insertion sequence IS6110.
                 /mobile_element_type="insertion sequence:IS6110-10"
mobile_element   2798376..2799284
                 /note="IS1081'-4, len: 909 nt. Defective Insertion sequence IS1081 element; truncated
                 at 3'-end. /mobile_element_type="insertion sequence:IS1081'-4"
mobile_element   2910865..2912896
                 /note="IS1602, len: 2032 nt. Insertion sequence IS1602.
                 /mobile_element_type="insertion sequence:IS1602"
```

```
     mobile_element  complement(2926434..2926832)
                     /note="IS1555', len: 399 nt. Probable defective Insertion sequence element, IS1555.
                     This region is a possible MT-complex-specific genomic island (See Becq et al.,2007).
                     /mobile_element_type="insertion sequence:IS1555'"
     mobile_element  2927507..2928915
                     /note="IS1604, len: 1409 nt. Insertion sequence IS1604. This region is a possible
                     MT-complex-specific genomic island (See Becq et al., 2007).
                     /mobile_element_type="insertion sequence:IS1604"
     mobile_element  complement(2931213..2932587)
                     /note="IS6110-11, len: 1375 nt. Insertion sequence IS6110. This region is a possible
                     MT-complex-specific genomic island (See Becq et al., 2007).
                     /mobile_element_type="insertion sequence:IS6110-11"
     mobile_element  complement(3003008..3005274)
                     /note="IS1539, len: 2267 nt. Insertion sequence IS1539.
                     /mobile_element_type="insertion sequence:IS1539"
     mobile_element  3096078..3098119
                     /note="IS1533, len: 2042 nt. Minimum region corresponding to Insertion sequence
                     IS1533. /mobile_element_type="insertion sequence:IS1533"
     mobile_element  complement(3141383..3143407)
                     /note="IS1538, len: 2025 nt. Similar to other Insertion sequence elements in M.
                     tuberculosis e.g. IS1535, IS1536,IS1537, & IS1539 (EM_NEW:MTCY274 Z74024
                     Mycobacterium tuberculosis cosmid Y274) /mobile_element_type="insertion
                     sequence:IS1538"
     mobile_element  3187259..3188582
                     /note="IS1081-5, len: 1324 nt. Insertion sequence IS1081.
                     /mobile_element_type="insertion sequence:IS1081-5"
     mobile_element  3287307..3288630
                     /note="IS1081-6, len: 1324 nt. Insertion sequence IS1081. This region is a possible
                     MT-complex-specific genomic island (See Becq et al., 2007).
                     /mobile_element_type="insertion sequence:IS1081-6"
     mobile_element  3351417..3352771
                     /note="IS6110-12, len: 1355 nt. Insertion sequence IS6110. This region is a possible
                     MT-complex-specific genomic island (See Becq et al., 2007).
                     /mobile_element_type="insertion sequence:IS6110-12"
     mobile_element  3352900..3354254
                     /note="IS6110-13, len: 1355 nt. Insertion sequence IS6110. This region is a possible
                     MT-complex-specific genomic island (See Becq et al., 2007).
                     /mobile_element_type="insertion sequence:IS6110-13"
     mobile_element  complement(3357501..3358532)
                     /note="IS1603, len: 1032 nt. Insertion sequence IS1603. This region is a possible
                     MT-complex-specific genomic island (See Becq et al., 2007).
                     /mobile_element_type="insertion sequence:IS1603"
     mobile_element  3510569..3511923
                     /note="IS6110-14, len: 1355 nt. Insertion sequence IS6110.
                     /mobile_element_type="insertion sequence:IS6110-14"
     mobile_element  3511924..3513009
                     /note="IS1547-2, len: 1086 nt. Region corresponding to Insertion sequence IS1547,
                     positions 1982 3067 in EM_NEW:MTY13470. /mobile_element_type="insertion
                     sequence:IS1547-2"
     mobile_element  3531115..3531603
                     /note="IS1608', len: 489 nt. Insertion sequence IS1608'.
                     /mobile_element_type="insertion sequence:IS1608'"
     mobile_element  complement(3531646..3532383)
                     /note="IS1561', len: 738 nt. Insertion sequence IS1561'.
                     /mobile_element_type="insertion sequence:IS1561'"
     mobile_element  3559490..3560844
                     /note="IS6110-15, len: 1355 nt. Insertion sequence IS6110.
                     /mobile_element_type="insertion sequence:IS6110-15"
     mobile_element  3564419..3565986
                     /note="IS1560-2, len: 1568 nt. Possible Insertion sequence element IS_1560. Second
                     copy in MTCY10G2 from 11273 to 12919. /mobile_element_type="insertion
                     sequence:IS1560-2"
     mobile_element  complement(3604894..3606976)
                     /note="IS1532, len: 2083 nt. Insertion sequence IS1532.
                     /mobile_element_type="insertion sequence:IS1532"
     mobile_element  complement(3608171..3609275)
                     /note="IS1540, len: 1163 nt. Insertion sequence IS1540.
                     /mobile_element_type="insertion sequence:IS1540"
     mobile_element  complement(3609765..3610609)
```

```
                /note="IS1552, len: 845 nt. Insertion sequence IS1552.
                /mobile_element_type="insertion sequence:IS1552"
mobile_element  3651248..3652602
                /note="IS6110-16, len: 1355 nt. Insertion sequence IS6110.
                /mobile_element_type="insertion sequence:IS6110-16"
mobile_element  3804250..3806385
                /note="IS1534, len: 2136 nt. Putative Insertion sequence element, IS1534 (IS15C10.2),
                that resembles IS21; possibly defective. /mobile_element_type="insertion
                sequence:IS1534"
mobile_element  complement(3807141..3808433)
                /note="IS1553, len: 1293 nt. Putative Insertion sequence element, IS1553.
                /mobile_element_type="insertion sequence:IS1553"
mobile_element  3978929..3980260
                /note="IS1557-3, len: 1332 nt. Insertion sequence IS1557.
                /mobile_element_type="insertion sequence:IS1557-3"
mobile_element  complement(4025964..4027836)
                /note="IS1537, len: 1873 nt. Insertion sequence IS1537.
                /mobile_element_type="insertion sequence:IS1537"
```

```
     mobile_element  complement(1073125..1073224)
                     /note="IS1560-1, len: 1513 nt. Insertion sequence IS1560.
                     /mobile_element_type="insertion sequence:IS1560-1"
     mobile_element  complement(1234431..1234614)
                     /note="IS1081-2, len: 1429 nt. Insertion sequence IS1081.
                     /mobile_element_type="insertion sequence:IS1081-2"
     mobile_element  complement(3544100..3544196)
                     /note="IS1532, len: 2083 nt. Insertion sequence IS1532.
                     /mobile_element_type="insertion sequence:IS1532"
     mobile_element  complement(3545391..3545391)
                     /note="IS1540, len: 1163 nt. Insertion sequence IS1540.
                     /mobile_element_type="insertion sequence:IS1540"
     mobile_element  complement(3545879..3545879)
                     /note="IS1552, len: 845 nt. Insertion sequence IS1552.
                     /mobile_element_type="insertion sequence:IS1552"
```

```
     CDS             complement(1646761..1646865)
                     /locus_tag="Rv1572c"
                     /note="Rv1572c, (MTCY336.31B), len: 34 aa. Partial ORF,part of REP13E12 repeat
                     element; 3' end of Rv1587c (MTCY336.17) after phage-like element (see citation
                     below). Similar to C-terminal ends of other REP13E12 repeat elements e.g. Rv1148,
                     Rv1945, Rv3467, etc. Length extended since first submission (+7 aa). This region is a
                     possible MT-complex-specific genomic island (See Becq et al.,2007)."
                     /codon_start=1
                     /transl_table=11
                     /product="hypothetical protein"
                     /protein_id="NP_216088.2"
                     /db_xref="GI:57116888 GeneID:886333"
                     /translation="MECSSAVHGQPRTNTFHHHEKLLRHNDEDNHDDP"
     CDS             1646881..1647291
                     /locus_tag="Rv1573"
                     /note="Rv1573, (MTCY336.31c), len: 136 aa. Probable phiRv1 phage protein (see
                     citation below). This region is a possible MT-complex-specific genomic island (See
                     Becq et al., 2007)."
                     /codon_start=1
                     /transl_table=11
                     /product="Probable PhiRv1 phage protein"
                     /protein_id="NP_216089.1"
                     /db_xref="GI:15608711 GeneID:886337"
                     /translation="MTTTPARFNHLVTVTDLETGDRAVCDRDQVAETIRAWFPDAPLEVREALVRLQAALNRHEHTGELEAFLRI
                     SVEHADAAGGDECGPAILAGRSGPEQAAINRQLGLAGDDEPDGDDTPPWSRMIGLGGGSPAEDER"
     CDS             1647497..1647808
                     /locus_tag="Rv1574"
                     /note="Rv1574, (MTCY336.30), len: 103 aa. Probable phiRV1 phage related protein (see
                     citation below); some similarity to N-terminus of Rv1575|MTCY441.17 Probable phiRV1
                     phage protein (166 aa), E(): 1.5e-06; and Rv2647|MTCY336.29c Probable phiRV2 phage
                     protein, E(): 3.5e-05. Helix turn helix motif present at aa 14-35 (+3.61 SD). This
                     region is a possible MT-complex-specific genomic island (See Becq et al., 2007)."
                     /codon_start=1
                     /transl_table=11
                     /product="Probable PhiRv1 phage related protein"
                     /protein_id="NP_216090.1"
                     /db_xref="GI:15608712 GeneID:886331"
                     /translation="MGYKPESERHSTKTDTAIGAALGISAGTYRRLKRIDNATHSDDKEIRRFAEKQMAPLVAGSPSWNARKPRS
                     ANARVVASVHRSPMPALVPWNQSRLSATLTRR"
     CDS             1647766..1648266
                     /locus_tag="Rv1575"
                     /experiment="EXISTENCE: identified in proteomics study"
                     /note="Rv1575, (MTCY336.29c), len: 166 aa. Probable phiRV1 phage protein (see
                     citation below), showing similarity in N-terminal part to Rv1574|MTCY336.30c Probable
                     phiRV1 phage protein (103 aa), FASTA score: opt: 375, E(): 3.8e-16,(60.2% identity in
                     103 aa overlap); and Rv2647 Probable phiRV2 phage protein. Start changed since first
                     submission (+49 aa). This region is a possible MT-complex-specific genomic island
                     (See Becq et al., 2007)."
                     /codon_start=1
                     /transl_table=11
                     /product="Probable PhiRv1 phage protein"
                     /protein_id="NP_216091.2"
                     /db_xref="GI:57116889 GeneID:886335"
                     /translation="MEPKPSQRHTDKEVGAALGISAGTYKRLKRIDNATRSDDKEIRLFAEKQMAPLAAGSPSWNGRKPSSGNRK
                     AATMAARLDILAWGPWAPSQNRSVVRRKQTLLSAQPSASPPAPTGGSNESTTQPAASWRVGGPAPLSRGRPRLALSYLRGSLHLQ
                     NSKRVAHQHI"
     CDS             complement(1648210..1649631)
                     /locus_tag="Rv1576c"
                     /experiment="EXISTENCE: identified in proteomics study"
                     /note="Rv1576c, (MTCY336.28), len: 473 aa. Probable phiRV1 phage protein (capsid
                     subunit) (see citation below). Highly similar to hypothetical Mycobacterium
                     tuberculosis protein Rv2650c|MTCY441.19 phiRV2 phage related protein, FASTA scores:
                     opt: 2782, E(): 0, (89.1% identity in 468 aa overlap). This region is a possible
                     MT-complex-specific genomic island (See Becq et al., 2007)."
                     /codon_start=1
                     /transl_table=11
                     /product="Probable PhiRv1 phage protein"
                     /protein_id="NP_216092.1"
                     /db_xref="GI:15608714 GeneID:886327"
```

```
                     /translation="MTEFDDIKNLSLPETRDAAKQLLDSVAGDLTGEAAQRFQALTRHAEELRAEQRRRGREAEEALRRYRAGEL
                     RVVPGAPTGGDDGDAPPGNSLRDTAFRTLDSCVRDGLMSSRAAETAETLCRTGPPQSTSWAQRWLAATGSRDYLGAFVKRVSNPV
                     AGHTVWTDREAAAWREAAAVAAEQRAMGLVDTQGGFLIPAALDPAILLSGDGSTNPIRQVARVVQTTSEIWRGVTSEGAEARWYS
                     EAQEVSDDSPALAQPAVPNYRGSCWIPFSIELEGDAASFVGEIGKILADSVEQLQAAAFVNGSGNGEPTGFVSALTGTSDQVVVG
                     AGSEAIVAADVYALQSALPPRFQASAAFAANLSTINTLRQAETSNGALKFPSLHDSPPMLAGKSVLEVSHMDTVDSAVTATNHPL
                     VLGDWKQFLIGDRVGSMVELVPHLFGPNRRPTGQRGFFAWFRVGSDVLVRNAFRVLKVETTA"
     CDS             complement(1649639..1650151)
                     /locus_tag="Rv1577c"
                     /note="Rv1577c, (MTCY336.27), len: 170 aa. Probable phiRv1 phage protein (prohead
                     protease) (see citation below). Highly similar to hypothetical protein
                     Rv2651c|MTCY441.20c phiRV2 prohead protease, FASTA scores: E(): 0, (89.3% identity in
                     169 aa overlap). Some similarity to VP4_BPHK7|P49860 putative bacteriophage HK97
                     prohead protease (gp4) (225 aa), FASTA results: opt: 176, E(): 1.3e-05, (27.3%
                     identity in 165 aa overlap). This region is a possible MT-complex-specific genomic
                     island (See Becq et al., 2007)."
                     /codon_start=1
                     /transl_table=11
                     /product="Probable PhiRv1 phage protein"
                     /protein_id="NP_216093.1"
                     /db_xref="GI:15608715 GeneID:886329"
                     /translation="MAELRSGEGRTVHGTIVPYNEATTVRDFDGEFQEMFAPGAFRRSIAERGHKLKLLVSHDARTRYPVGRAVE
                     LREEPHGLFGAFEIADTPDGDEALANVKAGVVDSFSVGFRPIRDRREGDVLVRVEAALLEVSLTGVPAYSGAQIAGVRAESLTVV
                     SRSTAEAWLSLLDW"
     CDS             complement(1650325..1650795)
                     /locus_tag="Rv1578c"
                     /inference="protein motif:PROSITE:PS00221"
                     /note="Rv1578c, (MTCY336.26), len: 156 aa. Probable phiRv1 phage protein (terminase)
                     (see citation below), highly similar to Rv2652c|MTCY441.21c phiRV2 phage protein from
                     Mycobacterium tuberculosis, FASTA scores: E(): 4.8e-22,(48.1% identity in 156 aa
                     overlap). Also similar to X65555|ARP3COS_1 hypothetical protein (cos site)
                     -actinophage RP3 (210 aa), FASTA scores: opt: 373, E(): 6.5e-17, (50.0% identity in
                     114 aa overlap). Contains MIP family signature (PS00221). This region is a possible
                     MT-complex-specific genomic island (See Becq et al.,2007)."
                     /codon_start=1
                     /transl_table=11
                     /product="Probable PhiRv1 phage protein"
                     /protein_id="NP_216094.1"
                     /db_xref="GI:15608716 GeneID:886322"
                     /translation="MPRPPKPARLKLVEGRSPGRDSGGRKVPESPKFIRQAPDAPDWLDAEALAEWRRVAPTLERLDLLKPEDRA
                     LLSAYCETWSVYVAAVQRVRAEGLTITSPKSGVVHRNPAVTVAETARMHLLRLASEFGLTPAAEQRLAVAPGDDGDGLNPFAPDR"
     CDS             complement(1650876..1651190)
                     /locus_tag="Rv1579c"
                     /note="Rv1579c, (MTCY336.25), len: 104 aa. Probable phiRv1 phage protein (see
                     citation below). This region is a possible MT-complex-specific genomic island (See
                     Becq et al., 2007)."
                     /codon_start=1
                     /transl_table=11
                     /product="Probable PhiRv1 phage protein"
                     /protein_id="NP_216095.1"
                     /db_xref="GI:15608717 GeneID:886369"
                     /translation="MTPINRPLTNDERQLMHELAVQVVCSQTGCSPDAAVEALESFAKDGTLILRGDTENAYLEAGGNVLVHADR
                     DWLAFHASYPGNDPLRDARPIEQDDDQGAGSPS"
     CDS             complement(1651187..1651459)
                     /locus_tag="Rv1580c"
                     /note="Rv1580c, (MTCY336.24), len: 90 aa. Probable phiRv1 phage protein (see citation
                     below). This region is a possible MT-complex-specific genomic island (See Becq et
                     al., 2007)."
                     /codon_start=1
                     /transl_table=11
                     /product="Probable PhiRv1 phage protein"
                     /protein_id="NP_216096.1"
                     /db_xref="GI:15608718 GeneID:886313"
                     /translation="MAETPDHAELRRRIADMAFNADVGMATCKRCGDAVPYIILPNLQTGEPVMGVADNKWKRANCPVDVGKPCP
                     FLIAEGVADSTDDTIEVDQ"
     CDS             complement(1651473..1651868)
                     /locus_tag="Rv1581c"
                     /note="Rv1581c, (MTCY336.23), len: 131 aa. Probable phiRv1 phage protein (see
                     citation below). This region is a possible MT-complex-specific genomic island (See
                     Becq et al., 2007)."
```

```
                     /codon_start=1
                     /transl_table=11
                     /product="Probable PhiRv1 phage protein"
                     /protein_id="NP_216097.1"
                     /db_xref="GI:15608719 GeneID:886318"
                     /translation="MTAVAITPASGGRHSVRFAYDSAIVSLIKSTIPAYARSWSAHTRCWFIDADWTPLLAAELRYHGHTVTGPA
                     DPAQQQCTDWAKALFRAVGPQRTPAVYRALSKVLHPDAPTGCPILQQQLNAARTALTNPA"
     CDS             complement(1652064..1653479)
                     /locus_tag="Rv1582c"
                     /experiment="EXISTENCE: identified in proteomics study"
                     /note="Rv1582c, (MTCY336.22), len: 471 aa. Probable phiRv1 phage protein (see
                     citation below). N-terminus is similar to C-terminus of Q38030 ORF9 Bacteriophage
                     phi-C31 (519 aa), FASTA scores: opt: 331, E(): 6.5e-15, (28.5% identity in 235 aa
                     overlap); and C-terminus to whole of Q38031 ORF10 of Bacteriophage phi-C31 (202 aa),
                     FASTA scores: opt: 353,E(): 1e-16, (31.1% identity in 190 aa overlap). Also similar
                     to part of AB016282|AB016282_42 Bacteriophage phi-105 (806 aa), FASTA scores: opt:
                     790, E(): 0, (32.7% identity in 459 aa overlap). Similarity to other phage proteins
                     described as putative DNA-polymerase or DNA-primase. Also slightly similar to
                     MTCY441.24c, FASTA scores: E(): 0.0055, (36.0% identity in 75 aa overlap). This
                     region is a possible MT-complex-specific genomic island (See Becq et al., 2007)."
                     /codon_start=1
                     /transl_table=11
                     /product="Probable PhiRv1 phage protein"
                     /protein_id="NP_216098.1"
                     /db_xref="GI:15608720 GeneID:886311"
                     /translation="MADIPYGTDYPDAPWIDRDGHVLIDDGGKPTQVHRGQARIAYRLAERYQDKLLHVAGIGWHSWDGRRWAAD
                     DRGEAKRAVLAELRQALSDSLNDKELRADVRKCESASGVAGVLDLAAALVPFAATVADLDSDPHLLNVANGTLDLHTLKLRPHAP
                     ADRITKICRGAYQSDTESPLWQAFLTRVLPDEGVRGFVQRLAGVGLLGTVREHVLAILIGVGANGKSVFDKAIRYALGDYACTAE
                     PDLFMHRENAHPTGEMDLRGVRWVAVSESEKDRRLAESTIKRLTGGDTIRARKMRQDFVEFTPSHTPLLITNHLPRVPGDDTAIW
                     RRIRVVPFEVVIPADEQDRELDARLQLEADSILSWAVAGWSDYQRIGLSQPDAVLAATSNYREDSDTIKRFIDDECVTSSPVLKA
                     TTTHLFEAWQRWRVQEGVPEISRKAFGQSLDTHGYPVTDKARDGRWRAGIAVRGADDFDD"
     CDS             complement(1653479..1653877)
                     /locus_tag="Rv1583c"
                     /note="Rv1583c, (MTCY336.21), len: 132 aa. Probable phiRv1 phage protein (see
                     citation below), highly similar to Rv2656c|MTCY441.25c phiRV2 phage protein (130 aa),
                     FASTA score: E(): 1.3e-33, (81.7% identity in 131 aa overlap). This region is a
                     possible MT-complex-specific genomic island (See Becq et al., 2007)."
                     /codon_start=1
                     /transl_table=11
                     /product="Probable PhiRv1 phage protein"
                     /protein_id="NP_216099.1"
                     /db_xref="GI:15608721 GeneID:886315"
                     /translation="MTAGAGGSPPTRRCPATEDRAPATVATPSSADPTASRAVSWWSVHEHVAPVLDAAGSWPMAGTPAWRQLDD
                     ADPRKWAAICDAARHWALRVETCQEAMAQASRDVSAAADWPGIAREIVRRRGVYIPRAGVA"
     CDS             complement(1653874..1654095)
                     /locus_tag="Rv1584c"
                     /note="Rv1584c, (MTCY336.20), len: 73 aa. Possible phiRv1 phage protein (putative
                     excisionase) (see citation below). This region is a possible MT-complex-specific
                     genomic island (See Becq et al., 2007)."
                     /codon_start=1
                     /transl_table=11
                     /product="Possible PhiRv1 phage protein"
                     /protein_id="NP_216100.1"
                     /db_xref="GI:15608722 GeneID:886307"
                     /translation="MSTIYHHRGRVAALSRSRASDDPEFIAAKTDLVAANIADYLIRTLAAAPPLTDEQRTRLAELLRPVRRSGG
                     AR"
     CDS             complement(1654151..1654666)
                     /locus_tag="Rv1585c"
                     /note="Rv1585c, (MTCY336.19), len: 171 aa. Possible phage phiRv1 protein (see Hatfull
                     2000). This region is a possible MT-complex-specific genomic island (See Becq et al.,
                     2007)."
                     /codon_start=1
                     /transl_table=11
                     /product="Possible phage PhiRv1 protein"
                     /protein_id="NP_216101.1"
                     /db_xref="GI:15608723 GeneID:886309"
                     /translation="MSRHHNIVIVCDHGRKGDGRIEHERCDLVAPIIWVDETQGWLPQAPAVATLLDDDNQPRAVIGLPPNESRL
                     RPEMRRDGWVRLHWEFACLRYGAAGVRTCEQRPVRVRNGDLQTLCENVPRLLTGLAGNPDYAPGFAVQSDAVVVAMWLWRTLCES
                     DTPNKLRATPTRGSC"
```

```
     CDS             complement(1654663..1656072)
                     /locus_tag="Rv1586c"
                     /note="Rv1586c, (MTCY336.18), len: 469 aa. Probable phiRv1 integrase, possibly member
                     of the serine family of recombinases (see citation below), similar to several
                     bacteriophage integrases e.g. Q37839 ORF469 protein from Bacteriophage R4 (469 aa),
                     FASTA scores: opt: 623, E(): 1.6e-29, (31.1% identity in 482 aa overlap); and
                     Bacteriophage TP901-1. This region is a possible MT-complex-specific genomic island
                     (See Becq et al.,2007)."
                     /codon_start=1
                     /transl_table=11
                     /product="Probable PhiRv1 integrase"
                     /protein_id="NP_216102.1"
                     /db_xref="GI:15608724 GeneID:886305"
                     /translation="MRYTTPVRAAVYLRISEDRSGEQLGVARQREDCLKLCGQRKWVPVEYLDNDVSASTGKRRPAYEQMLADIT
                     AGKIAAVVAWDLDRLHRRPIELEAFMSLADEKRLALATVAGDVDLATPQGRLVARLKGSVAAHETEHKKARQRRAARQKAERGHP
                     NWSKAFGYLPGPNGPEPDPRTAPLVKQAYADILAGASLGDVCRQWNDAGAFTITGRPWTTTTLSKFLRKPRNAGLRAYKGARYGP
                     VDRDAIVGKAQWSPLVDEATFWAAQAVLDAPGRAPGRKSVRRHLLTGLAGCGKCGNHLAGSYRTDGQVVYVCKACHGVAILADNI
                     EPILYHIVAERLAMPDAVDLLRREIHDAAEAETIRLELETLYGELDRLAVERAEGLLTARQVKISTDIVNAKITKLQARQQDQER
                     LRVFDGIPLGTPQVAGMIAELSPDRFRAVLDVLAEVVVQPVGKSGRIFNPERVQVNWR"
     CDS             complement(1655729..1656730)
                     /locus_tag="Rv1587c"
                     /note="Rv1587c, (MTCY336.17), len: 333 aa. Partial REP13E12 repeat protein (see
                     citation below), nearly identical (but has been interrupted by phiRv1 prophage) to
                     Q50655|MTCY251.13c|Rv0094c hypothetical 34.6 kDa protein from M. tuberculosis (317
                     aa), FASTA results: opt: 1511,E(): 1.1e-84, (97.75% identity in 224 aa overlap).
                     Codon usage suggests that translation may involve frameshifting of Rv1588c mRNA in
                     poly_C stretch into reading frame of Rv1587c. 3' end found in Rv1572c. Length
                     extended since first submission (+115 aa). This region is a possible
                     MT-complex-specific genomic island (See Becq et al.,2007)."
                     /codon_start=1
                     /transl_table=11
                     /product="Partial REP13E12 repeat protein"
                     /protein_id="NP_216103.2"
                     /db_xref="GI:57116890 GeneID:886303"
                     /translation="MLAKLAAPGATNPDDHTPVIDTTPDAAAIDRDTRSQAQRNHDGLLAGLRALIASGKLGQHNGLPVSIVVTT
                     TLTDLQTGAGKGFTGGGTLLPMADVIRMTSHAHHYSPASGRYPQAIFDHGTPLALYHTKRLASPAQRIMLFANDRGCTKPGCDAP
                     AYHSQAHHVTAWTSTGRTDITELTLACGPDNRLAEKGWTTHNNTHGHTEWLPPPHLDHGQPWTCEIHYTCACCCLPPNLRRPLRR
                     TARRGPPTRGLPKAVRAAKMGARRVPRQRRQRINRQAPPRLRADVGRHHRRQDRRRGGLGPGPAPSPSHRAGSLHVISRREAAGP
                     GHRRRRR"
     CDS             complement(2393568..2394023)
                     /locus_tag="Rv2309c"
                     /note="Rv2309c, (MTCY3G12.25), len: 151 aa. Possible integrase (fragment), similar to
                     others e.g. Q48908 integrase (fragment) from Mycobacterium paratuberculos (191 aa),
                     FASTA scores: opt: 279, E(): 3.2e-11, (40.4% identity in 136 aa overlap); etc. Also
                     similar to others from Mycobacterium tuberculosis e.g. Rv1055|MTV017.08 integrase
                     (fragment) (78 aa) (72.85% identity in 70 aa overlap); and Rv1054|MTV017.07 integrase
                     (fragment). Could belong to the 'phage' integrase family. This region is a possible
                     MT-complex-specific genomic island (See Becq et al.,2007)."
                     /codon_start=1
                     /transl_table=11
                     /product="Possible integrase (fragment)"
                     /protein_id="NP_216825.1"
                     /db_xref="GI:15609446 GeneID:885133"
                     /translation="MTGAGIVETTTNRVRHVPVPEPVSERLRDELPTEPNALVFPSYRGGHLPIEEYRRAFDKGCKAVGIADLVP
                     HGLRHTTASLAISAGANVKVVQRLLGHATAAMTLDRHGHLLSDDLAGVAGLLVQAIKSAAASLRYSDPDSVAVENISAAS"
     CDS             2752126..2753124
                     /locus_tag="Rv2646"
                     /note="Rv2646, (MTCY441.16), len: 332 aa. Probable integrase, similar to others e.g.
                     P06723|VINT_BP186|int integrase from Bacteriophage 186 (336 aa)s FASTA scores: opt:
                     198, E(): 6.3e-05, (30.45% identity in 138 aa overlap). Could be belong to the
                     'phage' integrase family."
                     /codon_start=1
                     /transl_table=11
                     /product="Probable integrase"
                     /protein_id="NP_217162.1"
                     /db_xref="GI:15609783 GeneID:887706"
                     /translation="MNTATRVRLARKRADRLNLKLIKNGHHFRLRDADEITLAVGHLGVVEAFLAAAKSQNKPPGPPPSLHAPPS
                     WRRDIDDYLLNLNAAGQRPATIRLRKTVLCAAAHGLGRPPADVTAEHLLDWLGKQQHLSPEGRKTYRSTLRGFFVWAYEMDRVRD
                     YVADSLPKVRCPKQPPRPAGDDVWQAALAKADRRIELMIRLAGEAGLRRAEAAQAHTGDLMDGGLLLVHGKGGKRRIVPISDYLA
```

```
                     ALIRDTPHGYLFPNGTGGHLTAEHVGKLVSRALPGDATMHTLRHRYATRAYRGSHNLRAVQQLLGHASIVTTERYTALCDDEVRA
                     AAAAAW"
     CDS             complement(2754015..2755454)
                     /locus_tag="Rv2650c"
                     /experiment="EXISTENCE: identified in proteomics study"
                     /note="Rv2650c, (MTCY441.19), len: 479 aa. Possible phiRv2 prophage protein (capsid
                     subunit) (see citation below),highly similar to O06614|Rv1576c|MTCY336.28 probable
                     phiRv1 phage protein from Mycobacterium tuberculosis (473 aa),FASTA scores: opt:
                     2782, E(): 2.8e-159, (89.1% identity in 468 aa overlap)."
                     /codon_start=1
                     /transl_table=11
                     /product="Possible PhiRv2 prophage protein"
                     /protein_id="NP_217166.1"
                     /db_xref="GI:15609787 GeneID:887478"
                     /translation="MTNEQHFADDGDIKQLSLDETRSAAKQLLDSVEGDLTGDVAQRFQALTRHAEELRAEQRRRGREAEEALRR
                     CRAGELRVVPGAPTGGDDGDAPPGNSLRDIAFRTLDVCVRDGLMSSRAAEAAETLCRTGPPQSTSWAQRWLAATGNRDYLGAFVK
                     RVSNPVAGHTTWTDREAAAWREAAAVAAEQRAMGLVDTAGGFLIPAALDPAILLSGDGSTNPIRQVARVVQTTSEVWRGVTSEGA
                     EAHWYSEAQEVSDDSPTLAQPAVPSYRGSCWIPFSLEIEGDAAGFVAEVGRVLADSVEQLQAAAFVSGSGNGEPTGFVSALTGTA
                     DYTVTGAGTEAVVAADVYALQSALPPRFQSNSAFAANLSTINVLRQAETANGALKFPSLHASPPMLAGKHIWEVSNMDTVDAAVT
                     ATNYPLVLGDWKQFIITDRVGSTVELVPHVFGGNRRPTGQRGFFCWFRVGSDVLVDNAFRVLKVQTTA"
     CDS             complement(2755462..2755995)
                     /locus_tag="Rv2651c"
                     /note="Rv2651c, (MTCY441.20c), len: 177 aa. Possible protease protein, phiRv2 phage
                     protein (prohead protease) (see citation below), showing some similarity with several
                     proteases e.g. Q9A4P4|CC2786 putative protease from Caulobacter crescentus (138 aa),
                     FASTA scores: opt: 206,E(): 2e-06, (36.35% identity in 132 aa overlap); Q9RNH0
                     putative prohead protease from Rhodobacter capsulatus (Rhodopseudomonas capsulata)
                     (184 aa), FASTA scores: opt: 196, E(): 1.1e-05, (35.05% identity in 137 aa overlap);
                     BAB35014|ECS1591 putative prohead protease from Escherichia coli strain O157:H7 (185
                     aa), FASTA scores: opt: 187, E(): 4.1e-05, (32.9% identity in 158 aa overlap); etc.
                     And highly similar to O06613|Rv1577c|MTCY336.27 Probable phiRV1 phage protein from
                     Mycobacterium tuberculosis (170 aa),FASTA scores: opt: 987, E(): 2.3e-56, (89.35%
                     identity in 169 aa overlap)."
                     /codon_start=1
                     /transl_table=11
                     /product="Possible PhiRv2 prophage protease"
                     /protein_id="NP_217167.1"
                     /db_xref="GI:15609788 GeneID:887837"
                     /translation="MSSILFRTAELRPGEGRTVYGVIVPYGEVTTVRDLDGEFREMFAPGAFRRSIAERGHKVKLLVSHDARTRY
                     PVGRAVELREEPHGLFGAFELANTPDGDEALANVKAGVVDAFSVGFRPIRDRREGDVIVRVEAALLEVSLTGVPAYLGAQIAGVR
                     AESLAVVSRSLAEARLALMDW"
     CDS             complement(2756148..2756774)
                     /locus_tag="Rv2652c"
                     /note="Rv2652c, (MTCY441.21c), len: 208 aa. Probable phiRv2 phage protein (terminase)
                     (see citation below), showing some similarity with AAK79859|Q97HW1|CAC1896 phage
                     terminase-like protein (small subunit) from Clostridium acetobutylicum (151 aa),
                     FASTA scores: opt: 155, E(): 0.012, (24.7% identity in 158 aa overlap); and Q9B019
                     hypothetical 17.8 KDA protein from Bacteriophage GMSE-1 (159 aa), FASTA scores: opt:
                     141, E(): 0.087, (27.65% identity in 159 aa overlap). Also highly similar to
                     O06612|Rv1578c|MTCY336.26 Probable phiRV1 phage protein from Mycobacterium
                     tuberculosis (156 aa), FASTA scores: opt: 448, E(): 1.2e-20, (48.1% identity in 156
                     aa overlap). Equivalent to AAK47043 from Mycobacterium tuberculosis strain CDC1551
                     but longer 45 aa."
                     /codon_start=1
                     /transl_table=11
                     /product="Probable PhiRv2 prophage protein"
                     /protein_id="NP_217168.1"
                     /db_xref="GI:15609789 GeneID:888577"
                     /translation="MPSPATARPDTATVGERVRAQVLWGVFWHHGIRDPKPGKRRVVLKMGRRGPAPAPAQLKLLGGRSPGRDSG
                     GRRVTPPAAFERVAPECPDWLPPGAKDMWGRVVPELAALNLLKESDLGVLTSFCVAWDQLMQAVTAYREQGFIATNARSRRVTVH
                     PAVAAARAATRDVLVLARELGCTPSAEANLAAVLAAAGDPDDDEFNPFAPDR"
     CDS             complement(2756806..2757129)
                     /locus_tag="Rv2653c"
                     /note="Rv2653c, (MTCY441.22c), len: 107 aa. Hypothetical unknown protein, possibly
                     phiRv2 phage protein (see citation below)."
                     /codon_start=1
                     /transl_table=11
                     /product="Possible PhiRv2 prophage protein"
                     /protein_id="NP_217169.1"
```

```
                     /db_xref="GI:15609790 GeneID:887367"
                     /translation="MTHKRTKRQPAIAAGLNAPRRNRVGRQHGWPADVPSAEQRRAQRQRDLEAIRRAYAEMVATSHEIDDDTAE
                     LALLSMHLDDEQRRLEAGMKLGWHPYHFPDEPDSKQ"
     CDS             complement(2757451..2758878)
                     /locus_tag="Rv2655c"
                     /experiment="EXISTENCE: identified in proteomics study"
                     /note="Rv2655c, (MTCY441.24c), len: 475 aa. Hypothetical protein, possibly phiRv2
                     phage protein (putative primase-like protein) (see citation below). C-terminus
                     similar to P22875|YXIS_SACER hypothetical 28.9 KDA protein (probably does not play a
                     direct role in plasmid integration or excision) from Saccharopolyspora erythraea
                     (Streptomyces erythraeus) plasmid pSE211 (263 aa), FASTA scores: opt: 389, E():
                     2.7e-15, (33.45% identity in 269 aa overlap). Weak similarity in N-terminus to
                     O06608|MTCY336.22|Rv1582c Probable phiRV1 phage protein from Mycobacterium
                     tuberculosis (471 aa), FASTA scores: opt: 133, E(): 2.5, (36.0% identity in 75 aa
                     overlap)."
                     /codon_start=1
                     /transl_table=11
                     /product="Possible PhiRv2 prophage protein"
                     /protein_id="NP_217171.1"
                     /db_xref="GI:15609792 GeneID:887388"
                     /translation="MADIPYGRDYPDPIWCDEDGQPMPPVGAELLDDIRAFLRRFVVYPSDHELIAHTLWIAHCWFMEAWDSTPR
                     IAFLSPEPGSGKSRALEVTEPLVPRPVHAINCTPAYLFRRVADPVGRPTVLYDECDTLFGPKAKEHEEIRGVINAGHRKGAVAGR
                     CVIRGKIVETEELPAYCAVALAGLDDLPDTIMSRSIVVRMRRRAPTEPVEPWRPRVNGPEAEKLHDRLANWAAAINPLESGWPAM
                     PDGVTDRRADVWESLVAVADTAGGHWPKTARATAETDATANRGAKPSIGVLLLRDIRRVFSDRDRMRTSDILTGLNRMEEGPWGS
                     IRRGDPLDARGLATRLGRYGIGPKFQHSGGEPPYKGYSRTQFEDAWSRYLSADDETPEERDLSVSAVSAVSPPVGDPGDATGATD
                     ATDLPEAGDLPYEPPAPNGHPNGDAPLCSGPGCPNKLLSTEAKAAGKCRPCRGRAAASARDGAR"
     CDS             complement(2758880..2759272)
                     /locus_tag="Rv2656c"
                     /note="Rv2656c, (MTCY441.25c), len: 130 aa. Probable phiRv2 phage protein (see
                     Hatfull 2000), highly similar to O06607|YF83_MYCTU|Rv1583c|MT3573.2|MTCY336.21
                     Probable phiRV1 phage protein from Mycobacterium tuberculosis (132 aa), FASTA scores:
                     opt: 734, E(): 2.5e-39, (81.5% identity in 131 aa overlap); and some similarity with
                     Q982T4|MLL8506 hypothetical protein from Rhizobium loti (Mesorhizobium loti) (204
                     aa), FASTA scores: opt: 104, E(): 9.7, (31.85% identity in 113 aa overlap)."
                     /codon_start=1
                     /transl_table=11
                     /product="Possible PhiRv2 prophage protein"
                     /protein_id="NP_217172.1"
                     /db_xref="GI:15609793 GeneID:888179"
                     /translation="MTAVGGSPPTRRCPATEDRAPATVATPSSTDPTASRAVSWWSVHEYVAPTLAAAVEWPMAGTPAWCDLDDT
                     DPVKWAAICDAARHWALRVETCQAASAEASRDVSAAADWPAVSREIQRRRDAYIRRVVV"
     CDS             complement(2759269..2759529)
                     /locus_tag="Rv2657c"
                     /note="Rv2657c, (MTCY441.26c), len: 86 aa. Probable phiRv2 phage protein
                     (excisionase) (see citation below), similar to O22001|VG36_BPMD2|36|G2 gene 36
                     protein (GP36) from Mycobacteriophage D29 (56 aa), FASTA scores: opt: 171, E():
                     9.6e-06, (48.0% identity in 50 aa overlap); and Q05246|VG36_BPML5|36 gene 36 protein
                     (GP36) from Mycobacteriophage L5 (56 aa), FASTA scores: opt: 169, E(): 1.3e-05, (50%
                     identity in 50 aa overlap). Similarity suggests alternative start at 21737. Contains
                     possible helix-turn-helix motif from aa 33 to 54 (Score 1655, +4.82 SD)."
                     /codon_start=1
                     /transl_table=11
                     /product="Probable PhiRv2 prophage protein"
                     /protein_id="NP_217173.1"
                     /db_xref="GI:15609794 GeneID:887399"
                     /translation="MCAFPSPSLGWTVSHETERPGMADAPPLSRRYITISEAAEYLAVTDRTVRQMIADGRLRGYRSGTRLVRLR
                     RDEVDGAMHPFGGAA"
     CDS             complement(2759911..2761038)
                     /locus_tag="Rv2659c"
                     /note="Rv2659c, (MTCY441.28c), len: 375 aa. Probable integrase, phiRv2 phage protein:
                     putative member of the phage integrase family of tyrosine recombinases (see Hatfull
                     2000), highly similar to others e.g. P22884|VINT_BPML5|33|int from Mycobacteriophage
                     L5 (371 aa), FASTA scores: opt: 836, E(): 1.2e-44, (39.0% identity in 372 aa
                     overlap); Q38361|VINT_BPMD2|33|int from Mycobacteriophage D29 (333 aa), FASTA scores:
                     opt: 786,E(): 1.4e-41, (40.55% identity in 338 aa overlap); etc. Seems belongs to the
                     'phage' integrase family."
                     /codon_start=1
                     /transl_table=11
                     /product="Probable PhiRv2 prophage integrase"
```

```
                     /protein_id="NP_217175.1"
                     /db_xref="GI:15609796 GeneID:885098"
                     /translation="MTQTGKRQRRKFGRIRQFNSGRWQASYTGPDGRVYIAPKTFNAKIDAEAWLTDRRREIDRQLWSPASGQED
                     RPGAPFGEYAEGWLKQRGIKDRTRAHYRKLLDNHILATFADTDLRDITPAAVRRWYATTAVGTPTMRAHSYSLLRAIMQTALADD
                     LIDSNPCRISGASTARRVHKIRPATLDELETITKAMPDPYQAFVLMAAWLAMRYGELTELRRKDIDLHGEVARVRRAVVRVGEGF
                     KVTTPKSDAGVRDISIPPHLIPAIEDHLHKHVNPGRESLLFPSVNDPNRHLAPSALYRMFYKARKAAGRPDLRVHDLRHSGAVLA
                     ASTGATLAELMQRLGHSTAGAALRYQHAAKGRDREIAALLSKLAENQEM"
     CDS             3854913..3855128
                     /locus_tag="Rv3751"
                     /note="Rv3751, (MTV025.099), len: 71 aa. Probable integrase (fragment), similar to
                     part of many e.g. Q48908 integrase (fragment) from Mycobacterium paratuberculosis
                     (191 aa),FASTA scores: opt: 206, E(): 5.5e-08, (57.65% identity in 59 aa overlap);
                     Q9ZWV7|int integrase from Corynephage 304L (395 aa), FASTA scores: opt: 156, E():
                     0.00036, (45.75% identity in 59 aa overlap); Q9K722|BH3551 integrase (phage-related
                     protein) from Bacillus halodurans (378 aa),FASTA scores: opt: 151, E(): 0.00079,
                     (46.15% identity in 52 aa overlap); etc. Also similarity with various conjugative
                     transposons. Also similar to Mycobacterium tuberculosis hypothetical proteins e.g.
                     P71903|Rv2309c|MTCY3G12.25 (151 aa), FASTA scores: opt: 193, E(): 3.8e-07, (50.85%
                     identity in 59 aa overlap); O53403|Rv1055|MTV017.08 (78 aa), FASTA scores: opt:
                     171,E(): 7.8e-06, (54.15% identity in 48 aa overlap); etc. This region is a possible
                     MT-complex-specific genomic island (See Becq et al., 2007)."
                     /codon_start=1
                     /transl_table=11
                     /product="Probable integrase (fragment)"
                     /protein_id="NP_218268.1"
                     /db_xref="GI:15610887 GeneID:885857"
                     /translation="MKRAKVQQITPHDLRHTAASLAVSAGVNVLALQRILGHKSAKVTLDTYADLFDADLDAVAVTLGKDADQQT"
```
